# Chromosome-level genome assembly of the Pacific geoduck *Panopea generosa* reveals major inter- and intrachromosomal rearrangements and substantial expansion of the copine gene family

**DOI:** 10.1093/gigascience/giad105

**Published:** 2023-12-19

**Authors:** Jing Wang, Qing Xu, Min Chen, Yang Chen, Chunde Wang, Nansheng Chen

**Affiliations:** CAS Key Laboratory of Marine Ecology and Environmental Sciences, Institute of Oceanology, Chinese Academy of Sciences, Qingdao 266071, China; Laboratory of Marine Ecology and Environmental Science, Qingdao National Laboratory for Marine Science and Technology, Qingdao 266237, China; Center for Ocean Mega-Science, Chinese Academy of Sciences, Qingdao 266071, China; CAS Key Laboratory of Marine Ecology and Environmental Sciences, Institute of Oceanology, Chinese Academy of Sciences, Qingdao 266071, China; Laboratory of Marine Ecology and Environmental Science, Qingdao National Laboratory for Marine Science and Technology, Qingdao 266237, China; Center for Ocean Mega-Science, Chinese Academy of Sciences, Qingdao 266071, China; Research and Development Center for Efficient Utilization of Coastal Bioresources, Yantai Institute of Coastal Zone Research, Chinese Academy of Sciences, Yantai 264003, China; CAS Key Laboratory of Marine Ecology and Environmental Sciences, Institute of Oceanology, Chinese Academy of Sciences, Qingdao 266071, China; Laboratory of Marine Ecology and Environmental Science, Qingdao National Laboratory for Marine Science and Technology, Qingdao 266237, China; Center for Ocean Mega-Science, Chinese Academy of Sciences, Qingdao 266071, China; Research and Development Center for Efficient Utilization of Coastal Bioresources, Yantai Institute of Coastal Zone Research, Chinese Academy of Sciences, Yantai 264003, China; Marine Science and Engineering College, Qingdao Agricultural University, Qingdao 266109, China; CAS Key Laboratory of Marine Ecology and Environmental Sciences, Institute of Oceanology, Chinese Academy of Sciences, Qingdao 266071, China; Laboratory of Marine Ecology and Environmental Science, Qingdao National Laboratory for Marine Science and Technology, Qingdao 266237, China; Center for Ocean Mega-Science, Chinese Academy of Sciences, Qingdao 266071, China; Department of Molecular Biology and Biochemistry, Simon Fraser University, Burnaby, British Columbia V5A 1S6 , Canada

**Keywords:** *Panopea generosa*, chromosome-level genome assembly, genetic breeding, evolutionary adaptation

## Abstract

The Pacific geoduck *Panopea generosa* (class Bivalvia, order Adapedonta, family Hiatellidae, genus *Panopea*) is the largest known burrowing bivalve with considerable commercial value. Pacific geoduck and other geoduck clams play important roles in maintaining ecosystem health for their filter feeding habit and coupling pelagic and benthic processes. Here, we report a high-quality chromosome-level genome assembly of *P. generosa* to characterize its phylogeny and molecular mechanisms of its life strategies. The assembled *P. generosa* genome consists of 19 chromosomes with a size of 1.47 Gb, a contig N50 length of 1.6 Mb, and a scaffold N50 length of 73.8 Mb. The BUSCO test of the genome assembly showed 93.0% completeness. Constructed chromosome synteny revealed many occurrences of inter- and intrachromosomal rearrangements between *P. generosa* and *Sinonovacula constricta*. Of the 35,034 predicted protein-coding genes, 30,700 (87.6%) could be functionally annotated in public databases, indicating the high quality of genome annotation. Comparison of gene copy numbers of gene families among *P. generosa* and 11 selected species identified 507 rapidly expanded *P. generosa* gene families that are functionally enriched in immune and gonad development and may be involved in its complex survival strategies. In particular, genes carrying the copine domains underwent additional duplications in *P. generosa*, which might be important for neuronal development and immune response. The availability of a fully annotated chromosome-level genome provides a valuable dataset for genetic breeding of *P. generosa*.

## Introduction

The Pacific geoduck *Panopea generosa* (NCBI:txid1049056; marinespecies.org:taxname:545994) is one member of genus *Panopea*, which includes the world's largest burrowing bivalves. *P. generosa* is usually found in low intertidal and subtidal sediments throughout the northeast Pacific Coast, including the United States (Alaska, Washington, and California), Canada (British Columbia), and Mexico (north Baja Pacific Coast) [1, 2]. Geoducks can reach more than 25 cm in shell length and more than 100 cm in siphon length [3]. Geoduck adults are usually buried in muddy-sandy sediment at depths ranging from 60 to 100 cm, with only their siphon tips exposed to respire, capture food, and release secretion/excretion products and gametes. The sedentary behavior may contribute to their long life spans, which can be as long as 168 years for *P. generosa* [4]. Due to these unique life strategies, it is expected that geoduck should have distinctive growth and development mechanisms, especially in relation to benthic life and immune system.

Geoduck clams play important roles in maintaining ecosystem health for their filter-feeding habit and coupling pelagic and benthic processes by ejecting undigested mucus-bound feces and pseudo feces to the sediment surface. They are prey for sea otters, fishes, crabs, and sea stars [5, 6]. As marine calcifiers, shell concentrations of *Panopea* inside Scalichnus burrows have been analyzed to reconstruct the sequence of events related to storm events [7]. Geoduck clams possess great commercial fishery value in Canada and the United States [8]. Since the recruitment of geoducks has been low due to overfishing and their vulnerability to environmental changes [9, 10], there has been an increasing interest in genetic breeding of geoducks.

Bivalves are an ancient lineage of bilaterian and are a diverse class of Mollusca. To date, the chromosome-level genomes of only about 40 bivalve species have been assembled [11]. These genomes can provide a resource for comparative genomics and for gaining evolutionary and other insights into bivalves and molluscs. These genomes show a remarkable level of diversity. For example, the assembled genome sizes of bivalves vary widely, ranging from 543.9 Mb in *Lutraria thynchaena* [12] to 2.6 Gb in *Modiolus philippinarum* [13] (Supplementary Table S1). Among bivalves, the genome sizes of most superorder Imparidentia species range from 1 to 1.8 Gb, and that of the species in the order Adapedonta, which includes *P. generosa*, ranges from 1 to 1.5 Gb [14].

The numbers of chromosomes also vary substantially among bivalves, suggesting active genome recombination during the evolution of bivalves [15]. While some species of the order Ostreida, including *Crassostrea gigas* [16], *Crassostrea virginica, Crassostrea hongkongensis* [17], *Crassostrea ariakensis* [18], *Crassostrea angulate*, and *Ostrea edulis*, have 10 chromosomes, the species of the order Pterioidea and Mytilida have 14 to 15 chromosomes, such as *Pinctada fucata* [19], *Mytilus coruscus* [20], and *Limnoperna fortunei* (Supplementary Table S1). The chromosome numbers of the order Myida vary from 16 to 17. Interestingly, the chromosome number of species in most other orders is 19, including Venerida, Cardiida, Unionida, Arcida, and Pectinida. The chromosome number of all reported species in the order Adapedonta, which includes *P. generosa*, is also 19.

Nevertheless, a high-quality chromosome-level reference genome of *P. generosa* is currently not available, hindering the development of geoduck genetic breeding programs. In this study, we report the first chromosome-scale genome assembly for *P. generosa* generated using cutting-edge technologies, including next-generation sequencing, long-read sequencing, and high-throughput chromosome conformation capture (Hi-C) technologies. We further performed gene family clustering, phylogenetic analysis, and gene family expansion and contraction analysis to understand its adaptation, growth, development, and immunity. The availability of this genome information will facilitate research in molecular evolution and genetic breeding.

## Results

### Genome sequencing and assembly

For the genome assembly of *P. generosa*, short reads were obtained for estimating the genome size, heterozygosity rate, and repeat content; long reads were obtained for initial genome assembly; and Hi-C reads were obtained for the construction of chromosomes (Table 1).

**Table 1:** Statistics of the DNA sequence data used for *P. generosa* genome assembly

Source	Platform	Library size	Clean data (Gb)	Read length (bp)	Sequencing coverage (×)
Genome-short reads	BGISEQ-500	300 bp	258.19	150	181
Genome-long reads	PacBio sequel	20 Kb	164.46	26,513*	115
Hi-C	BGISEQ-500	300 bp	233.49	150	163

* “26,513” indicates the N50 of subreads.

**Figure 1:**
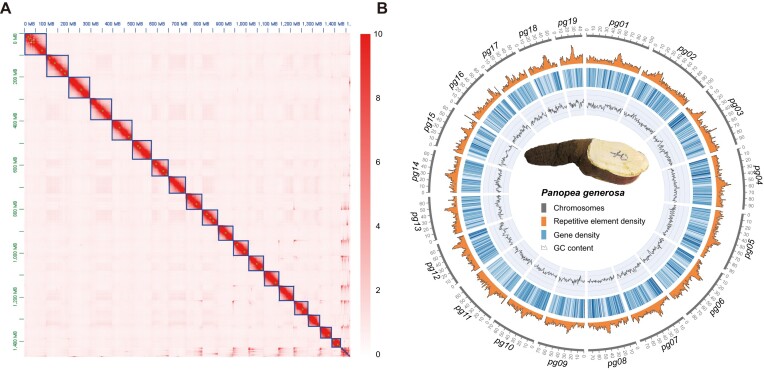
The *P. generosa* genome construction and and genome landscape. (A) Hi-C analysis of *P. generosa* genome contigs. Chromosomes are arranged in the size order from left to right and from top to bottom. The color bar illuminates the logarithm of the contact density from red (10) to white (0) in the plot. (B) The genomic landscape of *P. generosa* (from outer to inner circles): a, the 19 chromosomes; b–d, repetitive element density, gene density, and GC density across the genome, respectively, drawn in 1-Mb nonoverlapping windows.

Genome size, heterozygosity rate, and repeat content of *P. generosa* estimated by the *k*-mer analysis [21] of the short reads were 1.47 Gb, 1.37%, and 68.08%, respectively (Supplementary Fig. S1 and Supplementary Table S2). The assembled genome of *P. generosa*, which was generated using Pacific Biosciences (PacBio) long reads (N50 length = 26,513 bp) with Falcon and Hi-C data, consisted of 19 pseudomolecules (Fig. 1; Supplementary Table S3), with a contig anchoring rate of 94.70%. The assembled *P. generosa* genome has a total length of 1.47 Gb (1,474,161,289 bp) with a contig N50 length of 1.6 Mb and a scaffold N50 length of 73.79 Mb (Table 2).

**Table 2:** Statistics of the genome assembly of *P. generosa*

Statistics	Contig	Scaffold	Chromosome
Total number	2,086	39	19
Total length (bp)	1,473,137,789	1,474,161,289	1,432,060,667
Average length (bp)	706,202	37,799,007	75,371,614
N50 length (bp)	1,571,249	73,788,920	76,670,739
N90 length (bp)	418,579	53,843,121	53,843,121
Maximum length (bp)	6,469,558	101,196,518	101,196,518
Minimum length (bp)	17	29,000	1,432,060,667
GC content (%)	34.33	34.33	34.33
Anchored rate (%)	94.70		

The heterozygosity rate of *P. generosa* (1.37%) fell within the range of the heterozygosity rates of bivalves whose genomes have been sequenced and assembled, which vary broadly from 0.11% in *Margaritifera margaritifera* [22] to 3.20% in *Crassostrea gigas* [23]. The heterozygosity rate of *P. generosa* (1.37%) was close to that of another burrowing bivalve, *Sinonovacula constricta* (1.55%), and a deep-sea mussel, *Bathymodiolus platifrons* (1.03–1.24%) [13, 24] (Supplementary Table S4).

### Genome annotation and evaluation

The majority (57.99%) of the *P. generosa* genome was estimated to be repetitive elements (Table 3), which is similar to but lower than the predicted content of repetitive elements using *k*-mer analysis (68.08%). The top 3 most frequent categories of repetitive elements in the *P. generosa* genome were DNA transposons (21.6%), long interspersed nuclear elements (LINEs, 9.1%), and long terminal repeats (LTRs, 3.76%) (Table 3).

**Table 3:** Repetitive element annotations in *P. generosa*

	Type	Length (bp)	% in genome
**Transposable elements**	DNA	318,426,608	21.601
	LINE	134,171,392	9.102
	SINE	27,249,093	1.848
	LTR	55,380,608	3.756
	Other	31,141	0.002
	Unknown	151,891,763	10.304
	Total	583,000,390	39.548
**Tandem repeat**		347,641,207	23.582
**Total**		854,873,570	57.991

A total number of 35,034 protein-coding genes (PCGs) were annotated in the *P. generosa* genome. The average gene length, average coding sequence (CDS) length, average number of exons per gene, average exon length, and average intron length were 13,469 bp, 1,273 bp, 6,220 bp, and 2,171 bp, respectively. Of these PCGs, 30,700 (87.6%) had functional annotations assigned using comparisons to public databases, including NCBIs nr, Swiss-Prot, KEGG, KOG, TrEMBL, InterPro, and Gene Ontology (Supplementary Table S5), supporting the high quality of the genome.

The distribution of repetitive elements was highly uneven, with regional content of the repetitive elements varying from 34.76% to 84.89% in the *P. generosa* genome, with peaks occurring at regions with low gene density (Fig. 1B).

To evaluate the completeness of the assembly, the *P. generosa* genome was tested using BUSCO [25] with the metazoa_odb10 database (954 core genes). We found that 93.0% of the core genes used by BUSCO were identified as full length in the *P. generosa* genome (Table 4), which is higher than that of *S. constricta* (91.5%) [14] (Supplementary Table S6). We further tested the completeness of the predicted PCGs using BUSCO, which identified 88.4% of the core genes as full length, suggesting that quality of the predicted genes can be further improved in the future.

**Table 4:** BUSCO results for analysis of genome completeness for *P. generosa*

Type	Genome assembly		Gene set	
	Number of genes	Percentage	Number of genes	Percentage
Complete BUSCOs (C)	887	93.0	844	88.4
Complete and single-copy BUSCOs (S)	842	88.3	795	83.3
Complete and duplicated BUSCOs (D)	45	4.7	49	5.1
Fragmented BUSCOs (F)	34	3.6	49	5.1
Missing BUSCOs (M)	33	3.4	61	6.5
Total BUSCO groups	954	100	954	100

### Chromosomal synteny analysis between *P. generosa* and *S. constricta*

**Figure 2:**
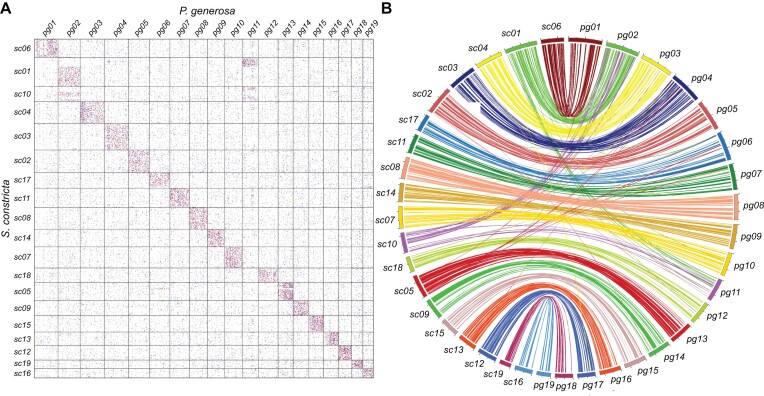
Chromosome synteny of *P. generosa* and *S. constricta*. (A) A dotplot displaying correspondence of homologous genes in *P. generosa* and *S. constricta*. The red, blue, and gray dots present 3 similarity levels of homologous gene pairs between *P. generosa* and *S. constricta*. The best-matched genes are shown in red, while the next best-matched genes are in blue and the least best-matched genes in gray. (B) A CIRCOS plot of synteny analysis between *P. generosa* and *S. constricta*. Lines in different colors depict different interchromosomal synteny.

Comparative analysis of genome-wide gene collinearity revealed a high chromosome synteny between *P. generosa* and *S. constricta*. Of the 19 *P. generosa* chromosomes, 17 chromosomes were found to have one-to-one correspondences with 17 *S. constricta* chromosomes (Fig. 2). However, several large-scale interchromosomal rearrangements were also identified, such as the arrangement among *P. generosa* chromosomes *pg02* and *pg11* and *S. constricta* chromosomes *sc01* and *sc10* (Fig. 2). Despite these large-scale interchromosomal rearrangements, the number of chromosomes in these 2 species is still 19.

In addition to these major interchromosomal rearrangement events, comparative analysis of these 2 genomes also revealed extensive intrachromosomal rearrangements, which resulted in low gene synteny within these chromosomes. Instead of a clear diagonal linear relationship between genes of these 2 species (*P. generosa* and *S. constricta*), a near-random scattering of the relationships was observed (Fig. 2A). These intrachromosomal rearrangement events are also clearly shown in Fig. 2B, such as *P. generosa* chromosome *pg01* versus *S. constricta* chromosome *sc06*.

### Evolutionary analysis of *P. generosa* and other bivalves

**Figure 3:**
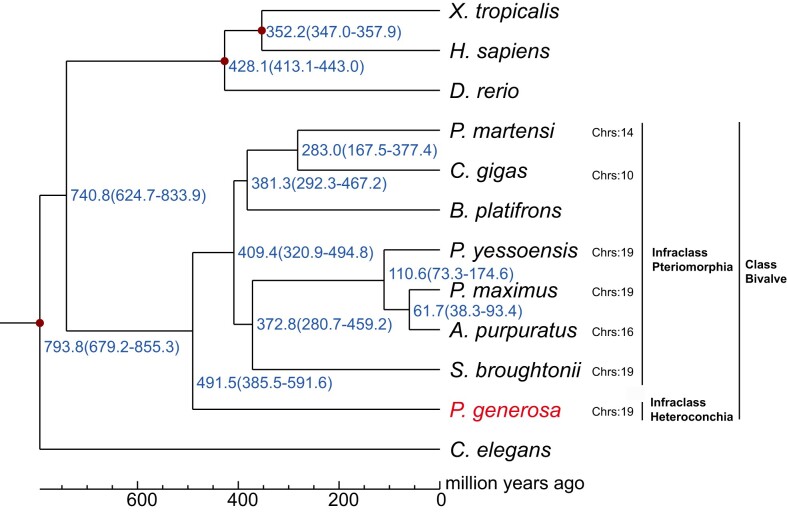
Phylogenetic analysis of *P. generosa* with related species. The estimated species divergence time (million years ago) and the 95% confidential intervals are labeled at each branch site. Divergence times used for time recalibration were illuminated as red dots in the tree. Numbers of chromosomes of each Bivalvia species are shown.

Phylogenetic analysis using 326 one-to-one single-copy orthologous genes from 12 species showed that *P. generosa* was tightly clustered with other bivalves as expected (Fig. 3). According to the phylogenetic tree, the divergence time of *P. generosa* from its nearest node, which represents the common ancestor of many other bivalves, was approximately 491.5 million years ago (Mya) (Fig. 3). This divergence time between *P. generosa* and other bivalves was similar to the divergence time between *S. constricta* and other bivalves [14]. Interestingly, the numbers of chromosomes of bivalves vary substantially, and closely related bivalves can have different numbers of chromosomes (Fig. 3). The oysters *C. gigas* with 10 chromosomes and *P. martensi* with 14 chromosomes diverged from the clam *S. broughtoni* and scallops with 19 chromosomes 409.4 Mya. In addition, the scallop *A. purpuratus* with 16 chromosomes diverged from another scallop, *P. maximus*, with 19 chromosomes 61.7 Mya.

### Comparative analysis of gene families

**Figure 4:**
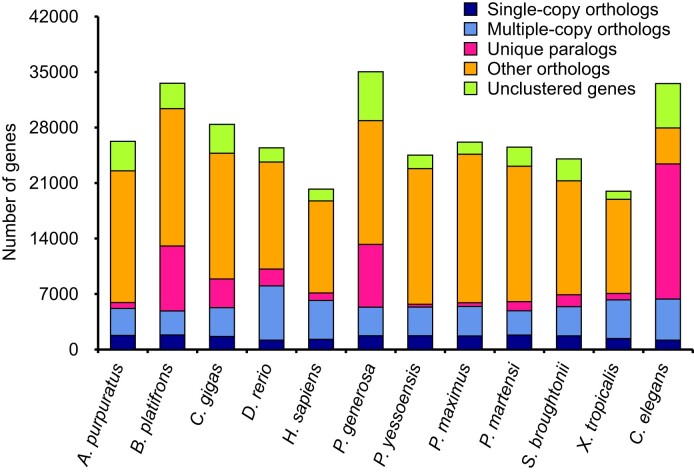
The distribution of single-copy orthologs, multiple-copy orthologs, unique paralogs, other orthologs, and unclustered genes in *P. generosa* and related species.

**Figure 5:**
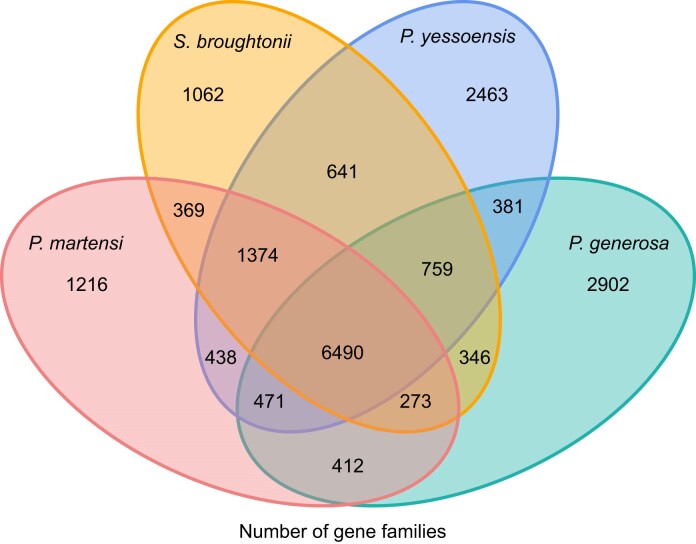
Distribution of shared gene families among *P. generosa, P. martensi, S. broughtonii*, and *P. yessoensis*. Intersections between species indicate the numbers of shared gene families, whereas unique family numbers are shown in species-specific areas. The center represents the number of families shared by all the 4 species.

In total, 30,616 gene families were identified among *P. generosa* and 11 other species (*Pinctada martensi, C. gigas, B. platifrons, Patinopecten yessoensis, Pecten maximus, Argopecten purpuratus, Scapharca broughtonii, Homo sapiens, Xenopus tropicaalis, Danio rerio*, and *Caenorhabditis elegans*) (Table 5, Fig. 4, Supplementary Table S7). As compared with the other 11 species, 7,917 genes belonging to 1,749 gene families were found to be *P. generosa* specific, which fell into the range of 1,567 to 15,051 species-specific gene families identified in 12 bivalves [26]. Comparative analysis of the PCGs of *P. martensi, S. broughtonii, P. yessoensis*, and *P. generos*a revealed 6,490 common gene families shared by these species and 2,902 gene families specific to *P. generosa* (Fig. 5).

**Table 5:** The PCGs of *P. generosa* and 11 other species for evolutionary analysis

Species	Total genes	Unclustered genes	Families	Unique families	Average genes per family
*P. generosa*	35,034	6,168	12,034	1,749	2.4
*A. purpuratus*	26,256	3,720	13,196	290	1.71
*B. platifrons*	33,584	3,197	12,409	1,775	2.45
*C. gigas*	28,402	3,638	11,818	828	2.1
*D. rerio*	25,444	1,791	9,210	295	2.57
*H. sapiens*	20,229	1,488	9,251	226	2.03
*P. yessoensis*	24,521	1,704	13,017	137	1.75
*P. maximus*	26,152	1,518	13,276	164	1.86
*P. martensi*	25,526	2,403	11,043	318	2.09
*S. broughtonii*	24,045	2,770	11,314	538	1.88
*X. tropicalis*	19,967	1,016	9,226	159	2.05
*C. elegans*	33,552	5,600	8,201	3,720	3.41

**Figure 6:**
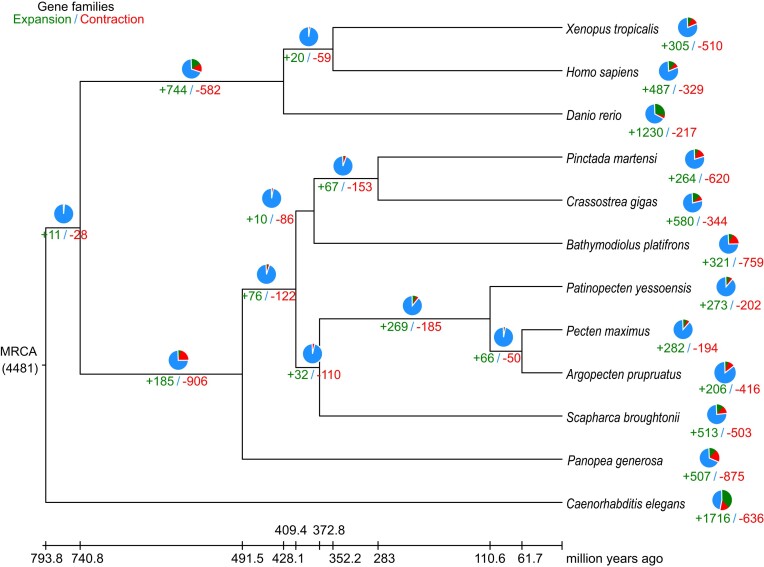
Dynamic evolution and distribution of gene families among *P. generosa* and related species. The numbers of gene gains (+) and losses (−) are shown on the branches, which are also displayed as pie plots: the green part for gene gain, the red part for gene losses, and the blue part for gene remaining. The divergence times are dated and displayed below the phylogenetic tree. MRCA, most recent common ancestor.

A total of 507 rapidly expanded gene families (involving 2,734 genes) and 875 rapidly contracted gene families (involving 792 genes) were identified in the *P. generosa* genome compared to the most recent common ancestor of both *P. generosa* and other 11 species (Fig. 6). The annotation with the KEGG pathway database [27] revealed that the genes of expanded families were distributed in 123 pathways, which were mainly enriched in organismal systems, genes associated with diseases, environmental information processing (e.g., phototransduction), and phosphatidylinositol signaling system, suggesting their important contribution to the adaptation of benthic bivalves. According to the enriched KEGG pathways of expanded gene families in *P. generosa* (Supplementary Table S8), there were a few significant enriched pathways (*Q* < 0.05) related to gonad development—for example, adrenergic signaling in cardiomyocytes and glycine, serine, and threonine metabolism, which have been shown to function as part of spermatogenesis of the fluted giant clam *Tridacna squamosa* [28]. Moreover, oocyte meiosis, apoptosis, Ras signaling pathway, calcium signaling pathway, steroid hormone biosynthesis, gonadotropin-releasing hormone (GnRH) signaling pathway, insulin signaling pathway, oxytocin signaling pathway, and ovarian steroidogenesis were documented to be enriched in *Procambarus clarkii* ovary development [29]. Geoducks have become a focus of significant aquaculture research and development with a considerable commercial value [30, 31]. The enriched gonad development-related pathways and genes could provide basic data for the further genetic breeding research of *P. generosa* and its closely related species.

**Figure 7:**
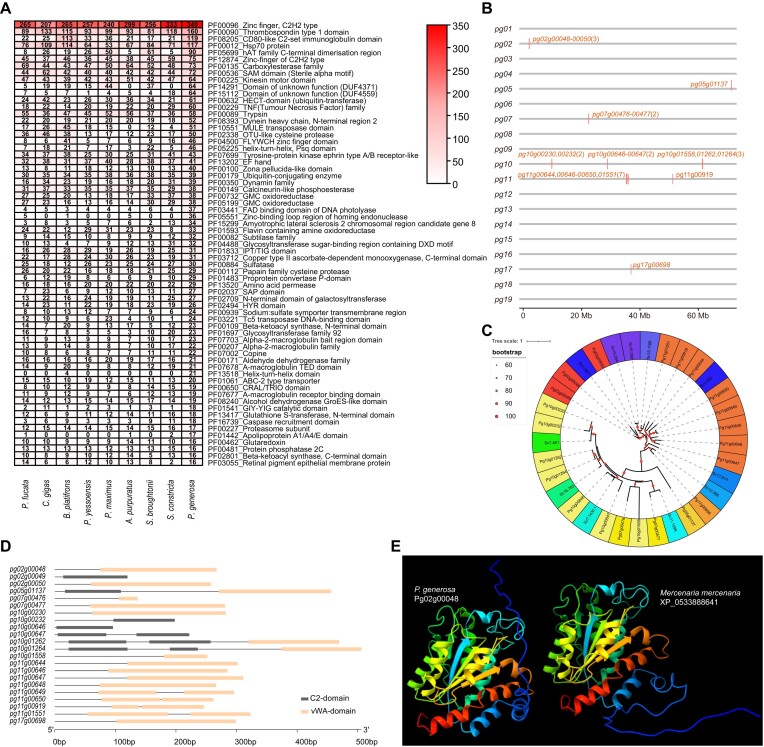
Comparative analysis of gene families based on Pfam annotation in *P. generosa* and related bivalves. (A) The 65 most abundant gene families in which *P. generosa* possesses more gene numbers than 8 other bivalves. (B) Chromosomal distribution of copine genes in *P. generosa genome*. (C) The phylogenetic tree of copine genes in *P. generosa* and *S. constricta*, shown in the background colors of red and pink, respectively. (D) Domain structures of copine genes predicted in *P. generosa* genome. (E) The 3-dimensional structures of Pg02g00048 gene in *P. generosa* and XP_0533888641 gene in *Mercenaria mercenaria*.

We further compared gene families in different bivalves by searching for functional domains contained in PCGs in *P. generosa* and 8 other bivalves using InterProScan [32]. Examination of the top 65 most frequent domains that had specific expansions in *P. generosa* (Fig. 7A) showed that the gene numbers of many important gene families were substantially expanded in *P. generosa*, including these containing the GIY-YIG catalytic domain (PF01541), the caspase recruitment domain (PF16739), the ApoA/ApoE domain (PF01442), and the copine domain (PF07002). In particular, the copy number of the copine gene family, which has been implicated in a range of cell signaling [33], was 22 in *P. generosa*, twice as many as those identified in *S. constricta*. Examination of positions of the *P. generosa* copine genes revealed that they were distributed in chromosomes *pg02, pg05, pg07, pg10, pg11*, and *pg17* (Fig. 7B). Interestingly, many genes formed local clusters (e.g., 8 copies in Pg11), suggesting that the large copine gene set observed in *P. generosa* might have been achieved via tandem duplication of the copine genes in recent evolution. Phylogenetic analysis of the copine genes annotated in *P. generosa* (22 genes) and *S. constricta* (11 genes) revealed good orthologous relationships, as well as one-to-multiple relationships (Fig. 7C), confirming that genes inside copine gene clusters in *P. generosa* (Fig. 7B) were highly similar.

Most of the copine homologs contain the vWA domain, but some have both the C2 domain and the vWA domain in *P. generosa* (Fig. 7D), like copine genes identified in other species [33]. Lengths of the coding sequences of some copies were comparatively short, which might be due to genome assembly or gene prediction errors. Prediction of 3-dimensional structures using AlphaFold2 [34] revealed that their structures are highly conserved among different species, suggesting the conservation of protein functions. For example, the 3-dimensional structure of Pg02g00048 in *P. generosa* showed high similarity to that of XP_0533888641 in *Mercenaria mercenaria* [35] (Fig. 7E).

## Discussion

Through the completion of this project, we have successfully constructed the first high-quality chromosome-level genome assembly of the ecologically and economically important bivalve, the Pacific geoduck *P. generosa*, enriching the expanding list of chromosome-level genomes of bivalves. The *P. generosa* genome represents the third genome of the third species in the order Adapedonta, after *S. constricta* and *Solen grandis*. The *P. generosa* genome assembly represented another instance of a chromosome-level genome assembly from a bivalve species [11], an ancient lineage of bilaterian, and a diverse class of Mollusca (Supplementary Table S1).

Comparative analysis between the genomes of *P. generosa* and its most closely related species, *S. constricta*, revealed extensive inter- and intrachromosomal exchanges. For example, large chromosomal fragments of *P. generosa pg02* and *pg11* matched to 2 *S. constricta* chromosomes (*sc01* and *sc10*), respectively (Fig. 2). Nevertheless, the numbers of chromosomes of both *P. generosa* and *S. constricta* were still identical (19 chromosomes). Within chromosomes, the order of genes showed even more extensive alterations, resulting in the lack of clear diagonal alignments (Fig. 2). Among bivalves, the numbers of chromosomes vary substantially, suggesting active genome recombination during the evolution of bivalves [15]. The chromosome numbers of bivalves in the infraclass Heteroconchia are 16 to 19, relatively higher than those in the infraclass Pteriomorphia, which are 10 to 19. In the infraclass Pteriomorphia, most oysters and scallops possess 19 chromosomes, similar to those of most clams in the infraclass Heteroconchia. In the phylogenetic analysis of the infraclass Pteriomorphia, the blood clam was closer to the scallops, compared with oysters (Fig. 3).

Comparative analysis revealed that the number of PCGs in many gene families with important functions also changes substantially during the evolution of this group (Fig. 7A). In particular, the copine gene family was found to be substantially expanded in *P. generosa*, with 22 copine genes identified. The number of copine genes in *P. generosa* was twice of those in *P. constricta*. Many of these 22 copine genes in *P. generosa* formed tandem clusters, with 1 cluster containing 8 copine genes, suggesting that these genes were formed via tandem duplications. The composition of functional domains, which includes the C2 and vWA domains (Fig. 7D), and the similarity of the 3-dimensional structure to that of known copine genes (Fig. 7E), suggests conservation of function of copine genes in *P. generosa*. Thus, the completion of the genome *P. generosa* facilitates comparative analysis of gene families to uncover important leads for exploring molecular insight into its physiology and evolution.

The successful construction of a chromosome-level genome assembly of *P. generosa* enables not only genomic identification and analysis of important genes in this organism but also comparative analysis of bivalve genomes, which is critical for tracking the species formation, evolution, and biodiversity of bivalves.

## Methods

### Sampling collection

Geoduck *P. generosa* samples were collected from the Strait of Georgia (49°41′12″N, 124°51′33″W) of British Columbia, Canada, in the spring of 2019. The samples showed typical morphological features of *P. generosa*. The identification of the samples was also supported by the high similarities of the molecular marker *cox1* to the reference sequence of *P. generosa* (PID of 99.55, coverage of 100%) [36]. The samples were transferred to a laboratory and kept in a tank with running water for a week. One sample was chosen and dissected on ice to collect tissue samples, including labial palp, heart, foot, gonad, gill, hepatopancreas, siphon, and mantle muscle. This animal was identified to be a female, as indicated by the presence of eggs in the smear of the gonad under a compound microscope. Dissected tissues were quickly frozen in liquid nitrogen and then stored at −80°C before DNA and RNA extraction.

### DNA library construction and sequencing

Genomic DNA of *P. generosa* was extracted using a standard phenol-chloroform extraction method [37]. The quality of DNA was determined by gel electrophoresis to ensure the DNA samples met library sequencing requirements. Sequence libraries with an insert size of 300 bp were constructed for a BGISEQ-500 sequencing platform (RRID:SCR_017979) according to the manufacturer’s protocol. The sequencing data produced were used in the genome size estimation by *k*-mer analysis [21] and for correcting errors in the Pilon (RRID:SCR_014731) assembly [38]. A Hi-C library with an insert size of 300 bp was constructed to provide long-range information (without position information) on the grouping and linear organization of sequences along entire chromosomes to assemble the scaffolds into chromosome-level scaffolds [39]. For Hi-C library construction, gonad tissue was dissociated, and cells were collected and crosslinked with 1% formaldehyde (Sigma) and 0.2 M glycine (Sigma). After that, the fixed powder was resuspended in nuclei isolation buffer and then incubated in 0.5% sodium dodecyl sulfate (SDS) for 10 minutes at 62°C. Then the reaction was quenched with 10% Triton X-100 (Sigma), and the nuclei were collected by centrifugation. Then the DNA was digested with MboI (NEB), and the overhang was filled and biotinylated before being ligated by T4 DNA ligase (NEB). Before library construction, the purified DNA was sheared, and biotin-containing fragments were captured on streptavidin-coated beads using Dynabeads MyOne Streptavidin T1 (Invitrogen). The fragments were then end-repaired and linked with adaptors before 8 cycles of polymerase chain reaction (PCR) with KAPA HiFi HotStart ReadyMix (Kapa Biosystem). After that, the Hi-C library was sequenced with a BGISEQ-500 platform. Also, a PacBio library with an insert size of 20 Kb was constructed to obtain long reads by the PacBio Sequel platform using the Sequel Sequencing Kit 3.0. The adapters and low-quality reads in raw data generated by the BGISEQ platform were cut off by SOAPnuke1.5.6 using the parameter as “-n 0.01 -l 20 -q 0.1 -i -Q 2 -G -M 2 -A 0.5 -d” [40]. PacBio raw data were filtered with the default parameters by using Pacific Biosciences SMRT analysis software (v2.3.1) to filter the low-quality reads.

### RNA library construction and sequencing

RNA sequencing (RNA-seq) and isoform sequencing (Iso-seq) were conducted to obtain transcriptome data to aid genome annotation. The total RNA was extracted by Trizol (Invitrogen) from 8 tissues of the same *P. generosa* individual, including labial palp, heart, foot, gonad, gill, hepatopancreas, siphon, and mantle muscle. The quality and quantity of RNA in each sample were assessed using a NanoDrop and an Agilent 2100 bioanalyzer (Thermo Fisher Scientific). For the construction of messenger RNA (mRNA) libraries for RNA-seq, the mRNA was enriched by mRNA Capture Beads (BGI, LB00V60) and incubated at 85°C for 8 minutes for fragmentation. Reverse transcription was performed with Strand Specificity Reagent and 1st Strand Enzyme Mix (Optimal Dual-mode mRNA Library Prep Kit, BGI, LR00R96) to generate the first-strand complementary DNA (cDNA). After that, the second-strand cDNA generation and end repair were performed with 2nd Strand Buffer and 2nd Strand Enzyme Master Mix. Then, the adaptors (BGI, LA00R04) were ligated to the cDNAs. Next, the library was purified and selected depending on product requirements for amplification. The mRNA libraries were sequenced using the BGISEQ-500 platform. For Iso-seq, the total RNA was extracted from the equally mixed tissues of the 8 tissues above. The PacBio SMRTbell library was prepared using the SMARTer PCR cDNA Synthesis kit (Clontech), the Qubit dsDNA HS Assay Kit 2.0 (Invitrogen), and the Agilent DNA 12000 kit (Agilent Technologies) and sequenced by the PacBio Sequel sequencer (RRID:SCR_017989) with Sequel Sequencing Kit 3.0.

### Genome size estimation and genome assembly

Genome size of *P. generosa* was estimated using *k*-mer analysis. Counting of *k*-mers was conducted using Jellyfish (RRID:SCR_005491, version 2.2.10) [21]. The genome size, heterozygosity, and repeat content were estimated using GCE 1.0.2 [41]. For genome assembly, long reads generated from the PacBio Sequel platform were assembled using Falcon (RRID:SCR_016089) [42], which was subsequently polished using Arrow. Short paired-end clean reads from BGISEQ-500 were then used for correcting postprocessing errors and resolving conflicts of assembly via Pilon (RRID:SCR_014731, version 1.22) [38]. The assembled contigs were corrected for misjoins, orders, orients, and anchored contigs from the draft assembly into a candidate chromosome-length assembly by Hi-C data using Juicer (RRID:SCR_017226) [43] and 3D-DNA [44]. The scaffolds shorter than 20 Kb were removed. Finally, the candidate assembly was reviewed with Juicebox Assembly Tools (RRID:SCR_021172) for quality control and interactive corrections [45]. The Hi-C heatmap was visualized using Juicebox (RRID:SCR_021172) (with the bin length of 100 kb) as the interactive signals between each pair of 2 bins. The completeness of genome assembly was assessed by BUSCO (RRID:SCR_015008, version 5.4.3) [25] using the metazoa_odb10 database. The genome landscape illustrating the length, repeat element density, gene density, and GC content was created by circos-0.69–9 (RRID:SCR_011798) [46].

### Annotations of gene structure and function

Homologous and *de novo* predictions were both applied to annotate transposable elements in the *P. generosa* genome. In homologous prediction, RepeatMasker (RRID:SCR_012954) and RepeatProteinMask [47] were used to screen the *P. generosa* genome for known transposable elements in the RepBase library (RRID:SCR_021169) [48]. In *de novo* prediction, RepeatModeler (version 1.0.4) was first used for *de novo* candidate database construction of repetitive elements, and repetitive sequences were then annotated using RepeatMasker. Tandem repeats were *de novo* predicted using Tandem repeats finder (version 4.07) [49]. The results were then integrated and duplicates were eliminated.

Three complementary approaches were adopted to predict PCGs in the *P. generosa* genome, including homology-based prediction, *de novo* annotation, and transcriptome-based prediction. For homology-based prediction, gene sets from 8 closely related bivalves (*P. yessoensis, P. fucata, Mytilus galloprovincialis, Limnoperna fortune, A. purpuratus, S. constricta, S. broughtonii*, and *C. gigas*) were used. First, protein repertoires of those organisms were aligned against the *P. generosa* genome using TBLASTN (RRID:SCR_011822) [50]. Then gene structures were predicted from these blast hits by Exonerate v2.2.0 [51]. The *de novo* gene prediction was performed using a combination of Augustus (RRID:SCR_008417) [52] and SNAP (RRID:SCR_007936) [53] with default settings. Models used for each gene predictor of Augustus and SNAP training were obtained from a set of high-quality proteins generated from the RNA-seq and ISO-seq dataset by MAKER 2 (RRID:SCR_005309). For transcriptome-based prediction using RNA-seq data, RNA-seq reads were directly mapped to the genome using TopHat2 (RRID:SCR_013035) [54]. The mapped reads were subsequently assembled into gene models (Cufflinks-set) by Cufflinks (RRID:SCR_014597) [55]. For transcriptome-based prediction based on Iso-seq data, Iso-seq reads were directly mapped to the genome using GMAP (RRID:SCR_008992) [56]. The mapped reads were subsequently assembled by PASA (RRID:SCR_014656) [57]. Gene predictions from the homology-based approach, *de novo* approach, and RNA-seq–based and Iso-seq–based evidences were merged, and redundancy was removed to form a comprehensive consensus gene set using Maker 2 (RRID:SCR_005309) [58]. To validate the completeness of the gene structure annotation, we also used BUSCO (version 5.4.3) with the metazoa_odb10 database [25].

### Collinearity analysis

Homologous PCGs in *P. generosa* and *S. constricta* were identified using BLAST v2.11.0 (blastp, E value 1e^−5^), which was used for subsequent analysis using WGDI [59]. WGDI analysis results included dotplot and syntenic blocks with default parameters.

### Phylogenetic analysis and divergence time estimation

Gene families were constructed using the OrthoMCL (RRID:SCR_007839) pipeline [53]. We selected *P. generosa* and other 11 species (*C. gigas, P. yessoensis, P. maximus, A. purpuratus, S. broughtonii, P. martensi, B. platifrons, H. sapiens, X. tropicaalis, D. rerio*, and *C. elegans*) for gene family analysis. For the gene set of each genome, only the transcript with the longest coding sequence was selected from alternate splice transcripts. Genes with fewer than 50 amino acids were removed from further analysis. Protein sequences were aligned by “all-vs-all BLASTP” (E value = 1e^−5^) [44]. Then, the Markov clustering (MCL) algorithm implemented in OrthoMCL was used to group orthologs and paralogs from all input species with an inflation value of 1.5 [60].

The phylogenetic tree was constructed following procedures described in previous studies [14, 35, 61]. Briefly, for phylogenetic tree construction and divergence time estimation, shared single-copy genes of *P. generosa* and 11 other species were used. The protein sequences of single-copy orthologs among the 12 species were aligned using MUSCLE v3.7 (RRID:SCR_011812) [62] with default parameters. Phylogenetic relationships were inferred based on the super-matrix estimated from the concatenated alignment of single-copy genes using the maximum likelihood [63] method implemented in RAxML v2.2 (RRID:SCR_006086) [64] with the optimal amino acid substitution model selected by the PROTGAMMALGX parameter.

Based on gene family identification and phylogenetic analysis, single-copy genes and mcmctree in PAML [65] were used to estimate divergence time [66–69]. The time correction points were *C. elegans* and *H. sapiens* (678.3–855.2 Mya), *D. rerio* and *H. sapiens* (413.1–443.0 Mya), and *X. tropicalis* and *H. sapiens* (347.0–357.9 Mya). The time correction points were taken from the Timetree website. The operating parameters of mcmctree were as follows: burn-in = 10,000, sample number = 1,000,000, and sample frequency = 50.

### Gene family analysis and 3-dimensional protein structure modeling

The clustering results of gene families and the phylogenetic tree with divergence time estimated were used to analyze the expansion and contraction of orthologous gene families between ancestor and each of the 12 species (*P. generosa* and the other 11 species) using a stochastic birth and death model with the lambda parameter by CAFE (RRID:SCR_005983, version 4.0) [70]. This model was further used to calculate the number of gene families along each lineage on the phylogenetic tree. A probabilistic graphical model was introduced to calculate the probability of transitions in gene family size from parent to child nodes. The family-wide *P* values were calculated in each lineage based on the conditional likelihood.

In addition, we also compared the gene families between *P. generosa* and the other 8 bivalves. Pfam domains of *P. generosa* and the other 8 bivalves were obtained using InterProscan [32] (5.54–87.0) and visualized using TBtools (v1.120) [71]. The positions of copine genes in *P. generosa* chromosomes were illustrated using R packages gggenes, and the structure of copine genes was illustrated using Gene Structure Display Server 2.0 online [72]. Phylogenetic trees of copine genes of *P. generosa* and *S. constricta* were constructed using the maximum likelihood [63] method implemented in MEGA (v7.0.26) with 1,000 bootstrap replicates.

Three-dimensional protein structure models were predicted using AlphaFold2 and visualized using ChimeraX [34].

## Supplementary Material

giad105_GIGA-D-22-00284_Original_Submission

giad105_GIGA-D-22-00284_Revision_1

giad105_GIGA-D-22-00284_Revision_2

giad105_GIGA-D-22-00284_Revision_3

giad105_Response_to_Reviewer_Comments_Original_Submission

giad105_Response_to_Reviewer_Comments_Revision_1

giad105_Response_to_Reviewer_Comments_Revision_2

giad105_Reviewer_1_Report_Original_Submission Timothy Gordon Stephens, Ph.D. -- 11/24/2022 Reviewed

giad105_Reviewer_1_Report_Revision_1 Timothy Gordon Stephens, Ph.D. -- 6/27/2023 Reviewed

giad105_Reviewer_1_Report_Revision_2 Timothy Gordon Stephens, Ph.D. -- 9/27/2023 Reviewed

giad105_Reviewer_2_Report_Original_Submission Ying Lu -- 3/20/2023 Reviewed

giad105_Reviewer_2_Report_Revision_1 Ying Lu -- 7/30/2023 Reviewed

giad105_Supplemental_Tables_and_Figures

## Data Availability

The whole-genome project of *P. generosa* has been deposited at NCBI/BioProject PRJNA859289. The raw next-generation sequencing reads of DNA are available at SRA (SRR22190027-SRR22190030), raw long-read PacBio sequencing reads of DNA are available at SRA (SRR22190026), raw next-generation sequencing reads of RNA are available at SRA (SRR22190032), raw Hi-C reads are available at SRA (SRR22190025), and raw long-read PacBio sequencing reads of RNA are available at SRA (SRR22190031). The genome assembly data have been deposited under accession No. JAPMAH000000000.1. All additional supporting data are available in the *GigaScience* repository, GigaDB [73].

## Additional Files

**Supplementary Fig. S1**. The 17-mer count distribution for the genome size estimation.

**Supplementary Figure S2**. The enriched KEGG pathways of significantly expanded gene families (*P* ≤ 0.01, top 20) in *P. generosa*.

**Supplementary Table S1**. The genome assembly information of bivalves in the public database.

**Supplementary Table S2**. Statistics of 17-mer analysis.

**Supplementary Table S3**. The chromosome information of *P. generosa*.

**Supplementary Table S4**. The heterozygosity of bivalves reported.

**Supplementary Table S5**. Functional annotation of the predicted protein-coding genes in the *P. generosa* genome assembly.

**Supplementary Table S6**. The assembly quality of chromosomal genomes of bivalves by BUSCO.

**Supplementary Table S7**. The information of 30,616 gene families of *P. generosa* and 11 other species.

**Supplementary Table S8**. The enriched KEGG pathways of expanded gene families in the *P. generosa* genome assembly.

## Abbreviations

BLAST: Basic Local Alignment Search Tool; bp: base pairs; BUSCO: Benchmarking Universal Single-Copy Orthologs; Gb: gigabase pairs; GC: guanine–cytosine; GnRH: gonadotropin-releasing hormone; Hi-C: high-throughput/resolution chromosome conformation capture; Iso-seq: isoform sequencing; kb: kilobase pairs; KEGG: Kyoto Encyclopedia of Genes and Genomes; Mb: megabase pairs; Mya: million years ago; NCBI: National Center for Biotechnology Information; PacBio: Pacific Biosciences; PCG: protein-coding gene; RNA-seq: RNA sequencing.
