# Supplementary material for: Chromosome-level genome assembly of the Pacific geoduck Panopea generosa reveals major inter- and intrachromosomal rearrangements and substantial expansion of the copine gene family
Source: Gigascience. 2023 Dec 19;12:giad105. doi: 10.1093/gigascience/giad105 (PMC10729735; doi:10.1093/gigascience/giad105)
Supplement: giad105_GIGA-D-22-00284_Original_Submission [file giad105_giga-d-22-00284_original_submission.pdf]

GigaScience

Chromosome-level genome assembly of the Pacific geoduck Panopea generosa

--Manuscript Draft--

Manuscript Number:	GIGA-D-22-00284
Full Title:	Chromosome-level genome assembly of the Pacific geoduck Panopea generosa
Article Type:	Data Note
Funding Information:	Strategic Priority Research Program of Chinese Academy of Sciences (XDB42000000) — Professor Nansheng Chen<br>Chinese Academy of Sciences Pioneer Hundred Talents Program — Professor Nansheng Chen<br>Taishan Scholar Foundation of Shandong Province — Professor Nansheng Chen<br>Qingdao Innovation and Creation Plan — Professor Nansheng Chen<br>Earmarked Workstation Fund for QRJH — Professor Chunde Wang, Professor Nansheng Chen
Abstract:	Background: The Pacific geoduck Panopea generosa (class Bivalve, order Adapedonta, family Hiatellidae, genus Panopea) is the largest known burrowing bivalve with considerable commercial value. Pacific geoduck and other geoduck clams play important roles in maintaining ecosystem health for their filter feeding habit and coupling pelagic and benthic processes. Chromosome-level genomes of geoduck clams will contribute to genetic breeding, as well as ecosystem and climate change biology.<br>Findings: Here, we report the first high-quality chromosome-scale genome assembly of P. generosa with the purpose to unravel its phylogenetic characteristics and molecular mechanisms of its life strategies, and promote research on genetic breeding. The assembled P. generosa genome was 1.45 Gb in size, with a contig N50 of 1.6 Mb and a scaffold N50 of 73.8 Mb. The assembly contained 39 scaffolds anchored to 19 chromosomes with an anchoring rate of 94.7%. BUSCO analysis showed 90.9% completeness in the 889 eukaryote core conserved genes in the assembled P. generosa genome. Of the 35,034 predicted protein-coding genes, 30,700 genes (87.63%) were functionally annotated. Comparative analysis of the genomes of P. generosa and 11 related species identified 507 expanded gene families and 875 contracted gene families in P. generosa. Enrichment analysis revealed significant expansion of immune and gonad development gene families that may promote its complex survival strategies.<br>Conclusions: The availability of the P. generosa genome and its annotated gene set may provide a useful molecular platform for researches on its genetic breeding.
Corresponding Author:	Nansheng Chen<br>Institute of Oceanology Chinese Academy of Sciences<br>Qingdao, Shandong CHINA
Corresponding Author Secondary Information:	
Corresponding Author's Institution:	Institute of Oceanology Chinese Academy of Sciences
Corresponding Author's Secondary Institution:	
First Author:	Jing Wang
First Author Secondary Information:	
Order of Authors:	Jing Wang<br>Qing Xu<br>Min Chen<br>Yang Chen

Powered by Editorial Manager® and ProduXion Manager® from Aries Systems Corporation

	Chunde Wang
	Nansheng Chen
**Order of Authors Secondary Information:**	
**Additional Information:**	
**Question**	**Response**
Are you submitting this manuscript to a special series or article collection?	No
**Experimental design and statistics**<br><br>Full details of the experimental design and statistical methods used should be given in the Methods section, as detailed in our Minimum Standards Reporting Checklist. Information essential to interpreting the data presented should be made available in the figure legends.<br><br>Have you included all the information requested in your manuscript?	Yes
**Resources**<br><br>A description of all resources used, including antibodies, cell lines, animals and software tools, with enough information to allow them to be uniquely identified, should be included in the Methods section. Authors are strongly encouraged to cite Research Resource Identifiers (RRIDs) for antibodies, model organisms and tools, where possible.<br><br>Have you included the information requested as detailed in our Minimum Standards Reporting Checklist?	Yes
**Availability of data and materials**<br><br>All datasets and code on which the conclusions of the paper rely must be either included in your submission or deposited in publicly available repositories (where available and ethically appropriate), referencing such data using	Yes

a unique identifier in the references and in the "Availability of Data and Materials" section of your manuscript.<br><br>Have you have met the above requirement as detailed in our Minimum Standards Reporting Checklist?	

Powered by Editorial Manager® and ProduXion Manager® from Aries Systems Corporation

Manuscript

Click here to access/download;Manuscript;GIGA_main_document.docx

Chromosome-level genome assembly of the Pacific geoduck *Panopea generosa*

Jing Wang[1,2,3], Qing Xu[1,2,3], Min Chen[4], Yang Chen[1,2,3], Chunde Wang[4,5*], Nansheng Chen[1,2,3,6*]

[1]CAS Key Laboratory of Marine Ecology and Environmental Sciences, Institute of Oceanology, Chinese Academy of Sciences, Qingdao, China

[2]Laboratory of Marine Ecology and Environmental Science, Qingdao National Laboratory for Marine Science and Technology, Qingdao, China

[3]Center for Ocean Mega-Science, Chinese Academy of Sciences, Qingdao, China

[4]Yantai Institute of Coastal Zone Research and Center for Ocean Mega-Science, Chinese Academy of Sciences, Yantai, China

[5]Marine Science and Engineering College, Qingdao Agricultural University, Qingdao, China

[6]Department of Molecular Biology and Biochemistry, Simon Fraser University, Burnaby, BC, Canada

Jing Wang Email: wangjing2019@qdio.ac.cn; Qing Xu Email: xuqing_77@163.com; Min Chen Email: mchen@yic.ac.cn; Yang Chen Email: cy4043@hevttc.edu.cn.

∗ Correspondence address. Chunde Wang E-mail: chundewang2007@163.com; Nansheng Chen Email: chenn@qdio.ac.cn

Abstract

Background: The Pacific geoduck *Panopea generosa* (class Bivalve, order Adapedonta, family Hiatellidae, genus *Panopea*) is the largest known burrowing bivalve with considerable commercial value. Pacific geoduck and other geoduck clams play important roles in maintaining ecosystem health for their filter feeding habit and coupling pelagic and benthic processes. Chromosome-level genomes of geoduck clams will contribute to genetic breeding, as well as ecosystem and climate change biology.

Findings: Here, we report the first high-quality chromosome-scale genome assembly of *P. generosa* with the purpose to unravel its phylogenetic characteristics and molecular mechanisms of its life strategies, and promote research on genetic breeding. The assembled *P. generosa* genome was 1.45 Gb in size, with a contig N50 of 1.6 Mb and a scaffold N50 of 73.8 Mb. The assembly contained 39 scaffolds anchored to 19 chromosomes with an anchoring rate of 94.7%. BUSCO analysis showed 90.9% completeness in the 889 eukaryote core conserved genes in the assembled *P. generosa* genome. Of the 35,034 predicted protein-coding genes, 30,700 genes (87.63%) were functionally annotated. Comparative analysis of the genomes of *P. generosa* and 11 related species identified 507 expanded gene families and 875 contracted gene families in *P. generosa*. Enrichment analysis revealed significant expansion of immune and gonad development gene families that may promote its complex survival strategies.

Conclusions: The availability of the *P. generosa* genome and its annotated gene set may provide a useful molecular platform for researches on its genetic breeding.

Keywords: *Panopea generosa*, chromosome-level genome assembly, genetic breeding,

evolutionary adaptation

Context

The Pacific geoduck *Panopea generosa* is one member of genus *Panopea* which includes the world's largest burrowing bivalves. *Panopea generosa* is usually found in low intertidal and subtidal sediments throughout the northeast Pacific coast, including the United States (Alaska, Washington, and California), Canada (British Columbia), and Mexico (north Baja Pacific Coast) [1, 2]. Geoducks can reach more than 25 cm in shell length, and more than 100 cm in siphon length [3]. Geoduck adults are usually buried in muddy-sandy sediment at depths ranging 60–100 cm, with only their siphon tips exposed to respire, capture food, and release secretion/excretion products and gametes. The sedentary behavior may contribute to their long life spans (which can be as long as 168 years) for *P. generosa* [4]. Due to these unique life strategies, it is expected that geoduck should have distinctive growth and development mechanisms, especially in relation to benthic life and immune system.

Geoduck clams play important roles in maintaining ecosystem health for their filter feeding habit and coupling pelagic and benthic processes by ejecting undigested mucus-bound feces and pseudo feces to the sediment surface. They are preys for sea otters, fishes, crabs, and sea stars [5, 6]. As marine calcifiers, shell concentrations of *Panopea* inside Scalichnus burrows have been analyzed to reconstruct the sequence of events related to storm events [7]. Geoduck clams possess great commercial fishery value in Canada and the USA [8]. Since the recruitment of geoducks have been low [9] due to overfishing and their vulnerability to environmental changes [10], there has been an increasing interests in genetic breeding of geoducks.

Nevertheless, high-quality chromosome-scale reference genome of *P. generosa* is currently not available, hindering the development of geoduck genetic breeding programs. In this study, we report the first chromosome-scale genome assembly for *P. generosa* by combining cutting-edge technologies including next-generation sequencing (BGIseq), PacBio Sequel sequencing, and high-throughput chromosome conformation capture (Hi-C) technologies. We further performed gene family clustering, phylogenetic analysis, and gene family expansion and contraction, in order to understand its adaptation, growth, development and immunity. The genome information will facilitate further researches in molecular evolution and genetic breeding.

Methods

Sampling collection

Geoducks *P. generosa* were collected from the Strait of Georgia (49°41′12″N, 124°51′33″W) of British Columbia, Canada in the spring of 2019. The animals were then transferred to the laboratory and kept in a tank with running water for a week. One animal was chosen and dissected on ice to collect tissue samples, including labial palp, heart, foot, gonad, gill, hepatopancreas, siphon, and mantle muscle. This animal was identified to be a female as indicated by the presence of eggs in the smear of the gonad under a compound microscope. Dissected tissues were quickly frozen in liquid nitrogen and then stored at −80°C before DNA and RNA extraction.

DNA library construction and sequencing

Genomic DNA of *P. generosa* was extracted using a standard phenol-chloroform

extraction method. The quality of DNA was determined by gel electrophoresis to ensure the DNA samples met library sequencing requirements. Sequence libraries with insert size of 300 bp were constructed for the sequence platform BGIseq-500 (BGISEQ-500, RRID:SCR_017979), which were used in the genome size estimation, k-mer analysis [11] and for correcting errors in the Pilon (Pilon, RRID:SCR_014731) assembly [12]. A Hi-C library with insert size of 300 bp was constructed to provide long-range information (without position information) on the grouping and linear organization of sequences along entire chromosomes to assemble the scaffolds into chromosome-level scaffolds [13]. And a PacBio library with insert size of 20 Kb was constructed to obtain long reads by the PacBio Sequel platform using the Sequel Sequencing Kit 3.0. The raw sequence data generated by the BGIseq platform were filtered by the following criteria: reads with adapters, reads with N bases more than 1% and reads with low-quality bases ($Q \leq 5$) more than 10%. Subreads of PacBio data were filtered by the default parameters.

RNA library construction and sequencing

RNA-seq and Iso-seq were conducted to obtain transcriptome data to aid genome annotation. The total RNAs was extracted by Trizol (Invitrogen, Carlsbad, CA, USA) from eight tissues of the same *P. generosa* individual, including labial palp, heart, foot, gonad, gill, hepatopancreas, siphon, and mantle muscle. Each RNA sample was qualified and quantified using a NanoDrop and an Agilent 2100 bioanalyzer (Thermo Fisher Scientific, MA, USA). For RNA-seq, the mRNA library was constructed by purification, fragmentation, a first-strand cDNA generation, a second-strand cDNA synthesis, and RNA index addition. The eight libraries were sequenced using the BGIseq-500 platform. For Iso-Seq, the total

RNA was extracted from the equally mixed tissues of the 8 tissues above. The PacBio SMRTbell library was prepared and sequenced by the PacBio Sequel sequencer (PacBio Sequel System, RRID:SCR_017989) with Sequel Sequencing Kit 3.0.

Genome size estimation and genome assembly

Genome size of *P. generosa* was estimated using k-mer analysis. Counting of k-mers was conducted using Jellyfish (Jellyfish, RRID:SCR_005491, version 2.2.10) [11]. For genome assembly, long reads generated from PacBio Sequel platform were assembled using Falcon (FALCON, RRID:SCR_016089) [14], which was subsequently polished using Arrow. Short paired-end clean reads from BGIseq-500 were then polished using Pilon (Pilon, RRID:SCR_014731, version 1.22) [12]. Chromosomes were then assembled using Hi-C data filtered by SOAPnuke (SOAPnuke, RRID:SCR_015025) and mapped by HiC-Pro (HiC-Pro, RRID:SCR_017643) [15]. Chromosomes were further corrected using Juicebox (Juicebox, RRID:SCR_021172) [16]. The completeness of genome assembly was assessed using BUSCO (BUSCO, RRID:SCR_015008, version 3.0.2) [17].

Annotations of gene structure and function

Homologous and *de novo* predictions were both applied to annotate repetitive sequences in the *P. generosa* genome. In homologous prediction, RepeatMasker (RepeatMasker, RRID:SCR_012954) and the associated RepeatProteinMask [18] were performed by alignment against Repbase database (Repbase, RRID:SCR_021169) [19]. In *de novo* prediction, Piler [20], RepeatScout [21] and RepeatModeler (version 1.0.4) [22] were used for de novo candidate database construction of repetitive elements, and repetitive sequences were then annotated using RepeatMasker. Besides, tandem repeats

were de novo predicted using Tandem repeats finder (version 4.07) [23]. The results were then integrated and redundancy was eliminated.

Three complementary approaches were adopted to predict protein-coding genes in *P. generosa* genome, including homology-based prediction, *De novo* annotation, and transcriptome-based prediction. For homology-based prediction, eight closely related species (*Patinopecten yessoensis, Pinctada fucata, Mytilus galloprovincialis, Limnoperna fortune, Argopecten purpuratus, Sinonovacula constricta, Scapharca broughtonii, and Crassostrea gigas*) from different branches were chosen to confirm the completeness of the gene set. First, protein repertoires of those organisms were aligned against the *P. generosa* genome using TBLASTN (TBLASTN, RRID:SCR_011822) [24]. The Basic Local Alignment Search Tool (blast) hits were then conjoined by Solar software (SOLAR, RRID:SCR_000850) [25]. Next, GeneWise (GeneWise, RRID:SCR_015054) [26] was used to predict the exact gene structure of the corresponding genomic region on each blast hit. Notably, homology predictions were denoted as "Homology-set". For *de novo* annotation, six programs were simultaneously used, including Augustus (Augustus, RRID:SCR_008417) [27], GeneID (GeneID, RRID:SCR_002473) [28], GeneScan (GENSCAN, RRID:SCR_012902) [29], GlimmerHMM (GlimmerHMM, RRID:SCR_002654) [30], FgeneSH [31] and snap (SNAP, RRID:SCR_007936) [32]. Among them, Augustus, snap and GlimmerHMM were trained using PASA-T-set gene models. For transcriptome-based prediction using RNA-Seq data, RNA-Seq reads were directly mapped to the genome using TopHat2 (TopHat, RRID:SCR_013035) [33]. The mapped reads were subsequently assembled into gene models (Cufflinks-set) by Cufflinks (Cufflinks,

RRID:SCR_014597) [34]. For transcriptome-based prediction based on Iso-Seq data, Iso-Seq reads were directly mapped to the genome using Gmap (GMAP, RRID:SCR_008992) [35]. The mapped reads were subsequently assembled by Pasa (PASA, RRID:SCR_014656) [36]. Gene predictions from the homology-based approach, *de novo* approach, RNA-Seq-based and Iso-Seq-based evidences were merged to form a comprehensive consensus gene set using EvidenceModeler (EVidenceModeler, RRID: SCR_014659) [37]. To validate the completeness of the gene structure annotation, we also used BUSCO (version 3.0.2) [17].

Phylogenetic analysis and divergence time estimation

Gene families were constructed according to OrthoMCL (OrthoMCL DB: Ortholog Groups of Protein Sequences, RRID:SCR_007839) pipeline. The protein-coding genes of *P. generosa* and other 11 species (*C. gigas*, *P. yessoensis*, *P. maximus*, *A. purpuratus*, *S. broughtonii*, *Pinctada martensi*, *Bathymodiolus platifrons*, *Homo sapiens*, *Xenopus tropicaalis*, *Danio rerio*, and *Caenorhabditis elegans*) were filtered. When multiple transcripts (suggesting alternative splicing) were found for a gene, only the transcript with the longest coding sequence was retained. Meanwhile, the encoding proteins with less than 50 amino acids were removed from further analysis. Protein sequences were aligned by “all-vs-all blastp” (E value = $1e^{-5}$ by default) [24], and clustered using orthomcl (OrthoMCL DB: Ortholog Groups of Protein Sequences, RRID: SCR_007839) 2.0 with the expansion coefficient 1.5 [38]. Finally, to perform multiple sequence alignment for single copy genes clustered, and concatenate sequence alignment of single-copy gene families. Based on the clustering results, the division of gene family was obtained.

To construct phylogenetic trees, protein sequences of each single copy gene of *P. generosa* and other 11 species were aligned using MUSCLE (MUSCLE, RRID:SCR_011812) [39], and then the protein alignment results were converted into CDS alignment results. Next, conserved sites were extracted using Gblocks (Gblocks, RRID:SCR_015945) [40], followed by combining all single copy genes according to the species order to form a super alignment matrix. The phylogenetic tree was constructed using maximum likelihood (ML) algorithm in RAxML (RAxML, RRID:SCR_006086) [41] with the optimal amino acid substitution model selected by the PROTGAMMAAUTO parameter.

Based on gene family identification and phylogenetic analysis, single copy genes and mcmctree in PAML [42] were used to estimate divergence time [43-46]. The time correction points were *C. elegans* and *H. sapiens* (678.3–855.2 MYA), *D. rerio* and *H. sapiens* (413.1–443.0 MYA), *X. tropicalis* and *H. sapiens* (347.0–357.9 MYA). The time correction points were taken from the Timetree website. The operating parameters of mcmctree: burn in = 10000, sample number = 1000000, sample frequency = 50.

Gene family expansion and contraction

The clustering results of gene families and the phylogenetic tree with divergence time estimated were used to analyze the expansion and contraction of orthologous gene families between ancestor and each of the 12 species (*P. generosa* and the other 11 species) using a stochastic birth and death model with lambda parameter by CAFE (CAFE, RRID:SCR_005983, version 4.0) [47]. This model was further used to calculate the number of gene families along each lineage on the phylogenetic tree. A probabilistic graphical

model was introduced to calculate the probability of transitions in gene family size from parent to child nodes. The family-wide P-Values and Viterbi P-Values were calculated in each lineage based on the conditional likelihood.

Results

Genome sequencing and assembly

Genome survey analysis using BGIseq reads estimated the genome size of *P. generosa* as 1.48 Gb (Table 1). The heterozygosity and repeated sequence content were estimated to be 1.37% and 57.99% (Supplementary Figure S1 and Table S1), respectively. Genome assembly using PacBio reads and Falcon assembler obtained an initial 1.51 Gb genome. Further assembly using Hi-C data obtained a genome with 19 chromosomes (Figure 1A). This assembly, which has a total length of 1,474,161,289 bp with a contig N50 of 1.57 Mb and a scaffold N50 of 73.79 Mb (Figure 1B; Table 2), included 39 scaffolds anchored to 19 chromosomes with an anchoring rate of 94.70%. As expected, the genomic regions with low gene density typically had high repeat content, while the regions with high repeat content usually had high GC content.

Table 1. Statistics of the DNA sequence data used for *P. generosa* genome assembly

Source	Platform	Library size	Clean data (Gb)	Read length (bp)	Sequencing coverage (x)
**Genome-**	BGISEQ-	300 bp	258.19	150	181

**short reads**	500				
**Genome-long reads**	PacBio sequel	20 Kb	164.46	26,513*	115
**Hi-C**	BGISEQ-500	300 bp	233.49	150	163

[a]"26,513*" indicated the N50 of subreads.

Table 2. Statistics of the genome assembly of *P. generosa*

Statistics	Scaffold	Contig
**Total Number (#)**	39	2,086
**Total length (bp)**	1,474,161,289	1,473,137,789
**Average Length (bp)**	37,799,007	706,202
**N50 Length (bp)**	73,788,920	1,571,249
**N90 Length (bp)**	53,843,121	418,579
**Maximum Length (bp)**	101,196,518	6,469,558
**Minimum Length (bp)**	29,000	17
**GC content**	34.33%	34.33%
**Anchored rate (%)**	94.70%	

The assembled genome size of *P. generosa* fell in the range of the genomes of bivalve species reported, which varied from 543.9 Mb in *Lutraria thynchaena* [48] to 2.6 Gb in

Modiolus philippinarum [49] (Supplementary Table S2). Among bivalve species, the genome sizes of most superorder Imparidentia species ranged from 1 Gb to 1.8 Gb, and that of the species in order Adapedonta ranged from 1 Gb to 1.5 Gb.

The numbers of chromosomes varied substantially among bivalve species. While the species of the order Ostreida, including *Crassostrea gigas* [50], *Crassostrea virginica*, *Crassostrea hongkongensis* [51], *Crassostrea ariakensis* [52], have 10 chromosomes, and the species of the order Ostreida, including *Pinctada fucata* [53] and the species of order Mytilida including *Mytilus coruscus* [54] and *Mytilus edulis* have14 chromosomes. The Myida order species *Dreissena polymorpha* and all *Argopecten* scallop have 16 chromosomes [55]. In contrast, the numbers of chromosomes of species in most other orders including Adapedonta, Venerida, Cardiida, Arcida, and Pectinida, are 19, except for *Corbicula fluminea* [56], which has 18 chromosomes.

Genome annotation and evaluation

The majority (57.99%) of the *P. generosa* genome was repetitive elements estimated using *de novo* searching and homolog prediction (Supplementary Table S3). Distribution of these repetitive elements was uneven with repetitive content per 1 Mb varied from 34.76% to 84.89% (Figure 1B). DNA transposons (21.6%), long interspersed nuclear elements (LINEs, 9.1%) and long terminal repeats (LTRs, 3.76%) were the top three categories of repetitive elements in the *P. generosa* genome (Table 3).

Table 3. Repetitive element annotations in *P. generosa*

Type	Repbase TEs		TE protiens		De novo		Combined TEs	
	Length (bp)	% in genome	Length (bp)	% in genome	Length (bp)	% in genome	Length (bp)	% in genome
**DNA**	47682857	3.23	1595949	0.11	290956252	19.74	318426608	21.6
**LINE**	30000303	2.04	27733009	1.88	106147965	7.2	134171392	9.1
**SINE**	1165771	0.08	0	0	26319255	1.79	27249093	1.85
**LTR**	15590650	1.06	6933113	0.47	43008577	2.92	55380608	3.76
**Other**	31141	0	0	0	0	0	31141	0
**Unknown**	0	0	0	0	151891763	10.3	151891763	10.3
**Total**	79231692	5.37	36258899	2.46	554218329	37.6	583000390	39.55

A total number of 35,034 protein-coding genes were annotated in the *P. generosa* genome. The mean number of exons per gene was 5.78 (Supplementary Table S4). Of these protein-coding genes, 30,700 genes were annotated to contain conserved functional motifs (Supplementary Table S5).

To evaluate the completeness of the assembly, the assembled *P. generosa* genome was assessed using BUSCO (Simao et al. 2015) with the metazoan odb9 database (978 core genes). We found that 90.9% of core genes were identified as full-length in *P. generosa* genome assembly and 43 (4.4%) core genes were captured as fragments (Table 4), suggesting that the genome assembly was of high quality.

Table 4. BUSCO results for analysis of genome completeness for *P. generosa*

Type	Number of genes	Percentage (%)
**Complete BUSCOs (C)**	889	90.9
**Complete and single-copy BUSCOs (S)**	837	85.6
**Complete and duplicated BUSCOs (D)**	52	5.3
**Fragmented BUSCOs (F)**	43	4.4
**Missing BUSCOs (M)**	46	4.7
**Total BUSCO groups searched**	978	100

Comparative analysis of gene families and evolutionary analysis

Using the protein-coding genes of *P. generosa* and other 11 species (*P. martensi*, *C. gigas*, *B. platifrons*, *P. yessoensis*, *P. maximus*, *A. purpuratus*, *S. groughtonii*, *H. sapiens*, *X. tropicaalis*, *D. rerio*, and *C. elegans*) (Table 5), 30,616 gene families were identified as single-copy and multiple-copy genes, unique paralogs, other orthologs, and unclustered genes, as well as 326 single-copy orthologous gene families (Figure 2). The analysis identified 7917 genes belonging to 1749 gene families that are specific to *P. generosa*. Comparative analysis of the genes of *P. martensi*, *S. broughtonii*, *P. yessoensis*, and *P. generosa* revealed 6940 gene families that are common to these animals and 2902 gene families that are specific to *P. generosa* (Figure 3). Phylogenetic analysis using 326 single-copy orthologous gene families from these 12 species showed that *P. generosa* was tightly clustered with other bivalve species as expected (Figure 4). According to the phylogenetic tree, the divergence time of *P. generosa* from its nearest node was approximately 491.5 Mya (Figure 4). In addition, the divergence time of *P. generosa* is earlier than other bivalve

species, which is consistent with that of *S. constricta*, a species close to *P. generosa* [57]. *B. platifrons*, *C. gigas* and *P. martensi*, which have 10 or 14 chromosomes, were clustered as a single clade and diverged from other bivalves with 19 chromosomes in the phylogenetic tree.

Table 5. The protein-coding genes of *P. generosa* and other 11 species for evolutionary analysis

**Species**	**Total genes**	**Unclustered genes**	**Families**	**Unique families**	**Ave. genes per family**
P. generosa	35,034	6,168	12,034	1,749	2.4
A. purpuratus	26,256	3,720	13,196	290	1.71
B. platifrons	33,584	3,197	12,409	1,775	2.45
C. gigas	28,402	3,638	11,818	828	2.1
D. rerio	25,444	1,791	9,210	295	2.57
H. sapiens	20,229	1,488	9,251	226	2.03
P. yessoensis	24,521	1,704	13,017	137	1.75
P. maximus	26,152	1,518	13,276	164	1.86
P. martensi	25,526	2,403	11,043	318	2.09
S. broughtonii	24,045	2,770	11,314	538	1.88
X. tropicalis	19,967	1,016	9,226	159	2.05
C. elegans	33,552	5,600	8,201	3,720	3.41

Gene family expansion and contraction in *P. generosa*

A total of 507 expanded gene families (involving 2,734 genes) and 875 contracted gene families (involving 792 genes) were identified in *P. generosa* genome compared to the most recent common ancestor of both *P. generosa* and the other 11 species (Figure 5). KEGG analysis revealed 166 pathways from the expanded gene families and 123 pathways from the contracted gene families were significantly enriched (Qvalue ≤ 0.05) with various biological processes (Supplementary Table S6), suggesting their important contribution to the adaptation of benthic bivalves. The enrichment analysis suggested that the significantly expanded genes were mainly represented in 5 biochemical metabolic pathways of KEGG category, including organismal systems, human diseases, metabolism, environmental information processing, and cellular processes. KEGG subcategories included signal transduction and endocrine system, which were ranked as the most enriched pathways, followed by infectious diseases (Bacterial), amino acid metabolism, lipid metabolism, digestive and immune system.

Significantly expanded and contracted genes were found to be related to ovarian development, immune, osmoregulation, and pigmentation (Figure 5, and Supplementary Table S6), which may be important for the adaptation of benthic *P. generosa*. According to enrichment analyses, there were a few significant enrichment pathways (Qvalue < 0.05) related to gonad development. For example, adrenergic signaling in cardiomyocytes, glycine, serine and threonine metabolism has been found as enriched pathways related to spermatogenesis of the fluted giant clam *Tridacna squamosa* [58]. Moreover, oocyte meiosis, apoptosis, Ras signaling pathway, calcium signaling pathway, steroid hormone

biosynthesis, GnRH signaling pathway, insulin signaling pathway, oxytocin signaling pathway, and ovarian steroidogenesis have been documented to be enriched in *Procambarus clarkii* ovary development [59]. Geoducks have become a focus of significant aquaculture research and development with a considerable commercial value [60, 61]. The enriched gonad development-related pathways and genes could provide basic data for the further genetic breeding research of *P. generosa* and its closely related species.

Meanwhile, many enrichment pathways (Qvalue < 0.05) related to immune have been significantly enriched and appeared to be complex. Toll-like receptor signaling pathway, natural killer cell mediated cytotoxicity, antigen processing and presentation, B cell receptor signaling pathway, Fc epsilon RI signaling pathway, Fc gamma R-mediated phagocytosis, leukocyte transendothelial migration, and chemokine signaling pathway have been found in the immune pathways of the clam *Saxidomus prupuratus* transcriptome [62]. Toll-like receptor signaling pathway and apoptosis have also been reported in shell mussel *Mytilus coruscus* in response to *Vibrio alginnolyficus* infection. Immune-related pathways antigen processing and presentation, lysosome, phagosome, and PI3K-Akt signaling pathway have been proved to be the pathways of differentially expressed genes of *Chamys farreri* in response to tetrabromobisphenol A stress [63]. The other immune pathways of peroxisome, Rap1 signaling pathway, Ras signaling pathway, cGMP-PKG signaling pathway, cAMP signaling pathway, cell adhesion molecules (CAMs), Bacterial invasion of epithelial cells, and inflammatory mediator regulation of TRP channels have also been found in the enriched pathways of *Procambarus clarkii* [59]. Two significantly enriched pathwaFcys, phagosome and apoptosis have been found to be shared between *S.*

constricta and *P. generosa* [57]. In contrast, other pathways such as the NOD-like receptor signaling pathway, focal adhesion, NF-kappa B signaling pathway, tumor necrosis factor (TNF) signaling pathway, and endocytosis enriched in *S. constricta* were not significantly enriched in *P. generosa*. These differences may reflect their different life strategies in adaptation to different survival depth below the substratum surface and pathogenic stresses.

Conclusions

In summary, a high-quality chromosome-level genome assembly of *P. generosa* was obtained in this study. The assembled genome size was 1.45 Gb, with a contig N50 of 1.6 Mb and 19 chromosomes. A total of 35,034 protein-coding genes were predicted in the *P. generosa* genome, of which 30,700 genes (87.63%) were functionally annotated. *P. generosa* is a highly complex species with a high heterozygosity of 1.37% and 57.99% repeat sequences in genome. This study may provide a high-quality genomic resource for future studies of the phylogenetic characteristics, evolution and adaptation, immunology and may other related studies. Most importantly, the assembly of *P. generosa* genome may greatly facilitate genetic breeding of geoducks.

Data Availability

The Whole Genome project of *P. generosa* has been deposited at National Genomics Data Center/bioproject PRJCA011372. The raw next-generation sequencing reads of DNA

are available at GSA (Experiment accession CRX506408, Run accession CRR570510–CRR570525); raw long-read Pacbio sequencing reads of DNA are available at GSA (Experiment accession CRX506406, Run accession CRR570502–CRR570508); raw next-generation sequencing reads of RNA are available at GSA (Experiment accession CRX506409, Run accession CRR570526); raw Hi-C reads are available at GSA (Experiment accession CRX506407, Run accession CRR570509); and raw long-read Pacbio sequencing reads of RNA are available at GSA (Experiment accession CRX506410, Run accession CRR570527). The genome assembly data have been deposited under accession No. GWHBMBK00000000.

Additional Files

Supplementary Figure S1. The 17-mer count distribution for the genome size estimation.

Supplementary Table S1. Statistics of 17-mer analysis.

Supplementary Table S2. The genome assembly information of bivalve species in the public database.

Supplementary Table S3. Statistics of repetitive element annotations in *P. generosa* using various methods.

Supplementary Table S4. The structural statistics of gene prediction in *P. generosa*.

Supplementary Table S5. Functional annotation of the predicted protein-coding genes in *P. generosa* genome assembly

Supplementary Table S6. The enriched KEGG pathways of expanded genes in *P. generosa* genome assembly.

Abbreviations

Akt: RAC serine/threonine-protein kinase; bp: base pairs; BLAST: Basic Local Alignment Search Tool; BUSCO: Benchmarking Universal Single-Copy Orthologs; BWA: Burrows-Wheeler Aligner; cAMP: cyclic adenosine monophosphate; CAMs: cell adhesion molecules; cGMP-PKG: cGMP-dependent protein kinase G; Gb: gigabase pairs; GC: guanine-cytosine; GnRH: Gonadotropin-releasing hormone; GO: gene ontology; Iso-seq: Hi-C: High-throughput/resolution chromosome conformation capture; Isoform sequencing; kb: kilobase pairs; KEGG: Kyoto Encyclopedia of Genes and Genomes; Mb: megabase pairs; MYA: million years ago; NCBI: National Center for Biotechnology Information; NF-kappa B: nuclear factor kappa-B; NOD-like receptor: nucleotide-binding oligomerization domain-like receptor; PacBio: Pacific Biosciences; RAxML: PI3K: phosphatidylinositol-4,5-bisphosphate 3-kinase catalytic subunit alpha/beta/delta; Randomized Axelerated Maximum Likelihood; Rap1: Ras-related protein1; RNA-seq: RNA sequencing; Iso-seq: Isoform-sequencing; tRNA: transfer RNA; TRP channel: transient receptor potential ion channel.

Competing Interests

The authors declare that they have no competing interests.

Funding

This study was supported by the Strategic Priority Research Program of Chinese

Academy of Sciences (XDB42000000), the Chinese Academy of Sciences Pioneer Hundred Talents Program (to Nansheng Chen), the Taishan Scholar Project Special Fund (to Nansheng Chen), and the Qingdao Innovation and Creation Plan (Talent Development Program-5th Annual Pioneer and Innovator Leadership Award to Nansheng Chen, 19-3-2-16-zhc) and an Earmarked Workstation Fund for QRJH (to Chunde Wang and Nansheng Chen).

Authors' Contributions

N.C. and C.W. conceived and designed the study. M.C. and Y.C. prepared the samples. J.W. and Q.X. performed analyses. J.W. wrote the paper with input from co-authors. All authors read and approved the final version for submission.

References

1. González-Peláez SS, Leyva-Valencia I, Pérez-Valencia SA, et al. Distribution limits of the geoduck clams *Panopea generosa* and *P. globosa* on the Pacific coast of Mexico. Malacologia. 2013;56:85-94.
2. Vadopalas B, Pietsch TW, Friedman CS. The proper name for the geoduck: resurrection of *Panopea generosa* Gould , 1850, from the synonymy of *Panopea abrupta* (Conrad, 1849) (Bivalvia: Myoida: Hiatellidae). Malacologia. 2010;52:169-73.
3. Goodwin CL, Pease BC. Geoduck, *Panopea abrupta* (Conrad, 1849), size, density, and quality as related to various environmental parameters in Puget Sound, Washington. J Shellfish Res 1991;10:65-77.
4. Orensanz JM, Hand CM, Parma AM, et al. Precaution in the harvest of Methuselah's clams the difficulty of getting timely feedback from slow-paced dynamics. Can J Fish Aquat Sci. 2004;61:1355-72.
5. Newell RIE. Ecosystem influences of natural and cultivated populations of suspension-feeding bivalve molluscs: A review. J Shellfish Res. 2004;23:51-61.
6. Straus KM, MacDonald PS, Crosson LM, et al. Effects of geoduck aquaculture on the environment: A synthesis of current knowledge., Washington Sea Grant Technical Report WSG-TR 13-02, 2013.
7. Santos A, Aguirre J, Rodríguez-Tovar FJ, et al. Multi-storm events recorded on *Panopea* burrows (Pliocene, Spain): The importance of sequestered information inside burrows. Palaeogeogr, Palaeoclimatol, Palaeoecol. 2018;507:155-67.
8. Bureau D, Hajas W, Hand CM, et al. Age, size structure and growth parameters of

geoducks (*Panopea abrupta*, Conrad 1849) from seven locations in British Columbia sampled in 2001 and 2002. Canadian Technical Report of Fisheries and Aquatic Sciences, 2003, p. 29.

9. Sloan NA, Robinson SMC. Age and gonad development in the geoduck clam *Panopea abrupta* (Conrad) from southern British Columbia, Canada. J Shellfish Res. 1984;4:131-7.

10. Valero JL, Canada O, Madryn P, et al. Geoduck (*Panopea abrupta*) recruitment in the Pacific Northwest: long-term changes in relation to climate. CalCOFI Reports. 2004;45:80-6.

11. Marçais G, Kingsford C. A fast, lock-free approach for efficient parallel counting of occurrences of k-mers. Bioinformatics. 2011;27:764-70.

12. Walker BJ, Abeel T, Shea T, et al. Pilon: an integrated tool for comprehensive microbial variant detection and genome assembly improvement. PloS one. 2014;9:e112963.

13. Burton JN, Adey A, Patwardhan RP, et al. Chromosome-scale scaffolding of de novo genome assemblies based on chromatin interactions. Nat Biotechnol. 2013;31:1119-25.

14. Pendleton M, Sebra R, Pang AW, et al. Assembly and diploid architecture of an individual human genome via single-molecule technologies. Nat Methods. 2015;12:780-6.

15. Dudchenko O, Batra SS, Omer AD, et al. De novo assembly of the *Aedes aegypti* genome using Hi-C yields chromosome-length scaffolds. Science. 2017;356:92-5.

16. Robinson JT, Turner D, Durand NC, et al. Juicebox.js provides a cloud-based

visualization system for Hi-C data. Cell Syst. 2018;6:256-8.

17. Simão FA, Waterhouse RM, Ioannidis P, et al. BUSCO: assessing genome assembly and annotation completeness with single-copy orthologs. Bioinformatics. 2015;31:3210-2.

18. Bergman CM, Quesneville H. Discovering and detecting transposable elements in genome sequences. Brief Bioinform. 2007;8:382-92.

19. Bao W, Kojima KK, Kohany O. Repbase Update, a database of repetitive elements in eukaryotic genomes. Mobile DNA. 2015;6:11.

20. Edgar RC, Myers EW. PILER: identification and classification of genomic repeats. Bioinformatics. 2005;21:i152-8.

21. Price AL, Jones NC, Pevzner PA. De novo identification of repeat families in large genomes. Bioinformatics. 2005;21 Suppl 1:i351-8.

22. Flynn JM, Hubley R, Goubert C, et al. RepeatModeler2 for automated genomic discovery of transposable element families. Proc Natl Acad Sci U S A. 2020;117:9451-7.

23. Benson G. Tandem repeats finder: a program to analyze DNA sequences. Nucleic Acids Res. 1999;27:573-80.

24. Altschul SF, Gish W, Miller W, et al. Basic local alignment search tool. J Mol Biol. 1990;215:403-10.

25. Yu XJ, Zheng HK, Wang J, et al. Detecting lineage-specific adaptive evolution of brain-expressed genes in human using rhesus macaque as outgroup. Genomics. 2006;88:745-51.

26. Birney E, Clamp M, Durbin R. GeneWise and Genomewise. Genome Res. 2004;14:988-95.

27. Stanke M, Morgenstern B. AUGUSTUS: a web server for gene prediction in eukaryotes that allows user-defined constraints. Nucleic Acids Res. 2005;33:W465-7.

28. Guigó R, Knudsen S, Drake N, et al. Prediction of gene structure. J Mol Biol. 1992;226:141-57.

29. Burge C, Karlin S. Prediction of complete gene structures in human genomic DNA. J Mol Biol. 1997;268:78-94.

30. Majoros WH, Pertea M, Salzberg SL. TigrScan and GlimmerHMM: two open source ab initio eukaryotic gene-finders. Bioinformatics. 2004;20:2878-9.

31. Solovyev V, Kosarev P, Seledsov I, et al. Automatic annotation of eukaryotic genes, pseudogenes and promoters. Genome Biol. 2006;7:S10.

32. Korf I. Gene finding in novel genomes. BMC bioinf. 2004;5:59.

33. Kim D, Pertea G, Trapnell C, et al. TopHat2: accurate alignment of transcriptomes in the presence of insertions, deletions and gene fusions. Genome Biol. 2013;14:R36.

34. Trapnell C, Roberts A, Goff L, et al. Differential gene and transcript expression analysis of RNA-seq experiments with TopHat and Cufflinks. Nat Protoc. 2012;7:562-78.

35. Wu TD, Watanabe CK. GMAP: a genomic mapping and alignment program for mRNA and EST sequences. Bioinformatics. 2005;21:1859-75.

36. Haas BJ, Delcher AL, Mount SM, et al. Improving the Arabidopsis genome annotation using maximal transcript alignment assemblies. Nucleic Acids Res. 2003;31:5654-66.

37. Haas BJ, Salzberg SL, Zhu W, et al. Automated eukaryotic gene structure annotation

using EVidenceModeler and the Program to Assemble Spliced Alignments. Genome Biol. 2008;9:R7.

38. Li L, Stoeckert CJ, Jr., Roos DS. OrthoMCL: identification of ortholog groups for eukaryotic genomes. Genome Res. 2003;13:2178-89.

39. Edgar RC. MUSCLE: multiple sequence alignment with high accuracy and high throughput. Nucleic Acids Res. 2004;32:1792-7.

40. Talavera G, Castresana J. Improvement of phylogenies after removing divergent and ambiguously aligned blocks from protein sequence alignments. Syst Biol. 2007;56:564-77.

41. Stamatakis A. RAxML-VI-HPC: maximum likelihood-based phylogenetic analyses with thousands of taxa and mixed models. Bioinformatics. 2006;22:2688-90.

42. Yang Z. PAML 4: phylogenetic analysis by maximum likelihood. Mol Biol Evol. 2007;24:1586-91.

43. Thorne JL, Kishino H, Painter IS. Estimating the rate of evolution of the rate of molecular evolution. Mol Biol Evol. 1998;15:1647-57.

44. Vogel JP, Garvin DF, Mockler TC, et al. Genome sequencing and analysis of the model grass *Brachypodium distachyon*. Nature. 2010;463:763-8.

45. Blanc G, Wolfe KH. Widespread paleopolyploidy in model plant species inferred from age distributions of duplicate genes. The Plant cell. 2004;16:1667-78.

46. Sanderson MJ. r8s: inferring absolute rates of molecular evolution and divergence times in the absence of a molecular clock. Bioinformatics. 2003;19:301-2.

47. Han MV, Thomas GW, Lugo-Martinez J, et al. Estimating gene gain and loss rates in

the presence of error in genome assembly and annotation using CAFE 3. Mol Biol Evol. 2013;30:1987-97.

48. Thai BT, Lee YP, Gan HM, et al. Whole genome assembly of the snout otter clam, *Lutraria rhynchaena*, using Nanopore and Illumina Data, benchmarked against bivalve genome assemblies. Front Genet. 2019;10:1158.

49. Sun J, Zhang Y, Xu T, et al. Adaptation to deep-sea chemosynthetic environments as revealed by mussel genomes. Nat Ecol Evol. 2017;1:0121.

50. Peñaloza C, Gutierrez AP, Eöry L, et al. A chromosome-level genome assembly for the Pacific oyster *Crassostrea gigas*. Gigascience. 2021;10:giab020.

51. Peng J, Li Q, Xu L, et al. Chromosome-level analysis of the *Crassostrea hongkongensis* genome reveals extensive duplication of immune-related genes in bivalves. Mol Ecol Resour. 2020;20:980-94.

52. Wu B, Chen X, Yu M, et al. Chromosome-level genome and population genomic analysis provide insights into the evolution and environmental adaptation of Jinjiang oyster *Crassostrea ariakensis*. Mol Ecol Resour. 2022;22:1529-44.

53. Du X, Fan G, Jiao Y, et al. The pearl oyster *Pinctada fucata martensii* genome and multi-omic analyses provide insights into biomineralization. Gigascience. 2017;6:1-12.

54. Yang JL, Feng DD, Liu J, et al. Chromosome-level genome assembly of the hard-shelled mussel *Mytilus coruscus*, a widely distributed species from the temperate areas of East Asia. Gigascience. 2021;10:giab024.

55. McCartney MA, Auch B, Kono T, et al. The genome of the zebra mussel, *Dreissena polymorpha*: a resource for comparative genomics, invasion genetics, and biocontrol.

G3 Genes Genom Genet 2021;12:jkab423.

56. Zhang T, Yin J, Tang S, et al. Dissecting the chromosome-level genome of the Asian Clam (*Corbicula fluminea*). Sci Rep. 2021;11:15021.

57. Ran Z, Li Z, Yan X, et al. Chromosome-level genome assembly of the razor clam *Sinonovacula constricta* (Lamarck, 1818). Mol Ecol Resour. 2019;19:1647-58.

58. Li J, Zhou Y, Zhou Z, et al. Comparative transcriptome analysis of three gonadal development stages reveals potential genes involved in gametogenesis of the fluted giant clam (*Tridacna squamosa*). BMC genomics. 2020;21:872.

59. Jiang H, Liu H, Ma X, et al. Transcriptome analysis of *Procambarus clarkii* to screen genes related to ovary development, immunity and growth. J Fish China. 2021;45:396-414.

60. Ren Y, Liu W, Pearce CM, et al. Effects of selected mixed-algal diets on growth and survival of early postset juveniles of the Pacific geoduck clam, *Panopea generosa* (Gould, 1850). Aquacult Nutr. 2014;21.

61. Nava-Gómez GE, Garcia-Esquivel Z, Carpizo-Ituarte E, et al. Survival and growth of geoduck clam larvae (*Panopea generosa*) in flow-through culture tanks under laboratory conditions. Aquacult Res. 2018;49:294-300.

62. Li H, Liu M, Ye S, et al. De novo assembly, gene annotation, and molecular marker development using Illumina paired-end transcriptome sequencing in the clam *Saxidomus purpuratus*. Genes Genom. 2017;39:675-85.

63. Hu F, Pan L, Cai Y, et al. Deep sequencing of the scallop *Chlamys farreri* transcriptome response to tetrabromobisphenol A (TBBPA) stress. Mar Genom. 2015;19:31-8.

Figure 1. The *P. generosa* genome contig contact matrix using Hi-C data and landscape. (A) Hi-C analysis of *P. generosa* genome contigs. Chromosomes are arranged in size order from left to right and from top to bottom. The color bar illuminates the logarithm of the contact density from red (10) to white (0) in the plot. (B) The genomic landscape of *P. generosa*: from outer to inner circles: a, the 19 chromosomes; b–d, repeat density, gene density, and GC density across the genome, respectively, drawn in 1 Mb non-overlapping windows.

Figure 2. The distribution of single-copy, multiple-copy, unique, other orthologs, and unclustered genes in *P. generosa* and related species.

Figure 3. Distribution of shared gene families among *P. generosa*, *P. martensi*, *S. broughtonii*, and *P. yessoensis*. Intersections between species indicate the numbers of shared gene families, whereas unique family numbers are shown in species-specific areas. The center represents the number of families shared by all species.

Figure 4. Phylogenetic analysis of *P. generosa* with related species. The estimated species divergence time (million years ago) and the 95% confidential intervals are labeled at each branch site. Divergence times used for time recalibration is illuminated as red dots in the tree.

Figure 5. Dynamic evolution and distribution of gene families among *P. generosa* and

related species. Green and red numbers represent gene family expansion and contraction, respectively. MRCA: most recent common ancestor.

Figure1

Click here to access/download;Figure;Figure1.pdf

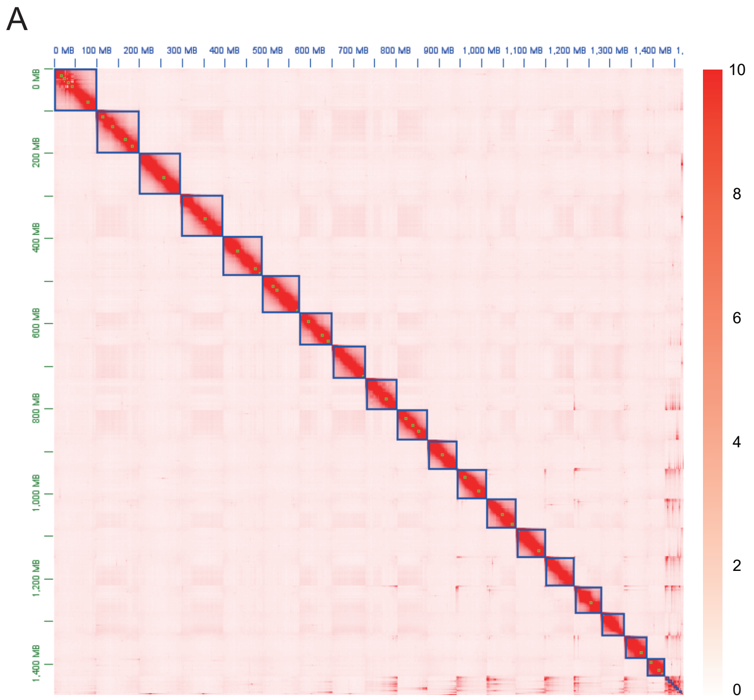

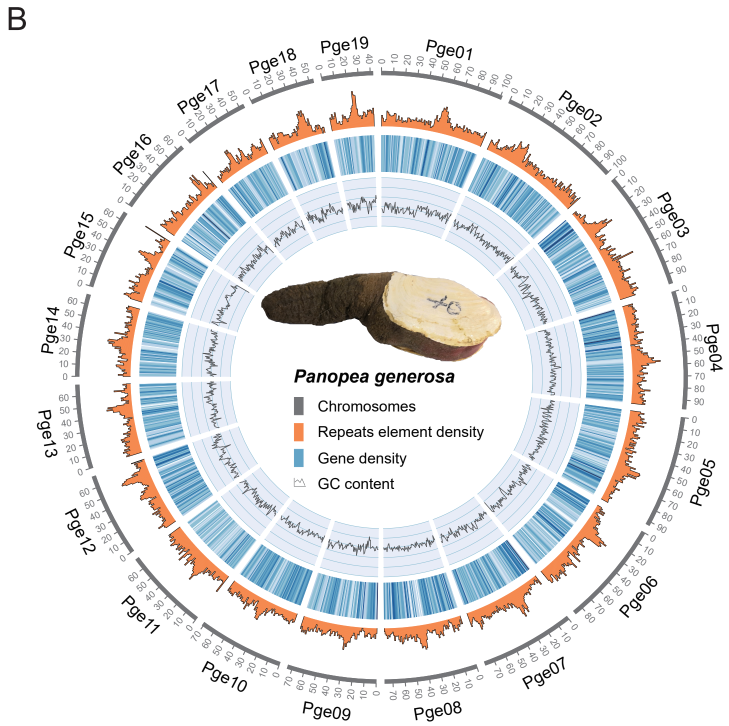

Figure2

Click here to access/download;Figure;Figure2.pdf

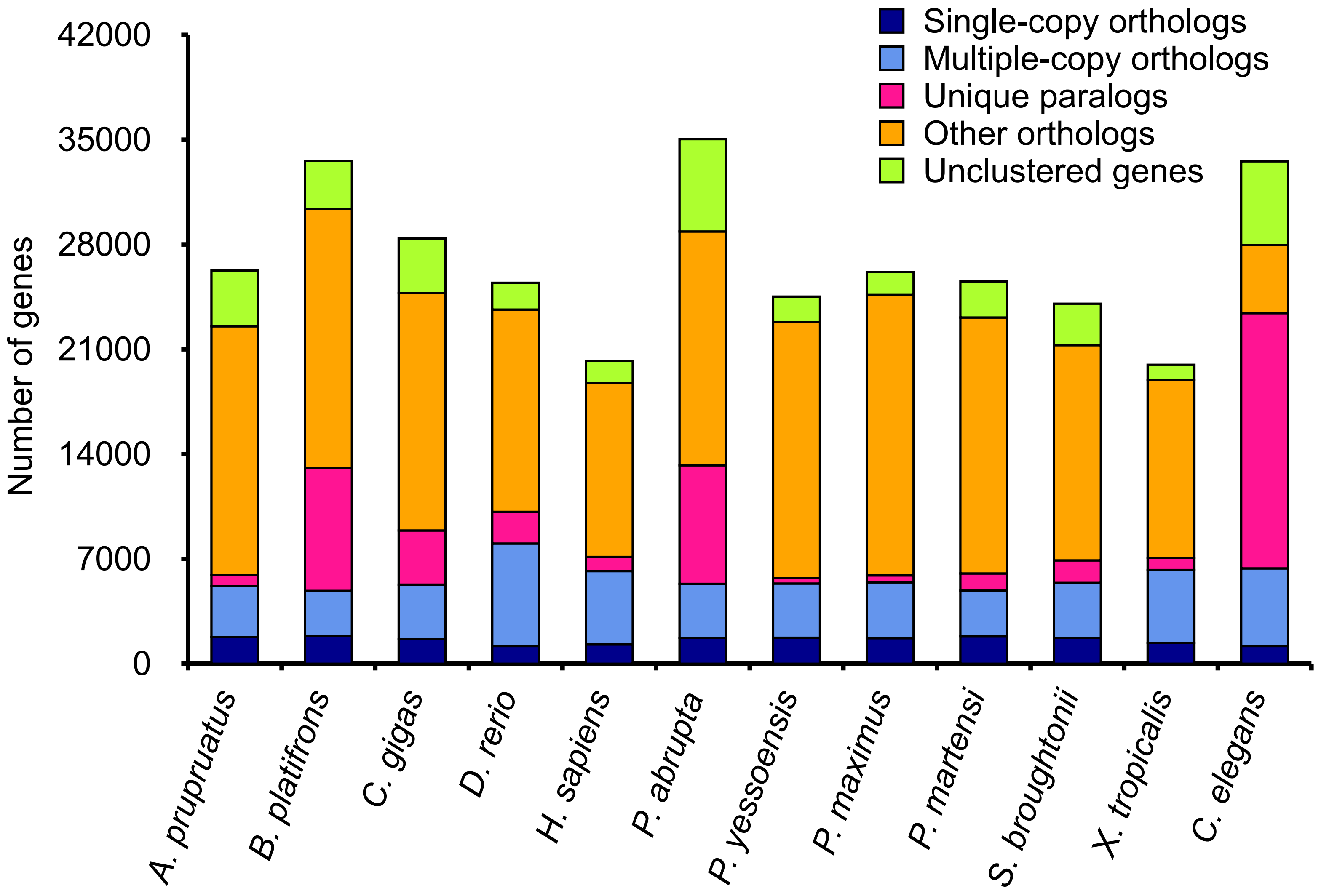

Figure3 Click here to access/download;Figure;Figure3.pdf

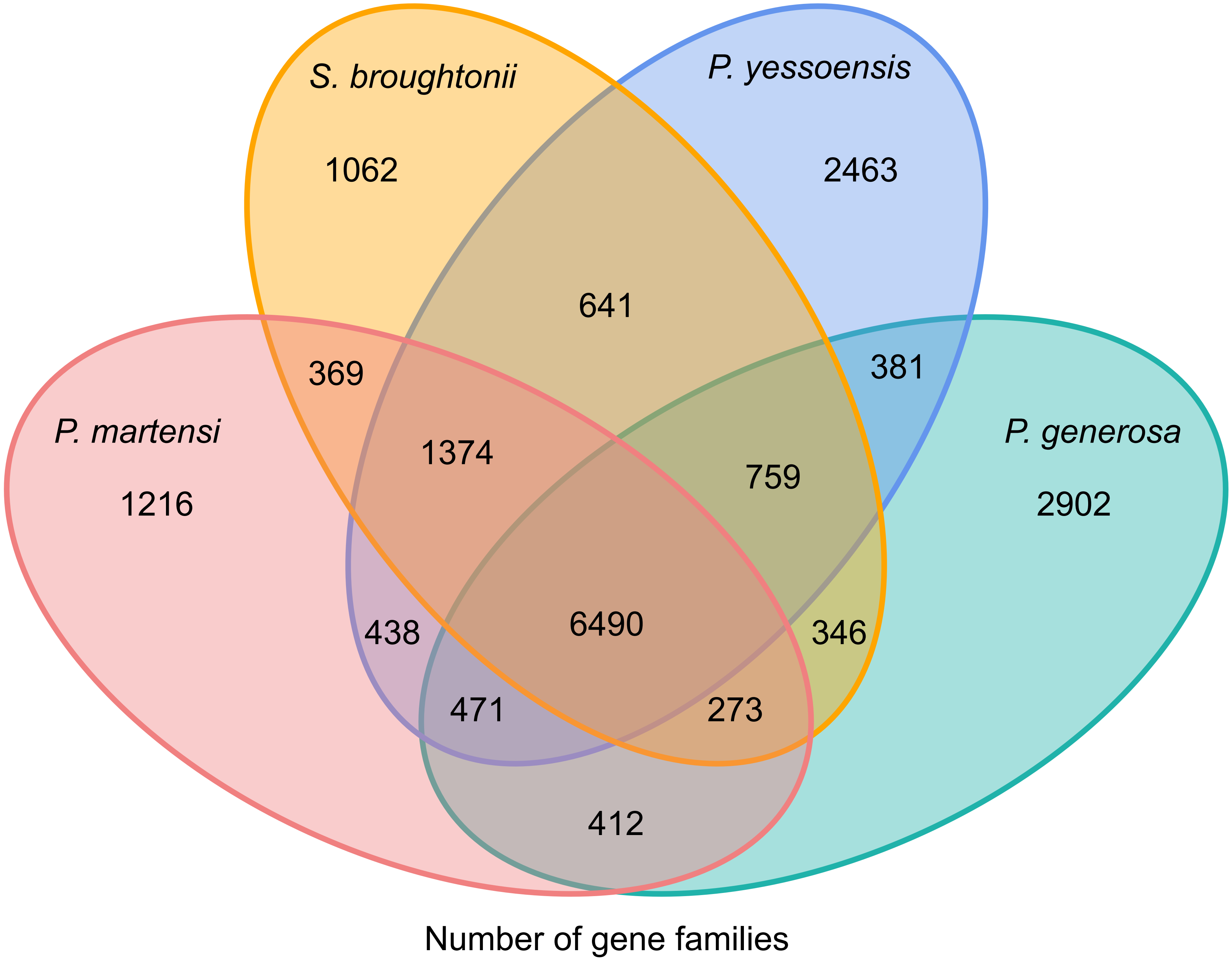

Figure4

Click here to access/download;Figure;Figure4.pdf

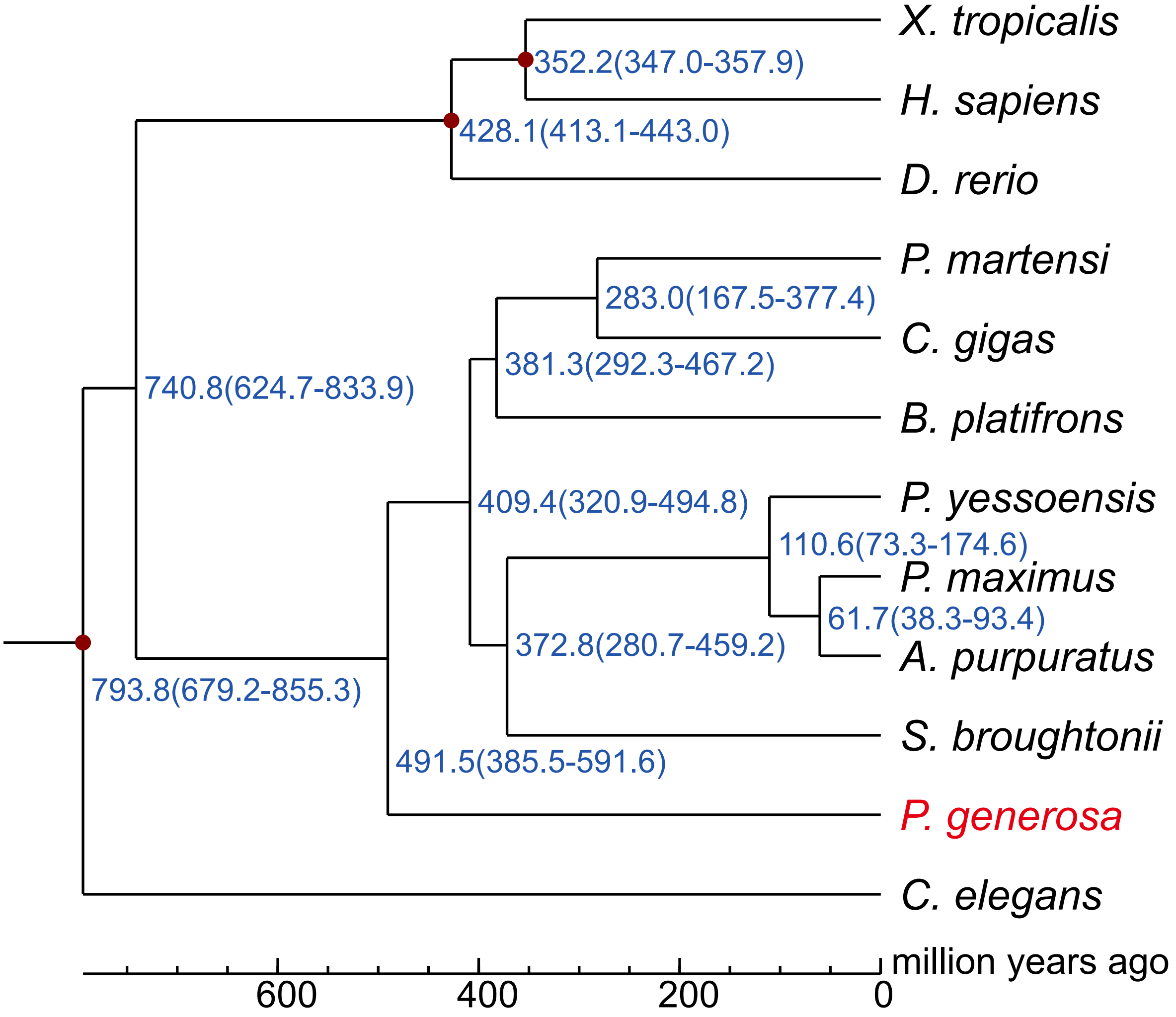

Figure5

Click here to access/download;Figure;Figure5.pdf

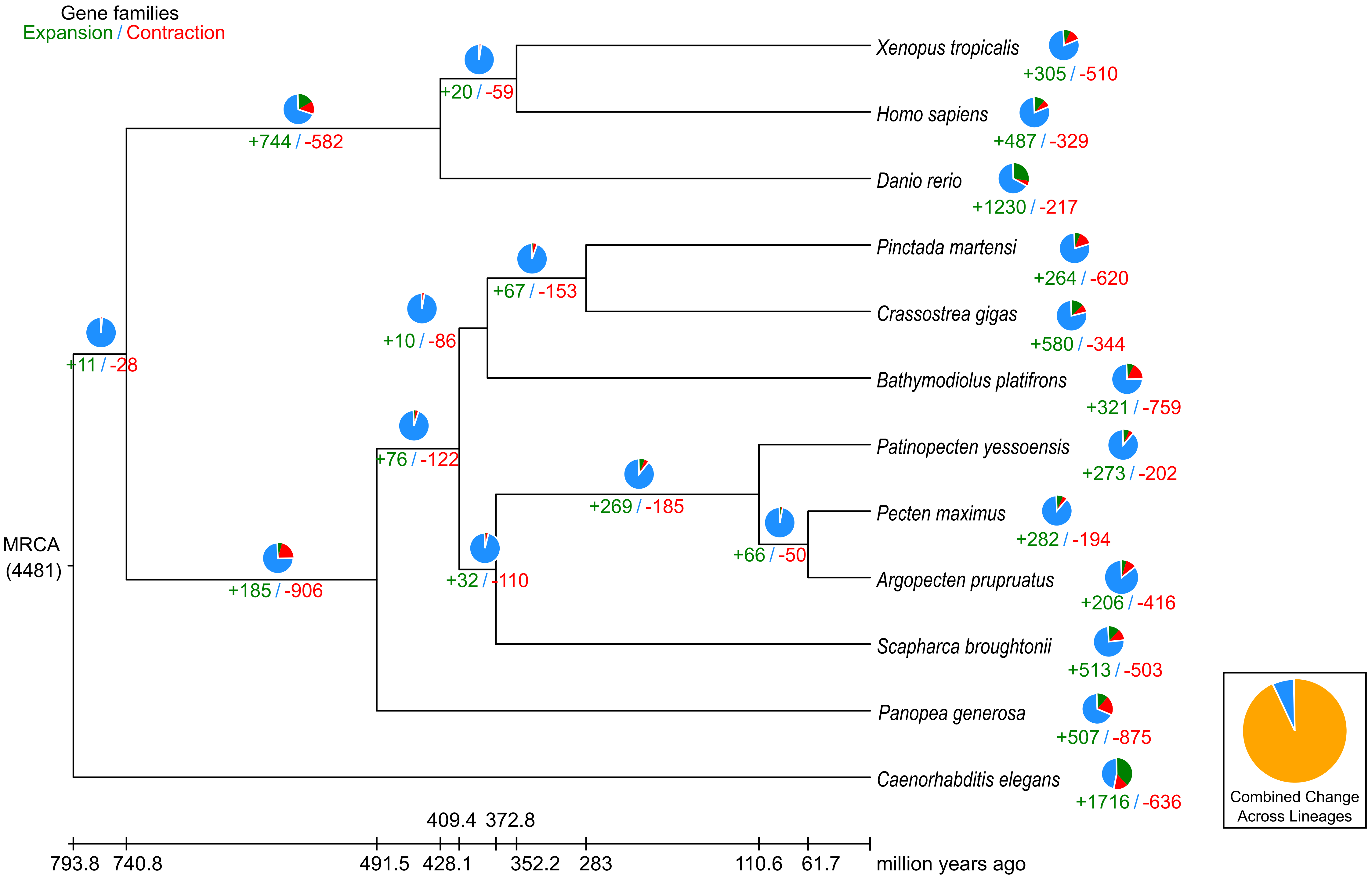

Click here to access/download
Supplementary Material
Supplementary Information.docx

Cover letter Click here to access/download;Personal Cover;cover_letter.docx

Dear Editor,

I am submitting our manuscript entitled “Chromosome-level genome assembly of the Pacific geoduck *Panopea generosa*” for your consideration to publish in GigaScience.

The Pacific geoduck *Panopea generosa* is a member of genus *Panopea* that includes the world's largest burrowing bivalves. In this manuscript, we report the first chromosome-scale genome assembly for *P. generosa*, which enables high-quality comparative analysis of genome assemblies of bivalves. Through comparative analysis, we identified differences between gene families of *P. generosa* and *Sinonovacula constricta*, a razor clam, which provide insights into its adaptation, growth, development and immunity.

We confirm that work described in this manuscript is original, and we have all authors’ permission to submit this manuscript to Gigascience. We also confirm that none of the material or related manuscripts has been published or is under consideration elsewhere, including the Internet. All authors have contributed to the creation of this manuscript for important intellectual content and approved the final manuscript. We declare there is no conflict of interest.

We appreciate your consideration and look forward to your response.

Yours sincerely,

Nansheng Chen
Key Laboratory of Marine Ecology and Environmental Sciences
Institute of Oceanology, Chinese Academy of Sciences
Add: 7 Nanhai Road, Qingdao 266071, China
Tel: +86-532-82893507
E-mail: chenn@qdio.ac.cn
