# Supplementary material for: Chromosome-level genome assembly of the Pacific geoduck Panopea generosa reveals major inter- and intrachromosomal rearrangements and substantial expansion of the copine gene family
Source: Gigascience. 2023 Dec 19;12:giad105. doi: 10.1093/gigascience/giad105 (PMC10729735; doi:10.1093/gigascience/giad105)
Supplement: giad105_GIGA-D-22-00284_Revision_1 [file giad105_giga-d-22-00284_revision_1.pdf]

GigaScience

Chromosome-level genome assembly of the Pacific geoduck Panopea generosa reveals major inter- and intra-chromosomal rearrangements and substantial expansion of the copine gene family

--Manuscript Draft--

**Manuscript Number:**	GIGA-D-22-00284R1
**Full Title:**	Chromosome-level genome assembly of the Pacific geoduck Panopea generosa reveals major inter- and intra-chromosomal rearrangements and substantial expansion of the copine gene family
**Article Type:**	Data Note
**Funding Information:**	Taishan Scholar Foundation of Shandong Province — Professor Nansheng Chen<br>Strategic Priority Research Program of Chinese Academy of Sciences (XDB42000000) — Professor Nansheng Chen<br>Chinese Academy of Sciences Pioneer Hundred Talents Program — Professor Nansheng Chen<br>Earmarked Workstation Fund for QRJH — Professor Chunde Wang, Professor Nansheng Chen
**Abstract:**	The Pacific geoduck Panopea generosa (class Bivalvia, order Adapedonta, family Hiatellidae, genus Panopea) is the largest known burrowing bivalve with considerable commercial value. Pacific geoduck and other geoduck clams play important roles in maintaining ecosystem health for their filter feeding habit and coupling pelagic and benthic processes. Chromosome-level genomes of geoduck clams will contribute to genetic breeding, as well as ecosystem and climate change biology. Here, we report the first high-quality chromosome-level genome assembly of P. generosa with the purpose to unravel its phylogenetic characteristics and molecular mechanisms of its life strategies, and promote research on genetic breeding. The assembled P. generosa genome consists of 19 chromosomes with a size of 1.47 Gb, a contig N50 of 1.6 Mb and a scaffold N50 of 73.8 Mb. BUSCO analysis showed 93.0% completeness. Comparative analysis of the genomes of two closely related species in the order Adapedonta, P. generosa and Sinonovacula constricta, revealed major inter- and intra-chromosomal exchanges. Of the 35,034 predicted protein-coding genes (PCGs), 30,700 genes (87.63%) were functionally annotated. Comparative analysis of the genomes of P. generosa and 11 related species identified 507 expanded gene families and 875 contracted gene families in P. generosa. Enrichment analysis revealed significant expansion of immune and gonad development gene families that may promote its complex survival strategies. In particular, the copine gene family, which plays an important role in calcium signaling, membrane trafficking, and cytoskeletal dynamics, and has been implicated in several physiological and pathological processes such as neuronal development, immune response substantially expanded in P. generosa with 22 members annotated. The availability of a chromosome-level P. generosa genome assembly and its annotated gene set provide a useful molecular platform for research on its genetic breeding.
**Corresponding Author:**	Nansheng Chen<br>Institute of Oceanology Chinese Academy of Sciences<br>Qingdao, Shandong CHINA
**Corresponding Author Secondary Information:**	
**Corresponding Author's Institution:**	Institute of Oceanology Chinese Academy of Sciences
**Corresponding Author's Secondary Institution:**	
**First Author:**	Jing Wang
**First Author Secondary Information:**	

Powered by Editorial Manager® and ProduXion Manager® from Aries Systems Corporation

**Order of Authors:**	Jing Wang
	Qing Xu
	Min Chen
	Yang Chen
	Chunde Wang
	Nansheng Chen
**Order of Authors Secondary Information:**	
**Response to Reviewers:**	Reviewer #1: The manuscript by Wang et al. reports the high-quality chromosome-level assembly of the economically important Pacific geoduck Panopea generosa. While I believe that this work will be of significant value to the scientific community, the manuscript requires significant rephrasing and additional details added before it can be published. Specifically, a lot of additional details need to be provided in the methods section, including which tools were used, their versions and parameters, as well as clarification of the points that I detail below. Rephrasing and additional details are also required in the results section to improve understanding by the reader. I also could not find Supplementary Tables S2 and S6 online. If I have missed them then I apologies, however, if they we not uploaded then this needs to be corrected for the next round of reviewers. Please see my specific comments below.<br>Response 1: We thank the reviewer for confirming the value of our work and for providing valuable suggestions for improving the manuscript. We have made additional analyses and have substantially revised the manuscript. Details are provided below.<br><br>Page 5: "They are preys for" should read "They are prey for"<br>Response 2: Corrected.<br><br>Page 5: "shell concentrations" of what? Did you mean "shell composition"? Also, I am unsure what you mean by "sequence of events related to storm events". What specifically are these events that are related to storms?<br>Response 3: These are terms used in palaeogeography, palaeoclimatology, and palaeoecology. The “shell concentrations” is used as a tool for palaeoenvironmental reconstructions and palaeoecological interpretations, has been increasingly recognized in the last decades with a wealth of publications (Santos A et al., Palaeogeography, Palaeoclimatology, Palaeoecology. 2018;507:155-67.). As Panopea species may reach 160 years in age and usually occupies a dynamic depositional setting, the trace fossils produced by this bivalve may provide a clear record of the long-term ability to respond to changes in the physical environments. As a result, they may be used as proxies for estimating the magnitude and frequency of sedimentation and erosion events.<br><br>Page 6: "gene family expansion and contraction" should probably read "gene family expansion and contraction analysis"<br>Response 4: Revised.<br><br>Page 6: "Genomic DNA of P. generosa was extracted using a standard phenol-chloroform extraction method" I understand that this process is commonly used for DNA extraction however additional detail about your exact application of the method or at minimum references to the protocol that you followed is required to assist the reader.<br>Response 5: A reference is added in the revised manuscript.<br><br>Page 7: "Sequence libraries with insert size of 300 bp were constructed" by who? A service provider? Using which protocol? This needs to be listed.<br>Response 6: Sequencing libraries were constructed by a sequence company, BGI Genomics, BGI-Shenzhen (China) using protocols according to manufacturer, which is Whole Genome Sequencing Library Preparation (DNBSEQ) BGI-NGS-JK-DNA-001 A0”. This information is included in the revised manuscript.<br><br>Page 7: "genome size estimation, k-mer analysis", weren't k-mers used to estimate genome size? This sentence makes it sound like they are two separate processes.<br>Response 7: We have revised this sentence in the revised manuscript.

	Page 7: "Hi-C library with insert size of 300 bp was constructed". Who constructed the library, using what protocol, and which company actually did the sequencing?<br>Response 8: The sequence libraries were constructed by a sequence company, BGI Genomics, BGI-Shenzhen (China). The protocol has been supplemented in the main text.<br><br>Page 7: "The raw sequence data generated …..". List programs, versions, and parameters used to filter the BGIseq and PacBio reads.<br>Response 9: Programs, versions, and parameters used to filter the BGISEQ and PacBio reads have been added in the revised manuscript.<br><br>Page 7: "Each RNA sample was qualified and". I don't think that this is the correct use of the word "qualified". Maybe try "The quality and quantity of RNA in each sample was assessed using a NanoDrop …."<br>Response 10: The description has been rephrased as “The quality and quantity of RNA in each sample was assessed using a NanoDrop and an Agilent 2100 bioanalyzer (Thermo Fisher Scientific, MA, USA)”.<br><br>Page 7: "For RNA-seq, the mRNA library was constructed by purification, fragmentation, a first-strand cDNA generation, a second-strand cDNA synthesis, and RNA index addition." List kits and protocols used for this analysis.<br>Response 11: The detailed information of kits and protocols used for RNA-seq has been added in the revised manuscript.<br><br>Page 8: "Counting of k-mers was conducted using Jellyfish". Which other tool or method was used to estimate genome size after k-mers were produced by jellyfish? Based on Figure S1 I would guess that you used your own workflow for genome size estimation. A description and references for this approach is required. Also, the "k" in k-mers should be italicized through the manuscript.<br>Response 12: We only applied jellyfish for estimating the genome size in this project, which is important for determining the quantity of PacBio sequencing needed for the genome assembly. Spelling has been revised in the revised manuscript.<br><br>Page 8: "Short paired-end clean reads from BGIseq-500 were then polished using Pilon" The short reads were used by Pilon to correct the assembly? This sentence makes it seem like Pilon was used to correct the short reads, which I don't think is correct.<br>Response 13: The sentence has been corrected as “Short paired-end clean reads from BGISEQ-500 were then used for correcting post-processing errors and resolving conflicts of assembly via Pilon (RRID:SCR_014731, version 1.22) [33]”.<br><br>Page 8: "Chromosomes were further corrected using Juicebox". More detail is required here since I assume you did some manual corrections using Juicebox. If you just used an automated correction workflow then these details need to be given.<br>Response 14: We have rephrased this part in the revised manuscript.<br><br>Page 8: "The completeness of genome assembly was assessed using BUSCO". Using which lineage dataset?<br>Response 15: The metazoa_odb10 was used in the BUSCO analysis. This information is added in the revised manuscript.<br><br>Page 8: (1) "In homologous prediction, RepeatMasker (RepeatMasker, RRID:SCR_012954) and the associated RepeatProteinMask [18] were performed by alignment against Repbase library (v21.12)". This sentence is a little hard to follow. I assume that you mean the repeats from Repbase were aligned against the reference genome using the mentioned programs? Based on how it is currently phrased, it is unclear what is being aligned against Repbase. (2) Also please provide details on any parameters given to each program when they are run. The following sentence can also be restructured to make it clear that repeats were identified de novo in the genome assembly and what the parameters used for each of the tools were.<br>Response 16:<br>(1) This sentence has been corrected as “In homologous prediction, RepeatMasker (RepeatMasker, RRID:SCR_012954) and RepeatProteinMask [18] were used to screen the P. generosa genome for known transposable elements in the RepBase

library (Repbase, RRID:SCR_021169) [19]".
(2) The following sentence has been corrected as "In de novo prediction, RepeatModeler (version 1.0.4) was first used for de novo candidate database construction of repetitive elements, and repetitive sequences were then annotated using RepeatMasker.".

Page 8: "Besides, tandem repeats". Remove the word "Besides" from this sentence.
Response 17: Removed.

Page 9: "The results were then integrated and redundancy was eliminated." How was redundancy eliminated? Using which tool?
Response 18: For clarity, we have changed "redundancy" to "duplicate".

Page 9: "from different branches were chosen to confirm the completeness of the gene set." I am unsure what you mean by this sentence. Do you mean one genome per taxonomic group was selected? What is mean by "to confirm the completeness"?
Response 19: This sentence has been rephrased as "For homology-based prediction, gene sets from eight closely related species in bivalves (Patinopecten yessoensis, Pinctada fucata, Mytilus galloprovincialis, Limnoperna fortune, Argopecten purpuratus, Sinonovacula constricta, Scapharca broughtonii, and Crassostrea gigas) were used".

Page 9: "hits were then conjoined by Solar software (SOLAR, RRID:SCR_000850) [25]." I don't think that this software is a gene prediction tool. From what I can tell it is a QLT analysis tool. Is this correct?
Response 20: Exonerate v2.2.0 was used to predict the gene structures.

Page 9: "For de novo annotation, six programs were simultaneously used…..". Version numbers and parameters are required for each of these tools.
Response 21: Details of the version numbers and parameters are provided in the revised manuscript.

Page 9: "Among them, Augustus, snap and GlimmerHMM were trained using PASA-T-set gene models." What is the "PASA-T-set"? This set of (I assume) RNA-seq based gene modes is never defined in the text.
Response 22: We have rephrased this part in the revised manuscript.

Page 10: "Gene predictions from the homology-based approach, …." List version, parameters, and evidence weights used for EvidenceModeler. Also, list lineage dataset used for BUSCO analysis.
Response 23: The description has been rephrased as "Gene predictions from the homology-based approach, de novo approach, RNA-Seq-based and Iso-Seq-based evidences were merged, and redundancy was removed to form a comprehensive consensus gene set using Maker 2 (RRID:SCR_005309) [52]". To validate the completeness of the gene structure annotation, we also used BUSCO (version5.4.3) with the metazoa_odb10 database [23].

Page 10: "Gene families were constructed according to OrthoMCL (OrthoMCL DB: Ortholog Groups of Protein Sequences, RRID:SCR_007839) pipeline" should probably read "Gene families were constructed using the OrthoMCL (OrthoMCL DB: Ortholog Groups of Protein Sequences, RRID:SCR_007839) pipeline"
Response 24: Corrected.

Page 10: "… Caenorhabditis elegans) were filtered. When multiple …" The transition between these two sentences is a little hard to follow. When I first read it, I didn't immediately understand that the following sentence lists the filtering steps. Please rephrase this section.
Response 25: These two sentences were rephrased as "We selected P. generosa and other 11 species (C. gigas, P. yessoensis, P. maximus, A. purpuratus, S. broughtonii, Pinctada martensi, Bathymodiolus platifrons, Homo sapiens, Xenopus tropicaalis, Danio rerio, and Caenorhabditis elegans) for gene family analysis. For the gene set of each genome, only the transcript with the longest coding sequence was selected from alternate splice transcripts. Genes with less than 50 amino acids were removed from further analysis.".

Powered by Editorial Manager® and ProduXion Manager® from Aries Systems Corporation

	Page 10: "(E value = 1e-5 by default) [24]" what does the "by default" mean here? If you are setting the e-value to be 1e-5 then you are not running the program using default parameters?<br>Response 26: The words “by default” have been removed from the manuscript.<br><br>Page 10-11: "SCR_007839) 2.0 with the expansion coefficient 1.5 [38]. Finally, to perform multiple sequence alignment for single copy genes clustered, and concatenate sequence alignment of single-copy gene families. Based on the clustering results, the division of gene family was obtained. To construct phylogenetic trees, protein sequences of each single copy gene of P. generosa and other 11 species were aligned using MUSCLE (MUSCLE, RRID:SCR_011812) [39], and then the protein alignment results were converted into CDS alignment results."<br>I am slightly confused by this section. You selected gene clusters that were single copy in how many species? What does "Based on the clustering results, the division of gene family was obtained" Mean? Do you mean that each cluster was considered a gene family? How were the protein alignment converted to CDS alignment? Using which tool? Please rephrase this section to make your approach clearer.<br>Response 27:<br>(1) We selected gene clusters that were single copy in P. generosa and 11 other species (C. gigas, P. yessoensis, P. maximus, A. purpuratus, S. broughtonii, Pinctada martensi, Bathymodiolus platifrons, Homo sapiens, Xenopus tropicaalis, Danio rerio, and Caenorhabditis elegans).<br>(2) The sentence "Based on the clustering results, the division of gene family was obtained" was removed in the revised manuscript.<br>(3) Each of the single-copy gene cluster consists of a single gene from each species. The construction procedures of the phylogenetic tree have been corrected in the revised manuscript.<br><br>Page 11: "Next, conserved sites were extracted ….." So you only used conserved sites for phylogenetic analysis? Conserved in how many species? All of them? Were these used for phylogenetic analysis? I assume not since if they are conserved (i.e., no variation between species) then they wouldn't tell you anything about how the species are related.<br>Response 28: The “conserved sites” should be better described as alignable sites.<br><br>Page 11: "The phylogenetic tree was constructed …." So, a single model was selected to represent the whole super alignment? Since each single copy gene family used to create the super alignment would likely have a slightly different optimal model, does it make sense to pick just a single model for all of them? Why not perform a partition aware phylogenetic analysis (like what is implemented in the IQtree program)?<br>Response 29: The phylogenetic tree was constructed following procedures described previous studies (Tian et al., 2021; Ran et al., 2019; Song et al., 2021).<br><br>Page 11: "The clustering results of gene families …" Were the gene families filtered in any way before being used for this analysis? You also use the term "orthologous gene families", which you hadn't before in the text, it would help the reader if you picked a single way of referring to the clusters and used it throughout the text.<br>Response 30: A sentence was added in the revised manuscript: “Then the Markov clustering (MCL) algorithm implemented in OrthoMCL was used to group orthologues and paralogues from all input species with an inflation value of 1.5”.<br><br>Page 11: "A probabilistic graphical model …." Implemented in which program? Same with the following sentence, which tool calculated the p-values?<br>Response 31: They were all done by CAFÉ.<br><br>Page 12: "Genome survey analysis using …" Do you mean "genome size estimation using k-mers"?<br>Response 32: Yes. This sentence has been corrected as “The genome size of P. generosa was estimated to be 1.47 Gb using k-mer analysis (Table 1)”.<br><br>Page 12: "included 39 scaffolds anchored to 19 chromosomes with an anchoring rate of 94.70%." I do not quite understand what you mean by this statement. So, there are 39 scaffolds that comprised 19 chromosomes? Or 19 of the scaffolds are the main

	chromosomes and 20 are unplaced scaffolds? Also, I have trouble believing that you only got 39 scaffolds from the assembly workflow. You will always get a lot of small scaffolds; I assume that these were removed at some stage, but I see no discussion of this in the methods. This need to be added so that people know how you generated an assembly with so few scaffolds.<br>Response 33: The P. generosa genome consists of 39 scaffolds whose lengths longer than 20 Kb, among which 19 chromosomes being the main chromosomes and 20 being unplaced scaffolds. We have supplemented the information in the main text.<br><br>Page 14: I could not find Supplementary Table S2 available anywhere, so I was unable to assess this section.<br>Response 34: It is now called Supplementary Table S1 in the revised manuscript.<br><br>Page 14: "and Mytilus edulis have14 chromosomes" add a space between "have 14"<br>Response 35: Corrected.<br><br>Page 14: Which tools were used to create the images in Figure 1? They should be explained in the methods and cited appropriately.<br>Response 36: Detailed methods and references are added in the revised manuscript.<br><br>Table 3: It might be best just to show the column with the combined results and the full table in supplements since you never discuss the other columns in the text. Also, this information is already kind of included as Supplementary table S3, not sure what the differences are between these two tables and if both need to be presented in the manuscript. I recommend consolidating everything into a less redundant format.<br>Response 37: We have integrated previous Table 3 and Supplementary Table S3 to create a new table (Table 3 in the revised manuscript).<br><br>Page 15: This number of predicted genes, number of exons, BUSCO completeness scores, and proportion of repeat genome content is hard to interpret without context of other closely related species. For instance, are these numbers different from what we would expect or are they standard and unremarkable? Without a big supplementary table listing all these stats for all related species it is not possible to really make any conclusions about this genome.<br>Response 38: We have re-analyzed the assembly quality of genomes of all bivalve species with chromosomal-level genome assemblies using BUSCO 5.4.3 and using the lineage-specific dataset of metazoa_odb10 (Supplementary Table S6 in the revised manuscript).<br><br>(1) Also, Supplementary table S4 lists the stats for the "Maker" gene set. Maker was not one of the listed tools used for gene prediction, so I am unsure exactly which gene set was used for this analysis. Also, looking at the data uploaded to the ftp site, only the "Maker" genes were provided. As far as I was aware, the final gene set should have been created using EvidenceModeler not Maker. Additionally, you list the results of functional annotation but don't provide the data anywhere. (2) Please add it to the additional files associated with the manuscript.<br>Response 39:<br>(1) We used “MAKER” to merge the annotation results by the homology-based approach, de novo approach, RNA-Seq-based and Iso-Seq-based evidences to create the final gene set. Thus, the gene set “Maker” is the final gene set used for analysis. The sentence has been corrected as “Gene predictions from the homology-based approach, de novo approach, RNA-Seq-based and Iso-Seq-based evidences were merged, and redundancy was removed to form a comprehensive consensus gene set using Maker 2 (RRID:SCR_005309) [52].”.<br>(2) The results of functional annotation has been added as Supplementary Table S9.<br><br>Page 15: What about the BUSCO scores for the predicted genes. How to they compare? You only list the genome stats in the results section but describe running BUSCO on the predicted genes in the methods.<br>Response 40: We have added BUSCO scores for the predicted genes in the revised manuscript (Table 4).<br><br>Page 16: "P. generosa and other 11 species" should read "P. generosa and 11 other species"

Powered by Editorial Manager® and ProduXion Manager® from Aries Systems Corporation

	Response 41: Corrected.<br><br>Page 16: "30,616 gene families were identified as single-copy and multiple-copy genes, unique paralogs, other orthologs, and unclustered genes" This sentence confused me. What are the definitions for each of these categories? They are not listed in the methods, so I am unable to assess exactly what this all means. Also, the exact numbers of each category should be presented so that we know how many of each were produced by this analysis.<br>Response 42: We showed the details of gene families in various categories for P. generosa and 11 other species in Figure 3 (in the revised manuscript). As per suggestion of the reviewer, we supplemented the table listing the information of all the 30,616 gene families among the 12 species in Supplementary Table S8.<br><br>Page 16: You state that "1749 gene families that are specific to P. generosa", however later in the paragraph you say that "2902 gene families that are specific to P. generosa". I am unsure why these number have changed or what the difference is between the two sets. The latter number is the one that is listed in Figure 3.<br>Response 43: This sentence has been corrected as “As compared with the other 11 species, there were 7917 genes belonging to 1749 gene families that are specific to P. generosa”<br><br>Page 16: (1) The number of gene families in Figure 3 that are common to the 4 species is listed as 6490 but in the text is stated as 6940. Is this a typo? (2) Also, are these gene families specific to just this group and not the other species in your analysis? Or can they be shared with the other species as well? This is not fully clear from the text.<br>Response 44:<br>(1) The number was a typo and has been corrected (the number is “6490”).<br>(2) These gene families are not specific to just this group. The numbers of gene families in Figure 3 (renamed as Figure 4 in the revised manuscript) were based on statistics of P. generosa and 11 other species (Supplementary Table S8). The numbers in statistic (Figure 4) showed the shared and specific gene family numbers among P. generosa, P. martensi, S. broughtonii, and P. yessoensis.<br><br>Page 16: "326 single-copy orthologous gene families from these 12 species showed". You are talking about single-copy orthogroups with genes identified in each species right? Or just a subset? This is not clear from your methods or results sections.<br>Response 45: The “326 single-copy orthologous gene families” represent single-copy orthologous genes shared by all 12 species.<br><br>Page 16: It might be helpful for the reader if you add a shaded box (or another form of annotation) indicating where the bivalve clade is in the tree and how many chromosomes each species has. Since I don't think you explicitly list this for each of your species in the text. ?<br>Response 46: We have added a black bar in the phylogenetic tree to mark the bivalve species (Figure 5 in the revised manuscript).<br><br>Page 18: Supplementary Table S6 does not appear to be available with the manuscript so I was unable to assess this section fully.<br>Response 47: “Supplementary Table S6” was renamed “Supplementary Table S9” in the revised version.<br><br>Page 18: "there were a few significant enrichment pathways" I think should read "there were a few significant enriched pathways"<br>Response 48: Corrected.<br><br>Page 18: "revealed 166 pathways" Pathways or genes? I can't assess what is being tested without the supplementary table.<br>Response 49: The pathway information was included in Supplementary Table S9.<br><br>Page 18: "mainly represented in 5 biochemical metabolic pathways of KEGG category…." Do you mean 5 metabolic KEGG categories? As it is written I am not sure exactly what you are trying to say in this sentence. Also, the high level KEGG categories are very general and not particularly informative, maybe just focus on the subcategories.

Powered by Editorial Manager® and ProduXion Manager® from Aries Systems Corporation

Response 50: This sentence was removed from the manuscript due to adjustment of the content.

Page 18: "Significantly expanded and contracted genes were found to be related to ovarian …." How can these categories that you list be significantly associated with both expanded and contracted gene families? If you are significantly expanded then they are unlikely to also come up as significantly contracted in your analysis?
Response 51: We agree with the reviewer that this sentence should be corrected as "Significantly expanded genes were found to be related to ovarian …". In the revised manuscript, this sentence was removed due to adjustment of the content.

Page 18: "According to enrichment analyses…." I think it should read "According to the enrichment analyses….". Also, I think you should rephrase this section as it is a little unclear which of the categories that you are listing are from your analysis (if any of them are?) and which are from the literature. Currently, you are mostly just listing categories from the literature and I am not really sure how they relate to your analysis or the conclusions that you are trying to make about this particular species.
Response 52: We have rephased this section as shown in the main text.

Page 19: "Two significantly enriched pathwaFcys…." I think additional letters accidentally got added to the word "pathways".
Response 53: This sentence has been corrected as "Two significantly enriched pathways…".

Page 20: "P. generosa is a highly complex species with a high heterozygosity of 1.37% and 57.99% repeat sequences in genome." I think this should be rephrased to something like "P. generosa is a complex species with a genome that has high heterozygosity (1.37%) and repetitive sequence content (57.99%).". Also, is 1.37% heterozygosity high? Compared to what?
Response 54: The heterozygosity of 1.37% is comparable with that of most bivalves (Supplementary Table S3). We revised the statements in the revised manuscript.

Page 19: "This study may provide a" Rephrase the last part of this section to something like "This study provides a high-quality genomic resource that will support future phylogenetic, evolution, adaptation, and immunological studies. Additionally, it will support the genetic breeding of geoducks.".
Response 55: Corrected.

Page 21: I was unable to find any of the ID that you list in the "Data Availability" section on NCBI. I assume they have not yet been made public?
Response 56: The data were released on April 06, 2023 (Although we submitted in November, 2022 with "Release immediately" option).

Page 22: Some of the abbreviations are formatted weirdly. For example, RAxML doesn't have a definition. Neither does Iso-seq. Please double check this section.
Response 57: We have revised the abbreviations in the revised version of the list.

Figure 5: What do the colors in the pie charts represent? The "combined change across lineages" pie chart is hard to interpret as there is no context for the colors or number given. Please add more detail into the figures and figure legends to help the reader interpret the data.
Response 58: The green parts represent gene gains, the red parts represent gene losses and the blue parts represent gene remained. The "combined change across lineages" pie chart is no use, which has been removed from this Figure (as Figure 6 in the revised version). The figure legend has been supplemented with detailed information.

Reviewer #2:
The manuscript reports a chromosome-level genome of the Pacific geoduck Panopea generosa using Pacbio and Hi-C technologies. As an important species in both agriculture and ecology, the Panopea generosa genome is a valuable reference data for the study of clams. The quality of the assemblies seems not bad for the mollusk species. The current genome manuscript is publishable when a substantial revision is

made.

1. The final genome should be described more clearly. It is necessary to display the statistic information of each chromosomes' assemblies, such as length and number of unclosed gaps and contigs.
Response 59: We added chromosome information in Table 2 in the revised manuscript. We also showed statistic information of each chromosomes' assemblies in Supplementary Table S4.

2. Distribution of 17-mer occurrence reflects a high heterozygosity (double-peak distribution) in the genome, which are not suitable for estimation of its genome size (k-mer analysis suitable for the single peak k-mer distribution). Do the authors have any experimental evidence (e.g. flow cytometry analysis) to define the genome size? The author should give more reliable information for it.
Response 60: We did not carry out cytometry analysis in this project. The k-mer analysis was done in this project for estimating the genome size, which was used to determining PacBio sequencing quantity for genome assembly.

3. The authors said that the sequenced individual was collected in Strait of Georgia. How do the authors identify it as the Pacific geoduck P. generosa, mitochondrial sequencing or morphological characterization? It should be clarified.
Response 61: The sample was identified both morphologically and molecularly. The sample showed typical morphological features of P. generosa. This identification was further confirmed by the high percentage identity of the molecular marker cox1 (99.55%) over the entire sequence (coverage = 100%). Details are added in the revised manuscript.

4. The phylogenetic analysis includes human, zebrafish and toad orthologs. The relationship between each of the three vertebrates and P. generosa are so remote that similarity among the homologs are low, which inevitably causes more errors in gene clustering. Besides, the authors use three time points to calibrate the speciation, C. elegans and H. sapiens (678.3-855.2 MYA), D. rerio and H. sapiens (413.1-443.0 MYA) and X. tropicalis and H. sapiens (347.0-357.9 MYA, all of which have the great evolution distance. These time points are not appropriate for the calculation of divergence time among clams. Also, the constructed phylogenetic tree does not have an outgroup. In short, the authors should redo the gene clustering with OrthoMCL by selecting most of the invertebrate species and use the time point among clams or mollusks in fossil calibration. An outgroup should be assigned in the constructed phylogenetic trees.
Response 62: The purpose of this phylogenetic analysis was to illustrate the evolutionary position of P. generosa in metazoan. For the high quality of the phylogenetic tree, we were selective about the genomes used for this analysis. Four model organisms were chosen because they are best studied organisms with high-quality genome assemblies and genome annotations, and they represent good indicators of evolutionary positions. Also, only bivalves with high quality genome assemblies were chosen in this analysis. With the increase of high-quality genome assemblies, more genome will be included in future analysis.

5 The authors carry out the enrichment analysis of the expanded gene families. Actually, it is a tedious work, which do not make sense. Any survey genomes can do that. The chromosome-lever genome is supposed to exhibit more chromosome structure, such as gene collinearity or chromosome synteny among different related species, which is able to reflect not only assembly quality but also data importance. Most of the genome sequencing and assembling are completed by a commercial sequencing company, which means that the manuscript should include more analyzed information by the authors themselves. Referencing the recently-published papers in Gigascience, the manuscript should normally make one or more tries of evolutionary analyzation or gene expression profiling, not just the description of the assembly itself. Accordingly, the authors, on the one hand, should improve this description by providing a couple of figures to illustrate the annotation of the expanded gene families; one the other hand, construct the gene/chromosome synteny among 3 or more related species (illustrated as the circus image) to check the chromosome arrangement of evolutionary analysis.
Response 63: We appreciate the suggestions. We have carried out additional synteny

	analysis and gene family analysis. In particular, through comparative analysis of the chromosome-level genome assembly of P. generosa and its closely related species S. constricta, we uncovered major inter-chromosome changes. We also observed substantial intra-chromosomal changes. A new figure (Figure 2) is included in the revised manuscript to describe these new results. We have also included the identification of expanded gene families with functional domains in P. generosa. In particular, the copine gene family showed substantial expansion in P. generosa, with twice as many genes in this gene family compared to its closely related species. A new figure (Figure 7) is included in the revised manuscript.<br><br>6. In main text, the BUSCO test indicates the genome completeness of 90.9%. However, in the supplementary files, the Hi-C BUSCO and annotation BUSCO test show the completeness at 93% and 88%, respectively. The authors should clarify it and provide the BUSCO test table in the manuscript.<br>Response 64: As the genome as evaluated multiple times using different BUSCO version, results were inconsistent in the original submission. BUSCO results have been revised in the revised manuscript.<br><br>7. The English writing of the manuscript should be improved when a revision is submitted.<br>Response 65: We have done extensive revision of the writing of the manuscript.
**Additional Information:**	
**Question**	**Response**
Are you submitting this manuscript to a special series or article collection?	No
**Experimental design and statistics**<br><br>Full details of the experimental design and statistical methods used should be given in the Methods section, as detailed in our Minimum Standards Reporting Checklist. Information essential to interpreting the data presented should be made available in the figure legends.<br><br>Have you included all the information requested in your manuscript?	Yes
**Resources**<br><br>A description of all resources used, including antibodies, cell lines, animals and software tools, with enough information to allow them to be uniquely identified, should be included in the Methods section. Authors are strongly encouraged to cite Research Resource Identifiers (RRIDs) for antibodies, model organisms and tools, where possible.	Yes

Powered by Editorial Manager® and ProduXion Manager® from Aries Systems Corporation

Have you included the information requested as detailed in our Minimum Standards Reporting Checklist?	
**Availability of data and materials**<br><br>All datasets and code on which the conclusions of the paper rely must be either included in your submission or deposited in publicly available repositories (where available and ethically appropriate), referencing such data using a unique identifier in the references and in the “Availability of Data and Materials” section of your manuscript.<br><br>Have you have met the above requirement as detailed in our Minimum Standards Reporting Checklist?	Yes

This manuscript file contains the revised main text, Tables, Figure legends with tracks of revisions. It presents how we

Click here to access/download;Manuscript;Manuscript_with_tracks_of_revisi

Chromosome-level genome assembly of the Pacific geoduck *Panopea generosa* reveals major inter- and intra-chromosomal rearrangements and substantial expansion of the copine gene family

Jing Wang[1,2,3], Qing Xu[1,2,3], Min Chen[4], Yang Chen[1,2,3], Chunde Wang[4,5*], Nansheng Chen[1,2,3,6*]

[1]CAS Key Laboratory of Marine Ecology and Environmental Sciences, Institute of Oceanology, Chinese Academy of Sciences, Qingdao, China

[2]Laboratory of Marine Ecology and Environmental Science, Qingdao National Laboratory for Marine Science and Technology, Qingdao, China

[3]Center for Ocean Mega-Science, Chinese Academy of Sciences, Qingdao, China

[4]Yantai Institute of Coastal Zone Research and Center for Ocean Mega-Science, Chinese Academy of Sciences, Yantai, China

[5]Marine Science and Engineering College, Qingdao Agricultural University, Qingdao, China

[6]Department of Molecular Biology and Biochemistry, Simon Fraser University, Burnaby, BC, Canada

Jing Wang Email: wangjing2019@qdio.ac.cn; Qing Xu Email: xuqing_77@163.com; Min Chen Email: mchen@yic.ac.cn; Yang Chen Email: cy4043@hevttc.edu.cn.

*Correspondence address. Chunde Wang, Yantai Institute of Coastal Zone Research and Center for Ocean Mega-Science, Chinese Academy of Sciences, Yantai, China. E-mail: chundewang2007@163.com; Nansheng Chen, CAS Key Laboratory of Marine Ecology and Environmental Sciences, Institute of Oceanology, Chinese Academy of Sciences, Qingdao, China. Email: chenn@qdio.ac.cn

Formatted: Justified, No widow/orphan control

Abstract

The Pacific geoduck *Panopea generosa* (class BivalveBivalvia, order Adapedonta, family Hiatellidae, genus *Panopea*) is the largest known burrowing bivalve with considerable commercial value. Pacific geoduck and other geoduck clams play important roles in maintaining ecosystem health for their filter feeding habit and coupling pelagic and benthic processes. Chromosome-level genomes of geoduck clams will contribute to genetic breeding, as well as ecosystem and climate change biology. Here, we report the first high-quality chromosome-scale chromosome-level genome assembly of *P. generosa* with the purpose to unravel its phylogenetic characteristics and molecular mechanisms of its life strategies, and promote research on genetic breeding. The assembled *P. generosa* genome consists of 19 chromosomes with a size of was 1.475 Gb in size, with a contig N50 of 1.6 Mb and a scaffold N50 of 73.8 Mb. The assembly contained 39 scaffolds anchored to 19 chromosomes with an anchoring rate of 94.7%. BUSCO analysis showed 90.993.0% completeness in the 889 eukaryote core conserved genes in the assembled *P. generosa* genome. Comparative analysis of the genomes of two closely related species in the order Adapedonta, *P. generosa* and *Sinonovacula constricta*, revealed major inter- and intra-chromosomal exchanges. Of the 35,034 predicted protein-coding genes (PCGs), 30,700 genes (87.63%) were functionally annotated. Comparative analysis of the genomes of *P. generosa* and 11 related species identified 507 expanded gene families and 875 contracted gene families in *P. generosa*. Enrichment analysis revealed significant expansion of immune and gonad development gene families that may promote its complex survival strategies. In particular, the copine gene family, which plays an important role in

calcium signaling, membrane trafficking, and cytoskeletal dynamics, and has been implicated in several physiological and pathological processes such as neuronal development, immune response substantially expanded in *P. generosa* with 22 members annotated. The availability of a chromosome-level ~~the~~ *P. generosa* genome assembly and its annotated gene set ~~may~~ provide a useful molecular platform for research~~es~~ on its genetic breeding.

Keywords: *Panopea generosa*, chromosome-level genome assembly, genetic breeding, evolutionary adaptation

~~Context~~Introduction

The Pacific geoduck *Panopea generosa* is one member of genus *Panopea* which includes the world's largest burrowing bivalves. ~~*Panopea*~~ *P. generosa* is usually found in low intertidal and subtidal sediments throughout the northeast Pacific coast, including the United States (Alaska, Washington, and California), Canada (British Columbia), and Mexico (north Baja Pacific Coast) [1, 2]. Geoducks can reach more than 25 cm in shell length, and more than 100 cm in siphon length [3]. Geoduck adults are usually buried in muddy-sandy sediment at depths ranging 60–100 cm, with only their siphon tips exposed to respire, capture food, and release secretion/excretion products and gametes. The sedentary behavior may contribute to their long life spans (which can be as long as 168 years) for *P. generosa* [4]. Due to these unique life strategies, it is expected that geoduck should have distinctive growth and development mechanisms, especially in relation to benthic life and immune system.

Geoduck clams play important roles in maintaining ecosystem health for their filter feeding habit and coupling pelagic and benthic processes by ejecting undigested mucus-bound feces and pseudo feces to the sediment surface. They are prey~~s~~ for sea otters, fishes, crabs, and sea stars [5, 6]. As marine calcifiers, shell concentrations of *Panopea* inside Scalichnus burrows have been analyzed to reconstruct the sequence of events related to storm events [7]. Geoduck clams possess great commercial fishery value in Canada and the USA [8]. Since the recruitment of geoducks have been low [9] due to overfishing and their vulnerability to environmental changes [10], there has been an increasing interest~~s~~ in genetic breeding of geoducks.

The assembled genome sizes of bivalves vary widely, ranging from 543.9 Mb in *Lutraria thynchaena* [11] to 2.6 Gb in *Modiolus philippinarum* [12] (Supplementary Table S1). Among bivalves, the genome sizes of most superorder Imparidentia species ranged from 1 Gb to 1.8 Gb, and that of the species in the order Adapedonta, which includes *P. generosa*, ranged from 1 Gb to 1.5 Gb [13].

The numbers of chromosomes also vary substantially among bivalves, suggesting active genome recombination in evolution in bivalves [14]. While the species of the order Ostreida, including *Crassostrea gigas* [15], *Crassostrea virginica*, *Crassostrea hongkongensis* [16], *Crassostrea ariakensis* [17], Crassostrea angulate, and Ostrea edulis, have 10 chromosomes, the species of the order Ostreida, including *Pinctada fucata* [18], *Pinctada imbricata* and the species of the order Mytilida including *Mytilus coruscus* [19] and *Mytilus edulis* have 14 chromosomes. The chromosome numbers of the order Cardiida varied from 17-19 [20]. Interestingly, the numbers of chromosomes of species in most other orders including Venerida, Arcida, and Pectinida, are 19, except for *Corbicula fluminea* [21] possessing 18 chromosomes and *Argopecten* scallop possessing 16 chromosomes. The reported numbers of chromosomes of species in the order Adapedonta, which includes *P. generosa*, are also 19.

Nevertheless, high-quality ~~chromosome-scale~~ chromosome-level reference genome of *P. generosa* is currently not available, hindering the development of geoduck genetic breeding programs. In this study, we report the first chromosome-scale genome assembly for *P. generosa* generated using~~by combining~~ cutting-edge technologies including next-generation sequencing~~ (BGIseq)~~, ~~PacBio Sequel~~ long read sequencing, and high-

throughput chromosome conformation capture (Hi-C) technologies. We further performed gene family clustering, phylogenetic analysis, and gene family expansion and contraction, in order to understand its adaptation, growth, development and immunity. The availability of the genome information will facilitate further researches in molecular evolution and genetic breeding.

Results

Genome sequencing and assembly

Genome survey analysis using BGIseq reads estimated the genome size of *P. generosa* as 1.48 Gb The genome size of *P. generosa* was estimated to be 1.48 Gb using *k*-mer analysis (Table 1) [22]. The heterozygosity and repeated sequence content were estimated to be 1.37% and 57.99% (Supplementary Figure S1 and Table S2), respectively. The heterozygosity of *P. generosa* was comparable to those of most bivalves (Supplementary Table S3). Genome assembly using PacBio long reads (N50 = 26,513 bp) and Falcon assembler obtained an initial size of 1.51 Gb genome. Further assembly using Hi-C data obtained a genome with 19 pseudomolecules, suggesting 19 chromosomes of the *P. generosa* genome (Figure 1A), with an anchoring rate of 94.70%. This genome assembly, which has a total length of 1,474,161,289 bp with a contig N50 of 1.57 Mb and a scaffold N50 of 73.79 Mb (Figure 1B; Table 2; Supplementary Table S4), included 39 scaffolds anchored to 19 chromosomes with an anchoring rate of 94.70%. As expected, the genomic regions with low gene density typically had high repeat content, while the regions with high repeat content usually had high GC content.

The assembled genome size of *P. generosa* (1.47 Gb) fell in the range of the reported

genomes of bivalves species reported, which varied from 543.9 Mb in *Lutraria thynchaena* [11] to 2.6 Gb in *Modiolus philippinarum* [12] (Supplementary Table S2S1). Among bivalve species, the genome sizes of most superorder Imparidentia species ranged from 1 Gb to 1.8 Gb, and that of the species in order Adapedonta ranged from 1 Gb to 1.5 Gb.

The numbers of chromosomes varied substantially among bivalve species. While the species of the order Ostreida, including *Crassostrea gigas* [50], *Crassostrea virginica*, *Crassostrea hongkongensis* [51], *Crassostrea ariakensis* [52], have 10 chromosomes, and the species of the order Ostreida, including *Pinctada fucata* [53] and the species of order Mytilida including *Mytilus coruscus* [54] and *Mytilus edulis* have14 chromosomes. The Myida order species *Dreissena polymorpha* and all *Argopecten* scallop have 16 chromosomes [55]. In contrast, the numbers of chromosomes of species in most other orders including Adapedonta, Venerida, Cardiida, Arcida, and Pectinida, are 19, except for *Corbicula fluminea* [56], which has 18 chromosomes.

Genome annotation and evaluation

The majority (57.99%) of the *P. generosa* genome was repetitive elements estimated using *de novo* searching and homolog prediction (Supplementary Table S3). Distribution of these repetitive elements was uneven with repetitive content per 1 Mb varied from 34.76% to 84.89% (Figure 1B). DNA transposons (21.6%), long interspersed nuclear elements (LINEs, 9.1%) and long terminal repeats (LTRs, 3.76%) were the top three categories of repetitive elements in the *P. generosa* genome (Table 3).

A total number of 35,034 protein-coding genesPCGs were annotated in the *P.*

genererosa genome. The mean number of exons per gene was 5.78 (Supplementary Table S4). Of these PCGsprotein-coding genes, 30,700 genes were annotated to contain conserved functional motifs (Supplementary Table S5, Table S7).

To evaluate the completeness of the assembly, the assembled *P. generosa* genome assembly and annotated protein-coding gene set waswere assessed using BUSCO [23] with the metazoan metazoa_odb109 database (978 954 core genes). , respectively. For the genome assembly, We we found that 90.993.0% of core genes were identified as full-length in the *P. generosa* genome assembly and 43 (4.4%) core genes were captured as fragments (Table 4), suggesting that the genome assembly was of high quality. , compared to 91.5% of the closely related species *S. constricta* [13] (Supplementary Table S6). Regarding the gene set, 88.4% of core genes were identified as full-length, suggesting that the quality of the gene set could be further improved.

Chromosomal synteny analysis between *P. generosa* and *S. constricta*

Although the *P. generosa* genome has 19 chromosomes as many other species in the order Adapedota, chromosomal synteny between *P. generosa* and other species in this order remains unknown. Comparative analysis between the genomes of *P. generosa* and *S. constricta*, whose genome has been assembled at the chromosome-level revealed that these two genomes have good chromosomal collinearity in general (Figure 2A). One-to-one chromosomal correspondences between *P. generosa* and *S. constricta* were obvious. Indeed, 17 of 19 *P. generosa* chromosomes showed clear one-to-one r correspondences *S. constricta* chromosomes (Figure 2A). However, major inter-chromosomal exchanges

were evident. For example, *P. generosa Pg02* matched to two *S. constricta* chromosomes (*Chr1* and *Chr10*). Similarly, *P. generosa Pg11* matched well to two *S. constricta* chromosomes (*Chr1* and *Chr10*).

In addition to these major inter-chromosomal exchange events, comparative analysis of these two genomes also revealed that extensive intra-chromosomal recombination events, which resulted in little co-linearity within chromosomes. Instead of a clear diagonal linear relationship between genes of these two species *P. generosa* and *S. constricta*, a near random scattering of the relationships were observed (Figure 2A). These intra-chromosomal recombination events were also clearly shown in Figure 2B.

Comparative analysis of gene families and evolutionary analysis

Using the protein-coding genes of *P. generosa* and other 11 species (*P. martensi*, *C. gigas*, *B. platifrons*, *P. yessoensis*, *P. maximus*, *A. purpuratus*, *S. groughtonii*, *H. sapiens*, *X. tropicaalis*, *D. rerio*, and *C. elegans*) (Table 5), In total, 30,616 gene families were identified among *P. generosa* and 11 other species (*Pinctada martensi*, *C. gigas*, *Bathymodiolus platifrons*, *Patinopecten yessoensis*, *Pecten maximus*, *Argopecten purpuratus*, *Scapharca broughtonii*, *Homo sapiens*, *Xenopus tropicaalis, Danio rerio, and Caenorhabditis elegans*) as single-copy and multiple-copy genes, unique paralogs, other orthologs, and unclustered genes, as well as 326 single-copy orthologous gene families (Table 5, Figure 23, Supplementary Table S8). As compared with the other 11 species, there were The analysis identified 7917 genes belonging to 1749 gene families that are specific to *P. generosa*. Comparative analysis of the genes of *P. martensi*, *S. broughtonii*, *P. yessoensis*, and *P. generosa* revealed 69406490 common gene families shared by that

~~are common to~~ these ~~animals~~species and 2902 gene families ~~that are~~ specific to *P. generosa* (Figure ~~3~~4). Phylogenetic analysis using 326 single-copy orthologous gene families from these 12 species showed that *P. generosa* was tightly clustered with other bivalves~~ species~~ as expected (Figure ~~4~~5). According to the phylogenetic tree, the divergence time of *P. generosa* from its nearest node was approximately 491.5 Mya (Figure ~~4~~5). In addition, the divergence time of *P. generosa* is earlier than other bivalves~~ species~~, which is consistent with that of *S. constricta* [13], a species close to *P. generosa*. *B. platifrons*, *C. gigas* and *P. martensi*, which have 10 or 14 chromosomes, were clustered as a single clade and diverged from other bivalves with 19 chromosomes in the phylogenetic tree.

~~**Gene family expansion and contraction in *P. generosa***~~

A total of 507 expanded gene families (involving 2,734 genes) and 875 contracted gene families (involving 792 genes) were identified in *P. generosa* genome compared to the most recent common ancestor of both *P. generosa* and the other 11 species (Figure ~~5~~6). KEGG analysis revealed 166 pathways from the expanded gene families ~~and 123 pathways from the contracted gene families~~ were ~~significantly~~ enriched ~~(Qvalue ≤ 0.05)~~ with various biological processes (Supplementary Table S~~9~~6)~~, suggesting their important contribution to the adaptation of benthic bivalves.~~ The enrichment analysis suggested that the significantly expanded genes of *P. generosa* were mainly represented in ~~5 biochemical metabolic pathways of KEGG category, including~~ organismal systems, human diseases, ~~metabolism,~~ and environmental information processing, such as phototransduction, Fluid

shear stress and atherosclerosis, Phosphatidylinositol signaling system, suggesting their important contribution to the adaptation of benthic bivalves.and cellular processes. KEGG subcategories included signal transduction and endocrine system, which were ranked as the most enriched pathways, followed by infectious diseases (Bacterial), amino acid metabolism, lipid metabolism, digestive and immune system.

Significantly expanded and contracted genes were found to be related to ovarian development, immune, osmoregulation, and pigmentation (Figur 5, and Supplementary Table S6), which may be important for the adaptation of benthic *P. generosa*. According to enrichment analysesMeanwhile, according to the enriched KEGG pathways of expanded gene families in *P. generosa* (Supplementary Table S9), there were a few significant enrichedment pathways (Qvalue < 0.05) related to gonad development. For example, adrenergic signaling in cardiomyocytes, and glycine, serine and threonine metabolism which has beenwere found as enriched pathways related to spermatogenesis of the fluted giant clam *Tridacna squamosa* [24]. Moreover, oocyte meiosis, apoptosis, Ras signaling pathway, calcium signaling pathway, steroid hormone biosynthesis, GnRH signaling pathway, insulin signaling pathway, oxytocin signaling pathway, and ovarian steroidogenesis have beenwere documented to be enriched in *Procambarus clarkii* ovary development [25]. Geoducks have become a focus of significant aquaculture research and development with a considerable commercial value [26, 27]. The enriched gonad development-related pathways and genes could provide basic data for the further genetic breeding research of *P. generosa* and its closely related species.

We further compared gene families in different bivalves by searching for functional

domains contained in PCGs in *P. generosa* and 8 other bivalves using InterProScan [28]. Examination of the top 65 domains of gene families that presented expansions in *P. generosa* (Figure 7A) showed that the gene numbers of many important gene families were substantially expanded in *P. generosa*, including these containing the GIY-YIG catalytic domain (PF01541), the caspase recruitment domain (PF16739), the ApoA/ApoE domain (PF01442), and the copine domain (PF07002). In particular, the copine gene family, which has been implicated in a range of cell signaling and cytoskeletal proteins, targeted to the membrane following increases in cellular calcium, has twice as many genes compared to other species. In *P. generosa*, the number of genes of the copine gene family was 22, comparing to 11 copine genes in *S. constricta*. The 22 copine genes in *P. generosa* were found in multiple chromosomes including *Pg02*, *Pg05*, *Pg07*, *Pg10*, *Pg11* and *Pg17* (Figure 7B). Interestingly, many genes formed local clusters, suggesting that the large copine gene set observed in *P. generosa* might have been achieved via tandem duplication of the copine genes in evolution. For example, eight genes were located in a single cluster in *Pg11* (Figure 7B).

Phylogenetic analysis of the copine genes annotated in *P. generosa* (22 genes) and *S. constricta* (11 genes) revealed good orthologous relationships, as well as one-to-multiple relationships (Figure 7C), confirming that genes inside copine gene clusters in *P. generosa* (Figure 7B) were highly similar.

Most of the 22 copine genes in *P. generosa* contain the vWA-domain, while some has both C2-domain and the vWA-domain (Figure 7D) as for copine genes identified in other species [29]. Some copine genes were short, which might be due to imperfect annotation

of these genes. Prediction of 3D structures of these candidate copine genes in *P. generosa* using AlphaFold2 [30] revealed that their structures are highly similar to those copine genes identified in other species. For example, the structure of *Pg02g00048* in *P. generosa* showed high similarity to that of *XP_0533888641* in *Mercenaria mercenaria* (Figure 7E).

Meanwhile, many enrichment pathways (Qvalue < 0.05) related to immune have been significantly enriched and appeared to be complex. Toll-like receptor signaling pathway, natural killer cell mediated cytotoxicity, antigen processing and presentation, B cell receptor signaling pathway, Fc epsilon RI signaling pathway, Fc gamma R-mediated phagocytosis, leukocyte transendothelial migration, and chemokine signaling pathway have been found in the immune pathways of the clam *Saxidomus prupuratus* transcriptome [62]. Toll-like receptor signaling pathway and apoptosis have also been reported in shell mussel *Mytilus coruscus* in response to *Vibrio alginnolyficus* infection. Immune-related pathways antigen processing and presentation, lysosome, phagosome, and PI3K-Akt signaling pathway have been proved to be the pathways of differentially expressed genes of *Chamys farreri* in response to tetrabromobisphenol A stress [63]. The other immune pathways of peroxisome, Rap1 signaling pathway, Ras signaling pathway, cGMP-PKG signaling pathway, cAMP signaling pathway, cell adhesion molecules (CAMs), Bacterial invasion of epithelial cells, and inflammatory mediator regulation of TRP channels have also been found in the enriched pathways of *Procambarus clarkii* [59]. Two significantly enriched pathwaFcys, phagosome and apoptosis have been found to be shared between *S. constricta* and *P. generosa* [57]. In contrast, other pathways such as the NOD-like receptor

signaling pathway, focal adhesion, NF-kappa B signaling pathway, tumor necrosis factor (TNF) signaling pathway, and endocytosis enriched in *S. constricta* were not significantly enriched in *P. generosa*. These differences may reflect their different life strategies in adaptation to different survival depth below the substratum surface and pathogenic stresses.

DiscussionConclusions

Through the construction of the first In summary, a high-quality chromosome-level genome assembly of the ecologically and economically important bivalves the Pacific geoduck *P. generosa* with cutting-edge genomic technologies, important insights into its genetic makeup and evolution have been gained. was obtained in this study. The assembled genome size consists of 19 chromosomes with a genome size ofwas 1.45 1.47 Gb, andwith a contig N50 of 1.6 Mb and 19 chromosomes. A total of 35,034 protein-coding genesPCGs were predicted in the *P. generosa* genome, of which 30,700 genes (87.63%) were functionally annotated, supporting the high-quality annotation of the *P. generosa* genes, and the conservation of PCGs in bivalves. *P. generosa* is a highly complex species with a high heterozygosity of 1.37% and 57.99% repeat sequences in genome.

The availability of the first high-quality chromosome-level *P. generosa* genome assembly enabled us to ascertain its genomic compositions, which in turn facilitate comparative analysis with other bivalve genomes. The *P. generosa* genome represents the third genome of the third species in the order Adapedonta, after *S. constricta* and *Solen grandis*. Although all the three genomes have the exactly same number of 19

chromosomes, comparative analysis between the genomes of *P. generosa* and its most closely related species *S. constricta* revealed extensive inter- and intra-chromosomal exchanges. Large chromosomal fragments of *P. generosa Pg02* and *Pg11* matched to two *S. constricta* chromosomes (*Chr1* and *Chr10*) and two *S. constricta* chromosomes (*Chr1* and *Chr10*), respectively (Figure 2). It is rather surprising that such large inter-chromosomal exchanges did not lead to the change of chromosomal numbers of either *P. generosa* or *S. constricta*. Within chromosomes, the order of genes showed even more extensive alterations (Figure 2).

In addition to the changes of organization of chromosomes and order of genes in the *P. generosa* and its closely related species *S. constricta*, the numbers of PCG members in gene families with important functions also changes substantially (Figure 7). In particular, 22 copine genes were identified in *P. generosa*, representing twice those in *P. constricta*. Many of these 22 copine genes in *P. generosa* formed tandem clusters, with one cluster containing eight copine genes (Figure 7B). The copine genes within each cluster showed high similarity (Figure 7C), further confirming tandem duplications. Copine genes in *P. generosa* contain C2 domain and vWA-domain (Figure 7D). Modeling with AlphaFold2 revealed that copines encoded by the *P. generosa* copine genes show high structural similarity with that encoded by known copine genes (Figure 7E). Thus, comparative analysis of gene families uncovered important leads for exploring molecular insight into its physiology and evolution.

Taken together,~~This~~ this study ~~may~~ provides ~~a~~ the first high-quality genomic resource ~~for~~ that will support future ~~studies of the~~ phylogenetic~~ characteristics~~, evolution~~ and~~

adaptation, and immunologicaly and may other related studies. Most importantly, the assembly of Additionally, *P. generosa* genome may greatly facilitate it will support the genetic breeding of geoducks.

Methods

Sampling collection

Geoducks *P. generosa* samples were collected from the Strait of Georgia (49°41′12″N, 124°51′33″W) of British Columbia, Canada in the spring of 2019. The samples showed typical morphological features of *P. generosa*. The identification of the samples was also supported by the high similarities of the molecular marker *cox1* to the reference sequence of *P. generosa* (PID of 99.55, coverage of 100%) [31]. The samplesanimals- were then transferred to the laboratory and kept in a tank with running water for a week. One sampleanimal was chosen and dissected on ice to collect tissue samples, including labial palp, heart, foot, gonad, gill, hepatopancreas, siphon, and mantle muscle. This animal was identified to be a female as indicated by the presence of eggs in the smear of the gonad under a compound microscope. Dissected tissues were quickly frozen in liquid nitrogen and then stored at −80°C before DNA and RNA extraction.

DNA library construction and sequencing

Genomic DNA of *P. generosa* was extracted using a standard phenol-chloroform extraction method [32]. The quality of DNA was determined by gel electrophoresis to ensure the DNA samples met library sequencing requirements. Sequence libraries with insert size of 300 bp were constructed for BGISEQ-500 sequencing platform (RRID:SCR_017979) according to manufacturer's protocol.for the sequence platform

BGIseq-500 (RRID:SCR_017979), which The sequencing data produced were used in the genome size estimation, by *k*-mer analysis [22] and for correcting errors in the Pilon (RRID:SCR_014731) assembly [33]. A Hi-C library with insert size of 300 bp was constructed to provide long-range information (without position information) on the grouping and linear organization of sequences along entire chromosomes to assemble the scaffolds into chromosome-level scaffolds [34]. For Hi-C library construction, gonad tissue was dissociated, and cells were collected and crosslinked with 1% formaldehyde (Sigma) and 0.2M glycine (Sigma). After that, the fixed powder was resuspended in nuclei isolation buffer and then incubated in 0.5% SDS for 10 min at 62°C. Then the reaction was quenched with 10% Triton X-100 (Sigma) and the nuclei were collected by centrifugation. Then the DNA was digested with MboI (NEB), and the overhang was filled and biotinylated before ligated by T4 DNA ligase (NEB). Before library construction, the purified DNA was sheared, and biotin-containing fragments were captured on streptavidin-coated beads using Dynabeads MyOne Streptavidin T1 (Invitrogen). The fragments were then end-repaired and linked with adaptors before eight cycles of PCR reaction with KAPA HiFi HotStart ReadyMix (Kapa Biosystem). After that, the Hi-C library was sequenced with BGISEQ-500 platform. And a PacBio library with insert size of 20 Kb was constructed to obtain long reads by the PacBio Sequel platform using the Sequel Sequencing Kit 3.0. The adapters and low-quality reads in raw sequence data generated by the BGISEQseq platform were filtered by the following criteria: reads with adapters, reads with N bases more than 1% and reads with low-quality bases (Q≤5) more than 10%. were cut off by SOAPnuke1.5.6 using the parameter as "-n 0.01 -l 20 -q 0.1 -i -Q 2 -G -M 2 -A 0.5 -d" [35].

Subreads of PacBio data were filtered by the default parameters.PacBio raw data were filtered with the default parameters by using Pacific Biosciences SMRT analysis software (v2.3.1) to filter the low quality reads.

RNA library construction and sequencing

RNA-seq Seq and Iso-seq Seq were conducted to obtain transcriptome data to aid genome annotation. The total RNAs was extracted by Trizol (Invitrogen, Carlsbad, CA, USA) from eight tissues of the same *P. generosa* individual, including labial palp, heart, foot, gonad, gill, hepatopancreas, siphon, and mantle muscle. Each RNA sample was qualified and quantified The quality and quantity of RNA in each sample was assessed using a NanoDrop and an Agilent 2100 bioanalyzer (Thermo Fisher Scientific, MA, USA). For The construction of mRNA libraries for RNA-seqSeq, the mRNA library was constructed by purification, fragmentation, a first-strand cDNA generation, a second-strand cDNA synthesis, and RNA index addition. the mRNA was enriched by mRNA Capture Beads (BGI, LB00V60), and incubated at 85°C for 8 minutes for fragmentation. Reverse transcription was performed with Strand Specificity Reagent and 1st Strand Enzyme Mix (Optimal Dual-mode mRNA Library Prep Kit, BGI, LR00R96) to generate the first strand cDNA. After that the second strand cDNA generation and end repair were performed with 2nd Strand Buffer and 2nd Strand Enzyme Master Mix. Then the adaptors (BGI, LA00R04) were ligated to the cDNAs. Then the library was purified and selected depending upon product requirements for amplification. The eight mRNA libraries were sequenced using the BGISEQseq-500 platform. For Iso-Seq, the total RNA was extracted from the equally mixed tissues of the 8 tissues above. The PacBio SMRTbell library was prepared using the

SMARTer PCR cDNA Synthesis kit (Clontech), the Qubit dsDNA HS Assay Kit 2.0 (Invitrogen) and the Agilent DNA 12000 kit (Agilent Technologies), and sequenced by the PacBio Sequel sequencer (RRID:SCR_017989) with Sequel Sequencing Kit 3.0.

Genome size estimation and genome assembly

Genome size of *P. generosa* was estimated using *k*-mer analysis. Counting of *k*-mers was conducted using Jellyfish (RRID:SCR_005491, version 2.2.10) [22]. For genome assembly, long reads generated from PacBio Sequel platform were assembled using Falcon (RRID:SCR_016089) [36], which was subsequently polished using Arrow. Short paired-end clean reads from BGIseqBGISEQ-500 were then polished using used for correcting post-processing errors and resolving conflicts of assembly via Pilon (RRID:SCR_014731, version 1.22) [33]. Chromosomes were then assembled using Hi-C data filtered by SOAPnuke (SOAPnuke, RRID:SCR_015025) and mapped by HiC-Pro (HiC-Pro, RRID:SCR_017643) [15]. The assembled contigs were corrected for mis-joins, orders, orients and anchored contigs from the draft assembly into a candidate chromosome-length assembly by Hi-C data using Juicer (RRID:SCR_017226) [37] and 3d-DNA [38]. The scaffolds shorter than 20 Kb were removed. Chromosomes were further corrected using Juicebox (Juicebox, RRID:SCR_021172) [16]. Finally, the candidate assembly were reviewed with Juicerbox Assembly Tools (RRID:SCR_021172) for quality control and interactive corrections [39]. The Hi-C heatmap was visualized using Juicebox (RRID:SCR_021172) presenting the counts of paired reads which each two bins aligned (with the bin length of 100 kb) as the interactive signals between each pair of two bins. The completeness of genome assembly was assessed using by BUSCO (RRID:SCR_015008,

Formatted: Font: Italic

Formatted: Font: Italic

version~~ 3.0.2~~5.4.3) [23]. using the metazoa_odb10 database. The genome landscape illustrating the length, repeat element density, gene density and GC content was created by circos-0.69-9 (RRID:SCR_011798) [40].

Annotations of gene structure and function

Homologous and *de novo* predictions were both applied to annotate ~~repetitive sequences~~ transposable elements in the *P. generosa* genome. ~~In homologous prediction, RepeatMasker (RepeatMasker, RRID:SCR_012954) and the associated RepeatProteinMask [18] were performed by alignment against Repbase database (Repbase, RRID:SCR_021169) [19].~~ In homologous prediction, RepeatMasker (RRID:SCR_012954) and RepeatProteinMask [41] were used to screen the *P. generosa* genome for known transposable elements in the RepBase library (RRID:SCR_021169) [42]. In *de novo* prediction, RepeatModeler (version 1.0.4) was first used for de novo candidate database construction of repetitive elements, and repetitive sequences were then annotated ~~Piler [20], RepeatScout [21] and RepeatModeler (version 1.0.4) [22] were used for de novo candidate database construction of repetitive elements, and repetitive sequences were then annotated~~ using RepeatMasker. ~~Besides, t~~Tandem repeats were de novo predicted using Tandem repeats finder (version 4.07) [43]. The results were then integrated and ~~redundancy was~~duplicates were eliminated.

Three complementary approaches were adopted to predict ~~protein-coding genes~~ PCGs in *P. generosa* genome, including homology-based prediction, ~~*De*~~ *de novo* annotation, and transcriptome-based prediction. For homology-based prediction, gene

sets from eight closely related bivalves species (*Patinopecten P. yessoensis, Pinctada P. fucata, Mytilus galloprovincialis, Limnoperna fortune, Argopecten A. purpuratus, Sinonovacula S. constricta, Scapharca S. broughtonii, and Crassostrea C. gigas*) were usedfrom different branches were chosen to confirm the completeness of the gene set. First, protein repertoires of those organisms were aligned against the *P. generosa* genome using TBLASTN (RRID:SCR_011822) [44]. The Basic Local Alignment Search Tool (blast) hits were then conjoined by Solar software (SOLAR, RRID:SCR_000850) [25]. Then gene structures were predicted from these blast hits by Exonerate v2.2.0 [45]. Next, GeneWise (GeneWise, RRID:SCR_015054) [26] was used to predict the exact gene structure of the corresponding genomic region on each blast hit. Notably, homology predictions were denoted as "Homology-set". For *de novo* annotation, six programs were simultaneously used, including Augustus (Augustus, RRID:SCR_008417) [27], GeneID (GeneID, RRID:SCR_002473) [28], GeneScan (GENSCAN, RRID:SCR_012902) [29], GlimmerHMM (GlimmerHMM, RRID:SCR_002654) [30], FgeneSH [31] and snap (SNAP, RRID:SCR_007936) [32]. *de novo* gene prediction was performed using a combination of Augustus (RRID:SCR_008417) [46] and SNAP (RRID:SCR_007936) [47] with default settings. Among them, Augustus, snap and GlimmerHMM were trained using PASA-T-set gene models. For transcriptome-based prediction using RNA-Seq data, RNA-Seq reads were directly mapped to the genome using TopHat2 (TopHat, RRID:SCR_013035) [48]. The mapped reads were subsequently assembled into gene models (Cufflinks-set) by Cufflinks (Cufflinks, RRID:SCR_014597) [49]. For transcriptome-based prediction based on Iso-Seq data, Iso-Seq reads were directly mapped to the genome using GmapGMAP

(GMAP, RRID:SCR_008992) [50]. The mapped reads were subsequently assembled by PasaPASA (PASA, RRID:SCR_014656) [51]. Gene predictions from the homology-based approach, *de novo* approach, RNA-Seq-based and Iso-Seq-based evidences were merged, and redundancy was removed to form a comprehensive consensus gene set using EvidenceModeler (EVidenceModeler, RRID: SCR_014659) [37] Maker 2 (RRID:SCR_005309) [52]. To validate the completeness of the gene structure annotation, we also used BUSCO (version 3.0.25.4.3) with the metazoa_odb10 database [23].

Phylogenetic analysis and divergence time estimation

Gene families were constructed according to using the OrthoMCL (RRID:SCR_007839) pipeline [53]. We selected The protein-coding genes of *P. generosa* and other 11 species (*C. gigas*, *P. yessoensis*, *P. maximus*, *A. purpuratus*, *S. broughtonii*, *Pinctada P. martensi*, *Bathymodiolus B. platifrons*, *Homo H. sapiens*, *Xenopus X. tropicaalis*, *D.anio rerio*, and *Caenorhabditis C. elegans*) were filteredfor gene family analysis.. When multiple transcripts (suggesting alternative splicing) were found for a gene, For the gene set of each genome, only the transcript with the longest coding sequence was retainedselected from alternate splice transcripts. Meanwhile, the encoding proteins Genes with less than 50 amino acids were removed from further analysis. Protein sequences were aligned by “all-vs-all blastp BLASTP” (E value = 1e^{-5} by default) [44],. Then the Markov clustering (MCL) algorithm implemented in OrthoMCL was used to group orthologues and paralogues from all input species and clustered using orthomcl (OrthoMCL DB: Ortholog Groups of Protein Sequences, RRID: SCR_007839) 2.0 with the expansion coefficient an inflation value of 1.5 [53]. Finally, to perform multiple sequence

alignment for single copy genes clustered, and concatenate sequence alignment of single-copy gene families. Based on the clustering results, the division of gene family was obtained.

The phylogenetic tree was constructed following procedures described previous studies [13, 54-55]. Briefly, for To construct phylogenetic trees construction and divergence time estimation, protein sequences of each shared single copy genes of *P. generosa* and other 11 other species were used.aligned using MUSCLE (MUSCLE, RRID:SCR_011812), and then the protein alignment results were converted into CDS alignment results. Next, conserved sites were extracted using Gblocks (Gblocks, RRID:SCR_015945) [40], followed by combining all single copy genes according to the species order to form a super alignment matrix. The phylogenetic tree was constructed using maximum likelihood (ML) algorithm in RAxML (RAxML, RRID:SCR_006086) [41] with the optimal amino acid substitution model selected by the PROTGAMMAAUTO parameter. The protein sequences of single-copy orthologs among the 12 species were aligned using MUSCLE v3.7 (RRID:SCR_011812) [56] with default parameters. Phylogenetic relationships were inferred based on the super-matrix estimated from the concatenated alignment of single-copy genes using the maximum likelihood (ML) [57] method implemented in RAxML v2.2 (RRID:SCR_006086) [58] with the optimal amino acid substitution model selected by the PROTGAMMALGX parameter.

Based on gene family identification and phylogenetic analysis, single copy genes and mcmctree in PAML [59] were used to estimate divergence time [60-63]. The time correction points were *C. elegans* and *H. sapiens* (678.3–855.2 MYA), *D. rerio* and *H. sapiens* (413.1–

443.0 MYA), *X. tropicalis* and *H. sapiens* (347.0–357.9 MYA). The time correction points were taken from the Timetree website. The operating parameters of mcmctree: burn in = 10000, sample number = 1000000, sample frequency = 50.

Gene family expansion and contraction

The clustering results of gene families and the phylogenetic tree with divergence time estimated were used to analyze the expansion and contraction of orthologous gene families between ancestor and each of the 12 species (*P. generosa* and the other 11 species) using a stochastic birth and death model with lambda parameter by CAFE (RRID:SCR_005983, version 4.0) [64]. This model was further used to calculate the number of gene families along each lineage on the phylogenetic tree. A probabilistic graphical model was introduced to calculate the probability of transitions in gene family size from parent to child nodes. The family-wide P-Values ~~and Viterbi P-Values~~ were calculated in each lineage based on the conditional likelihood.

Data Availability

The Whole Genome project of *P. generosa* has been deposited at ~~National Genomics Data Center~~NCBI/~~bioproject PRJCA011372~~BioProject PRJNA859289. The raw next-generation sequencing reads of DNA are available at SRA (SRR22190027-SRR22190030)~~GSA (Experiment accession CRX506408, Run accession CRR570510–CRR570525)~~; raw long-read ~~Pacbio~~ PacBio sequencing reads of DNA are available at SRA (SRR22190026~~GSA (Experiment accession CRX506406, Run accession CRR570502–CRR570508)~~; raw next-generation sequencing reads of RNA are available at SRA (SRR22190032)~~GSA (Experiment accession CRX506409, Run accession CRR570526)~~;

raw Hi-C reads are available at SRA (SRR22190025)GSA (Experiment accession CRX506407, Run accession CRR570509); and raw long-read Pacbio PacBio sequencing reads of RNA are available at SRA(SRR22190031)GSA (Experiment accession CRX506410, Run accession CRR570527). The genome assembly data have been deposited under accession No. JAPMAH000000000.1GWHBMBK00000000.

Additional Files

Supplementary Figure S1:. The 17-mer count distribution for the genome size estimation.

Supplementary Figure S2: The enriched KEGG pathways of significantly expanded gene families (p≤0.01, top 20) in *P. generosa*

Supplementary Table S1. : The genome assembly information of bivalves in the public database

Supplementary Table S2:. Statistics of 17-mer analysisThe genome assembly information of bivalve species in the public database.

Supplementary Table S3:. The heterozygosity of bivalves reported

Supplementary Table S4:. The chromosomes information of *P. generosa*Statistics of repetitive element annotations in *P. generosa* using various methods.,

The structural statistics of gene prediction in *P. generosa*.

Supplementary Table S5:. The structural statistics of gene prediction in *P. generosa* Functional annotation of the predicted protein-coding genes in *P. generosa* genome assembly

Supplementary Table S6:. The assembly quality of chromosomal genomes of bivalves by BUSCOThe enriched KEGG pathways of expanded genes in *P. generosa* genome assembly.

Supplementary Table S7: Functional annotation of the predicted protein-coding genes in *P. generosa* genome assembly

Supplementary Table S8: The information of 30,616 gene families of *P. generosa* and 11 other species

Supplementary Table S9: The enriched KEGG pathways of expanded gene families in *P. generosa* genome assembly

Abbreviations

PCG: protein-coding gene; Akt: RAC serine/threonine-protein kinase; bp: base pairs; BLAST: Basic Local Alignment Search Tool; BUSCO: Benchmarking Universal Single-Copy Orthologs; BWA: Burrows-Wheeler Aligner; cAMP: cyclic adenosine monophosphate; CAMs: cell adhesion molecules; cGMP-PKG: cGMP-dependent protein kinase G; Gb: gigabase pairs; GC: guanine-cytosine; GnRH: Gonadotropin-releasing hormone; GO: gene ontology; Iso-seq: Hi-C: High-throughput/resolution chromosome conformation capture; Isoform sequencing; kb: kilobase pairs; KEGG: Kyoto Encyclopedia of Genes and

Genomes; Mb: megabase pairs; MYA: million years ago; NCBI: National Center for Biotechnology Information; NF-kappa B: nuclear factor kappa-B; NOD-like receptor: nucleotide-binding oligomerization domain-like receptor; PacBio: Pacific Biosciences; RAxML: Randomized Axelerated Maximum Likelihood; PI3K: phosphatidylinositol-4,5-bisphosphate 3-kinase catalytic subunit alpha/beta/delta; Randomized Axelerated Maximum Likelihood; Rap1: Ras-related protein1; RNA-seqSeq: RNA sequencing; Iso-seqSeq: Isoform-sequencing; tRNA: transfer RNA; TRP channel: transient receptor potential ion channel.

Competing Interests

The authors declare that they have no competing interests.

Funding

This study was supported by the Taishan Scholar Project Special Fund (to Nansheng Chen), the Strategic Priority Research Program of Chinese Academy of Sciences (XDB42000000), the Chinese Academy of Sciences Pioneer Hundred Talents Program (to Nansheng Chen), the Taishan Scholar Project Special Fund (to Nansheng Chen), and the Qingdao Innovation and Creation Plan (Talent Development Program-5th Annual Pioneer and Innovator Leadership Award to Nansheng Chen, 19-3-2-16-zhc) and an Earmarked Workstation Fund for QRJH (to Chunde Wang and Nansheng Chen).

Authors' Contributions

N.C. and C.W. conceived and designed the study. M.C. and Y.C. prepared the samples. J.W. and Q.X. performed analyses. J.W. wrote the paper with input from co-authors. All authors read and approved the final version for submission.

References

1. González-Peláez SS, Leyva-Valencia I, Pérez-Valencia SA, et al. Distribution limits of the geoduck clams *Panopea generosa* and *P. globosa* on the Pacific coast of Mexico. Malacologia. 2013;56:85-94.
2. Vadopalas B, Pietsch TW, Friedman CS. The proper name for the geoduck: resurrection of *Panopea generosa* Gould , 1850, from the synonymy of *Panopea abrupta* (Conrad, 1849) (Bivalvia: Myoida: Hiatellidae). Malacologia. 2010;52:169-73.
3. Goodwin CL, Pease BC. Geoduck, *Panopea abrupta* (Conrad, 1849), size, density, and quality as related to various environmental parameters in Puget Sound, Washington. J Shellfish Res 1991;10:65-77.
4. Orensanz JM, Hand CM, Parma AM, et al. Precaution in the harvest of Methuselah's clams the difficulty of getting timely feedback from slow-paced dynamics. Can J Fish Aquat Sci. 2004;61:1355-72.
5. Newell RIE. Ecosystem influences of natural and cultivated populations of suspension-feeding bivalve molluscs: A review. J Shellfish Res. 2004;23:51-61.
6. Straus KM, MacDonald PS, Crosson LM, et al. Effects of geoduck aquaculture on the environment: A synthesis of current knowledge., Washington Sea Grant Technical Report WSG-TR 13-02, 2013.
7. Santos A, Aguirre J, Rodríguez-Tovar FJ, et al. Multi-storm events recorded on *Panopea* burrows (Pliocene, Spain): The importance of sequestered information inside burrows. Palaeogeogr, Palaeoclimatol, Palaeoecol. 2018;507:155-67.
8. Bureau D, Hajas W, Hand CM, et al. Age, size structure and growth parameters of

geoducks (*Panopea abrupta*, Conrad 1849) from seven locations in British Columbia sampled in 2001 and 2002. Canadian Technical Report of Fisheries and Aquatic Sciences, 2003, p. 29.

9. Sloan NA, Robinson SMC. Age and gonad development in the geoduck clam *Panopea abrupta* (Conrad) from southern British Columbia, Canada. J Shellfish Res. 1984;4:131-7.

10. Valero JL, Canada O, Madryn P, et al. Geoduck (*Panopea abrupta*) recruitment in the Pacific Northwest: long-term changes in relation to climate. CalCOFI Reports. 2004;45:80-6.

11. Marçais G, Kingsford C. A fast, lock-free approach for efficient parallel counting of occurrences of k-mers. Bioinformatics. 2011;27:764-70.

12. Walker BJ, Abeel T, Shea T, et al. Pilon: an integrated tool for comprehensive microbial variant detection and genome assembly improvement. PloS one. 2014;9:e112963.

13. Burton JN, Adey A, Patwardhan RP, et al. Chromosome-scale scaffolding of de novo genome assemblies based on chromatin interactions. Nat Biotechnol. 2013;31:1119-25.

14. Pendleton M, Sebra R, Pang AW, et al. Assembly and diploid architecture of an individual human genome via single-molecule technologies. Nat Methods. 2015;12:780-6.

15. Dudchenko O, Batra SS, Omer AD, et al. De novo assembly of the *Aedes aegypti* genome using Hi-C yields chromosome-length scaffolds. Science. 2017;356:92-5.

16. Robinson JT, Turner D, Durand NC, et al. Juicebox.js provides a cloud-based

visualization system for Hi-C data. Cell Syst. 2018;6:256-8.

17. Simão FA, Waterhouse RM, Ioannidis P, et al. BUSCO: assessing genome assembly and annotation completeness with single-copy orthologs. Bioinformatics. 2015;31:3210-2.

18. Bergman CM, Quesneville H. Discovering and detecting transposable elements in genome sequences. Brief Bioinform. 2007;8:382-92.

19. Bao W, Kojima KK, Kohany O. Repbase Update, a database of repetitive elements in eukaryotic genomes. Mobile DNA. 2015;6:11.

20. Edgar RC, Myers EW. PILER: identification and classification of genomic repeats. Bioinformatics. 2005;21:i152-8.

21. Price AL, Jones NC, Pevzner PA. De novo identification of repeat families in large genomes. Bioinformatics. 2005;21 Suppl 1:i351-8.

22. Flynn JM, Hubley R, Goubert C, et al. RepeatModeler2 for automated genomic discovery of transposable element families. Proc Natl Acad Sci U S A. 2020;117:9451-7.

23. Benson G. Tandem repeats finder: a program to analyze DNA sequences. Nucleic Acids Res. 1999;27:573-80.

24. Altschul SF, Gish W, Miller W, et al. Basic local alignment search tool. J Mol Biol. 1990;215:403-10.

25. Yu XJ, Zheng HK, Wang J, et al. Detecting lineage-specific adaptive evolution of brain-expressed genes in human using rhesus macaque as outgroup. Genomics. 2006;88:745-51.

26. Birney E, Clamp M, Durbin R. GeneWise and Genomewise. Genome Res. 2004;14:988-95.

27. Stanke M, Morgenstern B. AUGUSTUS: a web server for gene prediction in eukaryotes that allows user-defined constraints. Nucleic Acids Res. 2005;33:W465-7.

28. Guigó R, Knudsen S, Drake N, et al. Prediction of gene structure. J Mol Biol. 1992;226:141-57.

29. Burge C, Karlin S. Prediction of complete gene structures in human genomic DNA. J Mol Biol. 1997;268:78-94.

30. Majoros WH, Pertea M, Salzberg SL. TigrScan and GlimmerHMM: two open source ab initio eukaryotic gene-finders. Bioinformatics. 2004;20:2878-9.

31. Solovyev V, Kosarev P, Seledsov I, et al. Automatic annotation of eukaryotic genes, pseudogenes and promoters. Genome Biol. 2006;7:S10.

32. Korf I. Gene finding in novel genomes. BMC bioinf. 2004;5:59.

33. Kim D, Pertea G, Trapnell C, et al. TopHat2: accurate alignment of transcriptomes in the presence of insertions, deletions and gene fusions. Genome Biol. 2013;14:R36.

34. Trapnell C, Roberts A, Goff L, et al. Differential gene and transcript expression analysis of RNA-seq experiments with TopHat and Cufflinks. Nat Protoc. 2012;7:562-78.

35. Wu TD, Watanabe CK. GMAP: a genomic mapping and alignment program for mRNA and EST sequences. Bioinformatics. 2005;21:1859-75.

36. Haas BJ, Delcher AL, Mount SM, et al. Improving the Arabidopsis genome annotation using maximal transcript alignment assemblies. Nucleic Acids Res. 2003;31:5654-66.

37. Haas BJ, Salzberg SL, Zhu W, et al. Automated eukaryotic gene structure annotation

using EVidenceModeler and the Program to Assemble Spliced Alignments. Genome Biol. 2008;9:R7.

38. Li L, Stoeckert CJ, Jr., Roos DS. OrthoMCL: identification of ortholog groups for eukaryotic genomes. Genome Res. 2003;13:2178-89.

39. Edgar RC. MUSCLE: multiple sequence alignment with high accuracy and high throughput. Nucleic Acids Res. 2004;32:1792-7.

40. Talavera G, Castresana J. Improvement of phylogenies after removing divergent and ambiguously aligned blocks from protein sequence alignments. Syst Biol. 2007;56:564-77.

41. Stamatakis A. RAxML-VI-HPC: maximum likelihood-based phylogenetic analyses with thousands of taxa and mixed models. Bioinformatics. 2006;22:2688-90.

42. Yang Z. PAML 4: phylogenetic analysis by maximum likelihood. Mol Biol Evol. 2007;24:1586-91.

43. Thorne JL, Kishino H, Painter IS. Estimating the rate of evolution of the rate of molecular evolution. Mol Biol Evol. 1998;15:1647-57.

44. Vogel JP, Garvin DF, Mockler TC, et al. Genome sequencing and analysis of the model grass *Brachypodium distachyon*. Nature. 2010;463:763-8.

45. Blanc G, Wolfe KH. Widespread paleopolyploidy in model plant species inferred from age distributions of duplicate genes. The Plant cell. 2004;16:1667-78.

46. Sanderson MJ. r8s: inferring absolute rates of molecular evolution and divergence times in the absence of a molecular clock. Bioinformatics. 2003;19:301-2.

47. Han MV, Thomas GW, Lugo-Martinez J, et al. Estimating gene gain and loss rates in

the presence of error in genome assembly and annotation using CAFE 3. Mol Biol Evol. 2013;30:1987-97.

48. Thai BT, Lee YP, Gan HM, et al. Whole genome assembly of the snout otter clam, *Lutraria rhynchaena*, using Nanopore and Illumina Data, benchmarked against bivalve genome assemblies. Front Genet. 2019;10:1158.

49. Sun J, Zhang Y, Xu T, et al. Adaptation to deep-sea chemosynthetic environments as revealed by mussel genomes. Nat Ecol Evol. 2017;1:0121.

50. Peñaloza C, Gutierrez AP, Eöry L, et al. A chromosome-level genome assembly for the Pacific oyster *Crassostrea gigas*. Gigascience. 2021;10:giab020.

51. Peng J, Li Q, Xu L, et al. Chromosome-level analysis of the *Crassostrea hongkongensis* genome reveals extensive duplication of immune-related genes in bivalves. Mol Ecol Resour. 2020;20:980-94.

52. Wu B, Chen X, Yu M, et al. Chromosome-level genome and population genomic analysis provide insights into the evolution and environmental adaptation of Jinjiang oyster *Crassostrea ariakensis*. Mol Ecol Resour. 2022;22:1529-44.

53. Du X, Fan G, Jiao Y, et al. The pearl oyster *Pinctada fucata martensii* genome and multi-omic analyses provide insights into biomineralization. Gigascience. 2017;6:1-12.

54. Yang JL, Feng DD, Liu J, et al. Chromosome-level genome assembly of the hard-shelled mussel *Mytilus coruscus*, a widely distributed species from the temperate areas of East Asia. Gigascience. 2021;10:giab024.

55. McCartney MA, Auch B, Kono T, et al. The genome of the zebra mussel, *Dreissena polymorpha*: a resource for comparative genomics, invasion genetics, and biocontrol.

G3 Genes Genom Genet 2021;12:jkab423.

56. Zhang T, Yin J, Tang S, et al. Dissecting the chromosome-level genome of the Asian Clam (*Corbicula fluminea*). Sci Rep. 2021;11:15021.

57. Ran Z, Li Z, Yan X, et al. Chromosome-level genome assembly of the razor clam *Sinonovacula constricta* (Lamarck, 1818). Mol Ecol Resour. 2019;19:1647-58.

58. Li J, Zhou Y, Zhou Z, et al. Comparative transcriptome analysis of three gonadal development stages reveals potential genes involved in gametogenesis of the fluted giant clam (*Tridacna squamosa*). BMC genomics. 2020;21:872.

59. Jiang H, Liu H, Ma X, et al. Transcriptome analysis of *Procambarus clarkii* to screen genes related to ovary development, immunity and growth. J Fish China. 2021;45:396-414.

60. Ren Y, Liu W, Pearce CM, et al. Effects of selected mixed-algal diets on growth and survival of early postset juveniles of the Pacific geoduck clam, *Panopea generosa* (Gould, 1850). Aquacult Nutr. 2014;21.

61. Nava-Gómez GE, Garcia-Esquivel Z, Carpizo-Ituarte E, et al. Survival and growth of geoduck clam larvae (*Panopea generosa*) in flow-through culture tanks under laboratory conditions. Aquacult Res. 2018;49:294-300.

62. Li H, Liu M, Ye S, et al. De novo assembly, gene annotation, and molecular marker development using Illumina paired-end transcriptome sequencing in the clam *Saxidomus purpuratus*. Genes Genom. 2017;39:675-85.

63. Hu F, Pan L, Cai Y, et al. Deep sequencing of the scallop *Chlamys farreri* transcriptome response to tetrabromobisphenol A (TBBPA) stress. Mar Genom. 2015;19:31-8.

1. González-Peláez SS, Leyva-Valencia I, Pérez-Valencia SA, et al. Distribution limits of the geoduck clams *Panopea generosa* and *P. globosa* on the Pacific coast of Mexico. *Malacologia*. 2013;56:85-94.
2. Vadopalas B, Pietsch TW, Friedman CS. The proper name for the geoduck: resurrection of *Panopea generosa* Gould, 1850, from the synonymy of *Panopea abrupta* (Conrad, 1849) (Bivalvia: Myoida: Hiatellidae). *Malacologia*. 2010;52:169-173.
3. Goodwin CL, Pease BC. Geoduck, *Panopea abrupta* (Conrad, 1849), size, density, and quality as related to various environmental parameters in Puget Sound, Washington. *J Shellfish Res.* 1991;10:65-77.
4. Orensanz JM, Hand CM, Parma AM, et al. Precaution in the harvest of Methuselah's clams the difficulty of getting timely feedback from slow-paced dynamics. *Can J Fish Aquat Sci*. 2004;61:1355-1372.
5. Newell RIE. Ecosystem influences of natural and cultivated populations of suspension-feeding bivalve molluscs: A review. *J Shellfish Res.* 2004;23:51-61.
6. Straus KM, MacDonald PS, Crosson LM, et al. Effects of geoduck aquaculture on the environment: A synthesis of current knowledge., Washington Sea Grant Technical Report WSG-TR 13-02, 2013.
7. Santos A, Aguirre J, Rodríguez-Tovar FJ, et al. Multi-storm events recorded on *Panopea* burrows (Pliocene, Spain): The importance of sequestered information inside burrows. *Palaeogeogr Palaeoclimatol Palaeoecol*. 2018;507:155-167.
8. Bureau D., Hajas W., Hand C.M., et al. Age, size structure and growth parameters of geoducks (*Panopea abrupta*, Conrad 1849) from seven locations in British Columbia sampled in 2001 and 2002. Canadian Technical Report of Fisheries and Aquatic Sciences, 2003, p. 29.
9. Sloan NA, Robinson SMC. Age and gonad development in the geoduck clam *Panopea abrupta* (Conrad) from southern British Columbia, Canada. *J Shellfish Res.* 1984;4:131-137.
10. Valero JL, Canada O, Madryn P, et al. Geoduck (*Panopea abrupta*) recruitment in the Pacific Northwest: long-term changes in relation to climate. *CalCOFI Reports*. 2004;45:80-86.
11. Thai BT, Lee YP, Gan HM, et al. Whole genome assembly of the snout otter clam, *Lutraria rhynchaena*, using Nanopore and Illumina Data, benchmarked against bivalve genome assemblies. *Front Genet.* 2019;10:1158.
12. Sun J, Zhang Y, Xu T, et al. Adaptation to deep-sea chemosynthetic environments as revealed by mussel genomes. *Nat Ecol Evol*. 2017;1:0121.
13. Ran Z, Li Z, Yan X, et al. Chromosome-level genome assembly of the razor clam *Sinonovacula constricta* (Lamarck, 1818). *Mol Ecol Resour.* 2019;19:1647-1658.
14. Halanych KM, Kocot KM. Genome evolution: Shellfish genes. *Nat Ecol Evol.* 2017;1:0142.
15. Peñaloza C, Gutierrez AP, Eöry L, et al. A chromosome-level genome assembly for the Pacific oyster *Crassostrea gigas*. *GigaScience*. 2021;10:giab020.
16. Peng J, Li Q, Xu L, et al. Chromosome-level analysis of the *Crassostrea hongkongensis* genome reveals extensive duplication of immune-related genes in bivalves. *Mol Ecol Resour.* 2020;20:980-994.

17. Wu B, Chen X, Yu M, et al. Chromosome-level genome and population genomic analysis provide insights into the evolution and environmental adaptation of Jinjiang oyster *Crassostrea ariakensis*. *Mol Ecol Resour.* 2022;22:1529-1544.
18. Du X, Fan G, Jiao Y, et al. The pearl oyster *Pinctada fucata* martensii genome and multi-omic analyses provide insights into biomineralization. *GigaScience*. 2017;6:1-12.
19. Yang JL, Feng DD, Liu J, et al. Chromosome-level genome assembly of the hard-shelled mussel *Mytilus coruscus*, a widely distributed species from the temperate areas of East Asia. *GigaScience*. 2021;10:giab024.
20. Holmes A, Darbyshire T, Brennan M, et al. The genome sequence of *Gari tellinella* (Lamarck, 1818), a sunset clam. *Wellcome Open Res*. 2022;7:116.
21. Zhang T, Yin J, Tang S, et al. Dissecting the chromosome-level genome of the Asian Clam (*Corbicula fluminea*). *Sci Rep*. 2021;11:15021.
22. Marçais G, Kingsford C. A fast, lock-free approach for efficient parallel counting of occurrences of *k*-mers. *Bioinformatics*. 2011;27:764-770.
23. Simão FA, Waterhouse RM, Ioannidis P, et al. BUSCO: assessing genome assembly and annotation completeness with single-copy orthologs. *Bioinformatics*. 2015;31:3210-2.
24. Li J, Zhou Y, Zhou Z, et al. Comparative transcriptome analysis of three gonadal development stages reveals potential genes involved in gametogenesis of the fluted giant clam (Tridacna squamosa). *BMC Genom*. 2020;21:872.
25. Jiang H, Liu H, Ma X, et al. Transcriptome analysis of Procambarus clarkii to screen genes related to ovary development,immunity and growth. *J Fish China*. 2021;45:396-414.
26. Ren Y, Liu W, Pearce CM, et al. Effects of selected mixed-algal diets on growth and survival of early postset juveniles of the Pacific geoduck clam, *Panopea generosa* (Gould, 1850). *Aquac Nutr.* 2014;21.
27. Nava-Gómez GE, Garcia-Esquivel Z, Carpizo-Ituarte E, et al. Survival and growth of geoduck clam larvae (*Panopea generosa*) in flow-through culture tanks under laboratory conditions. *Aquac Res*. 2018;49:294-300.
28. Zdobnov EM, Apweiler R. InterProScan--an integration platform for the signature-recognition methods in InterPro. *Bioinformatics*. 2001;17:847-8.
29. Perestenko PV, Pooler AM, Noorbakhshnia M, et al. Copines-1, -2, -3, -6 and -7 show different calcium-dependent intracellular membrane translocation and targeting. *FEBS J*. 2010;277:5174-5189.
30. Jumper J, Evans R, Pritzel A, et al. Highly accurate protein structure prediction with AlphaFold. *Nature*. 2021;596:583-589.
31. Bisbal-Pardo CI, Del Río-Portilla MA, Rocha-Olivares A. The complete mitochondrial DNA of the Pacific Geoduck clam (*Panopea generosa*). *Mitochondrial DNA A*. 2016;27:1955-6.
32. Green M, J S. Molecular cloning: a laboratory manual. 4th Edn. Vol. II. New York, NK: Cold Spring Harbor Laboratory Press.
33. Walker BJ, Abeel T, Shea T, et al. Pilon: an integrated tool for comprehensive microbial variant detection and genome assembly improvement. *PloS ONE*. 2014;9:e112963.
34. Burton JN, Adey A, Patwardhan RP, et al. Chromosome-scale scaffolding of de novo

genome assemblies based on chromatin interactions. *Nat Biotechnol*. 2013;31:1119-25.
35. Chen Y, Chen Y, Shi C, et al. SOAPnuke: a MapReduce acceleration-supported software for integrated quality control and preprocessing of high-throughput sequencing data. *GigaScience*. 2018;7:1-6.
36. Pendleton M, Sebra R, Pang AW, et al. Assembly and diploid architecture of an individual human genome via single-molecule technologies. *Nat Methods*. 2015;12:780-6.
37. Durand NC, Shamim MS, Machol I, et al. Juicer provides a one-click system for analyzing loop-resolution Hi-C experiments. *Cell Syst*. 2016;3:95-8.
38. Dudchenko O, Batra SS, Omer AD, et al. De novo assembly of the *Aedes aegypti* genome using Hi-C yields chromosome-length scaffolds. *Science*. 2017;356:92-95.
39. Robinson JT, Turner D, Durand NC, et al. Juicebox.js provides a cloud-based visualization system for Hi-C data. *Cell Syst*. 2018;6:256-258.
40. Krzywinski M, Schein J, Birol I, et al. Circos: an information aesthetic for comparative genomics. *Genome Res*. 2009;19:1639-45.
41. Bergman CM, Quesneville H. Discovering and detecting transposable elements in genome sequences. *Brief Bioinform*. 2007;8:382-92.
42. Bao W, Kojima KK, Kohany O. Repbase Update, a database of repetitive elements in eukaryotic genomes. *Mob DNA*. 2015;6:11.
43. Benson G. Tandem repeats finder: a program to analyze DNA sequences. *Nucleic Acids Res.* 1999;27:573-80.
44. Altschul SF, Gish W, Miller W, et al. Basic local alignment search tool. *J Mol Biol*. 1990;215:403-10.
45. Slater GS, Birney E. Automated generation of heuristics for biological sequence comparison. *BMC Bioinform*. 2005;6:31.
46. Stanke M, Keller O, Gunduz I, et al. AUGUSTUS: ab initio prediction of alternative transcripts. *Nucleic Acids Res.* 2006;34:W435-9.
47. Johnson AD, Handsaker RE, Pulit SL, et al. SNAP: a web-based tool for identification and annotation of proxy SNPs using HapMap. *Bioinformatics*. 2008;24:2938-9.
48. Kim D, Pertea G, Trapnell C, et al. TopHat2: accurate alignment of transcriptomes in the presence of insertions, deletions and gene fusions. *Genome biology*. 2013;14:R36.
49. Trapnell C, Roberts A, Goff L, et al. Differential gene and transcript expression analysis of RNA-seq experiments with TopHat and Cufflinks. *Nat Protoc*. 2012;7:562-78.
50. Wu TD, Watanabe CK. GMAP: a genomic mapping and alignment program for mRNA and EST sequences. *Bioinformatics*. 2005;21:1859-75.
51. Haas BJ, Delcher AL, Mount SM, et al. Improving the *Arabidopsis* genome annotation using maximal transcript alignment assemblies. *Nucleic Acids Res.* 2003;31:5654-66.
52. Holt C, Yandell M. MAKER2: an annotation pipeline and genome-database management tool for second-generation genome projects. *BMC Bioinform*. 2011;12:491.
53. Li L, Stoeckert CJ, Jr., Roos DS. OrthoMCL: identification of ortholog groups for eukaryotic genomes. *Genome Res*. 2003;13:2178-89.
54. Song H, Guo X, Sun L, et al. The hard clam genome reveals massive expansion and

diversification of inhibitors of apoptosis in Bivalvia. BMC Biol. 2021 Jan 25;19(1):15.
55. Tian HF, Hu QM, Li Z. A high-quality de novo genome assembly of one swamp eel (*Monopterus albus*) strain with PacBio and Hi-C sequencing data. *G3: Genes Genom Genet*. 2021 Jan 18;11(1):jkaa032.
56. Edgar RC. MUSCLE: multiple sequence alignment with high accuracy and high throughput. *Nucleic Acids Res.* 2004;32:1792-7.
57. Guindon S, Gascuel O. A simple, fast, and accurate algorithm to estimate large phylogenies by maximum likelihood. *Syst Biol.* 2003;52:696-704.
58. Stamatakis A. RAxML-VI-HPC: maximum likelihood-based phylogenetic analyses with thousands of taxa and mixed models. *Bioinformatics*. 2006;22:2688-90.
59. Yang Z. PAML 4: phylogenetic analysis by maximum likelihood. *Mol Biol Evol.* 2007;24:1586-91.
60. Thorne JL, Kishino H, Painter IS. Estimating the rate of evolution of the rate of molecular evolution. *Mol Biol Evol.* 1998;15:1647-57.
61. Vogel JP, Garvin DF, Mockler TC, et al. Genome sequencing and analysis of the model grass Brachypodium distachyon. *Nature*. 2010;463:763-8.
62. Blanc G, Wolfe KH. Widespread paleopolyploidy in model plant species inferred from age distributions of duplicate genes. *The Plant cell*. 2004;16:1667-78.
63. Sanderson MJ. r8s: inferring absolute rates of molecular evolution and divergence times in the absence of a molecular clock. *Bioinformatics*. 2003;19:301-2.
64. Han MV, Thomas GW, Lugo-Martinez J, et al. Estimating gene gain and loss rates in the presence of error in genome assembly and annotation using CAFE 3. *Mol Biol Evol.* 2013;30:1987-97.

Table 1.: Statistics of the DNA sequence data used for *P. generosa* genome assembly

Source	Platform	Library size	Clean data (Gb)	Read length (bp)	Sequencing coverage (x)
**Genome-short reads**	BGISEQ-500	300 bp	258.19	150	181
**Genome-long reads**	PacBio sequel	20 Kb	164.46	26,513*	115
**Hi-C**	BGISEQ-500	300 bp	233.49	150	163

[a]"26,513*" indicated the N50 of subreads.

Table 2~~.~~ : Statistics of the genome assembly of *P. generosa*

Statistics	Contig	Scaffold	Chromosome~~Contig~~
**Total Number (#)**	2,086	39	19~~2,086~~
**Total length (bp)**	1,473,137,789	1,474,161,289	1,432,060,667~~1,473,137,789~~
**Average Length (bp)**	706,202	37,799,007	75,371,614~~706,202~~
**N50 Length (bp)**	1,571,249	73,788,920	76,670,739~~1,571,249~~
**N90 Length (bp)**	418,579	53,843,121	53,843,121~~418,579~~
**Maximum Length (bp)**	6,469,558	101,196,518	101,196,518~~6,469,558~~
**Minimum Length (bp)**	17	29,000	1,432,060,667~~17~~
**GC content**	34.33%	34.33%	34.33%~~34.33%~~
**Anchored rate (%)**		94.70%	

Table 3.: Repetitive element annotations in *P. generosa*

Repetitive sequence			Transposable elements		
**Type**	**Length (bp)**	**% in genome**	**Type**	**Length (bp)**	**% in genome**
**DNA**	318426608	21.6	**DNA**	318426608	21.6
**LINE**	134171392	9.1	**LINE**	134171392	9.1
**SINE**	27249093	1.85	**SINE**	27249093	1.85
**LTR**	55380608	3.76	**LTR**	55380608	3.76
**Other**	31141	0	**Other**	31141	0
**Unknown**	151891763	10.3	**Unknown**	151891763	10.3
**Tandem repeat**	347641207	23.58	**Total**	583000390	39.55
**Total**	854873570	57.99			

Table 4. : BUSCO results for analysis of genome completeness for *P. generosa*

Type	Number of genes	Percentage (%)
Complete BUSCOs (C)	889	90.9
Complete and single-copy BUSCOs (S)	837	85.6
Complete and duplicated BUSCOs (D)	52	5.3
Fragmented BUSCOs (F)	43	4.4
Missing BUSCOs (M)	46	4.7
Total BUSCO groups searched	978	100

Type	Genome assembly		Gene set	
	Number of genes	Percentage (%)	Number of genes	Percentage (%)
Complete BUSCOs (C)	887	93.0	844	88.4
Complete and single-copy BUSCOs (S)	842	88.3	795	83.3
Complete and duplicated BUSCOs (D)	45	4.7	49	5.1
Fragmented BUSCOs (F)	34	3.6	49	5.1
Missing BUSCOs (M)	33	3.4	61	6.5
Total BUSCO groups	954	100	954	100

Table 5~~.~~ : The ~~protein-coding genes~~PCGs of *P. generosa* and ~~other~~ 11 other species for evolutionary analysis

Species	Total genes	Unclustered genes	Families	Unique families	Ave. genes per family
P. generosa	35,034	6,168	12,034	1,749	2.4
A. purpuratus	26,256	3,720	13,196	290	1.71
B. platifrons	33,584	3,197	12,409	1,775	2.45
C. gigas	28,402	3,638	11,818	828	2.1
D. rerio	25,444	1,791	9,210	295	2.57
H. sapiens	20,229	1,488	9,251	226	2.03
P. yessoensis	24,521	1,704	13,017	137	1.75
P. maximus	26,152	1,518	13,276	164	1.86
P. martensi	25,526	2,403	11,043	318	2.09
S. broughtonii	24,045	2,770	11,314	538	1.88
X. tropicalis	19,967	1,016	9,226	159	2.05
C. elegans	33,552	5,600	8,201	3,720	3.41

Figure 1.–: The *P. generosa* genome contig contact matrix using Hi-C data and landscape. (A) Hi-C analysis of *P. generosa* genome contigs. Chromosomes are arranged in the size order from left to right and from top to bottom. The color bar illuminates the logarithm of the contact density from red (10) to white (0) in the plot. (B) The genomic landscape of *P. generosa*: from outer to inner circles: a, the 19 chromosomes; b–d, repetitive element density, gene density, and GC density across the genome, respectively, drawn in 1 Mb non-overlapping windows.

Figure 2: Chromosome synteny of *P. generosa* and *S. constricta*

Figure 23.–: The distribution of single-copy orthologs, multiple-copy orthologs, unique paralogs, other orthologs, and unclustered genes in *P. generosa* and related species.

Figure 34.–: Distribution of shared gene families among *P. generosa*, *P. martensi*, *S. broughtonii*, and *P. yessoensis*. Intersections between species indicate the numbers of shared gene families, whereas unique family numbers are shown in species-specific areas. The center represents the number of families shared by all the 4 species.

Figure 45.–: Phylogenetic analysis of *P. generosa* with related species. The estimated species divergence time (million years ago) and the 95% confidential intervals are labeled at each branch site. Divergence times used for time recalibration is illuminated as red dots in the tree.

Figure 56. : Dynamic evolution and distribution of gene families among *P. generosa* and related species. Green and red numbers represent gene family expansion and contraction, respectively. The numbers of gene gains (+) and losses (−) are shown on the branches, which are also displayed as pie plots: the green part for gene gain, the red part for gene losses and the blue part for gene remained. The divergence times are dated and displayed below the phylogenetic tree. MRCA: most recent common ancestor.

Figure 7. Comparative analysis of gene families based on Pfam annotation in *P. generosa* and related bivalves. A. The 65 most abundant gene families that *P. generosa* possessing more gene numbers than 8 other bivalves. B. The phylogenetic tree of genes annotated to PF07002 domains in *P. generosa* and *S. constricta*, shown in the background colors of red and pink, respectively. C. Chromosomal distribution of copine genes predicted in *P. generosa genome*. D. Domain structures of copine genes predicted in *P. generosa* genome. E. The 3D structures of *Pg02g00048* gene in *P. generosa* and *XP_0533888641* gene in *Mercenaria mercenaria*.

This manuscript file contains the revised main text and Figure legends without tracks of revisions.
Click here to access/download;Manuscript;Manuscript_without_tracks_of_re

Chromosome-level genome assembly of the Pacific geoduck *Panopea generosa* reveals major inter- and intra-chromosomal rearrangements and substantial expansion of the copine gene family

Jing Wang[1,2,3], Qing Xu[1,2,3], Min Chen[4], Yang Chen[1,2,3], Chunde Wang[4,5*], Nansheng Chen[1,2,3,6*]

[1]CAS Key Laboratory of Marine Ecology and Environmental Sciences, Institute of Oceanology, Chinese Academy of Sciences, Qingdao, China

[2]Laboratory of Marine Ecology and Environmental Science, Qingdao National Laboratory for Marine Science and Technology, Qingdao, China

[3]Center for Ocean Mega-Science, Chinese Academy of Sciences, Qingdao, China

[4]Yantai Institute of Coastal Zone Research and Center for Ocean Mega-Science, Chinese Academy of Sciences, Yantai, China

[5]Marine Science and Engineering College, Qingdao Agricultural University, Qingdao, China

[6]Department of Molecular Biology and Biochemistry, Simon Fraser University, Burnaby, BC, Canada

Jing Wang Email: wangjing2019@qdio.ac.cn; Qing Xu Email: xuqing_77@163.com; Min Chen Email: mchen@yic.ac.cn; Yang Chen Email: cy4043@hevttc.edu.cn.

∗Correspondence address. Chunde Wang, Yantai Institute of Coastal Zone Research and Center for Ocean Mega-Science, Chinese Academy of Sciences, Yantai, China. E-mail: chundewang2007@163.com; Nansheng Chen, CAS Key Laboratory of Marine Ecology and Environmental Sciences, Institute of Oceanology, Chinese Academy of Sciences,

Qingdao, China. Email: chenn@qdio.ac.cn

Abstract

The Pacific geoduck *Panopea generosa* (class Bivalvia, order Adapedonta, family Hiatellidae, genus *Panopea*) is the largest known burrowing bivalve with considerable commercial value. Pacific geoduck and other geoduck clams play important roles in maintaining ecosystem health for their filter feeding habit and coupling pelagic and benthic processes. Chromosome-level genomes of geoduck clams will contribute to genetic breeding, as well as ecosystem and climate change biology. Here, we report the first high-quality chromosome-level genome assembly of *P. generosa* with the purpose to unravel its phylogenetic characteristics and molecular mechanisms of its life strategies, and promote research on genetic breeding. The assembled *P. generosa* genome consists of 19 chromosomes with a size of 1.47 Gb, a contig N50 of 1.6 Mb and a scaffold N50 of 73.8 Mb. BUSCO analysis showed 93.0% completeness. Comparative analysis of the genomes of two closely related species in the order Adapedonta, *P. generosa* and *Sinonovacula constricta*, revealed major inter- and intra-chromosomal exchanges. Of the 35,034 predicted protein-coding genes (PCGs), 30,700 genes (87.63%) were functionally annotated. Comparative analysis of the genomes of *P. generosa* and 11 related species identified 507 expanded gene families and 875 contracted gene families in *P. generosa*. Enrichment analysis revealed significant expansion of immune and gonad development gene families that may promote its complex survival strategies. In particular, the copine gene family, which plays an important role in calcium signaling, membrane trafficking, and cytoskeletal dynamics, and has been implicated in several physiological and pathological processes such as neuronal development, immune response substantially expanded in *P.*

genererosa with 22 members annotated. The availability of a chromosome-level *P. generosa* genome assembly and its annotated gene set provide a useful molecular platform for research on its genetic breeding.

Keywords: *Panopea generosa*, chromosome-level genome assembly, genetic breeding, evolutionary adaptation

Introduction

The Pacific geoduck *Panopea generosa* is one member of genus *Panopea* which includes the world's largest burrowing bivalves. *P. generosa* is usually found in low intertidal and subtidal sediments throughout the northeast Pacific coast, including the United States (Alaska, Washington, and California), Canada (British Columbia), and Mexico (north Baja Pacific Coast) [1, 2]. Geoducks can reach more than 25 cm in shell length, and more than 100 cm in siphon length [3]. Geoduck adults are usually buried in muddy-sandy sediment at depths ranging 60–100 cm, with only their siphon tips exposed to respire, capture food, and release secretion/excretion products and gametes. The sedentary behavior may contribute to their long life spans (which can be as long as 168 years) for *P. generosa* [4]. Due to these unique life strategies, it is expected that geoduck should have distinctive growth and development mechanisms, especially in relation to benthic life and immune system.

Geoduck clams play important roles in maintaining ecosystem health for their filter feeding habit and coupling pelagic and benthic processes by ejecting undigested mucus-bound feces and pseudo feces to the sediment surface. They are prey for sea otters, fishes, crabs, and sea stars [5, 6]. As marine calcifiers, shell concentrations of *Panopea* inside Scalichnus burrows have been analyzed to reconstruct the sequence of events related to storm events [7]. Geoduck clams possess great commercial fishery value in Canada and the USA [8]. Since the recruitment of geoducks have been low [9] due to overfishing and their vulnerability to environmental changes [10], there has been an increasing interest in genetic breeding of geoducks.

The assembled genome sizes of bivalves vary widely, ranging from 543.9 Mb in *Lutraria thynchaena* [11] to 2.6 Gb in *Modiolus philippinarum* [12] (Supplementary Table S1). Among bivalves, the genome sizes of most superorder Imparidentia species ranged from 1 Gb to 1.8 Gb, and that of the species in the order Adapedonta, which includes *P. generosa*, ranged from 1 Gb to 1.5 Gb [13].

The numbers of chromosomes also vary substantially among bivalves, suggesting active genome recombination in evolution in bivalves [14]. While the species of the order Ostreida, including *Crassostrea gigas* [15], *Crassostrea virginica*, *Crassostrea hongkongensis* [16], *Crassostrea ariakensis* [17], Crassostrea angulate, and Ostrea edulis, have 10 chromosomes, the species of the order Ostreida, including *Pinctada fucata* [18], *Pinctada imbricata* and the species of the order Mytilida including *Mytilus coruscus* [19] and *Mytilus edulis* have 14 chromosomes. The chromosome numbers of the order Cardiida varied from 17-19 [20]. Interestingly, the numbers of chromosomes of species in most other orders including Venerida, Arcida, and Pectinida, are 19, except for *Corbicula fluminea* [21] possessing 18 chromosomes and *Argopecten* scallop possessing 16 chromosomes. The reported numbers of chromosomes of species in the order Adapedonta, which includes *P. generosa*, are also 19.

Nevertheless, high-quality chromosome-level reference genome of *P. generosa* is currently not available, hindering the development of geoduck genetic breeding programs. In this study, we report the first chromosome-scale genome assembly for *P. generosa* generated using cutting-edge technologies including next-generation sequencing, long read sequencing, and high-throughput chromosome conformation capture (Hi-C)

technologies. We further performed gene family clustering, phylogenetic analysis, and gene family expansion and contraction, in order to understand its adaptation, growth, development and immunity. The availability of the genome information will facilitate research in molecular evolution and genetic breeding.

Results

Genome sequencing and assembly

The genome size of *P. generosa* was estimated to be 1.47 Gb using *k*-mer analysis (Table 1) [22]. The heterozygosity and repeated sequence content were estimated to be 1.37% and 57.99% (Supplementary Figure S1 and Table S2), respectively. The heterozygosity of *P. generosa* was comparable to those of most bivalves (Supplementary Table S3). Genome assembly using PacBio long reads (N50 = 26,513 bp) and Falcon assembler obtained an initial size of 1.51 Gb. Further assembly using Hi-C data obtained a genome with 19 pseudomolecules, suggesting 19 chromosomes of the *P. generosa* genome (Figure 1A), with an anchoring rate of 94.70%. This genome assembly has a total length of 1,474,161,289 bp with a contig N50 of 1.57 Mb and a scaffold N50 of 73.79 Mb (Figure 1B; Table 2; Supplementary Table S4). As expected, the genomic regions with low gene density typically had high repeat content, while the regions with high repeat content usually had high GC content.

The assembled genome size of *P. generosa* (1.47 Gb) fell in the range of the reported genomes of bivalves, which varied from 543.9 Mb in *Lutraria thynchaena* [11] to 2.6 Gb in *Modiolus philippinarum* [12] (Supplementary Table S1).

Genome annotation and evaluation

The majority (57.99%) of the *P. generosa* genome was repetitive elements estimated using *de novo* searching and homolog prediction (Table 3). Distribution of these repetitive elements was uneven with repetitive content per 1 Mb varied from 34.76% to 84.89% (Figure 1B). DNA transposons (21.6%), long interspersed nuclear elements (LINEs, 9.1%) and long terminal repeats (LTRs, 3.76%) were the top three categories of repetitive elements in the *P. generosa* genome (Table 3).

A total number of 35,034 PCGs were annotated in the *P. generosa* genome. The mean number of exons per gene was 5.78 (Supplementary Table S4). Of these PCGs, 30,700 genes were annotated to contain conserved functional motifs (Supplementary Table S5, Table S7).

To evaluate the completeness of the assembly, the *P. generosa* genome assembly and annotated protein-coding gene set were assessed using BUSCO [23] with the metazoa_odb10 database (954 core genes), respectively. For the genome assembly, we found that 93.0% of core genes were identified as full-length in the *P. generosa* genome (Table 4), compared to 91.5% of the closely related species *S. constricta* [13] (Supplementary Table S6). Regarding the gene set, 88.4% of core genes were identified as full-length, suggesting that the quality of the gene set could be further improved.

Chromosomal synteny analysis between *P. generosa* and *S. constricta*

Although the *P. generosa* genome has 19 chromosomes as many other species in the order Adapedota, chromosomal synteny between *P. generosa* and other species in this order remains unknown. Comparative analysis between the genomes of *P. generosa* and *S. constricta*, whose genome has been assembled at the chromosome-level revealed that

these two genomes have good chromosomal collinearity in general (Figure 2A). One-to-one chromosomal correspondences between *P. generosa* and *S. constricta* were obvious. Indeed, 17 of 19 *P. generosa* chromosomes showed clear one-to-one r correspondences *S. constricta* chromosomes (Figure 2A). However, major inter-chromosomal exchanges were evident. For example, *P. generosa Pg02* matched to two *S. constricta* chromosomes (*Chr1* and *Chr10*). Similarly, *P. generosa Pg11* matched well to two *S. constricta* chromosomes (*Chr1* and *Chr10*).

In addition to these major inter-chromosomal exchange events, comparative analysis of these two genomes also revealed that extensive intra-chromosomal recombination events, which resulted in little co-linearity within chromosomes. Instead of a clear diagonal linear relationship between genes of these two species *P. generosa* and *S. constricta*, a near random scattering of the relationships were observed (Figure 2A). These intra-chromosomal recombination events were also clearly shown in Figure 2B.

Comparative analysis of gene families and evolutionary analysis

In total, 30,616 gene families were identified among *P. generosa* and 11 other species (*Pinctada martensi*, *C. gigas*, *Bathymodiolus platifrons*, *Patinopecten yessoensis*, *Pecten maximus*, *Argopecten purpuratus*, *Scapharca broughtonii*, *Homo sapiens*, *Xenopus tropicaalis, Danio rerio, and Caenorhabditis elegans*) (Table 5, Figure 3, Supplementary Table S8). As compared with the other 11 species, there were 7917 genes belonging to 1749 gene families that are specific to *P. generosa*. Comparative analysis of the genes of *P. martensi*, *S. broughtonii*, *P. yessoensis*, and *P. generosa* revealed 6490 common gene families shared by these species and 2902 gene families specific to *P. generosa* (Figure

4). Phylogenetic analysis using 326 single-copy orthologous gene families from these 12 species showed that *P. generosa* was tightly clustered with other bivalves as expected (Figure 5). According to the phylogenetic tree, the divergence time of *P. generosa* from its nearest node was approximately 491.5 Mya (Figure 5). In addition, the divergence time of *P. generosa* is earlier than other bivalves, which is consistent with that of *S. constricta* [13], a species close to *P. generosa*. *B. platifrons*, *C. gigas* and *P. martensi*, which have 10 or 14 chromosomes, were clustered as a single clade and diverged from other bivalves with 19 chromosomes in the phylogenetic tree.

A total of 507 expanded gene families (involving 2,734 genes) and 875 contracted gene families (involving 792 genes) were identified in *P. generosa* genome compared to the most recent common ancestor of both *P. generosa* and the other 11 species (Figure 6). KEGG analysis revealed 166 pathways from the expanded gene families were enriched with various biological processes (Supplementary Table S9) The enrichment analysis suggested that the significantly expanded genes of *P. generosa* were mainly represented in organismal systems, human diseases, and environmental information processing, such as phototransduction, Fluid shear stress and atherosclerosis, Phosphatidylinositol signaling system, suggesting their important contribution to the adaptation of benthic bivalves.

Meanwhile, according to the enriched KEGG pathways of expanded gene families in *P. generosa* (Supplementary Table S9), there were a few significant enriched pathways (Qvalue < 0.05) related to gonad development. For example, adrenergic signaling in cardiomyocytes, and glycine, serine and threonine metabolism which were found related

to spermatogenesis of the fluted giant clam *Tridacna squamosa* [24]. Moreover, oocyte meiosis, apoptosis, Ras signaling pathway, calcium signaling pathway, steroid hormone biosynthesis, GnRH signaling pathway, insulin signaling pathway, oxytocin signaling pathway, and ovarian steroidogenesis were documented to be enriched in *Procambarus clarkii* ovary development [25]. Geoducks have become a focus of significant aquaculture research and development with a considerable commercial value [26, 27]. The enriched gonad development-related pathways and genes could provide basic data for the further genetic breeding research of *P. generosa* and its closely related species.

We further compared gene families in different bivalves by searching for functional domains contained in PCGs in *P. generosa* and 8 other bivalves using InterProScan [28]. Examination of the top 65 domains of gene families that presented expansions in *P. generosa* (Figure 7A) showed that the gene numbers of many important gene families were substantially expanded in *P. generosa*, including these containing the GIY-YIG catalytic domain (PF01541), the caspase recruitment domain (PF16739), the ApoA/ApoE domain (PF01442), and the copine domain (PF07002). In particular, the copine gene family, which has been implicated in a range of cell signaling and cytoskeletal proteins, targeted to the membrane following increases in cellular calcium, has twice as many genes compared to other species. In *P. generosa*, the number of genes of the copine gene family was 22, comparing to 11 copine genes in *S. constricta*. The 22 copine genes in *P. generosa* were found in multiple chromosomes including *Pg02*, *Pg05*, *Pg07*, *Pg10*, *Pg11* and *Pg17* (Figure 7B). Interestingly, many genes formed local clusters, suggesting that the large copine gene set observed in *P. generosa* might have been achieved via tandem duplication of the copine

genes in evolution. For example, eight genes were located in a single cluster in *Pg11* (Figure 7B).

Phylogenetic analysis of the copine genes annotated in *P. generosa* (22 genes) and *S. constricta* (11 genes) revealed good orthologous relationships, as well as one-to-multiple relationships (Figure 7C), confirming that genes inside copine gene clusters in *P. generosa* (Figure 7B) were highly similar.

Most of the 22 copine genes in *P. generosa* contain the vWA-domain, while some has both C2-domain and the vWA-domain (Figure 7D) as for copine genes identified in other species [29]. Some copine genes were short, which might be due to imperfect annotation of these genes. Prediction of 3D structures of these candidate copine genes in *P. generosa* using AlphaFold2 [30] revealed that their structures are highly similar to those copine genes identified in other species. For example, the structure of *Pg02g00048* in *P. generosa* showed high similarity to that of *XP_0533888641* in *Mercenaria mercenaria* (Figure 7E).

Discussion

Through the construction of the first high-quality chromosome-level genome assembly of the ecologically and economically important bivalves the Pacific geoduck *P. generosa* with cutting-edge genomic technologies, important insights into its genetic makeup and evolution have been gained. The assembled genome size consists of 19 chromosomes with a genome size of 1.47 Gb, and a contig N50 of 1.6 Mb and 19 chromosomes. A total of 35,034 PCGs were predicted in the *P. generosa* genome, of which 30,700 genes (87.63%) were functionally annotated, supporting the high-quality annotation of the *P. generosa* genes, and the conservation of PCGs in bivalves. *P. generosa* is a highly

complex species with a heterozygosity of 1.37% and 57.99% repeat sequences in genome.

The availability of the first high-quality chromosome-level *P. generosa* genome assembly enabled us to ascertain its genomic compositions, which in turn facilitate comparative analysis with other bivalve genomes. The *P. generosa* genome represents the third genome of the third species in the order Adapedonta, after *S. constricta* and *Solen grandis*. Although all the three genomes have the exactly same number of 19 chromosomes, comparative analysis between the genomes of *P. generosa* and its most closely related species *S. constricta* revealed extensive inter- and intra-chromosomal exchanges. Large chromosomal fragments of *P. generosa Pg02* and *Pg11* matched to two *S. constricta* chromosomes (*Chr1* and *Chr10*) and two *S. constricta* chromosomes (*Chr1* and *Chr10*), respectively (Figure 2). It is rather surprising that such large inter-chromosomal exchanges did not lead to the change of chromosomal numbers of either *P. generosa* or *S. constricta*. Within chromosomes, the order of genes showed even more extensive alterations (Figure 2).

In addition to the changes of organization of chromosomes and order of genes in the *P. generosa* and its closely related species *S. constricta*, the numbers of PCG members in gene families with important functions also changes substantially (Figure 7). In particular, 22 copine genes were identified in *P. generosa*, representing twice those in *P. constricta*. Many of these 22 copine genes in *P. generosa* formed tandem clusters, with one cluster containing eight copine genes (Figure 7B). The copine genes within each cluster showed high similarity (Figure 7C), further confirming tandem duplications. Copine genes in *P. generosa* contain C2 domain and vWA-domain (Figure 7D). Modeling with AlphaFold2

revealed that copines encoded by the *P. generosa* copine genes show high structural similarity with that encoded by known copine genes (Figure 7E). Thus, comparative analysis of gene families uncovered important leads for exploring molecular insight into its physiology and evolution.

Taken together, this study provides the first high-quality genomic resource that will support future phylogenetic, evolution, and immunological studies. Additionally, it will support the genetic breeding of geoducks.

Methods

Sampling collection

Geoduck *P. generosa* samples were collected from the Strait of Georgia (49°41′12″N, 124°51′33″W) of British Columbia, Canada in the spring of 2019. The samples showed typical morphological features of *P. generosa*. The identification of the samples was also supported by the high similarities of the molecular marker *cox1* to the reference sequence of *P. generosa* (PID of 99.55, coverage of 100%) [31]. The samples- were transferred to laboratory and kept in a tank with running water for a week. One sample was chosen and dissected on ice to collect tissue samples, including labial palp, heart, foot, gonad, gill, hepatopancreas, siphon, and mantle muscle. This animal was identified to be a female as indicated by the presence of eggs in the smear of the gonad under a compound microscope. Dissected tissues were quickly frozen in liquid nitrogen and then stored at −80°C before DNA and RNA extraction.

DNA library construction and sequencing

Genomic DNA of *P. generosa* was extracted using a standard phenol-chloroform

extraction method [32]. The quality of DNA was determined by gel electrophoresis to ensure the DNA samples met library sequencing requirements. Sequence libraries with insert size of 300 bp were constructed for BGISEQ-500 sequencing platform (RRID:SCR_017979) according to manufacturer's protocol. The sequencing data produced were used in the genome size estimation by *k*-mer analysis [22] and for correcting errors in the Pilon (RRID:SCR_014731) assembly [33]. A Hi-C library with insert size of 300 bp was constructed to provide long-range information (without position information) on the grouping and linear organization of sequences along entire chromosomes to assemble the scaffolds into chromosome-level scaffolds [34]. For Hi-C library construction, gonad tissue was dissociated, and cells were collected and crosslinked with 1% formaldehyde (Sigma) and 0.2M glycine (Sigma). After that, the fixed powder was resuspended in nuclei isolation buffer and then incubated in 0.5% SDS for 10 min at 62°C. Then the reaction was quenched with 10% Triton X-100 (Sigma) and the nuclei were collected by centrifugation. Then the DNA was digested with MboI (NEB), and the overhang was filled and biotinylated before ligated by T4 DNA ligase (NEB). Before library construction, the purified DNA was sheared, and biotin-containing fragments were captured on streptavidin-coated beads using Dynabeads MyOne Streptavidin T1 (Invitrogen). The fragments were then end-repaired and linked with adaptors before eight cycles of PCR reaction with KAPA HiFi HotStart ReadyMix (Kapa Biosystem). After that, the Hi-C library was sequenced with BGISEQ-500 platform. And a PacBio library with insert size of 20 Kb was constructed to obtain long reads by the PacBio Sequel platform using the Sequel Sequencing Kit 3.0. The adapters and low-quality reads in raw data generated

by the BGISEQ platform were cut off by SOAPnuke1.5.6 using the parameter as "-n 0.01 -l 20 -q 0.1 -i -Q 2 -G -M 2 -A 0.5 -d" [35]. PacBio raw data were filtered with the default parameters by using Pacific Biosciences SMRT analysis software (v2.3.1) to filter the low quality reads.

RNA library construction and sequencing

RNA-Seq and Iso-Seq were conducted to obtain transcriptome data to aid genome annotation. The total RNAs was extracted by Trizol (Invitrogen, Carlsbad, CA, USA) from eight tissues of the same *P. generosa* individual, including labial palp, heart, foot, gonad, gill, hepatopancreas, siphon, and mantle muscle. The quality and quantity of RNA in each sample was assessed using a NanoDrop and an Agilent 2100 bioanalyzer (Thermo Fisher Scientific, MA, USA). The construction of mRNA libraries for RNA-Seq, the mRNA was enriched by mRNA Capture Beads (BGI, LB00V60), and incubated at 85°C for 8 minutes for fragmentation. Reverse transcription was performed with Strand Specificity Reagent and 1st Strand Enzyme Mix (Optimal Dual-mode mRNA Library Prep Kit, BGI, LR00R96) to generate the first strand cDNA. After that the second strand cDNA generation and end repair were performed with 2nd Strand Buffer and 2nd Strand Enzyme Master Mix. Then the adaptors (BGI, LA00R04) were ligated to the cDNAs. Then the library was purified and selected depending upon product requirements for amplification. The mRNA libraries were sequenced using the BGISEQ-500 platform. For Iso-Seq, the total RNA was extracted from the equally mixed tissues of the 8 tissues above. The PacBio SMRTbell library was prepared using the SMARTer PCR cDNA Synthesis kit (Clontech), the Qubit dsDNA HS Assay Kit 2.0 (Invitrogen) and the Agilent DNA 12000 kit (Agilent Technologies), and

sequenced by the PacBio Sequel sequencer (RRID:SCR_017989) with Sequel Sequencing Kit 3.0.

Genome size estimation and genome assembly

Genome size of *P. generosa* was estimated using *k*-mer analysis. Counting of *k*-mers was conducted using Jellyfish (RRID:SCR_005491, version 2.2.10) [22]. For genome assembly, long reads generated from PacBio Sequel platform were assembled using Falcon (RRID:SCR_016089) [36], which was subsequently polished using Arrow. Short paired-end clean reads from BGISEQ-500 were then used for correcting post-processing errors and resolving conflicts of assembly via Pilon (RRID:SCR_014731, version 1.22) [33]. The assembled contigs were corrected for mis-joins, orders, orients and anchored contigs from the draft assembly into a candidate chromosome-length assembly by Hi-C data using Juicer (RRID:SCR_017226) [37] and 3d-DNA [38]. The scaffolds shorter than 20 Kb were removed. Finally, the candidate assembly were reviewed with Juicerbox Assembly Tools (RRID:SCR_021172) for quality control and interactive corrections [39]. The Hi-C heatmap was visualized using Juicebox (RRID:SCR_021172) presenting the counts of paired reads which each two bins aligned (with the bin length of 100 kb) as the interactive signals between each pair of two bins. The completeness of genome assembly was assessed by BUSCO (RRID:SCR_015008, version5.4.3) [23]. using the metazoa_odb10 database. The genome landscape illustrating the length, repeat element density, gene density and GC content was created by circos-0.69-9 (RRID:SCR_011798) [40].

Annotations of gene structure and function

Homologous and *de novo* predictions were both applied to annotate transposable

elements in the *P. generosa* genome. In homologous prediction, RepeatMasker (RRID:SCR_012954) and RepeatProteinMask [41] were used to screen the *P. generosa* genome for known transposable elements in the RepBase library (RRID:SCR_021169) [42]. In *de novo* prediction, RepeatModeler (version 1.0.4) was first used for de novo candidate database construction of repetitive elements, and repetitive sequences were then annotated using RepeatMasker. Tandem repeats were de novo predicted using Tandem repeats finder (version 4.07) [43]. The results were then integrated and duplicates were eliminated.

Three complementary approaches were adopted to predict PCGs in *P. generosa* genome, including homology-based prediction, *de novo* annotation, and transcriptome-based prediction. For homology-based prediction, gene sets from eight closely related bivalves (*P. yessoensis, P. fucata, Mytilus galloprovincialis, Limnoperna fortune, A. purpuratus, S. constricta, S. broughtonii, and C. gigas*) were used. First, protein repertoires of those organisms were aligned against the *P. generosa* genome using TBLASTN (RRID:SCR_011822) [44]. Then gene structures were predicted from these blast hits by Exonerate v2.2.0 [45]. *de novo* gene prediction was performed using a combination of Augustus (RRID:SCR_008417) [46] and SNAP (RRID:SCR_007936) [47] with default settings. For transcriptome-based prediction using RNA-Seq data, RNA-Seq reads were directly mapped to the genome using TopHat2 (RRID:SCR_013035) [48]. The mapped reads were subsequently assembled into gene models (Cufflinks-set) by Cufflinks (RRID:SCR_014597) [49]. For transcriptome-based prediction based on Iso-Seq data, Iso-Seq reads were directly mapped to the genome using GMAP (RRID:SCR_008992) [50].

The mapped reads were subsequently assembled by PASA (RRID:SCR_014656) [51]. Gene predictions from the homology-based approach, *de novo* approach, RNA-Seq-based and Iso-Seq-based evidences were merged, and redundancy was removed to form a comprehensive consensus gene set using Maker 2 (RRID:SCR_005309) [52]. To validate the completeness of the gene structure annotation, we also used BUSCO (version5.4.3) with the metazoa_odb10 database [23].

Phylogenetic analysis and divergence time estimation

Gene families were constructed using the OrthoMCL (RRID:SCR_007839) pipeline [53]. We selected *P. generosa* and other 11 species (*C. gigas*, *P. yessoensis*, *P. maximus*, *A. purpuratus*, *S. broughtonii*, *P. martensi*, *B. platifrons*, *H. sapiens*, *X. tropicaalis*, *D. rerio*, and *C. elegans*) for gene family analysis. For the gene set of each genome, only the transcript with the longest coding sequence was selected from alternate splice transcripts. Genes with less than 50 amino acids were removed from further analysis. Protein sequences were aligned by “all-vs-all BLASTP” (E value = $1e^{-5}$) [44]. Then the Markov clustering (MCL) algorithm implemented in OrthoMCL was used to group orthologues and paralogues from all input species with an inflation value of 1.5 [53].

The phylogenetic tree was constructed following procedures described previous studies [13, 54-55]. Briefly, for phylogenetic tree construction and divergence time estimation, shared single copy genes of *P. generosa* and 11 other species were used. The protein sequences of single-copy orthologs among the 12 species were aligned using MUSCLE v3.7 (RRID:SCR_011812) [56] with default parameters. Phylogenetic relationships were inferred based on the super-matrix estimated from the concatenated

alignment of single-copy genes using the maximum likelihood (ML) [57] method implemented in RAxML v2.2 (RRID:SCR_006086) [58] with the optimal amino acid substitution model selected by the PROTGAMMALGX parameter.

Based on gene family identification and phylogenetic analysis, single copy genes and mcmctree in PAML [59] were used to estimate divergence time [60-63]. The time correction points were *C. elegans* and *H. sapiens* (678.3–855.2 MYA), *D. rerio* and *H. sapiens* (413.1–443.0 MYA), *X. tropicalis* and *H. sapiens* (347.0–357.9 MYA). The time correction points were taken from the Timetree website. The operating parameters of mcmctree: burn in = 10000, sample number = 1000000, sample frequency = 50.

Gene family expansion and contraction

The clustering results of gene families and the phylogenetic tree with divergence time estimated were used to analyze the expansion and contraction of orthologous gene families between ancestor and each of the 12 species (*P. generosa* and the other 11 species) using a stochastic birth and death model with lambda parameter by CAFE (RRID:SCR_005983, version 4.0) [64]. This model was further used to calculate the number of gene families along each lineage on the phylogenetic tree. A probabilistic graphical model was introduced to calculate the probability of transitions in gene family size from parent to child nodes. The family-wide P-Values were calculated in each lineage based on the conditional likelihood.

Data Availability

The Whole Genome project of *P. generosa* has been deposited at NCBI/BioProject PRJNA859289. The raw next-generation sequencing reads of DNA are available at SRA

(SRR22190027-SRR22190030); raw long-read PacBio sequencing reads of DNA are available at SRA (SRR22190026; raw next-generation sequencing reads of RNA are available at SRA (SRR22190032); raw Hi-C reads are available at SRA (SRR22190025); and raw long-read PacBio sequencing reads of RNA are available at SRA(SRR22190031). The genome assembly data have been deposited under accession No. JAPMAH000000000.1.

Additional Files

Supplementary Figure S1: The 17-mer count distribution for the genome size estimation.

Supplementary Figure S2: The enriched KEGG pathways of significantly expanded gene families (p≤0.01, top 20) in *P. generosa*

Supplementary Table S1: The genome assembly information of bivalves in the public database

Supplementary Table S2: Statistics of 17-mer analysis

Supplementary Table S3: The heterozygosity of bivalves reported

Supplementary Table S4: The chromosomes information of *P. generosa*

Supplementary Table S5: The structural statistics of gene prediction in *P. generosa*

Supplementary Table S6: The assembly quality of chromosomal genomes of bivalves by BUSCO

Supplementary Table S7: Functional annotation of the predicted protein-coding genes in *P. generosa* genome assembly

Supplementary Table S8: The information of 30,616 gene families of *P. generosa* and 11

other species

Supplementary Table S9: The enriched KEGG pathways of expanded gene families in *P. generosa* genome assembly

Abbreviations

PCG: protein-coding gene; Akt: RAC serine/threonine-protein kinase; bp: base pairs; BLAST: Basic Local Alignment Search Tool; BUSCO: Benchmarking Universal Single-Copy Orthologs; BWA: Burrows-Wheeler Aligner; cAMP: cyclic adenosine monophosphate; CAMs: cell adhesion molecules; cGMP-PKG: cGMP-dependent protein kinase G; Gb: gigabase pairs; GC: guanine-cytosine; GnRH: Gonadotropin-releasing hormone; GO: gene ontology; Hi-C: High-throughput/resolution chromosome conformation capture; kb: kilobase pairs; KEGG: Kyoto Encyclopedia of Genes and Genomes; Mb: megabase pairs; MYA: million years ago; NCBI: National Center for Biotechnology Information; NF-kappa B: nuclear factor kappa-B; NOD-like receptor: nucleotide-binding oligomerization domain-like receptor; PacBio: Pacific Biosciences; RAxML: Randomized Axelerated Maximum Likelihood; PI3K: phosphatidylinositol-4,5-bisphosphate 3-kinase catalytic subunit alpha/beta/delta; Rap1: Ras-related protein1; RNA-Seq: RNA sequencing; Iso-Seq: Isoform-sequencing; tRNA: transfer RNA; TRP channel: transient receptor potential ion channel.

Competing Interests

The authors declare that they have no competing interests.

Funding

This study was supported by the Taishan Scholar Project Special Fund (to Nansheng Chen), the Strategic Priority Research Program of Chinese Academy of Sciences (XDB42000000), the Chinese Academy of Sciences Pioneer Hundred Talents Program (to Nansheng Chen), and an Earmarked Workstation Fund for QRJH (to Chunde Wang and Nansheng Chen).

Authors' Contributions

N.C. and C.W. conceived and designed the study. M.C. and Y.C. prepared the samples. J.W. and Q.X. performed analyses. J.W. wrote the paper with input from co-authors. All authors read and approved the final version for submission.

References

1. González-Peláez SS, Leyva-Valencia I, Pérez-Valencia SA, et al. Distribution limits of the geoduck clams *Panopea generosa* and *P. globosa* on the Pacific coast of Mexico. *Malacologia*. 2013;56:85-94.
2. Vadopalas B, Pietsch TW, Friedman CS. The proper name for the geoduck: resurrection of *Panopea generosa* Gould, 1850, from the synonymy of *Panopea abrupta* (Conrad, 1849) (Bivalvia: Myoida: Hiatellidae). *Malacologia*. 2010;52:169-173.
3. Goodwin CL, Pease BC. Geoduck, *Panopea abrupta* (Conrad, 1849), size, density, and quality as related to various environmental parameters in Puget Sound, Washington. *J Shellfish Res.* 1991;10:65-77.
4. Orensanz JM, Hand CM, Parma AM, et al. Precaution in the harvest of Methuselah's clams the difficulty of getting timely feedback from slow-paced dynamics. *Can J Fish Aquat Sci*. 2004;61:1355-1372.
5. Newell RIE. Ecosystem influences of natural and cultivated populations of suspension-feeding bivalve molluscs: A review. *J Shellfish Res.* 2004;23:51-61.
6. Straus KM, MacDonald PS, Crosson LM, et al. Effects of geoduck aquaculture on the environment: A synthesis of current knowledge., Washington Sea Grant Technical Report WSG-TR 13-02, 2013.
7. Santos A, Aguirre J, Rodríguez-Tovar FJ, et al. Multi-storm events recorded on *Panopea* burrows (Pliocene, Spain): The importance of sequestered information inside burrows. *Palaeogeogr Palaeoclimatol Palaeoecol*. 2018;507:155-167.
8. Bureau D., Hajas W., Hand C.M., et al. Age, size structure and growth parameters of geoducks (*Panopea abrupta*, Conrad 1849) from seven locations in British Columbia sampled in 2001 and 2002. Canadian Technical Report of Fisheries and Aquatic Sciences, 2003, p. 29.
9. Sloan NA, Robinson SMC. Age and gonad development in the geoduck clam *Panopea abrupta* (Conrad) from southern British Columbia, Canada. *J Shellfish Res.* 1984;4:131-137.
10. Valero JL, Canada O, Madryn P, et al. Geoduck (*Panopea abrupta*) recruitment in the Pacific Northwest: long-term changes in relation to climate. *CalCOFI Reports*. 2004;45:80-86.
11. Thai BT, Lee YP, Gan HM, et al. Whole genome assembly of the snout otter clam, *Lutraria rhynchaena*, using Nanopore and Illumina Data, benchmarked against bivalve genome assemblies. *Front Genet.* 2019;10:1158.
12. Sun J, Zhang Y, Xu T, et al. Adaptation to deep-sea chemosynthetic environments as revealed by mussel genomes. *Nat Ecol Evol*. 2017;1:0121.
13. Ran Z, Li Z, Yan X, et al. Chromosome-level genome assembly of the razor clam *Sinonovacula constricta* (Lamarck, 1818). *Mol Ecol Resour.* 2019;19:1647-1658.
14. Halanych KM, Kocot KM. Genome evolution: Shellfish genes. *Nat Ecol Evol.* 2017;1:0142.
15. Peñaloza C, Gutierrez AP, Eöry L, et al. A chromosome-level genome assembly for the Pacific oyster *Crassostrea gigas*. *GigaScience*. 2021;10:giab020.
16. Peng J, Li Q, Xu L, et al. Chromosome-level analysis of the *Crassostrea hongkongensis*

genome reveals extensive duplication of immune-related genes in bivalves. *Mol Ecol Resour.* 2020;20:980-994.

17. Wu B, Chen X, Yu M, et al. Chromosome-level genome and population genomic analysis provide insights into the evolution and environmental adaptation of Jinjiang oyster *Crassostrea ariakensis*. *Mol Ecol Resour.* 2022;22:1529-1544.
18. Du X, Fan G, Jiao Y, et al. The pearl oyster *Pinctada fucata* martensii genome and multi-omic analyses provide insights into biomineralization. *GigaScience*. 2017;6:1-12.
19. Yang JL, Feng DD, Liu J, et al. Chromosome-level genome assembly of the hard-shelled mussel *Mytilus coruscus*, a widely distributed species from the temperate areas of East Asia. *GigaScience*. 2021;10:giab024.
20. Holmes A, Darbyshire T, Brennan M, et al. The genome sequence of *Gari tellinella* (Lamarck, 1818), a sunset clam. *Wellcome Open Res*. 2022;7:116.
21. Zhang T, Yin J, Tang S, et al. Dissecting the chromosome-level genome of the Asian Clam (*Corbicula fluminea*). *Sci Rep*. 2021;11:15021.
22. Marçais G, Kingsford C. A fast, lock-free approach for efficient parallel counting of occurrences of *k*-mers. *Bioinformatics*. 2011;27:764-770.
23. Simão FA, Waterhouse RM, Ioannidis P, et al. BUSCO: assessing genome assembly and annotation completeness with single-copy orthologs. *Bioinformatics*. 2015;31:3210-2.
24. Li J, Zhou Y, Zhou Z, et al. Comparative transcriptome analysis of three gonadal development stages reveals potential genes involved in gametogenesis of the fluted giant clam (Tridacna squamosa). *BMC Genom*. 2020;21:872.
25. Jiang H, Liu H, Ma X, et al. Transcriptome analysis of Procambarus clarkii to screen genes related to ovary development,immunity and growth. *J Fish China*. 2021;45:396-414.
26. Ren Y, Liu W, Pearce CM, et al. Effects of selected mixed-algal diets on growth and survival of early postset juveniles of the Pacific geoduck clam, *Panopea generosa* (Gould, 1850). *Aquac Nutr.* 2014;21.
27. Nava-Gómez GE, Garcia-Esquivel Z, Carpizo-Ituarte E, et al. Survival and growth of geoduck clam larvae (*Panopea generosa*) in flow-through culture tanks under laboratory conditions. *Aquac Res*. 2018;49:294-300.
28. Zdobnov EM, Apweiler R. InterProScan--an integration platform for the signature-recognition methods in InterPro. *Bioinformatics*. 2001;17:847-8.
29. Perestenko PV, Pooler AM, Noorbakhshnia M, et al. Copines-1, -2, -3, -6 and -7 show different calcium-dependent intracellular membrane translocation and targeting. *FEBS J*. 2010;277:5174-5189.
30. Jumper J, Evans R, Pritzel A, et al. Highly accurate protein structure prediction with AlphaFold. *Nature*. 2021;596:583-589.
31. Bisbal-Pardo CI, Del Río-Portilla MA, Rocha-Olivares A. The complete mitochondrial DNA of the Pacific Geoduck clam (*Panopea generosa*). *Mitochondrial DNA A*. 2016;27:1955-6.
32. Green M, J S. Molecular cloning: a laboratory manual. 4th Edn. Vol. II. New York, NK: Cold Spring Harbor Laboratory Press.
33. Walker BJ, Abeel T, Shea T, et al. Pilon: an integrated tool for comprehensive microbial

variant detection and genome assembly improvement. *PloS ONE*. 2014;9:e112963.
34. Burton JN, Adey A, Patwardhan RP, et al. Chromosome-scale scaffolding of de novo genome assemblies based on chromatin interactions. *Nat Biotechnol*. 2013;31:1119-25.
35. Chen Y, Chen Y, Shi C, et al. SOAPnuke: a MapReduce acceleration-supported software for integrated quality control and preprocessing of high-throughput sequencing data. *GigaScience*. 2018;7:1-6.
36. Pendleton M, Sebra R, Pang AW, et al. Assembly and diploid architecture of an individual human genome via single-molecule technologies. *Nat Methods*. 2015;12:780-6.
37. Durand NC, Shamim MS, Machol I, et al. Juicer provides a one-click system for analyzing loop-resolution Hi-C experiments. *Cell Syst*. 2016;3:95-8.
38. Dudchenko O, Batra SS, Omer AD, et al. De novo assembly of the *Aedes aegypti* genome using Hi-C yields chromosome-length scaffolds. *Science*. 2017;356:92-95.
39. Robinson JT, Turner D, Durand NC, et al. Juicebox.js provides a cloud-based visualization system for Hi-C data. *Cell Syst*. 2018;6:256-258.
40. Krzywinski M, Schein J, Birol I, et al. Circos: an information aesthetic for comparative genomics. *Genome Res*. 2009;19:1639-45.
41. Bergman CM, Quesneville H. Discovering and detecting transposable elements in genome sequences. *Brief Bioinform*. 2007;8:382-92.
42. Bao W, Kojima KK, Kohany O. Repbase Update, a database of repetitive elements in eukaryotic genomes. *Mob DNA*. 2015;6:11.
43. Benson G. Tandem repeats finder: a program to analyze DNA sequences. *Nucleic Acids Res.* 1999;27:573-80.
44. Altschul SF, Gish W, Miller W, et al. Basic local alignment search tool. *J Mol Biol*. 1990;215:403-10.
45. Slater GS, Birney E. Automated generation of heuristics for biological sequence comparison. *BMC Bioinform*. 2005;6:31.
46. Stanke M, Keller O, Gunduz I, et al. AUGUSTUS: ab initio prediction of alternative transcripts. *Nucleic Acids Res.* 2006;34:W435-9.
47. Johnson AD, Handsaker RE, Pulit SL, et al. SNAP: a web-based tool for identification and annotation of proxy SNPs using HapMap. *Bioinformatics*. 2008;24:2938-9.
48. Kim D, Pertea G, Trapnell C, et al. TopHat2: accurate alignment of transcriptomes in the presence of insertions, deletions and gene fusions. *Genome biology*. 2013;14:R36.
49. Trapnell C, Roberts A, Goff L, et al. Differential gene and transcript expression analysis of RNA-seq experiments with TopHat and Cufflinks. *Nat Protoc*. 2012;7:562-78.
50. Wu TD, Watanabe CK. GMAP: a genomic mapping and alignment program for mRNA and EST sequences. *Bioinformatics*. 2005;21:1859-75.
51. Haas BJ, Delcher AL, Mount SM, et al. Improving the *Arabidopsis* genome annotation using maximal transcript alignment assemblies. *Nucleic Acids Res.* 2003;31:5654-66.
52. Holt C, Yandell M. MAKER2: an annotation pipeline and genome-database management tool for second-generation genome projects. *BMC Bioinform*. 2011;12:491.
53. Li L, Stoeckert CJ, Jr., Roos DS. OrthoMCL: identification of ortholog groups for

eukaryotic genomes. *Genome Res*. 2003;13:2178-89.
54. Song H, Guo X, Sun L, et al. The hard clam genome reveals massive expansion and diversification of inhibitors of apoptosis in Bivalvia. BMC Biol. 2021 Jan 25;19(1):15.
55. Tian HF, Hu QM, Li Z. A high-quality de novo genome assembly of one swamp eel (*Monopterus albus*) strain with PacBio and Hi-C sequencing data. *G3: Genes Genom Genet*. 2021 Jan 18;11(1):jkaa032.
56. Edgar RC. MUSCLE: multiple sequence alignment with high accuracy and high throughput. *Nucleic Acids Res.* 2004;32:1792-7.
57. Guindon S, Gascuel O. A simple, fast, and accurate algorithm to estimate large phylogenies by maximum likelihood. *Syst Biol.* 2003;52:696-704.
58. Stamatakis A. RAxML-VI-HPC: maximum likelihood-based phylogenetic analyses with thousands of taxa and mixed models. *Bioinformatics*. 2006;22:2688-90.
59. Yang Z. PAML 4: phylogenetic analysis by maximum likelihood. *Mol Biol Evol.* 2007;24:1586-91.
60. Thorne JL, Kishino H, Painter IS. Estimating the rate of evolution of the rate of molecular evolution. *Mol Biol Evol.* 1998;15:1647-57.
61. Vogel JP, Garvin DF, Mockler TC, et al. Genome sequencing and analysis of the model grass Brachypodium distachyon. *Nature*. 2010;463:763-8.
62. Blanc G, Wolfe KH. Widespread paleopolyploidy in model plant species inferred from age distributions of duplicate genes. *The Plant cell*. 2004;16:1667-78.
63. Sanderson MJ. r8s: inferring absolute rates of molecular evolution and divergence times in the absence of a molecular clock. *Bioinformatics*. 2003;19:301-2.
64. Han MV, Thomas GW, Lugo-Martinez J, et al. Estimating gene gain and loss rates in the presence of error in genome assembly and annotation using CAFE 3. *Mol Biol Evol.* 2013;30:1987-97.

Figure 1: The *P. generosa* genome contig contact matrix using Hi-C data and landscape. (A) Hi-C analysis of *P. generosa* genome contigs. Chromosomes are arranged in the size order from left to right and from top to bottom. The color bar illuminates the logarithm of the contact density from red (10) to white (0) in the plot. (B) The genomic landscape of *P. generosa*: from outer to inner circles: a, the 19 chromosomes; b–d, repetitive element density, gene density, and GC density across the genome, respectively, drawn in 1 Mb non-overlapping windows.

Figure 2: Chromosome synteny of *P. generosa* and *S. constricta*

Figure 3: The distribution of single-copy orthologs, multiple-copy orthologs, unique paralogs, other orthologs, and unclustered genes in *P. generosa* and related species.

Figure 4: Distribution of shared gene families among *P. generosa*, *P. martensi*, *S. broughtonii*, and *P. yessoensis*. Intersections between species indicate the numbers of shared gene families, whereas unique family numbers are shown in species-specific areas. The center represents the number of families shared by all the 4 species.

Figure 5: Phylogenetic analysis of *P. generosa* with related species. The estimated species divergence time (million years ago) and the 95% confidential intervals are labeled at each branch site. Divergence times used for time recalibration is illuminated as red dots in the tree.

Figure 6: Dynamic evolution and distribution of gene families among *P. generosa* and related species. The numbers of gene gains (+) and losses (−) are shown on the branches, which are also displayed as pie plots: the green part for gene gain, the red part for gene losses and the blue part for gene remained. The divergence times are dated and displayed below the phylogenetic tree. MRCA: most recent common ancestor.

Figure 7. Comparative analysis of gene families based on Pfam annotation in *P. generosa* and related bivalves. A. The 65 most abundant gene families that *P. generosa* possessing more gene numbers than 8 other bivalves. B. The phylogenetic tree of genes annotated to PF07002 domains in *P. generosa* and *S. constricta*, shown in the background colors of red and pink, respectively. C. Chromosomal distribution of copine genes predicted in *P. generosa genome*. D. Domain structures of copine genes predicted in *P. generosa* genome. E. The 3D structures of *Pg02g00048* gene in *P. generosa* and *XP_0533888641* gene in *Mercenaria mercenaria*.

Table Click here to access/download;Table;Tables.docx

Table 1: Statistics of the DNA sequence data used for *P. generosa* genome assembly

Source	Platform	Library size	Clean data (Gb)	Read length (bp)	Sequencing coverage (x)
**Genome-short reads**	BGISEQ-500	300 bp	258.19	150	181
**Genome-long reads**	PacBio sequel	20 Kb	164.46	26,513*	115
**Hi-C**	BGISEQ-500	300 bp	233.49	150	163

[a]"26,513*" indicated the N50 of subreads.

Table 2: Statistics of the genome assembly of *P. generosa*

Statistics	Contig	Scaffold	Chromosome
**Total Number (#)**	2,086	39	19
**Total length (bp)**	1,473,137,789	1,474,161,289	1,432,060,667
**Average Length (bp)**	706,202	37,799,007	75,371,614
**N50 Length (bp)**	1,571,249	73,788,920	76,670,739
**N90 Length (bp)**	418,579	53,843,121	53,843,121
**Maximum Length (bp)**	6,469,558	101,196,518	101,196,518
**Minimum Length (bp)**	17	29,000	1,432,060,667
**GC content**	34.33%	34.33%	34.33%
**Anchored rate (%)**		94.70%	

Table 3: Repetitive element annotations in *P. generosa*

Repetitive sequence			Transposable elements		
**Type**	**Length (bp)**	**% in genome**	**Type**	**Length (bp)**	**% in genome**
**DNA**	318426608	21.6	**DNA**	318426608	21.6
**LINE**	134171392	9.1	**LINE**	134171392	9.1
**SINE**	27249093	1.85	**SINE**	27249093	1.85
**LTR**	55380608	3.76	**LTR**	55380608	3.76
**Other**	31141	0	**Other**	31141	0
**Unknown**	151891763	10.3	**Unknown**	151891763	10.3
**Tandem repeat**	347641207	23.58	**Total**	583000390	39.55
**Total**	854873570	57.99			

Table 4: BUSCO results for analysis of genome completeness for *P. generosa*

Type	Genome assembly		Gene set	
	Number of genes	Percentage (%)	Number of genes	Percentage (%)
**Complete BUSCOs (C)**	887	93.0	844	88.4
**Complete and single-copy BUSCOs (S)**	842	88.3	795	83.3
**Complete and duplicated BUSCOs (D)**	45	4.7	49	5.1
**Fragmented BUSCOs (F)**	34	3.6	49	5.1
**Missing BUSCOs (M)**	33	3.4	61	6.5
**Total BUSCO groups**	954	100	954	100

Table 5: The PCGs of *P. generosa* and 11 other species for evolutionary analysis

Species	Total genes	Unclustered genes	Families	Unique families	Ave. genes per family
P. generosa	35,034	6,168	12,034	1,749	2.4
A. purpuratus	26,256	3,720	13,196	290	1.71
B. platifrons	33,584	3,197	12,409	1,775	2.45
C. gigas	28,402	3,638	11,818	828	2.1
D. rerio	25,444	1,791	9,210	295	2.57
H. sapiens	20,229	1,488	9,251	226	2.03
P. yessoensis	24,521	1,704	13,017	137	1.75
P. maximus	26,152	1,518	13,276	164	1.86
P. martensi	25,526	2,403	11,043	318	2.09
S. broughtonii	24,045	2,770	11,314	538	1.88
X. tropicalis	19,967	1,016	9,226	159	2.05
C. elegans	33,552	5,600	8,201	3,720	3.41

Figure 1

Click here to access/download;Figure;Figure1.pdf

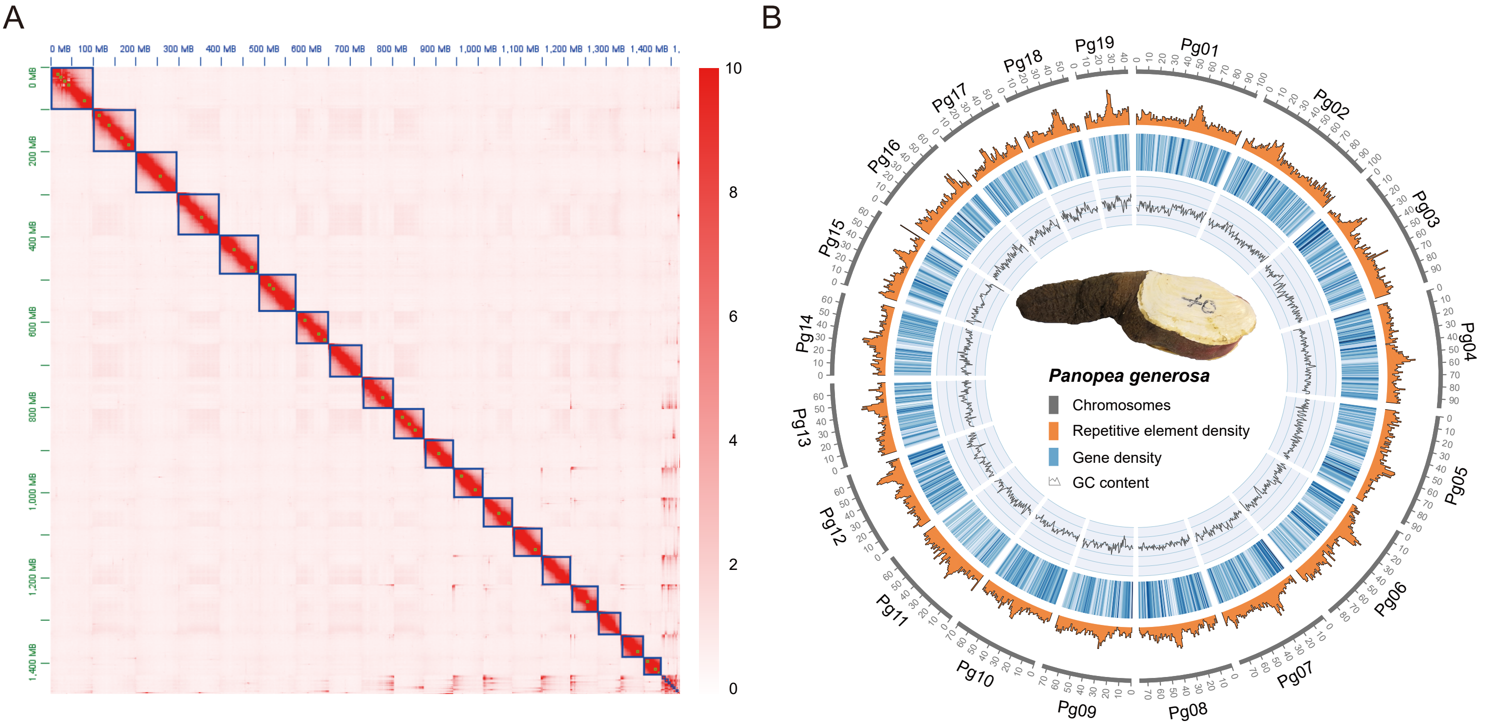

Figure 2

Click here to access/download;Figure;Figure2.pdf

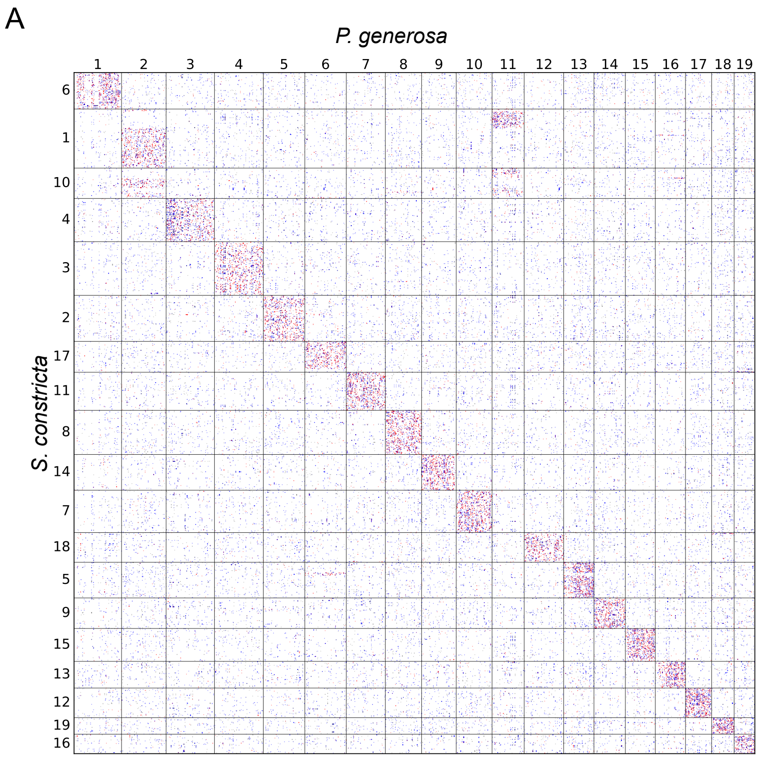

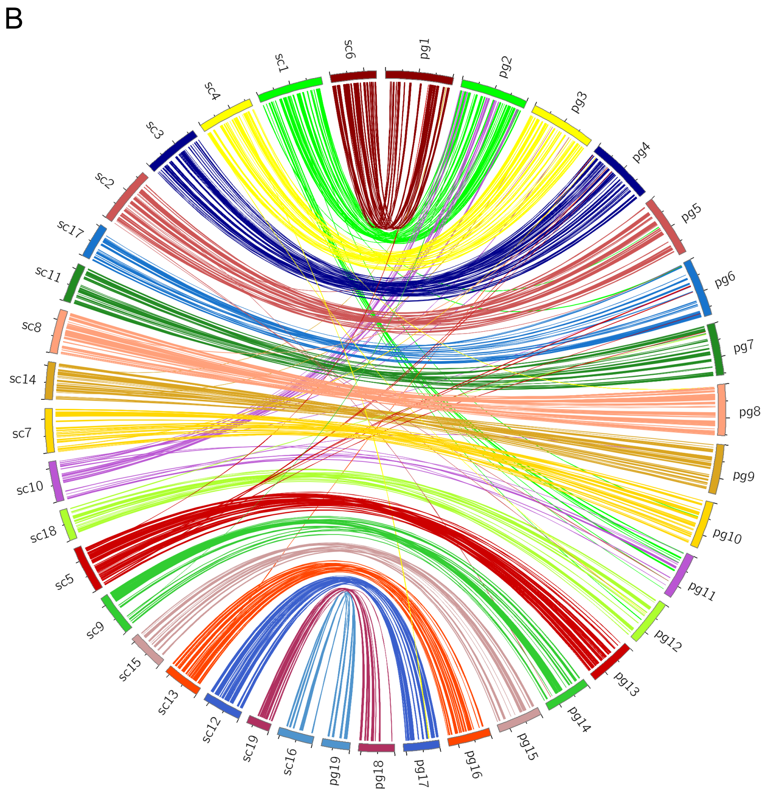

Figure 3

Click here to access/download;Figure;Figure3.pdf

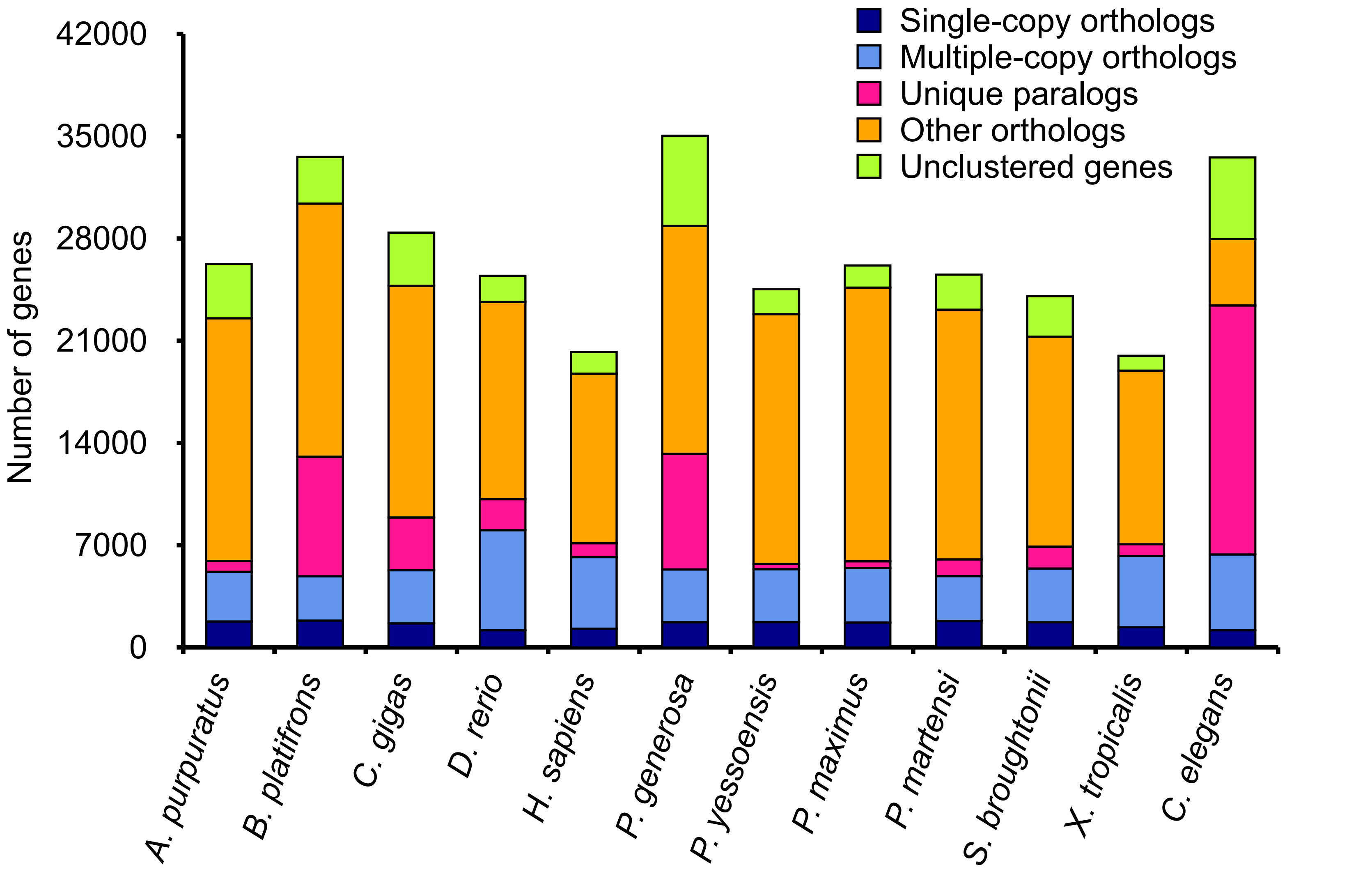

Figure 4

Click here to access/download;Figure;Figure4.pdf

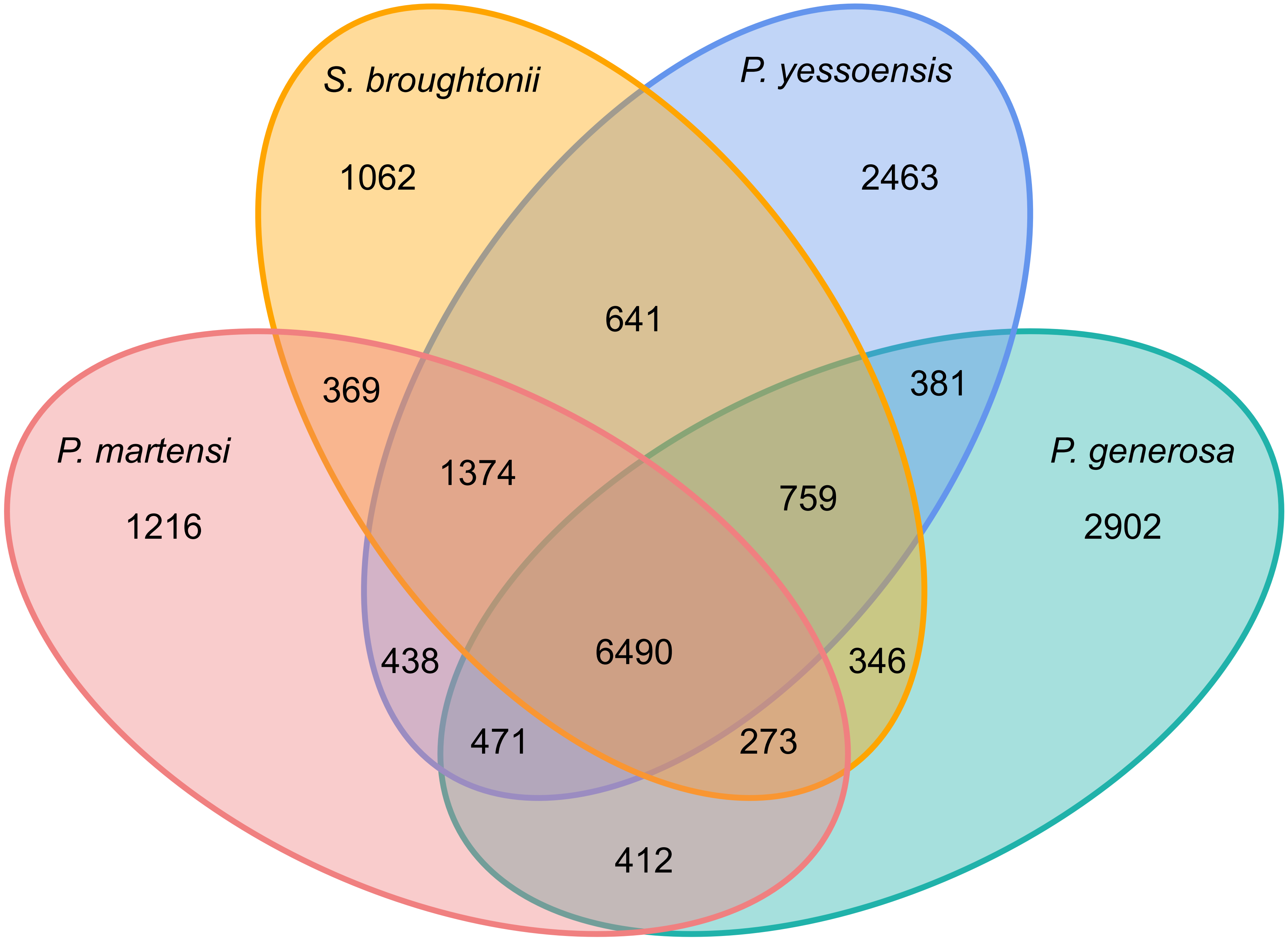

Number of gene families

Figure 5

Click here to access/download;Figure;Figure5.pdf

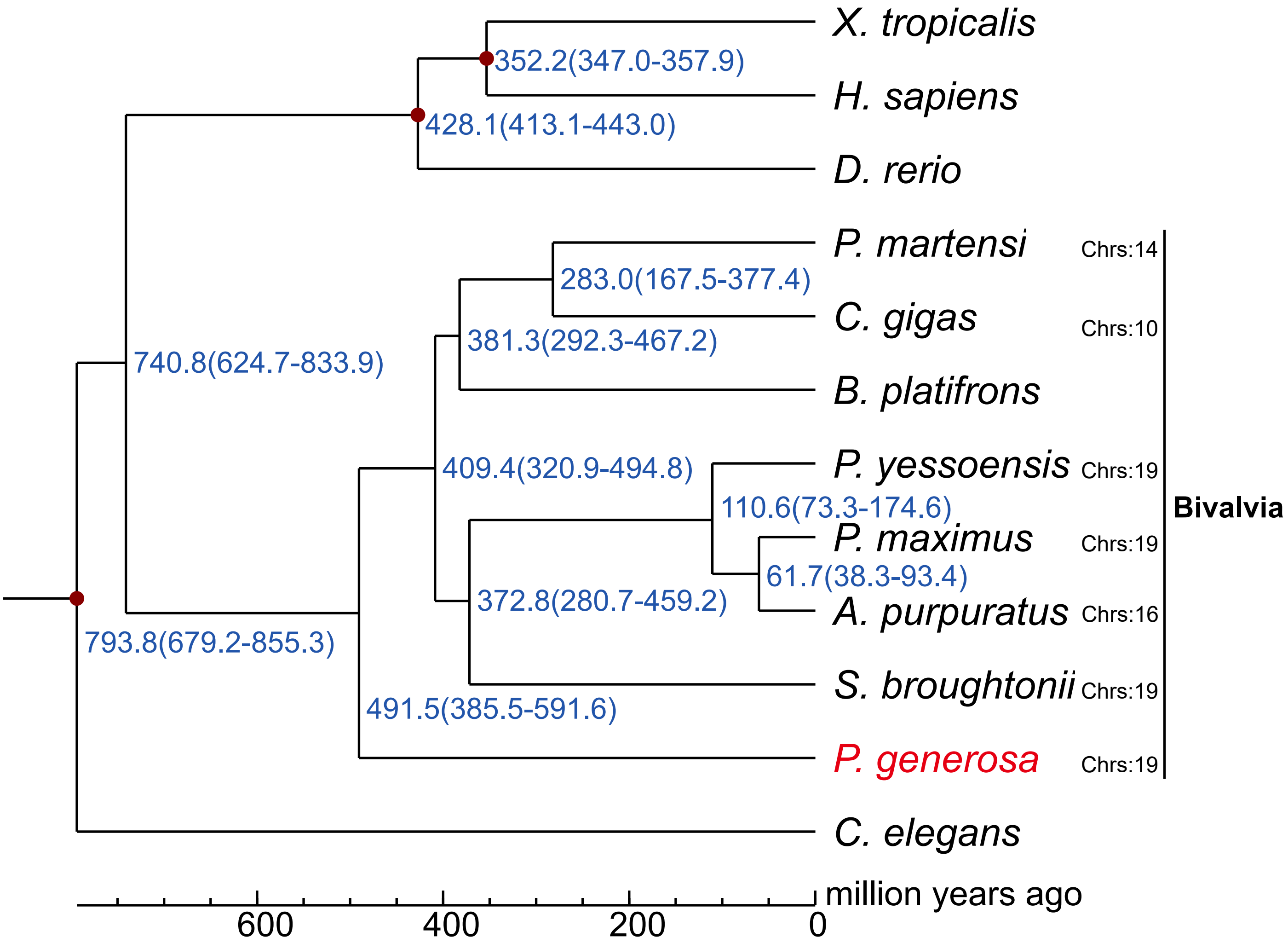

Figure 6 Click here to access/download;Figure;Figure6.pdf

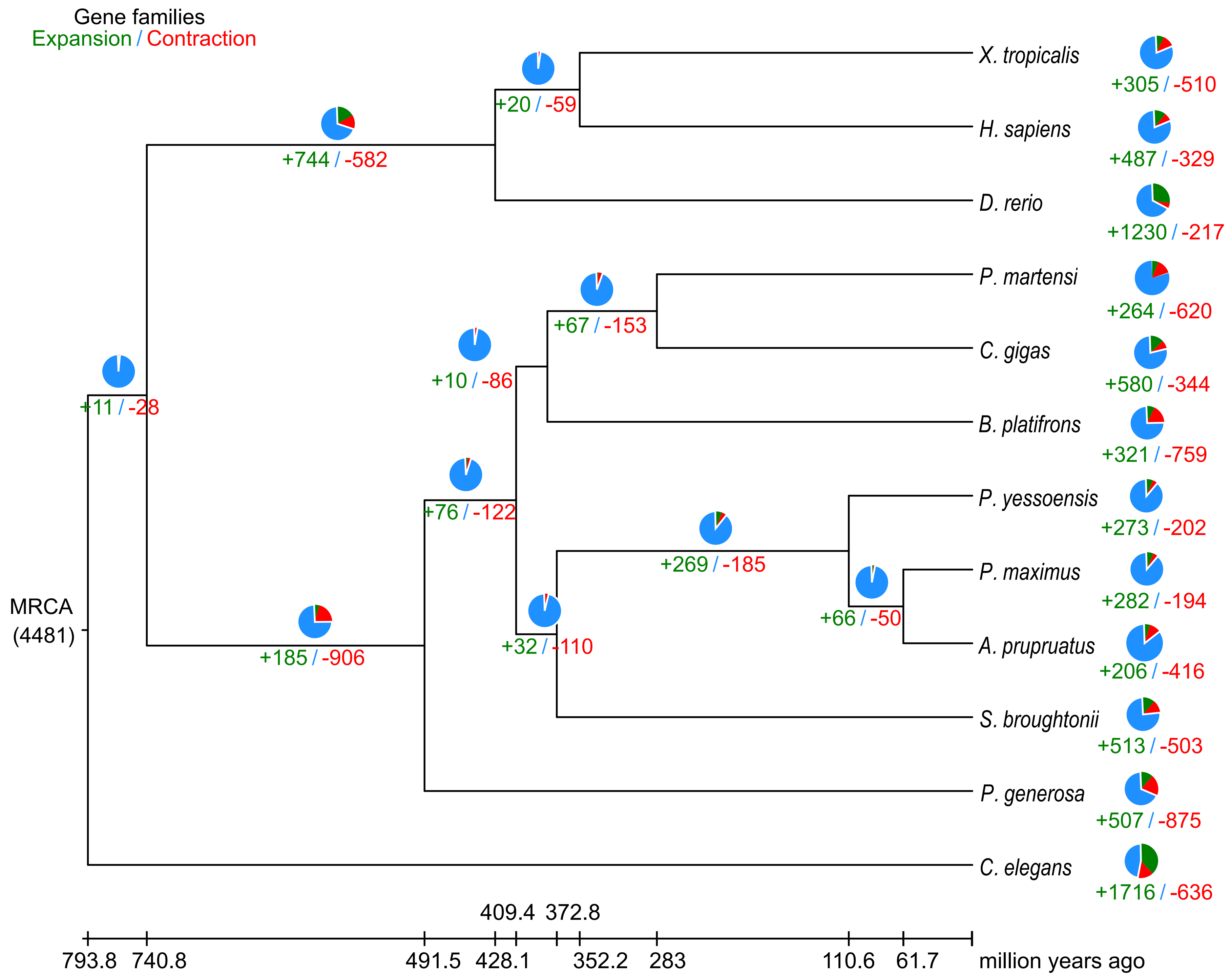

Figure 7 Click here to access/download;Figure;Figure7.pdf

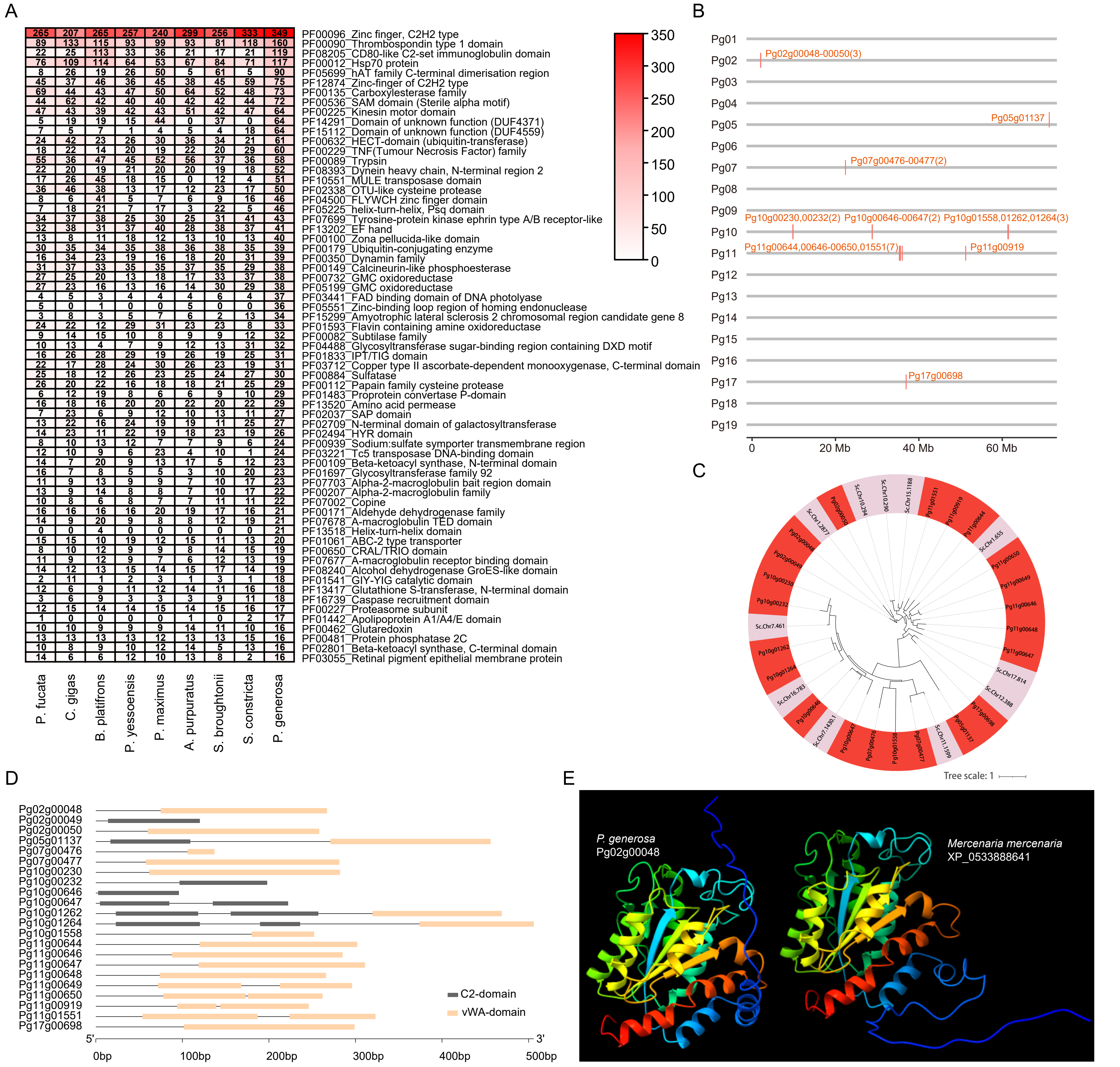

Supplementary Figure S1-S2; Table S2-S7

Click here to access/download
Supplementary Material
Supplementary Information.docx

Supplementary Table S1

Click here to access/download
Supplementary Material
Supplementary Information-TABLE S1.xlsx

Supplementary Table S8

Click here to access/download
Supplementary Material
Supplementary Information-TABLE S8.xlsx

Supplementary Table S9

Click here to access/download
Supplementary Material
Supplementary Information-TABLE S9.xlsx

Cover letter
Click here to access/download;Personal Cover;cover letter.docx

Dear Editors,

I would like to thank you for sending our manuscript (Manuscript number: GIGA-D-22-00284) for peer review and would also like to take advantage of this opportunity to thank the reviewers for their constructive comments and valuable suggestions. We have now addressed all concerns raised by the reviewers. The point-to-point replies to questions shown in the 'Response to Reviewers' box in the submission system.

The following is a summary of major revisions:

1. We have strengthened analysis on chromosomal synteny analysis and substantially gene family expansion of *Panopea generosa*, which uncovered surprising results including major inter- and intra-chromosomal exchanges, as well as substantial expansion of the coping gene family. We have thus added two figures illustrating these new results.

2. We have rephrased the title as “Chromosome-level genome assembly of the Pacific geoduck *Panopea generosa* reveals major inter- and intra-chromosomal rearrangements and substantial expansion of the copine gene family” to reflect the changes of the manuscript.

2. We have reformatted the manuscript according to the format of Data Note in the most recent issues of GigaScience.

We hope that the revised manuscript is now acceptable for publication in GigaScience, and we look forward to hearing from you.

Best wishes,

Nansheng Chen

Key Laboratory of Marine Ecology and Environmental Sciences
Institute of Oceanology, Chinese Academy of Sciences
Add: 7 Nanhai Road, Qingdao 266071, China
Tel: +86-532-82893507
E-mail: chenn@qdio.ac.cn
