# Supplementary material for: Chromosome-level genome assembly of the Pacific geoduck Panopea generosa reveals major inter- and intrachromosomal rearrangements and substantial expansion of the copine gene family
Source: Gigascience. 2023 Dec 19;12:giad105. doi: 10.1093/gigascience/giad105 (PMC10729735; doi:10.1093/gigascience/giad105)
Supplement: giad105_GIGA-D-22-00284_Revision_2 [file giad105_giga-d-22-00284_revision_2.pdf]

GigaScience

Chromosome-level genome assembly of the Pacific geoduck Panopea generosa reveals major inter- and intra-chromosomal rearrangements and substantial expansion of the copine gene family

--Manuscript Draft--

**Manuscript Number:**	GIGA-D-22-00284R2
**Full Title:**	Chromosome-level genome assembly of the Pacific geoduck Panopea generosa reveals major inter- and intra-chromosomal rearrangements and substantial expansion of the copine gene family
**Article Type:**	Data Note
**Funding Information:**	Taishan Scholar Foundation of Shandong Province — Professor Nansheng Chen<br>Strategic Priority Research Program of Chinese Academy of Sciences (XDB42000000) — Professor Nansheng Chen<br>Chinese Academy of Sciences Pioneer Hundred Talents Program — Professor Nansheng Chen<br>Earmarked Workstation Fund for QRJH — Professor Chunde Wang, Professor Nansheng Chen
**Abstract:**	The Pacific geoduck Panopea generosa (class Bivalvia, order Adapedonta, family Hiatellidae, genus Panopea) is the largest known burrowing bivalve with considerable commercial value. Pacific geoduck and other geoduck clams play important roles in maintaining ecosystem health for their filter feeding habit and coupling pelagic and benthic processes. Here, we report a high-quality chromosome-level genome assembly of P. generosa to characterize its phylogeny and molecular mechanisms of its life strategies. The assembled P. generosa genome consists of 19 chromosomes with a size of 1.47 Gb, a contig N50 length of 1.6 Mb, and a scaffold N50 length of 73.8 Mb. The BUSCO test of the genome assembly showed 93.0% completeness. Constructed chromosome synteny revealed many occurrences of inter- and intra-chromosomal rearrangements between P. generosa and Sinonovacula constricta. Of the 35,034 predicted protein coding genes (PCGs), 30,700 (87.6%) could be functionally annotated in public databases, indicating the high quality of genome annotation. Comparison of gene copy numbers of gene families among P. generosa and 11 selected species identified 507 rapidly expanded P. generosa gene families that are functionally enriched in immune and gonad development and may be involved in its complex survival strategies. In particular, genes carrying the copine domains underwent additional duplications in P. generosa, which might be important for neuronal development and immune response. The availability of a fully annotated chromosome-level genome provides a valuable data set for genetic breeding of P. generosa.
**Corresponding Author:**	Nansheng Chen<br>Institute of Oceanology Chinese Academy of Sciences<br>Qingdao, Shandong CHINA
**Corresponding Author Secondary Information:**	
**Corresponding Author's Institution:**	Institute of Oceanology Chinese Academy of Sciences
**Corresponding Author's Secondary Institution:**	
**First Author:**	Jing Wang
**First Author Secondary Information:**	
**Order of Authors:**	Jing Wang
	Qing Xu
	Min Chen

Powered by Editorial Manager® and ProduXion Manager® from Aries Systems Corporation

	Yang Chen
	Chunde Wang
	Nansheng Chen
**Order of Authors Secondary Information:**	
**Response to Reviewers:**	Manuscript number: GIGA-D-22-00284R1<br>Title: “Chromosome-level genome assembly of the Pacific geoduck Panopea generosa reveals major inter- and intra-chromosomal rearrangements and substantial expansion of the copine gene family”<br><br>Responses to reviewers:<br><br>Reviewer #1: The authors have made a good attempt as address the comments on the previous version of the manuscript. While this version is a significant improvement, I still feel that there are outstanding issues that need to be addressed before it can be accepted for publication. Generally, the phrasing of the manuscript has been greatly improved, however, additional work is still required (some of which I have tried to detail in my specific comments below). Additionally, the authors have included some interesting now analysis in this version of the manuscript, however, these results are occasionally not well integrated into the text of the manuscript (some of the results in the results section could be reordered to improve the flow of the manuscript), do not always have a clear purpose (such as the Alphafold2 results), or are not described in the methods. I would suggest that the authors carefully review the new analysis to make sure it all fits within the manuscript. I have tried to give specific comments addressing each of these issues below.<br>Response 1: Thanks for the comments and suggestions. We have re-organized the content, and have expanded the method section.<br><br>It would be nice if in the next round the authors added line numbers to the manuscript so that it is easier to call out parts of the manuscript.<br>Response 2: Added.<br><br>Page 3: "revealed major inter- and intra-chromosomal exchanges" - Do you mean "rearrangements" not "exchanges". "Exchanges" makes it sounds like the two species are swapping DNA (HGT), which I assume you don't mean.<br>Response 3: We meant “rearrangement”. Thanks for your correction and we have revised.<br><br>Page 3: "neuronal development, immune response" should probably read "neuronal development and immune response". The comma is a little confusing.<br>Response 4: Corrected.<br><br>Page 5: "(which can be as long as 168 years) for P. generosa [4]"<br>I assume it should read "(which can be as long as 168 years for P. generosa [4])"<br>Response 5: Corrected.<br><br>Page 6: "recombination in evolution in bivalves" should probably read "recombination during the evolution of bivalves"<br>Response 6: Corrected.<br><br>Page 6: "Nevertheless, high-quality chromosome-level" should probably read "Nevertheless, a high-quality chromosome-level"<br>Response 7: Corrected.<br><br>Page 7: "The availability of the genome information" should probably read "The availability of this genome information"<br>Response 8: Corrected.<br><br>Page 7: I am not sure how the results in Table 1 support the sentence "The genome size of P. generosa was estimated to be 1.47 Gb using k-mer analysis (Table 1) [22]." Nothing in that table describes genome size or any genome-related statistics.<br>Response 9: We thank the review for pointing out this inconsistency. We have rewritten the paragraph.

Powered by Editorial Manager® and ProduXion Manager® from Aries Systems Corporation

Page 7: I still feel that the method that the authors used to estimate genome size is not adequately described. In their response to my comment on the previous version of the manuscript the authors mentioned that Jellyfish was used for k-mer enumeration, however, once you have decomposed the data into k-mers you still need to use additional tools (such as GenomeScope) or formula (k-mer count / homozygous peak coverage) to produce the final genome size/heterozygosity/repeat content estimations that the authors cite.

Additionally, the authors quote heterozygosity and repeat sequence proportion values but don't indicate how they were estimated from the enumerated k-mers (i.e., using which tool or formula). More detail about k-mer analysis needs to be provided in the methods.
Response 10: We are sorry for not providing adequate details. The genome size, heterozygosity, and repeat content were estimated using GCE 1.0.2. Relevant details are incorporated in the revised manuscript.

Page 7: (1) "The heterozygosity of P. generosa was comparable to those of most bivalves (Supplementary Table S3)." You compared the value from your genome to those of published genomes and came to what conclusion? This sentence doesn't really add anything to the results since there is no take away from it. Please elaborate on how your heterozygosity compares to that of the published genomes.
(2) Also, it would be best to add reference to the genomes in Table S3, to acknowledge the authors whose data you are citing.
Response 11: (1) This part is now rephrased in the revised manuscript. (2) References and NCBI accession numbers have been added.

Page 7: I might suggest that "Genome assembly using PacBio long reads (N50 = 26,513 bp) and Falcon assembler obtained an initial size of 1.51 Gb. Further assembly using Hi-C data obtained a genome with 19 pseudomolecules, suggesting 19 chromosomes of the P. generosa genome (Figure 1A), with an anchoring rate of 94.70%. This genome assembly has a total length of 1,474,161,289 bp with a contig N50 of 1.57 Mb and a scaffold N50 of 73.79 Mb (Figure 1B; Table 2; Supplementary Table S4). As expected, the genomic regions with low gene density typically had high repeat content, while the regions with high repeat content usually had high GC content."
Should read something like:
"The genome assembly produced using PacBio long reads (N50 = 26,513 bp) and the Falcon assembler had an initial size of 1.51 Gb. Further refinement using Hi-C data (anchoring rate of 94.70%) produced an assembly with 19 pseudomolecules, putatively the 19 chromosomes predicted in P. generosa (Figure 1A). This genome assembly has a total length of 1,474,161,289 bp with a contig N50 of 1.57 Mb and a scaffold N50 of 73.79 Mb (Figure 1B; Table 2; Supplementary Table S4). As expected, the genomic regions with low gene density typically had high repeat content, while the regions with high repeat content typically had high GC content."
Response 12: This part is now rephrased in the revised manuscript.

Page 7: Great work with Table S1. A very comprehensive picture of the available genomes. Why do some of the genomes not have references? Are they unpublished? If so, then you should state the location where you downloaded/accessed the data.
Response 13: Corrected.

Page 8: "The majority (57.99%) of the P. generosa genome was repetitive elements estimated using de novo searching and homolog prediction (Table 3). Distribution of these repetitive elements was uneven with repetitive content per 1 Mb varied from 34.76% to 84.89% (Figure 1B)."
Should probably read:
"The majority (57.99%) of the P. generosa genome was estimated, using de novo searching and homolog prediction, to be repetitive elements (Table 3). Distribution of these repetitive elements was uneven across the genome, with repetitive content per 1 Mb varying from 34.76% to 84.89% (Figure 1B)."
Response 14: The two sentences have been modified, and the second sentence has been moved to the third paragraph in the part “Genome annotation and evaluation”.

Powered by Editorial Manager® and ProduXion Manager® from Aries Systems Corporation

	Table 3: It's a little bit repetitive having the "Reparative sequence" and "Transposable elements" columns with almost all the same rows. Potentially just show the "Repetitive sequence" column.<br>Also, the "Total" values do not add up correctly (the Total "% in genome" for the "Reparative sequence" column should be 70.19% by my calculations, not 57.99%). Please double check all values.<br>Response 15: The structure of Table 3 was reformatted to avoid the repetitive content. In addition, we checked all the values for correctness. In fact, the total values were not simply the sum of all the values in the columns because of potential overlaps among different types of repetitive sequences.<br><br>Page 8: "Of these PCGs, 30,700 genes were annotated to contain conserved functional motifs (Supplementary Table S5, Table S7)." Should probably read "Of these PCGs, 30,700 were annotated with conserved functional motifs (Supplementary Table S5, Table S7)."<br>Response 16: Corrected.<br><br>Page 8: "and annotated protein-coding gene set were". Just the annotated protein-coding genes? Or all protein-coding genes?<br>Also, can you use the "PCG" acronym here?<br>Response 17: We meant "all protein-coding genes".<br><br>Page 8: "To evaluate the completeness of the assembly, the P. generosa genome assembly and annotated protein-coding gene set were assessed using BUSCO [23] with the metazoa_odb10 database (954 core genes), respectively."<br>This sentence is a little confusing. What is the "respectively" referring to since there are not to elements at the start and end of the sentence that you are associating together. Please rephrase.<br>Response 18: This sentence has been rephrased.<br><br>Page 8: "Regarding the gene set,"<br>Can these genes be referred to as "PCGs"?<br>Response 19: Yes. This sentence has been rephrased.<br><br>Page 8: "Although the P. generosa genome has 19 chromosomes as many other species in the order Adapedota" should probably read "Whereas the P. generosa genome has 19 chromosomes, the same number as in other species in the order Adapedota"<br>Response 20: Thanks for your correction. This sentence has been removed in the revised manuscript.<br><br>Page 9: "whose genome has been assembled at the chromosome-level revealed that 8 these two genomes have good chromosomal collinearity in general" should probably read "whose genome has been assembled at the chromosome-level, revealed that in general these two genomes have good chromosomal collinearity"<br>Response 21: This sentence has been rephrased.<br><br>Page 9: "clear one-to-one r correspondences S. constricta chromosomes" should probably read "clear one-to-one r correspondence with S. constricta chromosomes"<br>Also, what is "r correspondence"? I am unfamiliar with that term.<br>Response 22: The word "r" was an error, and was removed. This sentence has been removed in the rephrased paragraph.<br><br>Page 9: Would the term "rearrangements" be more appropriate than "exchanges" to use throughout the manuscript?<br>Response 23: Yes, the words "exchanges" have been corrected into "rearrangements".<br><br>Page 9: "also revealed that extensive intra-chromosomal" should probably be "also revealed extensive intra-chromosomal"<br>Response 24: Corrected.<br><br>Page 9: Sorry, I still find the description of the shared and specific gene families confusing. You state that there are two different groups of gene families that are specific to P. generosa. I assume the 2902 set is considering just the three other

species shown in Figure 4?
Also, why were these three other species chosen for analysis and visualization in Figure 4?
Why focus on just these species in Figure 4? You use all species for the orthogroup analysis, what benefit does focusing on just these four add to the analysis/manuscript?
Response 25: The 2902 set was specific to P. generosa, which were not in the three other species in the old Figure 4 (Figure 5 as a new version). The three other species P. martensi, P. yessoensis, and S. broughtonii were chosen for comparison, considering that they were from different taxonomic group and possessing different phylogenetic relationship in the phylogenetic tree.

Page 10: "identified in P. generosa genome" should probably read "identified in the P. generosa genome"
Response 26: Corrected.

Page 10: "166 pathways from the expanded gene" Some of those pathways at the end of your list have large p- and q-values. I would suggest filtering the data using a reasonable cutoff.
Response 27: We filtered the data by “q-value≤0.05”, and then expanded genes enriched significantly in 123 pathways.

Page 10: "(Supplementary Table S9) The" should probably read "(Supplementary Table S9). The"
Response 28: Thanks for your correction. This part has been removed in the rephrased paragraph.

Page 10: "Fluid shear stress and atherosclerosis, Phosphatidylinositol signaling system, suggesting their important contribution to the adaptation of benthic bivalves." This part of the sentence is a little confusing. You are listing a lot of terms here and the sentence doesn't flow well as a result. Consider rephrasing.
Also, are you missing an "and" at the end of the list?
Response 29: The “and” has been added, and this sentence has been rephrased.

Page 10: "which were found related to" should probably read "which have been shown to function as part of"
Response 30: Corrected.

Page 11: "Examination of the top 65 domains of gene families that presented expansions in P. generosa (Figure 7A) showed that the gene numbers of many important gene families were substantially expanded in P. generosa, including these containing the GIY-YIG catalytic domain (PF01541), the caspase recruitment domain (PF16739), the ApoA/ApoE domain (PF01442), and the copine domain (PF07002)." This sentence is confusing. You are looking at the expanded gene families, of course they are"substantially expanded in P. generosa", that is why you are looking at them right? Please rephrase.
Also, are the domains that you list the top most abundant or just the ones that you found most interesting? This is unclear.
Response 31: The domains listed in Figue7A were the 65 most abundant gene families that P. generosa possessing more gene numbers than 8 other bivalves, not just the ones that we found most interesting.

Page 11: "In particular, the copine gene family,…."
This paragraph opens with a statement about InterProScan, but then only talks about Pfam domains and gene copy expansion. This whole section could be reordered so that your results about gene family expansion are together (up with the paragraph starting "A total of 507 expanded gene families"), and the functional results (Pfam + KEGG) are together.
Response 32: The evolution of gene families was analyzed using two different but complementary approaches. The analysis using CAFÉ identifies the expansion and contraction of gene families, whose impact are further evaluated by functional analysis using GO and KEGG analysis. The analysis using InterProScan focused on genes whose protein sequences harbor particular functional domains (i.e., Pfam domains).

Page 11: "Interestingly, many genes formed local clusters"

Powered by Editorial Manager® and ProduXion Manager® from Aries Systems Corporation

This is very interesting. Do you see a single copy of these genes in roughly the same chromosomes in S. constricta? That is, does S. constricta have the "original" gene from each of the local clusters in its chromosomes or have these genes also spread throughout the P. generosa genome and then expanded locally.
Also, is this a common mechanism for gene family expansion? i.e., do the other genes that show expansion in this species also have identifiable local clusters?
Response 33: There were 11 copine genes distributed in 8 chromosomes of S. constricta genome. In general, there were 1-2 copine genes in the same chromosome S. constricta genome. This is a common mechanism for gene family expansion, such as IAP genes in Bathymodiolus platifrons (Sun et al, 2017).

Sun et al. Adaptation to deep-sea chemosynthetic environments as revealed by mussel genomes. 2017. Nat. Ecol. Evol. 1, 0121.

Figure 7: Double check the figure legend. I think the captions for B and C are switched?
In the text you describe these genes as the "copine genes", but in the figure legend you say that they are "genes annotated to PF07002 domains". Are these the same or are these different trees? This need to be clarified/consistent terminology used.
What are the support values for each of the nodes in the tree? Do the genes group based on their location across the chromosome? I would assume so since they are local duplications but it might be good to annotate or indicate where in the tree the genes from these local clusters fall.
Response 34: The support values for nodes were shown in the revised tree, when the support value was above 60. The genes from the same chromosome were clustered together, such as copine genes from chromosome 11, or copine genes from chromosome 10 and 7.

Page 12: "as for copine genes identified in other species"
As for what? This sentence is phrased like you are going to say something else but don't. Do you mean "like for copine genes identified in other species"?
Response 35: Yes, we do mean "like copine genes identified in other species".

Page 12: "imperfect annotation" Do you mean "imperfect prediction"?
"Annotation" usually means "functional annotation". If you are talking about gene structure issues you are usually talking about "gene prediction".
Response 36: Yes, “imperfect prediction” is correct.

Page 12: "Through the construction of the first high-quality chromosome-level genome assembly of the ecologically and economically important bivalves the Pacific geoduck P. generosa with cutting-edge genomic technologies, important insights into its genetic makeup and evolution have been gained.
Should probably read:
"The construction of the first high-quality chromosome-level genome assembly using cutting-edge technologies of the ecologically and economically important bivalve, the Pacific geoduck P. generosa, provides important insights into its genetic makeup and evolution.
Response 37: Corrected. This sentence has been rephrased.

Page 12: "The assembled genome size consists of 19 chromosomes with a genome size of 1.47 Gb, and a contig N50 of 1.6 Mb and 19 chromosomes."
Should probably read:
"The assembled genome consists of 19 chromosomes, has a size of 1.47 Gb, and a contig N50 of 1.6 Mb."
Response 38: Corrected.

Page 12: "and the conservation of PCGs in bivalves."
Does this show the conservation of PCGs in bivalves? I would think only your orthogroup analysis would do that, not the gene prediction/annotation workflow.
Response 39: This sentence has been removed in the revised manuscript.

Pahe 12-13: "P. generosa is a highly complex species with a heterozygosity of 1.37% and 57.99% repeat sequences in genome."
Does this make it complex? Compared to what? It seems pretty standard compared to

Powered by Editorial Manager® and ProduXion Manager® from Aries Systems Corporation

other bivalves. Please justify or remove.
Response 40: This sentence has been removed in the revised manuscript.

Page 13: "Large chromosomal fragments of P. generosa Pg02 and Pg11 matched to two S. constricta chromosomes (Chr1 and Chr10) and two S. constricta chromosomes (Chr1 and Chr10), respectively (Figure 2)."
This sentence repeats the phrase "two S. constricta chromosomes (Chr1 and Chr10)", please rephrase.
Response 41: Corrected.

Page 13 and 14: It is unclear how important it is that the copine genes contain C2 and vWA-domains. Slightly more context for this statement is required to help the reader understand why this is interesting.
Also, what is the significance of the Alphafold2 results? It is unsurprising that homologous genes would have highly similar structures. Unless there is something else that these results add to the manuscript I would suggest removing the Alphafold2 results and discussion. Additionally, the Alphafold2 work is not described in the methods.
Response 42: Because the copine genes expanded in P. generosa, it is important to learn the information of their distribution and structure.

Page 17: "The Hi-C heatmap was visualized using Juicebox (RRID:SCR_021172) presenting the counts of paired reads which each two bins aligned (with the bin length of 100 kb) as the interactive signals between each pair of two bins."
This sentence is a little confusing. Please consider rephrasing to improve readability.
Response 43: Corrected.

Page 17: "version5.4.3) [23]. using the"
Remove the full stop.
Response 44: Corrected.

Page 18: "Exonerate v2.2.0"
Can Exonerate take hits and reconstruct genes? My understanding is that Exonerate (by default) does the alignment for you and builds the gene from that. If there is a specific functionality of Exonerate that take pre-computed hits then this needs to be detailed.
Response 45: We did not mean that Exonerate reconstruct genes. In fact BLASTN was used to find hits from the P. generosa genome, which were aligned with gene sets from eight closely related bivalves (Patinopecten yessoensis, Pinctada fucata, Mytilus galloprovincialis, Limnoperna fortune, Argopecten purpuratus, Sinonovacula constricta, Scapharca broughtonii, and Crassostrea gigas). Exonerate was used to predict the structures (such as exon and intron) of each blast hits.

Page 18: I assume you used pre-trained models for Augustus and SNAP de novo prediction? If so, which models?
Response 46: Models used for each gene predictor Augustus and Snap traning were obtained from a set of high-quality proteins generated from the RNA-Seq and Iso-Seq dataset by MAKER 2. This detail was added in the revised manuscript.

Page 19: "procedures described previous studies" should probably read "procedures described in previous studies"
Response 47: Corrected.

Page 20: I see no new methods describing the InterProScan, Alphafold2, phylogenetic analysis (Figure 7C), or synteny/chromosome reorganization analysis.
The methods for these new analyses need to be extensively described.
Response 48: Corrected.

Figures: Please make sure that you adequately describe the figure in each figure legend. For example, the figure legend for Figure 2 does not describe each panel (A and B), the panels in Figure 7 maybe mislabeled, etc.
Response 49: Corrected.

Powered by Editorial Manager® and ProduXion Manager® from Aries Systems Corporation

	Reviewer #2: The authors improve the manuscript somewhat by adding more analyses of rapidly expended gene clusters and a couple of potential functional genes, as well as the chromosome synteny between two related species. As far as the whole manuscript is concerned, most of the studies in genomics or evolution analysis are so superficial that it cannot make much sense sometimes, except a chromosome-level genome assembly. In view of the data value of the genome assemblies, I think this manuscript is able to be published as a DATANOTE paper when the second round revision is done. The authors should revise the manuscript according to all the listed corrections and comments (D1 - D18) in the attached file. The final manuscript should also match the format of the DATANOTE paper. As to the terrible English writing (no improvement at all), I have tried to edit the abstract and result sections as possible. The discussion section should be re-written.<br>Response 50: Thanks for the comments and correction. We have checked all the problems in the revised manuscript.
**Additional Information:**	
**Question**	**Response**
Are you submitting this manuscript to a special series or article collection?	No
**Experimental design and statistics**<br><br>Full details of the experimental design and statistical methods used should be given in the Methods section, as detailed in our Minimum Standards Reporting Checklist. Information essential to interpreting the data presented should be made available in the figure legends.<br><br>Have you included all the information requested in your manuscript?	Yes
**Resources**<br><br>A description of all resources used, including antibodies, cell lines, animals and software tools, with enough information to allow them to be uniquely identified, should be included in the Methods section. Authors are strongly encouraged to cite Research Resource Identifiers (RRIDs) for antibodies, model organisms and tools, where possible.<br><br>Have you included the information requested as detailed in our Minimum Standards Reporting Checklist?	Yes
**Availability of data and materials**	Yes

All datasets and code on which the conclusions of the paper rely must be either included in your submission or deposited in publicly available repositories (where available and ethically appropriate), referencing such data using a unique identifier in the references and in the “Availability of Data and Materials” section of your manuscript.<br><br>Have you have met the above requirement as detailed in our Minimum Standards Reporting Checklist?	

Powered by Editorial Manager® and ProduXion Manager® from Aries Systems Corporation

This manuscript is clean after accepting all the revision. Click here to access/download;Manuscript;Manuscript_without_tracks_of_re

Chromosome-level genome assembly of the Pacific geoduck *Panopea generosa* reveals major inter- and intra-chromosomal rearrangements and substantial expansion of the copine gene family

Jing Wang[1,2,3], Qing Xu[1,2,3], Min Chen[4], Yang Chen[1,2,3], Chunde Wang[4,5*], Nansheng Chen[1,2,3,6*]

[1]CAS Key Laboratory of Marine Ecology and Environmental Sciences, Institute of Oceanology, Chinese Academy of Sciences, Qingdao, China

[2]Laboratory of Marine Ecology and Environmental Science, Qingdao National Laboratory for Marine Science and Technology, Qingdao, China

[3]Center for Ocean Mega-Science, Chinese Academy of Sciences, Qingdao, China

[4]Yantai Institute of Coastal Zone Research and Center for Ocean Mega-Science, Chinese Academy of Sciences, Yantai, China

[5]Marine Science and Engineering College, Qingdao Agricultural University, Qingdao, China

[6]Department of Molecular Biology and Biochemistry, Simon Fraser University, Burnaby, BC, Canada

Jing Wang Email: wangjing2019@qdio.ac.cn; Qing Xu Email: xuqing_77@163.com; Min Chen Email: mchen@yic.ac.cn; Yang Chen Email: cy4043@hevttc.edu.cn.

∗Correspondence address. Chunde Wang, Yantai Institute of Coastal Zone Research and Center for Ocean Mega-Science, Chinese Academy of Sciences, Yantai, China. E-mail: chundewang2007@163.com; Nansheng Chen, CAS Key Laboratory of Marine Ecology and Environmental Sciences, Institute of Oceanology, Chinese Academy of Sciences,

Qingdao, China. Email: chenn@qdio.ac.cn

Abstract

The Pacific geoduck *Panopea generosa* (class Bivalvia, order Adapedonta, family Hiatellidae, genus *Panopea*) is the largest known burrowing bivalve with considerable commercial value. Pacific geoduck and other geoduck clams play important roles in maintaining ecosystem health for their filter feeding habit and coupling pelagic and benthic processes. Here, we report a high-quality chromosome-level genome assembly of *P. generosa* to characterize its phylogeny and molecular mechanisms of its life strategies. The assembled *P. generosa* genome consists of 19 chromosomes with a size of 1.47 Gb, a contig N50 length of 1.6 Mb, and a scaffold N50 length of 73.8 Mb. The BUSCO test of the genome assembly showed 93.0% completeness. Constructed chromosome synteny revealed many occurrences of inter- and intra-chromosomal rearrangements between *P. generosa* and *Sinonovacula constricta*. Of the 35,034 predicted protein-coding genes (PCGs), 30,700 (87.6%) could be functionally annotated in public databases, indicating the high quality of genome annotation. Comparison of gene copy numbers of gene families among *P. generosa* and 11 selected species identified 507 rapidly expanded *P. generosa* gene families that are functionally enriched in immune and gonad development and may be involved in its complex survival strategies. In particular, genes carrying the copine domains underwent additional duplications in *P. generosa*, which might be important for neuronal development and immune response. The availability of a fully annotated chromosome-level genome provides a valuable data set for genetic breeding of *P. generosa*.

Keywords: *Panopea generosa*, chromosome-level genome assembly, genetic breeding,

evolutionary adaptation

Introduction

The Pacific geoduck *Panopea generosa* is one member of genus *Panopea* which includes the world's largest burrowing bivalves. *P. generosa* is usually found in low intertidal and subtidal sediments throughout the northeast Pacific coast, including the United States (Alaska, Washington, and California), Canada (British Columbia), and Mexico (north Baja Pacific Coast) [1, 2]. Geoducks can reach more than 25 cm in shell length, and more than 100 cm in siphon length [3]. Geoduck adults are usually buried in muddy-sandy sediment at depths ranging 60–100 cm, with only their siphon tips exposed to respire, capture food, and release secretion/excretion products and gametes. The sedentary behavior may contribute to their long life spans which can be as long as 168 years for *P. generosa* [4]. Due to these unique life strategies, it is expected that geoduck should have distinctive growth and development mechanisms, especially in relation to benthic life and immune system.

Geoduck clams play important roles in maintaining ecosystem health for their filter feeding habit and coupling pelagic and benthic processes by ejecting undigested mucus-bound feces and pseudo feces to the sediment surface. They are prey for sea otters, fishes, crabs, and sea stars [5, 6]. As marine calcifiers, shell concentrations of *Panopea* inside Scalichnus burrows have been analyzed to reconstruct the sequence of events related to storm events [7]. Geoduck clams possess great commercial fishery value in Canada and the USA [8]. Since the recruitment of geoducks have been low due to overfishing and their vulnerability to environmental changes [9, 10], there has been an increasing interest in genetic breeding of geoducks.

Bivalves are an ancient lineage of bilaterian, and are a diverse Class of Mollusca. To date, the chromosome-level genomes of only about 40 bivalve species have been assembled [11]. These genomes can provide a resource for comparative genomic for gaining evolutionary and other insights of bivalves and even molluscs. These genomes show a remarkable level of diversity. For example, the assembled genome sizes of bivalves vary widely, ranging from 543.9 Mb in *Lutraria thynchaena* [12] to 2.6 Gb in *Modiolus philippinarum* [13] (Supplementary Table S1). Among bivalves, the genome sizes of most superorder Imparidentia species ranged from 1 Gb to 1.8 Gb, and that of the species in the order Adapedonta, which includes *P. generosa*, ranged from 1 Gb to 1.5 Gb [14].

The numbers of chromosomes also vary substantially among bivalves, suggesting active genome recombination during the evolution of bivalves [15]. While some species of the order Ostreida, including *Crassostrea gigas* [16], *Crassostrea virginica*, *Crassostrea hongkongensis* [17], *Crassostrea ariakensis* [18], *Crassostrea angulate*, and *Ostrea edulis*, have 10 chromosomes, the species of the order Pterioidea and Mytilida have 14–15 chromosomes, such as *Pinctada fucata* [19], *Mytilus coruscus* [20] and *Limnoperna fortunei* (Supplementary Table S1). The chromosome numbers of the order Myida varied from 16–17. Interestingly, the chromosome number of species in most other orders is 19, including Venerida, Cardiida, Unionida, Arcida, and Pectinida . The chromosome number of all reported species in the order Adapedonta, which includes *P. generosa*, is also 19.

Nevertheless, a high-quality chromosome-level reference genome of *P. generosa* is currently not available, hindering the development of geoduck genetic breeding programs. In this study, we reported the first chromosome-scale genome assembly for *P. generosa*

generated using cutting-edge technologies including next-generation sequencing, long read sequencing, and high-throughput chromosome conformation capture (Hi-C) technologies. We further performed gene family clustering, phylogenetic analysis, and gene family expansion and contraction, in order to understand its adaptation, growth, development and immunity. The availability of this genome information will facilitate research in molecular evolution and genetic breeding.

Results

Genome sequencing and assembly

For the genome assembly of *P. generosa*, short reads were obtained for estimating the genome size, heterozygosity rate, and repeat content, long reads were obtained for initial genome assembly, and Hi-C reads were obtained for the construction of chromosomes (Table 1).

Genome size, heterozygosity rate, and repeat content of *P. generosa* estimated by the *k*-mer analysis [21] of the short reads were 1.47 Gb, 1.37% and 68.08% respectively (Supplementary Figure S1 and Table S2). The assembled genome of *P. generosa*, which was generated using PacBio long reads (N50 length = 26,513 bp) with Falcon and Hi-C data, consisted of 19 pseudomolecules (Figure 1; Supplementary Table S3), with a contig anchoring rate of 94.70%. The assembled *P. generosa* genome has a total length of 1.47 Gb (1,474,161,289 bp) with a contig N50 length of 1.6 Mb and a scaffold N50 length of 73.79 Mb (Table 2).

Commented [D1]: Anchored contigs or scaffolds?? It should be clarified.

The heterozygosity rate of *P. generosa* (1.37%) fell within the range of the heterozygosity rates of bivalves whose genomes have been sequenced and assembled,

which vary broadly from 0.11% in *Margaritifera margaritifera* [22] to 3.20% in *Crassostrea gigas* [23], The heterozygosity rate of *P. generosa* (1.37%) was close to that of another burrowing bivalve *Sinonovacula constricta* (1.55%) and a deep-sea mussel *Bathymodiolus platifrons* (1.03–1.24%) [13, 24] (Supplementary Table S4).

Genome annotation and evaluation

The majority (57.99%) of the *P. generosa* genome was estimated to be repetitive elements (Table 3), which is similar to but lower than the predicted content of repetitive elements using *k*-mer analysis (68.08%). The top three most frequent categories of repetitive elements in the *P. generosa* genome were DNA transposons (21.6%), long interspersed nuclear elements (LINEs, 9.1%) and long terminal repeats (LTRs, 3.76%) (Table 3).

Commented [D2]: The k-mer analysis estimates the content of the repetitive seqeunces is 57.99%. The de novo annotation of the repeats also estimaltes 57.99%. Obviously, the authors don't understand diference between the two methods, which cannot have exactly the same statistic number. It should be corrected.

A total number of 35,034 protein-coding genes (PCGs) were annotated in the *P. generosa* genome. The average gene length, average CDS length, average number of exons per gene, average exon length, and average intron length were 13,469 bp, 1273 bp, 6, 220 bp, and 2171 bp, respectively. Of these PCGs, 30,700 (87.6%) were annotated with conserved functional motifs according to the public database including Nr, Swissprot, KEGG, KOG, TrEMBL, Interpro, and GO (Supplementary Table S5), suggesting the high quality of genome annotation.

Commented [D3]: Uhe used database in annotation should be clarified.

The distribution of repetitive elements was highly uneven, with regional content of the repetitive elements varied from 34.76% to 84.89% in the *P. generosa* genome, with peaks occurring at regions with low gene density (Figure 1B).

To evaluate the completeness of the assembly, the *P. generosa* genome was tested

using BUSCO [25] with the metazoa_odb10 database (954 core genes). We found that 93.0% of core PCGs were identified as full-length in the *P. generosa* genome (Table 4), higher than that of *S. constricta* (91.5%) [14] (Supplementary Table S6). We further tested the completeness of annotated PCGs using BUSCO, which were aligned to 88.4% of the full-length core orthologs, suggesting that quality of the annotation can be further improved in the future.

Chromosomal synteny analysis between *P. generosa* and *S. constricta*

Comparative analysis of genome-wide gene collinearity revealed a high chromosome synteny between *P. generosa* and *S. constricta*. Of the 19 *P. generosa* chromosomes, 17 chromosomes were found to have one-to-one correspondences with 17 *S. constricta* chromosomes (Figure 2). However, several large-scale inter-chromosomal rearrangements were also identified, such as the arrangement among *P. generosa* chromosomes *pg02* and *pg11* and *S. constricta* chromosomes *sc01* and *sc10* (Figure 2). Despite these large-scale inter-chromosomal rearrangements, the numbers of chromosomes of these two species were both 19.

In addition to these major inter-chromosomal rearrangement events, comparative analysis of these two genomes also revealed extensive intra-chromosomal rearrangements, which resulted in low gene synteny within these chromosomes. Instead of a clear diagonal linear relationship between genes of these two species *P. generosa* and *S. constricta*, a near random scattering of the relationships were observed (Figure 2A). These intra-chromosomal rearrangement events were also clearly shown in Figure 2B, such as *P. generosa* chromosome *pg01* vs *S. constricta* chromosome *sc06*.

Commented [D4]: Tthe authors should describe which region of which chromosome, as well as the invovled length, underwent the rearrangement, not to tell the readers themselve to look for it in your figure.

Evolutionary analysis of *P. generosa* and other bivalves

Phylogenetic analysis using 326 one-to-one single-copy orthologous genes of the 12 species showed that *P. generosa* was tightly clustered with other bivalves as expected (Figure 3). According to the phylogenetic tree, the divergence time of *P. generosa* from its nearest node, which represents the common ancestor of many other bivalves, was approximately 491.5 Mya (Figure 3). This divergence time between *P. generosa* and other bivalves was similar to the divergence time between *S. constricta* and other bivalves [14]. Interestingly, the numbers of chromosomes of bivalves vary substantially, and closely related bivalves can have different numbers of chromosomes (Figure 3). The oysters *C. gigas* with 10 chromosomes and *P. martensi* with 14 chromosomes diverged from the clam *S. broughtoni* and scallops with 19 chromosomes 409.4 MYA. What's more, the scallop *A. purpuratus* with 16 chromosomes diverged from another scallop *P. maximus* with 19 chromosomes 61.7 MYA.

Comparative analysis of gene families

In total, 30,616 gene families were identified among *P. generosa* and 11 other species (*Pinctada martensi*, *C. gigas*, *B. platifrons*, *Patinopecten yessoensis*, *Pecten maximus*, *Argopecten purpuratus*, *Scapharca broughtonii*, *Homo sapiens*, *Xenopus tropicaalis, Danio rerio, and Caenorhabditis elegans*) (Table 5, Figure 4, Supplementary Table S7). As compared with the other 11 species, 7917 genes belonging to 1749 gene families were found to be *P. generosa*-specific, which fell into the range of 1567–15051 species-specific gene families identified in 12 bivalves [26]. Comparative analysis of the PCGs of *P. martensi*, *S. broughtonii*, *P. yessoensis*, and *P. generosa* revealed 6490 common gene

Commented [D5]: Why no fossil calibration in estimating the divergence time ??? it should be used.

Commented [静王6R5]: The time correction points were taken from the Timetree website.

Commented [D7]: Is it correct?? Annotation of too many species-specific gene clusters were caused by the wrong analysis or annotation. Are many repetitive element genes included in your annotations. It should be corrected.

Commented [静王8R7]: We have checked our analysis again. The PCG annotation was carried out shielding the repetitive elements. Besides, the 7917 species-specific genes or 1749 species-specific gene families are not unnormal. In this study, B. platifrons has 1,775 species-specific gene families. In a previous study, the species-specific gene family number was 1567-15051.

families shared by these species and 2902 gene families specific to *P. generosa* (Figure 5).

A total of 507 rapidly expanded gene families (involving 2,734 genes) and 875 rapidly contracted gene families (involving 792 genes) were identified in the *P. generosa* genome compared to the most recent common ancestor of both *P. generosa* and other 11 species (Figure 6). The annotation with the KEGG pathway database [27] revealed that the genes of expanded families were distributed in 123 pathways, which were mainly enriched in organismal systems, genes associated with diseases, environmental information processing (e.g. phototransduction), and phosphatidylinositol signaling system, suggesting their important contribution to the adaptation of benthic bivalves. According to the enriched KEGG pathways of expanded gene families in *P. generosa* (Supplementary Table S8), there were a few significant enriched pathways (Q value < 0.05) related to gonad development. For example, adrenergic signaling in cardiomyocytes, and glycine, serine and threonine metabolism which have been shown to function as part of spermatogenesis of the fluted giant clam *Tridacna squamosa* [28]. Moreover, oocyte meiosis, apoptosis, Ras signaling pathway, calcium signaling pathway, steroid hormone biosynthesis, GnRH signaling pathway, insulin signaling pathway, oxytocin signaling pathway, and ovarian steroidogenesis were documented to be enriched in *Procambarus clarkii* ovary development [29]. Geoducks have become a focus of significant aquaculture research and development with a considerable commercial value [30, 31]. The enriched gonad development-related pathways and genes could provide basic data for the further genetic breeding research of *P. generosa* and its closely related species.

Commented [D9]: Can *P. generosa* have the human disease??

Commented [静王10R9]: The words have been corrected.

We further compared gene families in different bivalves by searching for functional domains contained in PCGs in *P. generosa* and eight other bivalves using InterProScan [32]. Examination of the top 65 most frequent domains that had specific expansions in *P. generosa* (Figure 7A) showed that the gene numbers of many important gene families were substantially expanded in *P. generosa*, including these containing the GIY-YIG catalytic domain (PF01541), the caspase recruitment domain (PF16739), the ApoA/ApoE domain (PF01442), and the copine domain (PF07002). In particular, the copy number of the copine gene family, which has been implicated in a range of cell signaling [33], was 22 in *P. generosa*, twice as many as those identified in *S. constricta*. Examination of positions of the *P. generosa* copine genes revealed that they were distributed in chromosomes *pg02*, *pg05*, *pg07*, *pg10*, *pg11* and *pg17* (Figure 7B). Interestingly, many genes formed local clusters (e.g. eight copies in Pg11), suggesting that the large copine gene set observed in *P. generosa* might have been achieved via tandem duplication of the copine genes in recent evolution. Phylogenetic analysis of the copine genes annotated in *P. generosa* (22 genes) and *S. constricta* (11 genes) revealed good orthologous relationships, as well as one-to-multiple relationships (Figure 7C), confirming that genes inside copine gene clusters in *P. generosa* (Figure 7B) were highly similar.

Most of the copine homologs contain the vWA-domain, while some has both C2-domain and the vWA-domain in *P. generosa* (Figure 7D) like copine genes identified in other species [33]. Lengths of the coding sequences of some copies were comparatively short, which might be due to errors of genome assembly or gene prediction. Prediction of 3D structures using AlphaFold2 [34] revealed that their structures are highly conserved

Commented [D11]: Add the references for the function information of copine domain?

Commented [D12]: Don't italicize the chromosome number. Theck all the maintext and correct them.

Commented [静王13R12]: These chromosome number presents the chromosome nucleotide sequences. Therefore, we italicized them.

among different species, suggesting the conservation of protein functions. For example, the three-dimensional structure of Pg02g00048 in *P. generosa* showed high similarity to that of XP_0533888641 in *Mercenaria mercenaria* [35] (Figure 7E).

Discussion

Through the completion of this project, we have successfully constructed the first high-quality chromosome-level genome assembly of the ecologically and economically important bivalves the Pacific geoduck *P. generosa*, enriching the expanding list of chromosome-level genomes of bivalves. The assembled *P. generosa* genome consists of 19 chromosomes with a genome size of 1.47 Gb, and a contig N50 of 1.6 Mb. The *P. generosa* genome represents the third genome of the third species in the order Adapedonta, after *S. constricta* and *Solen grandis*. The *P. generosa* genome assembly represented another instance of chromosome-level genomes of the bivalve species [11], an ancient lineage of bilaterian, and a diverse Class of Mollusca (Supplementary Table S1).

The assembled *P. generosa* genome size (1.47 Gb) fell within the range of the reported genomes of bivalves, which varied from 543.9 Mb in *Lutraria thynchaena* [12] to 2.6 Gb in *Modiolus philippinarum* [13] (Supplementary Table S1). The assembled *P. generosa* genome size (1.47 Gb) also fell within the genome size range of superorder Imparidentia species (1 Gb to 1.8 Gb), and the genome size range of the order Adapedonta species (1 Gb to 1.5Gb), which includes *P. generosa* [14].

Although both the two genomes have the exactly same number of 19 chromosomes, comparative analysis between the genomes of *P. generosa* and its most closely related species *S. constricta* revealed extensive inter- and intra-chromosomal exchanges. Large

Commented [D14]: Don't italicize it.

Commented [D15]: Don't italicize it.

Commented [D16]: The discussion section is the worst part of this manuscipt when the authors keep repeating the information the result section have mentioned. The discussion part should be re-written by adding more extended thoughts and information that the result section not mensioned or not deeply mentioned.

chromosomal fragments of *P. generosa pg02* and *pg11* matched to two *S. constricta* chromosomes (*sc01* and *sc10*), respectively (Figure 2). Despite such large inter-chromosomal exchanges, the numbers of chromosomes of both *P. generosa* and *S. constricta* were identical. Within chromosomes, the order of genes showed even more extensive alterations, resulting in the lack of clear diagonal alignments (Figure 2). Among bivalves the numbers of chromosomes vary substantially, suggesting active genome recombination during the evolution of bivalves [15]. The chromosome numbers of bivalves in the Infraclass Heteroconchia are 16–19, relatively higher than those in the Infraclass Pteriomorphia which are 10–19. In the Infraclass Pteriomorphia most oysters and scallops possess 19 chromosomes, similar to those of most clams in the Infraclass Heteroconchia. In the phylogenetic analysis of the Infraclass Pteriomorphia the blood clam was closer to the scallops, compared with oysters (Figure 3). What is interesting, the Manila clam *R. philippinarum* was clustered in the clade of the Infraclass Pteriomorphia (Figure 3).

Comparative analysis revealed that the numbers of PCGs of many gene families with important functions also changes substantially in evolution (Figure 7A). In particular, the copine gene family was found to be substantially expanded in *P. generosa*, with 22 copine genes were identified. The number of copine genes in *P. generosa* was twice of those in *P. constricta*. Many of these 22 copine genes in *P. generosa* formed tandem clusters, with one cluster containing eight copine genes, suggesting that these genes were formed via tandem duplications. The composition of functional domains, which include C2 domain and vWA-domain (Figure 7D), and the similarity of three-dimensional structure to that of known copine genes (Figure 7E) suggests functional conservation of function of copine genes in

P. generosa. Thus, the completion of *P. generosa* facilitates genome comparative analysis of gene families to uncover important leads for exploring molecular insight into its physiology and evolution.

The successful construction of *P. generosa* chromosome-level genome not only enables genomic identification and analysis of important genes in this organism, but also enables comparative analysis of bivalve genomes, which is critical for tracking the species formation, evolution, and biodiversity of bivalves.

Commented [D17]: I don't konw whether there is diference between this paragraph and the corresponding pargraph in the result section.

Commented [D18]: The conclusive description is not simply to repeat the above-mentioned information. The similar sentenses can be found several times in this maintext. This conclusion paragraph should be re-written.

Methods

Sampling collection

Geoduck *P. generosa* samples were collected from the Strait of Georgia (49°41′12″N, 124°51′33″W) of British Columbia, Canada in the spring of 2019. The samples showed typical morphological features of *P. generosa*. The identification of the samples was also supported by the high similarities of the molecular marker *cox1* to the reference sequence of *P. generosa* (PID of 99.55, coverage of 100%) [36]. The samples- were transferred to laboratory and kept in a tank with running water for a week. One sample was chosen and dissected on ice to collect tissue samples, including labial palp, heart, foot, gonad, gill, hepatopancreas, siphon, and mantle muscle. This animal was identified to be a female as indicated by the presence of eggs in the smear of the gonad under a compound microscope. Dissected tissues were quickly frozen in liquid nitrogen and then stored at -80°C before DNA and RNA extraction.

DNA library construction and sequencing

Genomic DNA of *P. generosa* was extracted using a standard phenol-chloroform

extraction method [37]. The quality of DNA was determined by gel electrophoresis to ensure the DNA samples met library sequencing requirements. Sequence libraries with insert size of 300 bp were constructed for BGISEQ-500 sequencing platform (RRID:SCR_017979) according to manufacturer's protocol. The sequencing data produced were used in the genome size estimation by *k*-mer analysis [21] and for correcting errors in the Pilon (RRID:SCR_014731) assembly [38]. A Hi-C library with insert size of 300 bp was constructed to provide long-range information (without position information) on the grouping and linear organization of sequences along entire chromosomes to assemble the scaffolds into chromosome-level scaffolds [39]. For Hi-C library construction, gonad tissue was dissociated, and cells were collected and crosslinked with 1% formaldehyde (Sigma) and 0.2M glycine (Sigma). After that, the fixed powder was resuspended in nuclei isolation buffer and then incubated in 0.5% SDS for 10 min at 62°C. Then the reaction was quenched with 10% Triton X-100 (Sigma) and the nuclei were collected by centrifugation. Then the DNA was digested with MboI (NEB), and the overhang was filled and biotinylated before ligated by T4 DNA ligase (NEB). Before library construction, the purified DNA was sheared, and biotin-containing fragments were captured on streptavidin-coated beads using Dynabeads MyOne Streptavidin T1 (Invitrogen). The fragments were then end-repaired and linked with adaptors before eight cycles of PCR reaction with KAPA HiFi HotStart ReadyMix (Kapa Biosystem). After that, the Hi-C library was sequenced with BGISEQ-500 platform. And a PacBio library with insert size of 20 Kb was constructed to obtain long reads by the PacBio Sequel platform using the Sequel Sequencing Kit 3.0. The adapters and low-quality reads in raw data generated

by the BGISEQ platform were cut off by SOAPnuke1.5.6 using the parameter as "-n 0.01 -l 20 -q 0.1 -i -Q 2 -G -M 2 -A 0.5 -d" [40]. PacBio raw data were filtered with the default parameters by using Pacific Biosciences SMRT analysis software (v2.3.1) to filter the low quality reads.

RNA library construction and sequencing

RNA-Seq and Iso-Seq were conducted to obtain transcriptome data to aid genome annotation. The total RNAs was extracted by Trizol (Invitrogen, Carlsbad, CA, USA) from eight tissues of the same *P. generosa* individual, including labial palp, heart, foot, gonad, gill, hepatopancreas, siphon, and mantle muscle. The quality and quantity of RNA in each sample was assessed using a NanoDrop and an Agilent 2100 bioanalyzer (Thermo Fisher Scientific, MA, USA). The construction of mRNA libraries for RNA-Seq, the mRNA was enriched by mRNA Capture Beads (BGI, LB00V60), and incubated at 85°C for 8 minutes for fragmentation. Reverse transcription was performed with Strand Specificity Reagent and 1st Strand Enzyme Mix (Optimal Dual-mode mRNA Library Prep Kit, BGI, LR00R96) to generate the first strand cDNA. After that the second strand cDNA generation and end repair were performed with 2nd Strand Buffer and 2nd Strand Enzyme Master Mix. Then the adaptors (BGI, LA00R04) were ligated to the cDNAs. Then the library was purified and selected depending upon product requirements for amplification. The mRNA libraries were sequenced using the BGISEQ-500 platform. For Iso-Seq, the total RNA was extracted from the equally mixed tissues of the 8 tissues above. The PacBio SMRTbell library was prepared using the SMARTer PCR cDNA Synthesis kit (Clontech), the Qubit dsDNA HS Assay Kit 2.0 (Invitrogen) and the Agilent DNA 12000 kit (Agilent Technologies), and

sequenced by the PacBio Sequel sequencer (RRID:SCR_017989) with Sequel Sequencing Kit 3.0.

Genome size estimation and genome assembly

Genome size of *P. generosa* was estimated using *k*-mer analysis. Counting of *k*-mers was conducted using Jellyfish (RRID:SCR_005491, version 2.2.10) [21]. The genome size, heterozygosity, and repeat content were estimated using GCE 1.0.2 [41]. For genome assembly, long reads generated from PacBio Sequel platform were assembled using Falcon (RRID:SCR_016089) [42], which was subsequently polished using Arrow. Short paired-end clean reads from BGISEQ-500 were then used for correcting post-processing errors and resolving conflicts of assembly via Pilon (RRID:SCR_014731, version 1.22) [38]. The assembled contigs were corrected for mis-joins, orders, orients and anchored contigs from the draft assembly into a candidate chromosome-length assembly by Hi-C data using Juicer (RRID:SCR_017226) [43] and 3d-DNA [44]. The scaffolds shorter than 20 Kb were removed. Finally, the candidate assembly were reviewed with Juicebox Assembly Tools (RRID:SCR_021172) for quality control and interactive corrections [45]. The Hi-C heatmap was visualized using Juicebox (RRID:SCR_021172) (with the bin length of 100 kb) as the interactive signals between each pair of two bins. The completeness of genome assembly was assessed by BUSCO (RRID:SCR_015008, version5.4.3) [25] using the metazoa_odb10 database. The genome landscape illustrating the length, repeat element density, gene density and GC content was created by circos-0.69-9 (RRID:SCR_011798) [46].

Annotations of gene structure and function

Homologous and *de novo* predictions were both applied to annotate transposable elements in the *P. generosa* genome. In homologous prediction, RepeatMasker (RRID:SCR_012954) and RepeatProteinMask [47] were used to screen the *P. generosa* genome for known transposable elements in the RepBase library (RRID:SCR_021169) [48]. In *de novo* prediction, RepeatModeler (version 1.0.4) was first used for de novo candidate database construction of repetitive elements, and repetitive sequences were then annotated using RepeatMasker. Tandem repeats were de novo predicted using Tandem repeats finder (version 4.07) [49]. The results were then integrated and duplicates were eliminated.

Three complementary approaches were adopted to predict PCGs in *P. generosa* genome, including homology-based prediction, *de novo* annotation, and transcriptome-based prediction. For homology-based prediction, gene sets from eight closely related bivalves (*P. yessoensis, P. fucata, Mytilus galloprovincialis, Limnoperna fortune, A. purpuratus, S. constricta, S. broughtonii, and C. gigas*) were used. First, protein repertoires of those organisms were aligned against the *P. generosa* genome using TBLASTN (RRID:SCR_011822) [50]. Then gene structures were predicted from these blast hits by Exonerate v2.2.0 [51]. *de novo* gene prediction was performed using a combination of Augustus (RRID:SCR_008417) [52] and SNAP (RRID:SCR_007936) [53] with default settings. Models used for each gene predictor Augustus and SNAP training were obtained from a set of high-quality proteins generated from the RNA-Seq and ISO-seq dataset by MAKER 2 (RRID:SCR_005309). For transcriptome-based prediction using RNA-Seq data, RNA-Seq reads were directly mapped to the genome using TopHat2 (RRID:SCR_013035)

[54]. The mapped reads were subsequently assembled into gene models (Cufflinks-set) by Cufflinks (RRID:SCR_014597) [55]. For transcriptome-based prediction based on Iso-Seq data, Iso-Seq reads were directly mapped to the genome using GMAP (RRID:SCR_008992) [56]. The mapped reads were subsequently assembled by PASA (RRID:SCR_014656) [57]. Gene predictions from the homology-based approach, *de novo* approach, RNA-Seq-based and Iso-Seq-based evidences were merged, and redundancy was removed to form a comprehensive consensus gene set using Maker 2 (RRID:SCR_005309) [58]. To validate the completeness of the gene structure annotation, we also used BUSCO (version5.4.3) with the metazoa_odb10 database [25].

Collinearity analysis

Homologous PCGs in *P. generosa* and *S. constricta* were identified using BLAST v2.11.0 (blastp, E value $1e^{-5}$), which were used for subsequent analysis using WGDI [59]. WGDI analysis results included dotplot and syntenic blocks with default parameters.

Phylogenetic analysis and divergence time estimation

Gene families were constructed using the OrthoMCL (RRID:SCR_007839) pipeline [53]. We selected *P. generosa* and other 11 species (*C. gigas*, *P. yessoensis*, *P. maximus*, *A. purpuratus*, *S. broughtonii*, *P. martensi*, *B. platifrons*, *H. sapiens*, *X. tropicaalis*, *D. rerio*, and *C. elegans*) for gene family analysis. For the gene set of each genome, only the transcript with the longest coding sequence was selected from alternate splice transcripts. Genes with less than 50 amino acids were removed from further analysis. Protein sequences were aligned by “all-vs-all BLASTP” (E value = $1e^{-5}$) [44]. Then the Markov clustering (MCL) algorithm implemented in OrthoMCL was used to group orthologues and

paralogues from all input species with an inflation value of 1.5 [60].

The phylogenetic tree was constructed following procedures described in previous studies [14, 35, 61]. Briefly, for phylogenetic tree construction and divergence time estimation, shared single copy genes of *P. generosa* and 11 other species were used. The protein sequences of single-copy orthologs among the 12 species were aligned using MUSCLE v3.7 (RRID:SCR_011812) [62] with default parameters. Phylogenetic relationships were inferred based on the super-matrix estimated from the concatenated alignment of single-copy genes using the maximum likelihood (ML) [63] method implemented in RAxML v2.2 (RRID:SCR_006086) [64] with the optimal amino acid substitution model selected by the PROTGAMMALGX parameter.

Based on gene family identification and phylogenetic analysis, single copy genes and mcmctree in PAML [65] were used to estimate divergence time [66-69]. The time correction points were *C. elegans* and *H. sapiens* (678.3–855.2 MYA), *D. rerio* and *H. sapiens* (413.1–443.0 MYA), *X. tropicalis* and *H. sapiens* (347.0–357.9 MYA). The time correction points were taken from the Timetree website. The operating parameters of mcmctree: burn in = 10000, sample number = 1000000, sample frequency = 50.

Gene family analysis and three dimensional protein structure modeling

The clustering results of gene families and the phylogenetic tree with divergence time estimated were used to analyze the expansion and contraction of orthologous gene families between ancestor and each of the 12 species (*P. generosa* and the other 11 species) using a stochastic birth and death model with lambda parameter by CAFE (RRID:SCR_005983, version 4.0) [70]. This model was further used to calculate the

number of gene families along each lineage on the phylogenetic tree. A probabilistic graphical model was introduced to calculate the probability of transitions in gene family size from parent to child nodes. The family-wide P-Values were calculated in each lineage based on the conditional likelihood.

In addition, we also compared the gene families between *P. generosa* and the other eight bivalves. Pfam domains of *P. generosa* and the other eight bivalves were obtained using InterProscan [32] (5.54-87.0) and visualized using TBtools (v1.120) [71]. The positions of copine genes in *P. generosa* chromosomes were illustrated using R packages gggenes, and the structure of copine genes were illustrated using Gene Structure Display Server 2.0 on line [72]. Phylogenetic trees of copine genes of *P. generosa* and *S. constricta* were constructed using the maximum likelihood (ML) [63] method implemented in MEGA (v7.0.26) with 1000 bootstrap replicates.

Three-dimensional protein structure models were predicted using AlphaFold2 and visualized using ChimeraX [34].

Data Availability

The Whole Genome project of *P. generosa* has been deposited at NCBI/BioProject PRJNA859289. The raw next-generation sequencing reads of DNA are available at SRA (SRR22190027-SRR22190030); raw long-read PacBio sequencing reads of DNA are available at SRA (SRR22190026; raw next-generation sequencing reads of RNA are available at SRA (SRR22190032); raw Hi-C reads are available at SRA (SRR22190025); and raw long-read PacBio sequencing reads of RNA are available at SRA(SRR22190031). The genome assembly data have been deposited under accession No.

JAPMAH000000000.1.

Additional Files

Supplementary Figure S1: The 17-mer count distribution for the genome size estimation.

Supplementary Figure S2: The enriched KEGG pathways of significantly expanded gene families ($p \leq 0.01$, top 20) in *P. generosa*

Supplementary Table S1: The genome assembly information of bivalves in the public database

Supplementary Table S2: Statistics of 17-mer analysis

Supplementary Table S3: The chromosomes information of *P. generosa*

Supplementary Table S4: The heterozygosity of bivalves reported

Supplementary Table S5: Functional annotation of the predicted protein-coding genes in *P. generosa* genome assembly

Supplementary Table S6: The assembly quality of chromosomal genomes of bivalves by BUSCO

Supplementary Table S7: The information of 30,616 gene families of *P. generosa* and 11 other species

Supplementary Table S8: The enriched KEGG pathways of expanded gene families in *P. generosa* genome assembly

Abbreviations

PCG: protein-coding gene; Akt: RAC serine/threonine-protein kinase; bp: base pairs;

BLAST: Basic Local Alignment Search Tool; BUSCO: Benchmarking Universal Single-Copy Orthologs; BWA: Burrows-Wheeler Aligner; cAMP: cyclic adenosine monophosphate; CAMs: cell adhesion molecules; cGMP-PKG: cGMP-dependent protein kinase G; Gb: gigabase pairs; GC: guanine-cytosine; GnRH: Gonadotropin-releasing hormone; GO: gene ontology; Hi-C: High-throughput/resolution chromosome conformation capture; kb: kilobase pairs; KEGG: Kyoto Encyclopedia of Genes and Genomes; Mb: megabase pairs; MYA: million years ago; NCBI: National Center for Biotechnology Information; NF-kappa B: nuclear factor kappa-B; NOD-like receptor: nucleotide-binding oligomerization domain-like receptor; PacBio: Pacific Biosciences; RAxML: Randomized Axelerated Maximum Likelihood; PI3K: phosphatidylinositol-4,5-bisphosphate 3-kinase catalytic subunit alpha/beta/delta; Rap1: Ras-related protein1; RNA-Seq: RNA sequencing; Iso-Seq: Isoform-sequencing; tRNA: transfer RNA; TRP channel: transient receptor potential ion channel.

Competing Interests

The authors declare that they have no competing interests.

Funding

This study was supported by the Taishan Scholar Project Special Fund (to Nansheng Chen), the Strategic Priority Research Program of Chinese Academy of Sciences (XDB42000000), the Chinese Academy of Sciences Pioneer Hundred Talents Program (to Nansheng Chen), and an Earmarked Workstation Fund for QRJH (to Chunde Wang and

Nansheng Chen).

Authors' Contributions

N.C. and C.W. conceived and designed the study. M.C. and Y.C. prepared the samples. J.W. and Q.X. performed analyses. J.W. wrote the paper with input from co-authors. All authors read and approved the final version for submission.

References

1. González-Peláez SS, Leyva-Valencia I, Pérez-Valencia SA, et al. Distribution limits of the geoduck clams *Panopea generosa* and *P. globosa* on the Pacific coast of Mexico. *Malacologia* 2013;**56**:85-94.
2. Vadopalas B, Pietsch TW, Friedman CS. The proper name for the geoduck: resurrection of *Panopea generosa* Gould , 1850, from the synonymy of *Panopea abrupta* (Conrad, 1849) (Bivalvia: Myoida: Hiatellidae). *Malacologia* 2010;**52**:169-73.
3. Goodwin CL, Pease BC. Geoduck, *Panopea abrupta* (Conrad, 1849), size, density, and quality as related to various environmental parameters in Puget Sound, Washington. *J Shellfish Res* 1991;**10**:65-77.
4. Orensanz JM, Hand CM, Parma AM, et al. Precaution in the harvest of Methuselah's clams the difficulty of getting timely feedback from slow-paced dynamics. *Can J Fish Aquat Sci* 2004;**61**:1355-72.
5. Newell RIE. Ecosystem influences of natural and cultivated populations of suspension-feeding bivalve molluscs: A review. *J Shellfish Res* 2004;**23**:51-61.
6. Straus KM, MacDonald PS, Crosson LM, et al. Effects of geoduck aquaculture on the environment: A synthesis of current knowledge. Washington Sea Grant Technical Report WSG-TR 13-02, 2013.
7. Santos A, Aguirre J, Rodríguez-Tovar FJ, et al. Multi-storm events recorded on *Panopea* burrows (Pliocene, Spain): The importance of sequestered information inside burrows. *Palaeogeogr Palaeoclimatol Palaeoecol* 2018;**507**:155-67.
8. Bureau D., Hajas W., Hand C.M., et al. Age, size structure and growth parameters of geoducks (*Panopea abrupta*, Conrad 1849) from seven locations in British Columbia sampled in 2001 and 2002. Canadian Technical Report of Fisheries and Aquatic Sciences, 2003, p. 29.
9. Valero JL, Canada O, Madryn P, et al. Geoduck (*Panopea abrupta*) recruitment in the Pacific Northwest: long-term changes in relation to climate. *CalCOFI Reports* 2004;**45**:80-6.
10. Sloan NA, Robinson SMC. Age and gonad development in the geoduck clam *Panopea abrupta* (Conrad) from southern British Columbia, Canada. *J Shellfish Res* 1984;**4**:131-7.
11. Liu F, Li Y, Yu H, et al. MolluscDB: an integrated functional and evolutionary genomics database for the hyper-diverse animal phylum Mollusca. *Nucleic Acids Res* 2020;**49**:D988-D997.
12. Thai BT, Lee YP, Gan HM, et al. Whole genome assembly of the snout otter clam, *Lutraria rhynchaena*, using Nanopore and Illumina Data, benchmarked against bivalve genome assemblies. *Front Genet* 2019;**10**:1158.
13. Sun J, Zhang Y, Xu T, et al. Adaptation to deep-sea chemosynthetic environments as revealed by mussel genomes. *Nat Ecol Evol* 2017;**1**:0121.
14. Ran Z, Li Z, Yan X, et al. Chromosome-level genome assembly of the razor clam *Sinonovacula constricta* (Lamarck, 1818). *Mol Ecol Resour* 2019;**19**:1647-58.
15. Halanych KM, Kocot KM. Genome evolution: Shellfish genes. *Nat Ecol Evol* 2017;**1**:0142.

16. Peñaloza C, Gutierrez AP, Eöry L, et al. A chromosome-level genome assembly for the Pacific oyster *Crassostrea gigas*. *GigaScience* 2021;**10**:giab020.
17. Peng J, Li Q, Xu L, et al. Chromosome-level analysis of the *Crassostrea hongkongensis* genome reveals extensive duplication of immune-related genes in bivalves. *Mol Ecol Resour* 2020;**20**:980-94.
18. Wu B, Chen X, Yu M, et al. Chromosome-level genome and population genomic analysis provide insights into the evolution and environmental adaptation of Jinjiang oyster *Crassostrea ariakensis*. *Mol Ecol Resour* 2021;**22**:1529-44.
19. Du X, Fan G, Jiao Y, et al. The pearl oyster *Pinctada fucata martensii* genome and multi-omic analyses provide insights into biomineralization. *GigaScience* 2017;**6**:1-12.
20. Yang JL, Feng DD, Liu J, et al. Chromosome-level genome assembly of the hard-shelled mussel *Mytilus coruscus*, a widely distributed species from the temperate areas of East Asia. *GigaScience* 2021;**10**:giab024.
21. Marçais G, Kingsford C. A fast, lock-free approach for efficient parallel counting of occurrences of k-mers. *Bioinformatics* 2011;**27**:764-70.
22. Gomes-Dos-Santos A, Lopes-Lima M, Machado AM, et al. The Crown Pearl: a draft genome assembly of the European freshwater pearl mussel *Margaritifera margaritifera* (Linnaeus, 1758). *DNA Res* 2021;**28**:1–10.
23. Zhang G, Fang X, Guo X, et al. The oyster genome reveals stress adaptation and complexity of shell formation. *Nature* 2012;**490**:49-54.
24. Zhang T, Yin J, Tang S, et al. Dissecting the chromosome-level genome of the Asian Clam (*Corbicula fluminea*). *Sci Rep* 2021;**11**:15021.
25. Simão FA, Waterhouse RM, Ioannidis P, et al. BUSCO: assessing genome assembly and annotation completeness with single-copy orthologs. *Bioinformatics* 2015;**31**:3210-2.
26. Teng W, Xie X, Nie H, et al. Chromosome-level genome assembly of *Scapharca kagoshimensis* reveals the expanded molecular basis of heme biosynthesis in ark shells. *Mol Ecol Resour* 2022;**22**:295-306.
27. Kanehisa M, Goto S, Kawashima S, et al. The KEGG resource for deciphering the genome. *Nucleic Acids Res* 2004;**32**:D277-D280.
28. Li J, Zhou Y, Zhou Z, et al. Comparative transcriptome analysis of three gonadal development stages reveals potential genes involved in gametogenesis of the fluted giant clam (Tridacna squamosa). *BMC Genomics* 2020;**21**:872.
29. Jiang H, Liu H, Ma X, et al. Transcriptome analysis of *Procambarus clarkii* to screen genes related to ovary development,immunity and growth. *J Fish Chn* 2021;**45**:396-414.
30. Ren Y, Liu W, Pearce CM, et al. Effects of selected mixed-algal diets on growth and survival of early postset juveniles of the Pacific geoduck clam, *Panopea generosa* (Gould, 1850). *Aquac Nutr* 2015;**21**: 152-61.
31. Nava-Gómez GE, Garcia-Esquivel Z, Carpizo-Ituarte E, et al. Survival and growth of geoduck clam larvae (*Panopea generosa*) in flow-through culture tanks under laboratory conditions. *Aquac Res* 2018;**49**:294-300.
32. Zdobnov EM, Apweiler R. InterProScan--an integration platform for the signature-recognition methods in InterPro. *Bioinformatics* 2001;**17**:847-8.

33. Perestenko PV, Pooler AM, Noorbakhshnia M, et al. Copines-1, -2, -3, -6 and -7 show different calcium-dependent intracellular membrane translocation and targeting. *FEBS J* 2010;**277**:5174-89.
34. Jumper J, Evans R, Pritzel A, et al. Highly accurate protein structure prediction with AlphaFold. *Nature* 2021;**596**:583-9.
35. Song H, Guo X, Sun L, et al. The hard clam genome reveals massive expansion and diversification of inhibitors of apoptosis in Bivalvia. *BMC Biology* 2021;**19**:15.
36. Bisbal-Pardo CI, Del Río-Portilla MA, Rocha-Olivares A. The complete mitochondrial DNA of the Pacific Geoduck clam (*Panopea generosa*). *Mitochondrial DNA A DNA Mapp Seq Anal* 2016;**27**:1955-6.
37. Green M, J S. Molecular cloning: a laboratory manual. 4th Edn. Vol. II. New York, NK: Cold Spring Harbor Laboratory Press.
38. Walker BJ, Abeel T, Shea T, et al. Pilon: an integrated tool for comprehensive microbial variant detection and genome assembly improvement. *PloS One* 2014;**9**:e112963.
39. Burton JN, Adey A, Patwardhan RP, et al. Chromosome-scale scaffolding of de novo genome assemblies based on chromatin interactions. *Nat Biotechnol* 2013;**31**:1119-25.
40. Chen Y, Chen Y, Shi C, et al. SOAPnuke: a MapReduce acceleration-supported software for integrated quality control and preprocessing of high-throughput sequencing data. *GigaScience* 2018;**7**:1-6.
41. Liu B, Shi Y, Yuan J, et al. Estimation of genomic characteristics by analyzing *k*-mer frequency in de novo genome projects. *arXiv: Genomics* 2013.
42. Pendleton M, Sebra R, Pang AW, et al. Assembly and diploid architecture of an individual human genome via single-molecule technologies. *Nat Methods* 2015;**12**:780-6.
43. Durand NC, Shamim MS, Machol I, et al. Juicer provides a one-click system for analyzing loop-resolution Hi-C experiments. *Cell Syst* 2016;**3**:95-8.
44. Dudchenko O, Batra SS, Omer AD, et al. De novo assembly of the *Aedes aegypti* genome using Hi-C yields chromosome-length scaffolds. *Science* 2017;**356**:92-5.
45. Robinson JT, Turner D, Durand NC, et al. Juicebox.js provides a cloud-based visualization system for Hi-C data. *Cell Syst* 2018;**6**:256-8.
46. Krzywinski M, Schein J, Birol I, et al. Circos: an information aesthetic for comparative genomics. *Genome Res* 2009;**19**:1639-45.
47. Bergman CM, Quesneville H. Discovering and detecting transposable elements in genome sequences. *Brief Bioinform* 2007;**8**:382-92.
48. Bao W, Kojima KK, Kohany O. Repbase Update, a database of repetitive elements in eukaryotic genomes. *Mobile DNA* 2015;**6**:11.
49. Benson G. Tandem repeats finder: a program to analyze DNA sequences. *Nucleic Acids Res* 1999;**27**:573-80.
50. Altschul SF, Gish W, Miller W, et al. Basic local alignment search tool. *J Mol Biol* 1990;**215**:403-10.
51. Slater GS, Birney E. Automated generation of heuristics for biological sequence comparison. *BMC Bioinformatics* 2005;**6**:31.
52. Stanke M, Morgenstern B. AUGUSTUS: a web server for gene prediction in eukaryotes

that allows user-defined constraints. *Nucleic Acids Res* 2005;**33**:W465-W467.

53. Johnson AD, Handsaker RE, Pulit SL, et al. SNAP: a web-based tool for identification and annotation of proxy SNPs using HapMap. *Bioinformatics* 2008;**24**:2938-9.
54. Kim D, Pertea G, Trapnell C, et al. TopHat2: accurate alignment of transcriptomes in the presence of insertions, deletions and gene fusions. *Genome Biol* 2013;**14**:R36.
55. Trapnell C, Roberts A, Goff L, et al. Differential gene and transcript expression analysis of RNA-seq experiments with TopHat and Cufflinks. *Nat Protoc* 2012;**7**:562-78.
56. Wu TD, Watanabe CK. GMAP: a genomic mapping and alignment program for mRNA and EST sequences. *Bioinformatics* 2005;**21**:1859-75.
57. Haas BJ, Delcher AL, Mount SM, et al. Improving the Arabidopsis genome annotation using maximal transcript alignment assemblies. *Nucleic Acids Res* 2003;**31**:5654-66.
58. Holt C, Yandell M. MAKER2: an annotation pipeline and genome-database management tool for second-generation genome projects. *BMC Bioinformatics* 2011;**12**:491.
59. Sun P, Jiao B, Yang Y, et al. WGDI: A user-friendly toolkit for evolutionary analyses of whole-genome duplications and ancestral karyotypes. *Mol Plant* 2022;15:1841-51.
60. Li L, Stoeckert CJ, Jr., Roos DS. OrthoMCL: identification of ortholog groups for eukaryotic genomes. *Genome Res* 2003;**13**:2178-89.
61. Tian HF, Hu QM, Li Z. A high-quality de novo genome assembly of one swamp eel (*Monopterus albus*) strain with PacBio and Hi-C sequencing data. *G3 (Bethesda)* 2021;11.
62. Edgar RC. MUSCLE: multiple sequence alignment with high accuracy and high throughput. *Nucleic Acids Res* 2004;**32**:1792-7.
63. Guindon S, Gascuel O. A simple, fast, and accurate algorithm to estimate large phylogenies by maximum likelihood. *Syst Biol* 2003;**52**:696-704.
64. Stamatakis A. RAxML-VI-HPC: maximum likelihood-based phylogenetic analyses with thousands of taxa and mixed models. *Bioinformatics* 2006;**22**:2688-90.
65. Yang Z. PAML 4: phylogenetic analysis by maximum likelihood. *Mol Biol Evol* 2007;**24**:1586-91.
66. Thorne JL, Kishino H, Painter IS. Estimating the rate of evolution of the rate of molecular evolution. *Mol Biol Evol* 1998;**15**:1647-57.
67. Vogel JP, Garvin DF, Mockler TC, et al. Genome sequencing and analysis of the model grass Brachypodium distachyon. *Nature* 2010;**463**:763-8.
68. Blanc G, Wolfe KH. Widespread paleopolyploidy in model plant species inferred from age distributions of duplicate genes. *Plant Cell* 2004;**16**:1667-78.
69. Sanderson MJ. r8s: inferring absolute rates of molecular evolution and divergence times in the absence of a molecular clock. *Bioinformatics* 2003;**19**:301-2.
70. Han MV, Thomas GW, Lugo-Martinez J, et al. Estimating gene gain and loss rates in the presence of error in genome assembly and annotation using CAFE 3. *Mol Biol Evol* 2013;**30**:1987-97.
71. Chen C, Chen H, Zhang Y, et al. TBtools: An integrative toolkit developed for interactive analyses of big biological data. *Mol Plant* 2020;**13**:1194-202.
72. Hu B, Jin J, Guo AY, et al. GSDS 2.0: an upgraded gene feature visualization server. *Bioinformatics* 2015;**31**:1296-7.

Figure 1: The *P. generosa* genome contig contact matrix using Hi-C data and landscape. (A) Hi-C analysis of *P. generosa* genome contigs. Chromosomes are arranged in the size order from left to right and from top to bottom. The color bar illuminates the logarithm of the contact density from red (10) to white (0) in the plot. (B) The genomic landscape of *P. generosa*: from outer to inner circles: a, the 19 chromosomes; b–d, repetitive element density, gene density, and GC density across the genome, respectively, drawn in 1 Mb non-overlapping windows.

Figure 2: Chromosome synteny of *P. generosa* and *S. constricta*. (A) A dotplot of correspondence of homologous genes in *P. generosa* and *S. constricta*. The red, blue, and gray dots present three similarity levels of homologous gene pairs between *P. generosa* and *S. constricta*. The best match genes are shown in red, while the next best match genes in blue and the least best match genes in gray. (B) A circos plot of synteny analysis between *P. generosa* and *S. constricta*. Line in different colors depicts interchromosomal synteny.

Figure 3: Phylogenetic analysis of *P. generosa* with related species. The estimated species divergence time (million years ago) and the 95% confidential intervals are labeled at each branch site. Divergence times used for time recalibration is illuminated as red dots in the tree.

Figure 4: The distribution of single-copy orthologs, multiple-copy orthologs, unique paralogs, other orthologs, and unclustered genes in *P. generosa* and related species.

Figure 5: Distribution of shared gene families among *P. generosa*, *P. martensi*, *S. broughtonii*, and *P. yessoensis*. Intersections between species indicate the numbers of shared gene families, whereas unique family numbers are shown in species-specific areas. The center represents the number of families shared by all the 4 species.

Figure 6: Dynamic evolution and distribution of gene families among *P. generosa* and related species. The numbers of gene gains (+) and losses (−) are shown on the branches, which are also displayed as pie plots: the green part for gene gain, the red part for gene losses and the blue part for gene remained. The divergence times are dated and displayed below the phylogenetic tree. MRCA: most recent common ancestor.

Figure 7: Comparative analysis of gene families based on Pfam annotation in *P. generosa* and related bivalves. A. The 65 most abundant gene families that *P. generosa* possessing more gene numbers than 8 other bivalves. B. Chromosomal distribution of copine genes in *P. generosa genome*. C. The phylogenetic tree of copine genes in *P. generosa* and *S. constricta*, shown in the background colors of red and pink, respectively.D. Domain structures of copine genes predicted in *P. generosa* genome. E. The 3D structures of Pg02g00048 gene in *P. generosa* and XP_0533888641 gene in *Mercenaria mercenaria*.

This manuscript retains all traces of revision.
Click here to access/download;Manuscript;Manuscript_with_tracks_of_revisi

Formatted

Chromosome-level genome assembly of the Pacific geoduck *Panopea generosa* reveals major inter- and intra-chromosomal rearrangements and substantial expansion of the copine gene family

Jing Wang[1,2,3], Qing Xu[1,2,3], Min Chen[4], Yang Chen[1,2,3], Chunde Wang[4,5*], Nansheng Chen[1,2,3,6*]

[1]CAS Key Laboratory of Marine Ecology and Environmental Sciences, Institute of Oceanology, Chinese Academy of Sciences, Qingdao, China

[2]Laboratory of Marine Ecology and Environmental Science, Qingdao National Laboratory for Marine Science and Technology, Qingdao, China

[3]Center for Ocean Mega-Science, Chinese Academy of Sciences, Qingdao, China

[4]Yantai Institute of Coastal Zone Research and Center for Ocean Mega-Science, Chinese Academy of Sciences, Yantai, China

[5]Marine Science and Engineering College, Qingdao Agricultural University, Qingdao, China

[6]Department of Molecular Biology and Biochemistry, Simon Fraser University, Burnaby, BC, Canada

Jing Wang Email: wangjing2019@qdio.ac.cn; Qing Xu Email: xuqing_77@163.com; Min Chen Email: mchen@yic.ac.cn; Yang Chen Email: cy4043@hevttc.edu.cn.

*Correspondence address. Chunde Wang, Yantai Institute of Coastal Zone Research and Center for Ocean Mega-Science, Chinese Academy of Sciences, Yantai, China. E-mail: chundewang2007@163.com; Nansheng Chen, CAS Key Laboratory of Marine Ecology and Environmental Sciences, Institute of Oceanology, Chinese Academy of Sciences,

Qingdao, China. Email: chenn@qdio.ac.cn

Abstract

The Pacific geoduck *Panopea generosa* (class Bivalvia, order Adapedonta, family Hiatellidae, genus *Panopea*) is the largest known burrowing bivalve with considerable commercial value. Pacific geoduck and other geoduck clams play important roles in maintaining ecosystem health for their filter feeding habit and coupling pelagic and benthic processes.~~ Chromosome-level genomes of geoduck clams will contribute to genetic breeding, as well as ecosystem and climate change biology.~~ Here, we report ~~the first~~a high-quality chromosome-level genome assembly of *P. generosa* ~~with the purpose~~ to ~~unravel~~ characterize its ~~phylogene~~phylogeny~~tic~~ ~~characteristics~~ and molecular mechanisms of its life strategies~~, and promote research on genetic breeding~~. The assembled *P. generosa* genome consists of 19 chromosomes with a size of 1.47 Gb, ~~with~~ , a contig N50 length of 1.6 Mb, and a scaffold N50 length of 73.8 Mb. The BUSCO ~~analysis~~ test of the genome assembly showed 93.0% completeness. Constructed chromosome synteny revealed ~~the~~ many occurrences of inter- and intra-chromosomal rearrangements ~~Comparative analysis of the genomes of~~ between ~~two closely related species in the order Adapedonta,~~ *P. generosa* and *Sinonovacula constricta*~~, revealed major inter- and intra-chromosomal exchanges~~. Of the 35,034 predicted protein-coding genes (PCGs), 30,700 ~~genes~~ (87.6~~3~~%) could be ~~were~~ functionally annotated ~~with~~in ~~the~~ public databases, indicating the high quality of genome annotation. ~~Comparative analysis~~Comparison of gene copy numbers of gene families among ~~the genomes of~~ *P. generosa* and 11 ~~related~~selected species identified 507 rapidly expanded *P. generosa* gene families that are ~~and 875 contracted gene families~~ ~~in~~~~according to the phylogeny of the species *P. generosa*, of which the annotations~~

~~displayed that they were~~ functionally~~-~~. ~~E~~enriched in ~~ment analysis revealed significant expansion of~~ immune and gonad development ~~gene families~~ ~~involved~~and may be involved in ~~that may promote~~ its complex survival strategies. In particular, ~~the~~ genes carrying the copine domains underwent ~~more~~ additional duplications in *P. generosa*, which might be important ~~to~~for ~~in~~ neuronal development and immune response. ~~gene family, which plays an important role in calcium signaling, membrane trafficking, and cytoskeletal dynamics, and has been implicated in several physiological and pathological processes such as neuronal development, immune response substantially expanded in *P. generosa* with 22 members annotated.~~ The availability of a fully annotated chromosome-level ~~*P. generosa*~~ genome ~~assembly and its annotated gene set provide~~provide~~d~~s a ~~useful molecular platform~~ ~~fundamental~~valuable data set for ~~research on its~~ ~~the study of~~ genetic breeding ~~in~~of *P. generosa*.

Keywords: *Panopea generosa*, chromosome-level genome assembly, genetic breeding, evolutionary adaptation

Introduction

The Pacific geoduck *Panopea generosa* is one member of genus *Panopea* which includes the world's largest burrowing bivalves. *P. generosa* is usually found in low intertidal and subtidal sediments throughout the northeast Pacific coast, including the United States (Alaska, Washington, and California), Canada (British Columbia), and Mexico (north Baja Pacific Coast) [1, 2]. Geoducks can reach more than 25 cm in shell length, and more than 100 cm in siphon length [3]. Geoduck adults are usually buried in muddy-sandy sediment at depths ranging 60–100 cm, with only their siphon tips exposed to respire, capture food, and release secretion/excretion products and gametes. The sedentary behavior may contribute to their long life spans ~~(~~which can be as long as 168 years~~)~~ for *P. generosa* [4]. Due to these unique life strategies, it is expected that geoduck should have distinctive growth and development mechanisms, especially in relation to benthic life and immune system.

Geoduck clams play important roles in maintaining ecosystem health for their filter feeding habit and coupling pelagic and benthic processes by ejecting undigested mucus-bound feces and pseudo feces to the sediment surface. They are prey for sea otters, fishes, crabs, and sea stars [5, 6]. As marine calcifiers, shell concentrations of *Panopea* inside Scalichnus burrows have been analyzed to reconstruct the sequence of events related to storm events [7]. Geoduck clams possess great commercial fishery value in Canada and the USA [8]. Since the recruitment of geoducks have been low due to overfishing and their vulnerability to environmental changes [9, 10], there has been an increasing interest in genetic breeding of geoducks.

Bivalves are an ancient lineage of bilaterian, and are a diverse Class of Mollusca. To date, the chromosome-level genomes of only about 40 bivalve species have been assembled [11]. These genomes can provide a resource for comparative genomic for gaining evolutionary and other insights of bivalves and even molluscs. These genomes show a remarkable level of diversity. For example, the~~The~~ assembled genome sizes of bivalves vary widely, ranging from 543.9 Mb in *Lutraria thynchaena* [12] to 2.6 Gb in *Modiolus philippinarum* [13] (Supplementary Table S1). Among bivalves, the genome sizes of most superorder Imparidentia species ranged from 1 Gb to 1.8 Gb, and that of the species in the order Adapedonta, which includes *P. generosa*, ranged from 1 Gb to 1.5 Gb [14].

The numbers of chromosomes also vary substantially among bivalves, suggesting active genome recombination during~~in~~ the evolution ~~in~~of bivalves [15]. While some ~~the~~ species of the order Ostreida, including *Crassostrea gigas* [16], *Crassostrea virginica*, *Crassostrea hongkongensis* [17], *Crassostrea ariakensis* [18], *Crassostrea angulate*, and *Ostrea edulis*, have 10 chromosomes, the species of the order ~~Ostreida~~Pterioidea and Mytilida~~,~~ have 14–15 chromosomes, such as ~~including~~ *Pinctada fucata* [19], ~~*Pinctada imbricata* and the species of the order Mytilida including~~ *Mytilus coruscus* [20] and *Limnoperna fortunei*~~*Mytilus edulis* have 14 chromosomes~~ (Supplementary Table S1). The chromosome numbers of the order Myida ~~Cardiida~~ varied from ~~17~~16–~~19~~17 ~~[21]~~. Interestingly, the chromosome number~~s of chromosomes~~ of species in most other orders is 19, including Venerida, Cardiida, ~~Venerida,~~ Unionida, Arcida, and Pectinida~~, are 19.~~ ~~,~~ ~~except for *Corbicula fluminea* [22] possessing 18 chromosomes and *Argopecten* scallop~~

possessing 16 chromosomes. The chromosome reported numbers of chromosomes of all reported species in the order Adapedonta, which includes *P. generosa*, are is also 19.

Nevertheless, a high-quality chromosome-level reference genome of *P. generosa* is currently not available, hindering the development of geoduck genetic breeding programs. In this study, we reported the first chromosome-scale genome assembly for *P. generosa* generated using cutting-edge technologies including next-generation sequencing, long read sequencing, and high-throughput chromosome conformation capture (Hi-C) technologies. We further performed gene family clustering, phylogenetic analysis, and gene family expansion and contraction, in order to understand its adaptation, growth, development and immunity. The availability of the this genome information will facilitate research in molecular evolution and genetic breeding.

Results

Genome sequencing and assembly

For the genome assembly of *P. generosa*, short reads were obtained for estimating the genome size, heterozygosity rate, and repeat contentsurvey analysis, long reads were obtained for initial genome assembly, and Hi-C reads were obtained for scaffolding and the construction of chromosomes (Table 1).

Formatted: Font: Italic

–Genome size, heterozygosity rate, and repeat content of *P. generosa* estimated by The sequence data information was summarized in Table 1. The genome size of *P. generosa* was estimated to be 1.47 48 Gb using Tthe *k*-mer analysis (Table 1Supplementary Figure S1) [21] of the short reads sitywere 1.487 Gb. , The heterozygosityus rate and repeated repetitive sequence content were estimated to be 1.37%

and 68.0857.99% (Supplementary Figure S1 and Table S2), respectively (Supplementary Figure S1 and Table S2).

The assembled genome of *P. generosa*, which was generated using PacBio long reads (N50 length = 26,513 bp) with Falcon and Hi-C data, consisted of 19 pseudomolecules (Figure 1; Supplementary Table S43), with a contig anchoring rate of 94.70%. The assembled *P. generosa* genome has a total length of 1.47 Gb (1,474,161,289 bp) with a contig N50 length of 1.6 Mb and a scaffold N50 length of 73.79 Mb (Table 2).

The heterozygosity rate of *P. generosa* (1.37%) fell within the range of Tthe heterozygosity ratesies of bivalves whose genomes have been sequenced and assembled, which werevary broadly from 0.11% in *Margaritifera margaritifera* [22] to –3.20% in *Crassostrea gigas* ()[23]according to the previous studies., The heterozygosity rate of *P. generosa* (1.37%) The heterozygosities of *Potamilus streckersoni* [26], *Venustaconcha ellipsiformis* [27], and *Margaritifera margaritifera* [24] in Unionida were lowest, 0.11%–0.60%. The heterozygosity of *P. generosa* was much lower thanclose to –that of another burrowing bivalve *M. philippinarumSinonovacula constricta* (1.552.02%) and a[13] but slightly higher than that of deep-sea mussel *Bathymodiolus platifrons* (1.03–1.24%) [13, 24] comparable to those of most bivalves (Supplementary Table S3S4).

Genome assemblye produced using PacBio long reads (N50 length = 26,513 bp) and with Falcon assembler obtained an initial size of 1.51 Gb. Further assembly usingand Hi-C data obtainedgenerated a genome with 19 pseudomolecules, suggesting 19 chromosomes of the *P. generosa* genome (Figure 1A), with an anchoring rate of 94.70%. This genome assemblyies haves a total length of

Commented [D1]: Anchored contigs or scaffolds?? It should be clarified.

Formatted: Font: Italic

Formatted: Font: Italic

Formatted: Font: Italic

Formatted: Font: Italic

Formatted: Font: Italic

Formatted: Font: Italic

Commented [D2]: high or low?? it should be clarified.

Commented [D3]: Anchored contigs or scaffolds?? It should be clarified.

1,474,161,289 bp with a contig N50 length of 1.61.57 Mb and a scaffold N50 length of 73.79 Mb (Figure 1B; Table 2; Supplementary Table S4). As expected, the genomic regions with low gene density typically had high repeat content, while the regions with high repeat content usually had high GC content.

The assembled genome size of *P. generosa* (1.47 Gb) fell in the range of the reported genomes of bivalves, which varied from 543.9 Mb in *Lutraria thynchaena* [12] to 2.6 Gb in *Modiolus philippinarum* [13] (Supplementary Table S1).

Genome annotation and evaluation

The majority (57.99%) of the *P. generosa* genome was estimated using *de novo* searching and homolog prediction, to be repetitive elements estimated using *de novo* searching and homolog prediction (Table 3), which is similar to but lower than the predicted amountcontent of repetitive elements using *k*-mer analysis (68.08%). The top three most frequent categories of repetitive elements in the *P. generosa* genome were Distribution Content of these repetitive elements was uneven with repetitive content per 1 Mb varied from 34.76% to 84.89% in the genome (Figure 1B). DNA transposons (21.6%), long interspersed nuclear elements (LINEs, 9.1%) and long terminal repeats (LTRs, 3.76%) were the top three categories of repetitive elements in the *P. generosa* genome (Table 3).

A total number of 35,034 protein-coding genes (PCGs) were annotated in the *P. generosa* genome. The average gene length, the average CDS length, the average number of exons per gene, the average exon length, and the average intron length were 13,469 bp, 1273 bp, 6, 220 bp, and 2171 bp, respectively mean number of exons per gene was 5.78 exons per gene (Supplementary Table S4). Of these PCGs, 30,700 (87.6%)

Commented [D4]: I don't agree with this point. The genic regions have high GC content. The repetitive elements usually located on intergenic or intronic regions. The authors should check it.

Commented [D5]: The k-mer analysis estimates the content of the repetitive seqeunces is 57.99%. The de novo annotation of the repeats also estimaltes 57.99%. Obviously, the authors don't understand diference between the two methods, which cannot have exactly the same statistic number. It should be corrected.

~~genes~~ were annotated with~~to contain~~ conserved functional motifs according to the public database ~~of~~including Nr, Swissprot, KEGG, KOG, TrEMBL, Interpro, and GO (Supplementary Table S~~5, Table S7~~5), suggesting the high quality of genome annotation.

The distribution of repetitive elements was highly uneven ~~(Figure 1B)~~, with regional content of the repetitive elements varied from 34.76% to 84.89% in the *P. generosa* genome ~~(Figure 1B)~~, with peaks occurring at regions with low gene density (Figure 1B).

To evaluate the completeness of the assembly, the *P. generosa* ~~*P. generosa*~~ genome was tested using BUSCO~~assemblyies and annotated protein-coding gene~~PCGs ~~set were assessed using the BUSCO test~~ [25] with the metazoa_odb10 database (954 core genes)~~, respectively~~. ~~For the genome assembly, w~~We found that 93.0% of core PCGs~~genes~~ were identified as full-length in the *P. generosa* genome (Table 4), higher than that ~~compared to 91.5%~~ of ~~the closely related species~~ *S. constricta* (91.5%) [14] (Supplementary Table S6)~~,~~. We further tested the completeness of annotated ~~while. Regarding the gene set the~~ PCGs using BUSCO, which were aligned to~~,~~ 88.4% of the full-length core ~~genes were identified as full-length~~orthologs, suggesting that ~~the~~ quality of the ~~gene set~~annotation ~~could~~were ~~expected to~~can be ~~further~~ further improved in the future.

Chromosomal synteny analysis between *P. generosa* and *S. constricta*

~~Although the *P. generosa* genome has 19 chromosomes as many other species in the order Adapedota, chromosomal synteny between *P. generosa* and other species in this order remains unknown.~~ Comparative analysis of genome-wide gene collinearity~~between the genomes of *P. generosa* and *S. constricta*, whose genome has been assembled at the chromosome-level~~ revealed a high chromosome synteny between *P. generosa* and *S.*

Commented [D6]: Uhe used database in annotation should be clarified.

Formatted: Font: Italic

constricta. Of the 19 *P*., *generosa* chromosomes, 17 chromosomes were found to have one-to-one correspondences with 17 *S. constricta* chromosomes – that these two genomes have good chromosomal collinearity in general . when (Figure 2A). Oneone-to-one chromosomal correspondences between *P. generosa* and *S. constricta* were obviousobserved . (Figure 2A). However, Indeed, 17 of 19 *P. generosa* chromosomes showed clear one-to-one r correspondences *S. constricta* chromosomes (Figure 2A). However, majorBesides, several large-scale inter-chromosomal exchangesrearrangements were also evidentidentified, such as the arrangement among . For example, *P. generosa* chromosomes *Pg02* *Ppg02* and *Ppg11* matched to twoand – *S. constricta* chromosomes *Chr sc01* and *Chr sc10* (Figure 2). Despite these large-scale inter-chromosomal rearrangements, the numbers of chromosomes of these two species were both 19. *chromosomes (Chr1 and Chr10)*. Similarly, *P. generosa* Pg11 matched well to two *S. constricta* chromosomes (*Chr1* and *Chr10*).

In addition to these major inter-chromosomal exchange rearrangement events, comparative analysis of these two genomes also revealedthat extensive intra-chromosomal recombinationrearrangements events, which resulted inlittle co-linearity low gene synteny within chromosomesthese chromosomesregions. Instead of a clear diagonal linear relationship between genes of these two species *P. generosa* and *S. constricta*, a near random scattering of the relationships were observed (Figure 2A). These intra-chromosomal recombination rearrangement events were also clearly shown in Figure 2B, such as *P. generosa* chromosome *Pppg01* vs *S. constricta* chromosome *Chr sesc06*. .

Comparative analysis of gene families and eEvolutionary analysis of *P. generosa*

Formatted: Font: Italic

Formatted: Font: Italic

Formatted: Font: Italic

Formatted: Font: Italic

Formatted: Font: Italic

Formatted: Font: Italic

Formatted: Font: Italic

Formatted: Font: Italic

Formatted: Font: Italic

Formatted: Font: Italic

Formatted: Font: Italic

Formatted: Font: Italic

Formatted: Font: Not Italic

Commented [D7]: Tthe authors should describe which region of which chromosome, as well as the invovled length, underwent the rearrangement, not to tell the readers themselve to look for it in your figure.

Formatted: Font: Italic

Formatted: Font: Italic

Formatted: Font: Italic

Commented [D8]: Why no fossil calibration in estimating the divergence time ??? it should be used.

Commented [静王9R8]: The time correction points were taken from the Timetree website.

Formatted: Highlight

Formatted: Font: Italic

and other bivalves~~ of *P. generosa* and other bivalves~~

~~In total, 30,616 gene families were identified among *P. generosa* and 11 other species (*Pinctada martensi*, *C. gigas*, *Bathymodiolus* *B. platifrons*, *Patinopecten yessoensis*, *Pecten maximus*, *Argopecten purpuratus*, *Scapharca broughtonii*, *Homo sapiens*, *Xenopus tropicaalis*, *Danio rerio*, and *Caenorhabditis elegans*) (Table 5, Figure 3, Supplementary Table S8S7). As compared with the other 11 species, there were 7917 genes belonging to 1749 gene families that are specific to *P. generosa*. Comparative analysis of the genes of *P. martensi*, *S. broughtonii*, *P. yessoensis*, and *P. generosa* revealed 6490 common gene families shared by these species and 2902 gene families specific to *P. generosa* (Figure 4).~~ Phylogenetic analysis using 326 one-to-one single-copy orthologous genes ~~families from~~of these 12 species showed that *P. generosa* was tightly clustered with other bivalves as expected (Figure ~~5~~3). According to the phylogenetic tree, the divergence time of *P. generosa* from its nearest node, which represents the common ancestor of many other bivalves, was approximately 491.5 Mya (Figure ~~5~~3)~~). In addition, the divergence time of *P. generosa* is~~.. This divergence time between *P. generosa* and other bivalves was similar to the divergence time between ~~earlier than other bivalves~~ ~~but, which is~~ consistent with ~~that of~~ *S. constricta* and other ~~v~~bivalves [14]~~, a species close to *P. generosa*~~. Interestingly, the numbers of chromosomes of bivalves vary substantially, and closely related bivalves can have different numbers of chromosomes (Figure ~~5~~3). The oysters *C. gigas* with 10 chromosomes and *P. martensi* with 14 chromosomes diverged from the clam *S. broughtoni* and scallops with 19 chromosomes 409.4 MYA. What's more, the scallop *A. purpuratus* with 16 chromosomes diverged from another scallop *P. maximus*

Formatted: Font: Italic

Commented [D10]: Is it correct?? Annotation of too many species-specific gene clusters were caused by the wrong analysis or annotation. Are many repetitive element genes included in your annotations. It should be corrected.

Commented [静王11R10]: We have checked our analysis again. The PCG annotation was carried out shielding the repetitive elements. Besides, the 7917 species-specific genes or 1749 species-specific gene families are not unnormal. In this study, B. platifrons has 1,775 species-specific gene families. In other genome studies, P. yessoensis has 2,257 species-specific ones when compared with C. gagas and P. fucata (DOI:10.1038/s41559-017-0120).

Formatted: Highlight

Formatted: Highlight

with 19 chromosomes 61.7 MYA. ~~were clustered as a single clade and diverged from other bivalves with 19 chromosomes in the phylogenetic tree at 409.4??? Mya.~~

Comparative analysis of gene families

In total, 30,616 gene families were identified among *P. generosa* and 11 other species (*Pinctada martensi*, *C. gigas*, *B. platifrons*, *Patinopecten yessoensis*, *Pecten maximus*, *Argopecten purpuratus*, *Scapharca broughtonii*, *Homo sapiens*, *Xenopus tropicaalis*, *Danio rerio, and Caenorhabditis elegans*) (Table 5, Figure ~~3~~4, Supplementary Table S7). As compared with the other 11 species, 7917 genes belonging to 1749 gene families were found to be~~specific to~~ *P. generosa*-specific~~.~~, which fell into the range of 1567–15051 species-specific gene families identified in 12 bivalves [26]. Comparative analysis of the PCGs of *P. martensi*, *S. broughtonii*, *P. yessoensis*, and *P. generosa* revealed 6490 common gene families shared by these species and 2902 gene families specific to *P. generosa* (Figure ~~4~~5).

A total of 507 rapidly expanded gene families (involving 2,734 genes) and 875 rapidly contracted gene families (involving 792 genes) were identified in the *P. generosa* genome compared to the most recent common ancestor of both *P. generosa* and ~~the~~ other 11 species (Figure 6). The ~~KEGG analysis~~ annotation with the KEGG pathway database [27] revealed that the genes of expanded families were distributed in ~~166~~ 123 pathways, which ~~from the expanded gene families~~ were mainly enriched ~~with various biological processes (Supplementary Table S9) The enrichment analysis suggested that the significantly expanded genes of *P. generosa* were mainly represented~~ in organismal systems, genes associated with ~~human~~ diseases, ~~and~~

Commented [D12]: when??

Formatted: Highlight

Commented [D13]: Is it correct?? Annotation of too many species-specific gene clusters were caused by the wrong analysis or annotation. Are many repetitive element genes included in your annotations. It should be corrected.

Commented [静王14R13]: We have checked our analysis again. The PCG annotation was carried out shielding the repetitive elements. Besides, the 7917 species-specific genes or 1749 species-specific gene families are not unnormal. In this study, B. platifrons has 1,775 species-specific gene families. In a previous study, the species-specific gene family number was 1567-15051.

Commented [D15]: Can *P. generosa* have the human disease??

Commented [静王16R15]: The words have been corrected.

Formatted: Font color: Auto

environmental information processing~~, such as~~ (e.g. phototransduction), ~~Fluid~~ ~~fluid shear stress and atherosclerosis, ~~ and ~~P~~phosphatidylinositol signaling system, suggesting their important contribution to the adaptation of benthic bivalves.

~~Meanwhile, a~~According to the enriched KEGG pathways of expanded gene families in *P. generosa* (Supplementary Table ~~S9~~S8), there were a few significant enriched pathways (Q_value < 0.05) related to gonad development. For example, adrenergic signaling in cardiomyocytes, and glycine, serine and threonine metabolism which ~~were found related to~~ have been shown to function as part of spermatogenesis of the fluted giant clam *Tridacna squamosa* [28]. Moreover, oocyte meiosis, apoptosis, Ras signaling pathway, calcium signaling pathway, steroid hormone biosynthesis, GnRH signaling pathway, insulin signaling pathway, oxytocin signaling pathway, and ovarian steroidogenesis were documented to be enriched in *Procambarus clarkii* ovary development [29]. Geoducks have become a focus of significant aquaculture research and development with a considerable commercial value [30, 31]. The enriched gonad development-related pathways and genes could provide basic data for the further genetic breeding research of *P. generosa* and its closely related species.

We further compared gene families in different bivalves by searching for functional domains contained in PCGs in *P. generosa* and ~~8~~ eight other bivalves using InterProScan [32]. Examination of the top 65 most frequent domains ~~of gene families~~ that ~~presented~~ had specific expansions in *P. generosa* (Figure 7A) showed that the gene numbers of many important gene families were substantially expanded in *P. generosa*, including these containing the GIY-YIG catalytic domain (PF01541), the

Commented [D17]: Can *P. generosa* have that disease?? The KEGG annotation outputs include the information of all the speices. The authors should read the KEGG analysis outputs carefully and discard the items that cannot happen to your speices.

caspase recruitment domain (PF16739), the ApoA/ApoE domain (PF01442), and the copine domain (PF07002). In particular, the copy number of the copine gene family, which has been implicated in a range of cell signaling ~~and cytoskeletal proteins, targeted to the membrane following increases in cellular calcium~~ [33], ~~. Copy number of~~ ~~is~~ was 22 in ~~—~~, ~~.~~ *P. generosa* ~~has 22 copies of copine genes,~~ ~~has~~ twice as many ~~genes~~ as ~~compared to~~ those identified ~~other species~~ ~~(22 in *P. generosa* to 11~~ in *S. constricta* ~~)~~. Examination of positions of ~~. In *P. generosa*, the number of genes of the copine gene family was 22, comparing to 11 copine genes in *S. constricta*. T~~ the ~~22 copine genes in~~ *P. generosa* copine genes revealed that they were distributed ~~were found in multiple chromosomes including~~ in chromosomes ~~*Pg02*~~ *pg02*, ~~*Pg05*~~ *pg05*, ~~*Pg07*~~ *pg07*, ~~*Pg10*~~ *pg10*, ~~*Pg11*~~ *pg11*, and ~~*Pg17*~~ *pg17* (Figure 7B). Interestingly, many genes formed local clusters (e.g. ~~8~~ eight copies in Pg11), suggesting that the large copine gene set observed in *P. generosa* might have been achieved via tandem duplication of the copine genes in recent evolution. ~~For example, eight genes were located in a single cluster in *Pg11* (Figure 7B).~~

Phylogenetic analysis of the copine genes annotated in *P. generosa* (22 genes) and *S. constricta* (11 genes) revealed good orthologous relationships, as well as one-to-multiple relationships (Figure 7C), confirming that genes inside copine gene clusters in *P. generosa* (Figure 7B) were highly similar.

Most of the ~~22~~ copine ~~genes~~ homologs ~~in *P. generosa*~~ contain the vWA-domain, while some has both C2-domain and the vWA-domain in *P. generosa* (Figure 7D) like ~~as for~~ copine genes identified in other species [33]. Lengths of ~~S~~some ~~copine genes~~ ~~copies~~ ' the

Commented [D18]: Add the references for the function information of copine domain?

Formatted: Font: Italic
Formatted: Font: Not Italic
Formatted: Font: Italic
Formatted: Font: Not Italic

Commented [D19]: Don't italicize the chromosome number. Theck all the maintext and correct them.

Commented [静王20R19]: These chromosome number presents the chromosome nucleotide sequences. Therefore, we italicized them.

Formatted: Font: Italic
Formatted: Font: Not Italic
Formatted: Font: Italic
Formatted: Font: Not Italic
Formatted: Font: Italic
Formatted: Font: Not Italic
Formatted: Font: Italic

coding sequences of some copies ~~were~~ w~~as~~ere comparatively short, which might be due to errors of ~~imperfect~~ genome assembly or gene prediction.~~annotation of these genes.~~ Prediction of 3D structures ~~of these candidate copine genes in *P. generosa*~~ using AlphaFold2 [34] revealed that their structures are highly ~~similar~~ conserved ~~to those copine genes~~among different ~~identified in other~~ species, suggesting the conservation of protein functions. For example, the three-dimensional structure of ~~*Pg02g00048*~~Pg02g00048 in *P. generosa* showed high similarity to that of ~~*XP_0533888641*~~XP_0533888641 in *Mercenaria mercenaria* [35] (Figure 7E).

Discussion

Through the completion of this project, we have successfully ~~construction~~ constructed ~~of~~ the first high-quality chromosome-level genome assembly of the ecologically and economically important bivalves the Pacific geoduck *P. generosa*, enriching the expanding list of chromosome-level genomes of bivalves ~~with cutting-edge genomic technologies.~~. The assembled *P. generosa* genome ~~size~~ consists of 19 chromosomes with a genome size of 1.47 Gb, and a contig N50 of 1.6 Mb. The *P. generosa* genome represents the third genome of the third species in the order Adapedonta, after *S. constricta* and *Solen grandis*. The *P. generosa* genome assembly represented another instance of ~~o date, the~~ chromosome-level genome~~s~~s of the ~~more than 40~~ bivalve species [11]~~have been assembled~~, an ancient lineage of bilaterian, and a diverse Class of Mollusca (Supplementary Table S1).

The assembled *P. generosa* genome size (1.47 Gb) fell within the range of the reported genomes of bivalves, which varied from 543.9 Mb in *Lutraria thynchaena* [12] to 2.6 Gb in

Commented [D21]: Don't italicize it.

Commented [D22]: Don't italicize it.

Commented [D23]: The discussion section is the worst part of this manuscipt when the authors keep repeating the information the result section have mentioned. The discussion part should be re-written by adding more extended thoughts and information that the result section not mensioned or not deeply mentioned.

Formatted: Font: Italic

Modiolus philippinarum [13] (Supplementary Table S1). The assembled *P. generosa* genome size (1.47 Gb) also fell within important insights into its genetic makeup and evolution have been gained. The assembled *P. generosa* genome size consists of 19 chromosomes with a genome size of 1.47 Gb, and a contig N50 of 1.6 MbTable and 19 chromosomes. The assembled genome sizes of bivalves vary widely, ranging from 543.9 Mb in *Lutraria thynchaena* [12] to 2.6 Gb in *Modiolus philippinarum* [13] (Supplementary Table S1). Among bivalves, the genome size ranges of most superorder Imparidentia species ranged from (1 Gb to 1.8 Gb), and the genome size range of that of the species in the order Adapedonta species (1 Gb to 1.5Gb), which includes *P. generosa*, ranged from 1 Gb to 1.5 Gb [14].

Although both the two genomes have the exactly same number of 19 chromosomes, comparative analysis between the genomes of *P. generosa* and its most closely related species *S. constricta* revealed extensive inter- and intra-chromosomal exchanges. Large chromosomal fragments of *P. generosa pg02* and *pg11* matched to two *S. constricta* chromosomes (*sc01* and *sc10*), respectively (Figure 2). Despite such large inter-chromosomal exchanges, the numbers of chromosomes of both *P. generosa* and *S. constricta* were identical. Within chromosomes, the order of genes showed even more extensive alterations, resulting in the lack of clear diagonal alignments (Figure 2). Among bivalves the numbers of chromosomes vary substantially, suggesting active genome recombination during the evolution of bivalves [15]. The chromosome numbers of bivalves in the Infraclass Heteroconchia are 16–19, relatively higher than those in the Infraclass Pteriomorphia which are 10–19. In the Infraclass Pteriomorphia most oysters and scallops

possess 19 chromosomes, similar to those of most clams in the Infraclass Heteroconchia. In the phylogenetic analysis of the Infraclass Pteriomorphia the blood clam was closer to the scallops, compared with oysters (Figure 3). What is interesting, the Manila clam *R. philippinarum* was clustered in the clade of the Infraclass Pteriomorphia (Figure 3).

A total of 35,034 PCGs were predicted in the *P. generosa* genome, of which 30,700 genes (87.63%) were functionally annotated, supporting the high-quality annotation of the *P. generosa* genes, and the conservation of PCGs in bivalves. *P. generosa* is a highly complex species with a heterozygosity of 1.37% and 57.9968.08% repeat sequences in genome.

The availability of the first high-quality chromosome-level *P. generosa* genome assembly enabled us to ascertain its genomic compositions, which in turn facilitate comparative analysis with other bivalve genomes. The *P. generosa* genome represents the third genome of the third species in the order Adapedonta, after *S. constricta* and *Solen grandis*. Although all the three genomes have the exactly same number of 19 chromosomes, comparative analysis between the genomes of *P. generosa* and its most closely related species *S. constricta* revealed extensive inter- and intra-chromosomal exchanges. Large chromosomal fragments of *P. generosa* *Pg02* *pg02* and *Pg11* *pg11* matched to two *S. constricta* chromosomes (*Chr1* *sc1* and *Chr10sc10*) and two *S. constricta* chromosomes (*Chr1* and *Chr10*), respectively (Figure 2). It is rather surprising thatDespite such large inter-chromosomal exchanges, did not lead to the change ofthe numbers of chromosomal chromosomes numbers of

either both P. *generosa* or and *S. constricta* did not change. Within chromosomes, the order of genes showed even more extensive alterations, resulting in the lack of clear diagonal alignments (Figure 2).

Through genome annotation, which identified 35,034 PCGs were predicted in the *P. generosa* genome, and cComparative analysis revealed thatomparative analysis,

In addition to the changes of organization of chromosomes and order of genes in the *P. generosa* and its closely related species *S. constricta*, the numbers of PCG members in of many gene families with important functions also changes substantially in evolution (Figure 7A). In particular, the copine gene family was found to be substantially expanded in *P. generosa*, with 22 copine genes were identified in *P. generosa*.. The number of copine genes in *P. generosa* representing was –twice of those in *P. constricta*. Many of these 22 copine genes in *P. generosa* formed tandem clusters, with one cluster containing eight copine genes (Figure 7B). The copine genes within each cluster showed high similarity (Figure 7C), further confirming, suggesting that these genes were formed via tandem duplications. The composition of functional domains, which include Copine genes in *P. generosa* contain C2 domain and vWA-domain (Figure 7D), and the similarity of three-dimensional structure to that of . Modeling with AlphaFold2 revealed that copines encoded by the *P. generosa* copine genes show high structural similarity with that encoded by known copine genes (Figure 7E) suggests functional conservation of function of copine genes in *P. generosa*. Thus, the completion of *P. generosa* facilitates genome comparative analysis of gene families to uncovered important leads for exploring molecular insight into its physiology and evolution.

Formatted: English (Canada)

Commented [D24]: I don't konw whether there is diference between this paragraph and the corresponding pargraph in the result section.

~~Taken together, this study provides t~~The successful construction of *P. generosa* chromosome-level genome not only enables genomic identification and analysis of important genes in this organism, but also enables comparative analysis of bivalve genomes, which is critical for tracking the species formation, evolution, and biodiversity of bivalves. ~~first high-quality genomic resource that will support future phylogenetic, evolution, and immunological studies. Additionally, it will support the genetic breeding of geoducks.~~

Commented [D25]: The conclusive description is not simply to repeat the above-mentioned information. The similar sentenses can be found several times in this maintext. This conclusion paragraph should be re-written.

Methods

Sampling collection

Geoduck *P. generosa* samples were collected from the Strait of Georgia (49°41′12″N, 124°51′33″W) of British Columbia, Canada in the spring of 2019. The samples showed typical morphological features of *P. generosa*. The identification of the samples was also supported by the high similarities of the molecular marker *cox1* to the reference sequence of *P. generosa* (PID of 99.55, coverage of 100%) [36]. The samples- were transferred to laboratory and kept in a tank with running water for a week. One sample was chosen and dissected on ice to collect tissue samples, including labial palp, heart, foot, gonad, gill, hepatopancreas, siphon, and mantle muscle. This animal was identified to be a female as indicated by the presence of eggs in the smear of the gonad under a compound microscope. Dissected tissues were quickly frozen in liquid nitrogen and then stored at −80°C before DNA and RNA extraction.

DNA library construction and sequencing

Genomic DNA of *P. generosa* was extracted using a standard phenol-chloroform extraction method [37]. The quality of DNA was determined by gel electrophoresis to

ensure the DNA samples met library sequencing requirements. Sequence libraries with insert size of 300 bp were constructed for BGISEQ-500 sequencing platform (RRID:SCR_017979) according to manufacturer's protocol. The sequencing data produced were used in the genome size estimation by *k*-mer analysis [21] and for correcting errors in the Pilon (RRID:SCR_014731) assembly [38]. A Hi-C library with insert size of 300 bp was constructed to provide long-range information (without position information) on the grouping and linear organization of sequences along entire chromosomes to assemble the scaffolds into chromosome-level scaffolds [39]. For Hi-C library construction, gonad tissue was dissociated, and cells were collected and crosslinked with 1% formaldehyde (Sigma) and 0.2M glycine (Sigma). After that, the fixed powder was resuspended in nuclei isolation buffer and then incubated in 0.5% SDS for 10 min at 62°C. Then the reaction was quenched with 10% Triton X-100 (Sigma) and the nuclei were collected by centrifugation. Then the DNA was digested with MboI (NEB), and the overhang was filled and biotinylated before ligated by T4 DNA ligase (NEB). Before library construction, the purified DNA was sheared, and biotin-containing fragments were captured on streptavidin-coated beads using Dynabeads MyOne Streptavidin T1 (Invitrogen). The fragments were then end-repaired and linked with adaptors before eight cycles of PCR reaction with KAPA HiFi HotStart ReadyMix (Kapa Biosystem). After that, the Hi-C library was sequenced with BGISEQ-500 platform. And a PacBio library with insert size of 20 Kb was constructed to obtain long reads by the PacBio Sequel platform using the Sequel Sequencing Kit 3.0. The adapters and low-quality reads in raw data generated by the BGISEQ platform were cut off by SOAPnuke1.5.6 using the parameter as "-n 0.01

-l 20 -q 0.1 -i -Q 2 -G -M 2 -A 0.5 -d" [40]. PacBio raw data were filtered with the default parameters by using Pacific Biosciences SMRT analysis software (v2.3.1) to filter the low quality reads.

RNA library construction and sequencing

RNA-Seq and Iso-Seq were conducted to obtain transcriptome data to aid genome annotation. The total RNAs was extracted by Trizol (Invitrogen, Carlsbad, CA, USA) from eight tissues of the same *P. generosa* individual, including labial palp, heart, foot, gonad, gill, hepatopancreas, siphon, and mantle muscle. The quality and quantity of RNA in each sample was assessed using a NanoDrop and an Agilent 2100 bioanalyzer (Thermo Fisher Scientific, MA, USA). The construction of mRNA libraries for RNA-Seq, the mRNA was enriched by mRNA Capture Beads (BGI, LB00V60), and incubated at 85°C for 8 minutes for fragmentation. Reverse transcription was performed with Strand Specificity Reagent and 1st Strand Enzyme Mix (Optimal Dual-mode mRNA Library Prep Kit, BGI, LR00R96) to generate the first strand cDNA. After that the second strand cDNA generation and end repair were performed with 2nd Strand Buffer and 2nd Strand Enzyme Master Mix. Then the adaptors (BGI, LA00R04) were ligated to the cDNAs. Then the library was purified and selected depending upon product requirements for amplification. The mRNA libraries were sequenced using the BGISEQ-500 platform. For Iso-Seq, the total RNA was extracted from the equally mixed tissues of the 8 tissues above. The PacBio SMRTbell library was prepared using the SMARTer PCR cDNA Synthesis kit (Clontech), the Qubit dsDNA HS Assay Kit 2.0 (Invitrogen) and the Agilent DNA 12000 kit (Agilent Technologies), and sequenced by the PacBio Sequel sequencer (RRID:SCR_017989) with Sequel

Sequencing Kit 3.0.

Genome size estimation and genome assembly

Genome size of *P. generosa* was estimated using *k*-mer analysis. Counting of *k*-mers was conducted using Jellyfish (RRID:SCR_005491, version 2.2.10) [21]. The genome size, heterozygosity, and repeat content were estimated using GCE 1.0.2 [41]. For genome assembly, long reads generated from PacBio Sequel platform were assembled using Falcon (RRID:SCR_016089) [42], which was subsequently polished using Arrow. Short paired-end clean reads from BGISEQ-500 were then used for correcting post-processing errors and resolving conflicts of assembly via Pilon (RRID:SCR_014731, version 1.22) [38]. The assembled contigs were corrected for mis-joins, orders, orients and anchored contigs from the draft assembly into a candidate chromosome-length assembly by Hi-C data using Juicer (RRID:SCR_017226) [43] and 3d-DNA [44]. The scaffolds shorter than 20 Kb were removed. Finally, the candidate assembly were reviewed with Juice~~r~~box Assembly Tools (RRID:SCR_021172) for quality control and interactive corrections [45]. The Hi-C heatmap was visualized using Juicebox (RRID:SCR_021172) ~~presenting the counts of paired reads which each two bins aligned~~ (with the bin length of 100 kb) as the interactive signals between each pair of two bins. The completeness of genome assembly was assessed by BUSCO (RRID:SCR_015008, version5.4.3) [25]~~.~~ using the metazoa_odb10 database. The genome landscape illustrating the length, repeat element density, gene density and GC content was created by circos-0.69-9 (RRID:SCR_011798) [46].

Annotations of gene structure and function

Homologous and *de novo* predictions were both applied to annotate transposable

elements in the *P. generosa* genome. In homologous prediction, RepeatMasker (RRID:SCR_012954) and RepeatProteinMask [47] were used to screen the *P. generosa* genome for known transposable elements in the RepBase library (RRID:SCR_021169) [48]. In *de novo* prediction, RepeatModeler (version 1.0.4) was first used for de novo candidate database construction of repetitive elements, and repetitive sequences were then annotated using RepeatMasker. Tandem repeats were de novo predicted using Tandem repeats finder (version 4.07) [49]. The results were then integrated and duplicates were eliminated.

Three complementary approaches were adopted to predict PCGs in *P. generosa* genome, including homology-based prediction, *de novo* annotation, and transcriptome-based prediction. For homology-based prediction, gene sets from eight closely related bivalves (*P. yessoensis, P. fucata, Mytilus galloprovincialis, Limnoperna fortune, A. purpuratus, S. constricta, S. broughtonii, and C. gigas*) were used. First, protein repertoires of those organisms were aligned against the *P. generosa* genome using TBLASTN (RRID:SCR_011822) [50]. Then gene structures were predicted from these blast hits by Exonerate v2.2.0 [51]. *de novo* gene prediction was performed using a combination of Augustus (RRID:SCR_008417) [52] and SNAP (RRID:SCR_007936) [53] with default settings. Models used for each gene predictor Augustus and SNAP training were obtained from a set of high-quality proteins generated from the RNA-Seq and ISO-seq dataset by MAKER 2 (RRID:SCR_005309). For transcriptome-based prediction using RNA-Seq data, RNA-Seq reads were directly mapped to the genome using TopHat2 (RRID:SCR_013035) [54]. The mapped reads were subsequently assembled into gene models (Cufflinks-set) by

Cufflinks (RRID:SCR_014597) [55]. For transcriptome-based prediction based on Iso-Seq data, Iso-Seq reads were directly mapped to the genome using GMAP (RRID:SCR_008992) [56]. The mapped reads were subsequently assembled by PASA (RRID:SCR_014656) [57]. Gene predictions from the homology-based approach, *de novo* approach, RNA-Seq-based and Iso-Seq-based evidences were merged, and redundancy was removed to form a comprehensive consensus gene set using Maker 2 (RRID:SCR_005309) [58]. To validate the completeness of the gene structure annotation, we also used BUSCO (version5.4.3) with the metazoa_odb10 database [25].

Collinearity analysis

Homologous PCGs in *P. generosa* and *S. constricta* were identified using BLAST v2.11.0 (blastp, E value $1e^{-5}$), which were used for subsequent analysis using WGDI [59]. WGDI analysis results included dotplot and syntenic blocks with default parameters.

Formatted: Indent: Left: 0"

Phylogenetic analysis and divergence time estimation

Gene families were constructed using the OrthoMCL (RRID:SCR_007839) pipeline [53]. We selected *P. generosa* and other 11 species (*C. gigas*, *P. yessoensis*, *P. maximus*, *A. purpuratus*, *S. broughtonii*, *P. martensi*, *B. platifrons*, *H. sapiens*, *X. tropicaalis*, *D. rerio*, and *C. elegans*) for gene family analysis. For the gene set of each genome, only the transcript with the longest coding sequence was selected from alternate splice transcripts. Genes with less than 50 amino acids were removed from further analysis. Protein sequences were aligned by “all-vs-all BLASTP” (E value = $1e^{-5}$) [44]. Then the Markov clustering (MCL) algorithm implemented in OrthoMCL was used to group orthologues and

paralogues from all input species with an inflation value of 1.5 [60].

The phylogenetic tree was constructed following procedures described in previous studies [14, 35, 61]. Briefly, for phylogenetic tree construction and divergence time estimation, shared single copy genes of *P. generosa* and 11 other species were used. The protein sequences of single-copy orthologs among the 12 species were aligned using MUSCLE v3.7 (RRID:SCR_011812) [62] with default parameters. Phylogenetic relationships were inferred based on the super-matrix estimated from the concatenated alignment of single-copy genes using the maximum likelihood (ML) [63] method implemented in RAxML v2.2 (RRID:SCR_006086) [64] with the optimal amino acid substitution model selected by the PROTGAMMALGX parameter.

Based on gene family identification and phylogenetic analysis, single copy genes and mcmctree in PAML [65] were used to estimate divergence time [66-69]. The time correction points were *C. elegans* and *H. sapiens* (678.3–855.2 MYA), *D. rerio* and *H. sapiens* (413.1–443.0 MYA), *X. tropicalis* and *H. sapiens* (347.0–357.9 MYA). The time correction points were taken from the Timetree website. The operating parameters of mcmctree: burn in = 10000, sample number = 1000000, sample frequency = 50.

Gene family ~~expansion and contraction~~analysis and three dimensional protein structure modeling

The clustering results of gene families and the phylogenetic tree with divergence time estimated were used to analyze the expansion and contraction of orthologous gene families between ancestor and each of the 12 species (*P. generosa* and the other 11 species) using a stochastic birth and death model with lambda parameter by CAFE

(RRID:SCR_005983, version 4.0) [70]. This model was further used to calculate the number of gene families along each lineage on the phylogenetic tree. A probabilistic graphical model was introduced to calculate the probability of transitions in gene family size from parent to child nodes. The family-wide P-Values were calculated in each lineage based on the conditional likelihood.

In addition, we also compared the gene families between *P. generosa* and the other eight bivalves. Pfam domains of *P. generosa* and the other eight bivalves were obtained using InterProscan [32] (5.54-87.0) and visualized using TBtools (v1.120) [71]. The positions of copine genes in *P. generosa* chromosomes were illustrated using R packages gggenes, and the structure of copine genes were illustrated using Gene Structure Display Server 2.0 on line [72]. Phylogenetic trees of copine genes of *P. generosa* and *S. constricta* were constructed using the maximum likelihood (ML) [63] method implemented in MEGA (v7.0.26) with 1000 bootstrap replicates.

Three-dimensional protein structure models were predicted using AlphaFold2 and visualized using ChimeraX [34].

Data Availability

The Whole Genome project of *P. generosa* has been deposited at NCBI/BioProject PRJNA859289. The raw next-generation sequencing reads of DNA are available at SRA (SRR22190027-SRR22190030); raw long-read PacBio sequencing reads of DNA are available at SRA (SRR22190026; raw next-generation sequencing reads of RNA are available at SRA (SRR22190032); raw Hi-C reads are available at SRA (SRR22190025); and raw long-read PacBio sequencing reads of RNA are available at SRA(SRR22190031).

The genome assembly data have been deposited under accession No. JAPMAH000000000.1.

Additional Files

Supplementary Figure S1: The 17-mer count distribution for the genome size estimation.

Supplementary Figure S2: The enriched KEGG pathways of significantly expanded gene families (p≤0.01, top 20) in *P. generosa*

Supplementary Table S1: The genome assembly information of bivalves in the public database

Supplementary Table S2: Statistics of 17-mer analysis

Supplementary Table S3: The chromosomes information of *P. generosa* ~~The heterozygosity of bivalves reported~~

Supplementary Table S4: The heterozygosity of bivalves reported ~~The chromosomes information of *P. generosa*~~

Supplementary Table S5: Functional annotation of the predicted protein-coding genes in *P. generosa* genome assembly ~~The structural statistics of gene prediction in *P. generosa*~~

Supplementary Table S6: The assembly quality of chromosomal genomes of bivalves by BUSCO ~~The assembly quality of chromosomal genomes of bivalves by BUSCO~~

Supplementary Table S7: The information of 30,616 gene families of *P. generosa* and 11 other species ~~Functional annotation of the predicted protein-coding genes in *P. generosa* genome assembly~~

Supplementary Table S8: The enriched KEGG pathways of expanded gene families in *P.*

generosa genome assembly The information of 30,616 gene families of *P. generosa* and 11 other species

Supplementary Table S9: The enriched KEGG pathways of expanded gene families in *P. generosa* genome assembly

Abbreviations

PCG: protein-coding gene; Akt: RAC serine/threonine-protein kinase; bp: base pairs; BLAST: Basic Local Alignment Search Tool; BUSCO: Benchmarking Universal Single-Copy Orthologs; BWA: Burrows-Wheeler Aligner; cAMP: cyclic adenosine monophosphate; CAMs: cell adhesion molecules; cGMP-PKG: cGMP-dependent protein kinase G; Gb: gigabase pairs; GC: guanine-cytosine; GnRH: Gonadotropin-releasing hormone; GO: gene ontology; Hi-C: High-throughput/resolution chromosome conformation capture; kb: kilobase pairs; KEGG: Kyoto Encyclopedia of Genes and Genomes; Mb: megabase pairs; MYA: million years ago; NCBI: National Center for Biotechnology Information; NF-kappa B: nuclear factor kappa-B; NOD-like receptor: nucleotide-binding oligomerization domain-like receptor; PacBio: Pacific Biosciences; RAxML: Randomized Axelerated Maximum Likelihood; PI3K: phosphatidylinositol-4,5-bisphosphate 3-kinase catalytic subunit alpha/beta/delta; Rap1: Ras-related protein1; RNA-Seq: RNA sequencing; Iso-Seq: Isoform-sequencing; tRNA: transfer RNA; TRP channel: transient receptor potential ion channel.

Competing Interests

The authors declare that they have no competing interests.

Funding

This study was supported by the Taishan Scholar Project Special Fund (to Nansheng Chen), the Strategic Priority Research Program of Chinese Academy of Sciences (XDB42000000), the Chinese Academy of Sciences Pioneer Hundred Talents Program (to Nansheng Chen), and an Earmarked Workstation Fund for QRJH (to Chunde Wang and Nansheng Chen).

Authors' Contributions

N.C. and C.W. conceived and designed the study. M.C. and Y.C. prepared the samples. J.W. and Q.X. performed analyses. J.W. wrote the paper with input from co-authors. All authors read and approved the final version for submission.

References

1. González-Peláez SS, Leyva-Valencia I, Pérez-Valencia SA, et al. Distribution limits of the geoduck clams *Panopea generosa* and *P. globosa* on the Pacific coast of Mexico. *Malacologia* 2013;**56**:85-94.
2. Vadopalas B, Pietsch TW, Friedman CS. The proper name for the geoduck: resurrection of *Panopea generosa* Gould , 1850, from the synonymy of *Panopea abrupta* (Conrad, 1849) (Bivalvia: Myoida: Hiatellidae). *Malacologia* 2010;**52**:169-73.
3. Goodwin CL, Pease BC. Geoduck, *Panopea abrupta* (Conrad, 1849), size, density, and quality as related to various environmental parameters in Puget Sound, Washington. *J Shellfish Res* 1991;**10**:65-77.
4. Orensanz JM, Hand CM, Parma AM, et al. Precaution in the harvest of Methuselah's clams the difficulty of getting timely feedback from slow-paced dynamics. *Can J Fish Aquat Sci* 2004;**61**:1355-72.
5. Newell RIE. Ecosystem influences of natural and cultivated populations of suspension-feeding bivalve molluscs: A review. *J Shellfish Res* 2004;**23**:51-61.
6. Straus KM, MacDonald PS, Crosson LM, et al. Effects of geoduck aquaculture on the environment: A synthesis of current knowledge. Washington Sea Grant Technical Report WSG-TR 13-02, 2013.
7. Santos A, Aguirre J, Rodríguez-Tovar FJ, et al. Multi-storm events recorded on *Panopea* burrows (Pliocene, Spain): The importance of sequestered information inside burrows. *Palaeogeogr Palaeoclimatol Palaeoecol* 2018;**507**:155-67.
8. Bureau D., Hajas W., Hand C.M., et al. Age, size structure and growth parameters of geoducks (*Panopea abrupta*, Conrad 1849) from seven locations in British Columbia sampled in 2001 and 2002. Canadian Technical Report of Fisheries and Aquatic Sciences, 2003, p. 29.
9. Valero JL, Canada O, Madryn P, et al. Geoduck (*Panopea abrupta*) recruitment in the Pacific Northwest: long-term changes in relation to climate. *CalCOFI Reports* 2004;**45**:80-6.
10. Sloan NA, Robinson SMC. Age and gonad development in the geoduck clam *Panopea abrupta* (Conrad) from southern British Columbia, Canada. *J Shellfish Res* 1984;**4**:131-7.
11. Liu F, Li Y, Yu H, et al. MolluscDB: an integrated functional and evolutionary genomics database for the hyper-diverse animal phylum Mollusca. *Nucleic Acids Res* 2020;**49**:D988-D997.
12. Thai BT, Lee YP, Gan HM, et al. Whole genome assembly of the snout otter clam, *Lutraria rhynchaena*, using Nanopore and Illumina Data, benchmarked against bivalve genome assemblies. *Front Genet* 2019;**10**:1158.
13. Sun J, Zhang Y, Xu T, et al. Adaptation to deep-sea chemosynthetic environments as revealed by mussel genomes. *Nat Ecol Evol* 2017;**1**:0121.
14. Ran Z, Li Z, Yan X, et al. Chromosome-level genome assembly of the razor clam *Sinonovacula constricta* (Lamarck, 1818). *Mol Ecol Resour* 2019;**19**:1647-58.
15. Halanych KM, Kocot KM. Genome evolution: Shellfish genes. *Nat Ecol Evol* 2017;**1**:0142.

16. Peñaloza C, Gutierrez AP, Eöry L, et al. A chromosome-level genome assembly for the Pacific oyster *Crassostrea gigas*. *GigaScience* 2021;**10**:giab020.
17. Peng J, Li Q, Xu L, et al. Chromosome-level analysis of the *Crassostrea hongkongensis* genome reveals extensive duplication of immune-related genes in bivalves. *Mol Ecol Resour* 2020;**20**:980-94.
18. Wu B, Chen X, Yu M, et al. Chromosome-level genome and population genomic analysis provide insights into the evolution and environmental adaptation of Jinjiang oyster *Crassostrea ariakensis*. *Mol Ecol Resour* 2021;**22**:1529-44.
19. Du X, Fan G, Jiao Y, et al. The pearl oyster *Pinctada fucata martensii* genome and multi-omic analyses provide insights into biomineralization. *GigaScience* 2017;**6**:1-12.
20. Yang JL, Feng DD, Liu J, et al. Chromosome-level genome assembly of the hard-shelled mussel *Mytilus coruscus*, a widely distributed species from the temperate areas of East Asia. *GigaScience* 2021;**10**:giab024.
21. Marçais G, Kingsford C. A fast, lock-free approach for efficient parallel counting of occurrences of k-mers. *Bioinformatics* 2011;**27**:764-70.
22. Gomes-Dos-Santos A, Lopes-Lima M, Machado AM, et al. The Crown Pearl: a draft genome assembly of the European freshwater pearl mussel *Margaritifera margaritifera* (Linnaeus, 1758). *DNA Res* 2021;**28**:1–10.
23. Zhang G, Fang X, Guo X, et al. The oyster genome reveals stress adaptation and complexity of shell formation. *Nature* 2012;**490**:49-54.
24. Zhang T, Yin J, Tang S, et al. Dissecting the chromosome-level genome of the Asian Clam (*Corbicula fluminea*). *Sci Rep* 2021;**11**:15021.
25. Simão FA, Waterhouse RM, Ioannidis P, et al. BUSCO: assessing genome assembly and annotation completeness with single-copy orthologs. *Bioinformatics* 2015;**31**:3210-2.
26. Teng W, Xie X, Nie H, et al. Chromosome-level genome assembly of *Scapharca kagoshimensis* reveals the expanded molecular basis of heme biosynthesis in ark shells. *Mol Ecol Resour* 2022;**22**:295-306.
27. Kanehisa M, Goto S, Kawashima S, et al. The KEGG resource for deciphering the genome. *Nucleic Acids Res* 2004;**32**:D277-D280.
28. Li J, Zhou Y, Zhou Z, et al. Comparative transcriptome analysis of three gonadal development stages reveals potential genes involved in gametogenesis of the fluted giant clam (Tridacna squamosa). *BMC Genomics* 2020;**21**:872.
29. Jiang H, Liu H, Ma X, et al. Transcriptome analysis of *Procambarus clarkii* to screen genes related to ovary development,immunity and growth. *J Fish Chn* 2021;**45**:396-414.
30. Ren Y, Liu W, Pearce CM, et al. Effects of selected mixed-algal diets on growth and survival of early postset juveniles of the Pacific geoduck clam, *Panopea generosa* (Gould, 1850). *Aquac Nutr* 2015;**21**: 152-61.
31. Nava-Gómez GE, Garcia-Esquivel Z, Carpizo-Ituarte E, et al. Survival and growth of geoduck clam larvae (*Panopea generosa*) in flow-through culture tanks under laboratory conditions. *Aquac Res* 2018;**49**:294-300.
32. Zdobnov EM, Apweiler R. InterProScan--an integration platform for the signature-recognition methods in InterPro. *Bioinformatics* 2001;**17**:847-8.

33. Perestenko PV, Pooler AM, Noorbakhshnia M, et al. Copines-1, -2, -3, -6 and -7 show different calcium-dependent intracellular membrane translocation and targeting. *FEBS J* 2010;**277**:5174-89.
34. Jumper J, Evans R, Pritzel A, et al. Highly accurate protein structure prediction with AlphaFold. *Nature* 2021;**596**:583-9.
35. Song H, Guo X, Sun L, et al. The hard clam genome reveals massive expansion and diversification of inhibitors of apoptosis in Bivalvia. *BMC Biology* 2021;**19**:15.
36. Bisbal-Pardo CI, Del Río-Portilla MA, Rocha-Olivares A. The complete mitochondrial DNA of the Pacific Geoduck clam (*Panopea generosa*). *Mitochondrial DNA A DNA Mapp Seq Anal* 2016;**27**:1955-6.
37. Green M, J S. Molecular cloning: a laboratory manual. 4th Edn. Vol. II. New York, NK: Cold Spring Harbor Laboratory Press.
38. Walker BJ, Abeel T, Shea T, et al. Pilon: an integrated tool for comprehensive microbial variant detection and genome assembly improvement. *PloS One* 2014;**9**:e112963.
39. Burton JN, Adey A, Patwardhan RP, et al. Chromosome-scale scaffolding of de novo genome assemblies based on chromatin interactions. *Nat Biotechnol* 2013;**31**:1119-25.
40. Chen Y, Chen Y, Shi C, et al. SOAPnuke: a MapReduce acceleration-supported software for integrated quality control and preprocessing of high-throughput sequencing data. *GigaScience* 2018;**7**:1-6.
41. Liu B, Shi Y, Yuan J, et al. Estimation of genomic characteristics by analyzing *k*-mer frequency in de novo genome projects. *arXiv: Genomics* 2013.
42. Pendleton M, Sebra R, Pang AW, et al. Assembly and diploid architecture of an individual human genome via single-molecule technologies. *Nat Methods* 2015;**12**:780-6.
43. Durand NC, Shamim MS, Machol I, et al. Juicer provides a one-click system for analyzing loop-resolution Hi-C experiments. *Cell Syst* 2016;**3**:95-8.
44. Dudchenko O, Batra SS, Omer AD, et al. De novo assembly of the *Aedes aegypti* genome using Hi-C yields chromosome-length scaffolds. *Science* 2017;**356**:92-5.
45. Robinson JT, Turner D, Durand NC, et al. Juicebox.js provides a cloud-based visualization system for Hi-C data. *Cell Syst* 2018;**6**:256-8.
46. Krzywinski M, Schein J, Birol I, et al. Circos: an information aesthetic for comparative genomics. *Genome Res* 2009;**19**:1639-45.
47. Bergman CM, Quesneville H. Discovering and detecting transposable elements in genome sequences. *Brief Bioinform* 2007;**8**:382-92.
48. Bao W, Kojima KK, Kohany O. Repbase Update, a database of repetitive elements in eukaryotic genomes. *Mobile DNA* 2015;**6**:11.
49. Benson G. Tandem repeats finder: a program to analyze DNA sequences. *Nucleic Acids Res* 1999;**27**:573-80.
50. Altschul SF, Gish W, Miller W, et al. Basic local alignment search tool. *J Mol Biol* 1990;**215**:403-10.
51. Slater GS, Birney E. Automated generation of heuristics for biological sequence comparison. *BMC Bioinformatics* 2005;**6**:31.
52. Stanke M, Morgenstern B. AUGUSTUS: a web server for gene prediction in eukaryotes

that allows user-defined constraints. *Nucleic Acids Res* 2005;**33**:W465-W467.
53. Johnson AD, Handsaker RE, Pulit SL, et al. SNAP: a web-based tool for identification and annotation of proxy SNPs using HapMap. *Bioinformatics* 2008;**24**:2938-9.
54. Kim D, Pertea G, Trapnell C, et al. TopHat2: accurate alignment of transcriptomes in the presence of insertions, deletions and gene fusions. *Genome Biol* 2013;**14**:R36.
55. Trapnell C, Roberts A, Goff L, et al. Differential gene and transcript expression analysis of RNA-seq experiments with TopHat and Cufflinks. *Nat Protoc* 2012;**7**:562-78.
56. Wu TD, Watanabe CK. GMAP: a genomic mapping and alignment program for mRNA and EST sequences. *Bioinformatics* 2005;**21**:1859-75.
57. Haas BJ, Delcher AL, Mount SM, et al. Improving the Arabidopsis genome annotation using maximal transcript alignment assemblies. *Nucleic Acids Res* 2003;**31**:5654-66.
58. Holt C, Yandell M. MAKER2: an annotation pipeline and genome-database management tool for second-generation genome projects. *BMC Bioinformatics* 2011;**12**:491.
59. Sun P, Jiao B, Yang Y, et al. WGDI: A user-friendly toolkit for evolutionary analyses of whole-genome duplications and ancestral karyotypes. *Mol Plant* 2022;15:1841-51.
60. Li L, Stoeckert CJ, Jr., Roos DS. OrthoMCL: identification of ortholog groups for eukaryotic genomes. *Genome Res* 2003;**13**:2178-89.
61. Tian HF, Hu QM, Li Z. A high-quality de novo genome assembly of one swamp eel (*Monopterus albus*) strain with PacBio and Hi-C sequencing data. *G3 (Bethesda)* 2021;11.
62. Edgar RC. MUSCLE: multiple sequence alignment with high accuracy and high throughput. *Nucleic Acids Res* 2004;**32**:1792-7.
63. Guindon S, Gascuel O. A simple, fast, and accurate algorithm to estimate large phylogenies by maximum likelihood. *Syst Biol* 2003;**52**:696-704.
64. Stamatakis A. RAxML-VI-HPC: maximum likelihood-based phylogenetic analyses with thousands of taxa and mixed models. *Bioinformatics* 2006;**22**:2688-90.
65. Yang Z. PAML 4: phylogenetic analysis by maximum likelihood. *Mol Biol Evol* 2007;**24**:1586-91.
66. Thorne JL, Kishino H, Painter IS. Estimating the rate of evolution of the rate of molecular evolution. *Mol Biol Evol* 1998;**15**:1647-57.
67. Vogel JP, Garvin DF, Mockler TC, et al. Genome sequencing and analysis of the model grass Brachypodium distachyon. *Nature* 2010;**463**:763-8.
68. Blanc G, Wolfe KH. Widespread paleopolyploidy in model plant species inferred from age distributions of duplicate genes. *Plant Cell* 2004;**16**:1667-78.
69. Sanderson MJ. r8s: inferring absolute rates of molecular evolution and divergence times in the absence of a molecular clock. *Bioinformatics* 2003;**19**:301-2.
70. Han MV, Thomas GW, Lugo-Martinez J, et al. Estimating gene gain and loss rates in the presence of error in genome assembly and annotation using CAFE 3. *Mol Biol Evol* 2013;**30**:1987-97.
71. Chen C, Chen H, Zhang Y, et al. TBtools: An integrative toolkit developed for interactive analyses of big biological data. *Mol Plant* 2020;**13**:1194-202.
72. Hu B, Jin J, Guo AY, et al. GSDS 2.0: an upgraded gene feature visualization server. *Bioinformatics* 2015;**31**:1296-7.

Figure 1: The *P. generosa* genome contig contact matrix using Hi-C data and landscape. (A) Hi-C analysis of *P. generosa* genome contigs. Chromosomes are arranged in the size order from left to right and from top to bottom. The color bar illuminates the logarithm of the contact density from red (10) to white (0) in the plot. (B) The genomic landscape of *P. generosa*: from outer to inner circles: a, the 19 chromosomes; b–d, repetitive element density, gene density, and GC density across the genome, respectively, drawn in 1 Mb non-overlapping windows.

Figure 2: Chromosome synteny of *P. generosa* and *S. constricta*. (A) A dotplot of correspondence of homologous genes in *P. generosa* and *S. constricta*. The red, blue, and gray dots present three similarity levels of homologous gene pairs between *P. generosa* and *S. constricta*. The best match genes are shown in red, while the next best match genes in blue and the least best match genes in gray. (B) A circos plot of synteny analysis between *P. generosa* and *S. constricta*. Line in different colors depicts interchromosomal synteny.

Figure 3: Phylogenetic analysis of *P. generosa* with related species. The estimated species divergence time (million years ago) and the 95% confidential intervals are labeled at each branch site. Divergence times used for time recalibration is illuminated as red dots in the tree.~~The distribution of single-copy orthologs, multiple-copy orthologs, unique paralogs, other orthologs, and unclustered genes in *P. generosa* and related species.~~

Figure 4: The distribution of single-copy orthologs, multiple-copy orthologs, unique

Formatted: Font: Bold

Formatted: Font: Bold

Formatted: Font: Bold, Not Italic

Formatted: Font: Bold

Formatted: Font: Bold

paralogs, other orthologs, and unclustered genes in *P. generosa* and related species.~~Distribution of shared gene families among *P. generosa*, *P. martensi*, *S. broughtonii*, and *P. yessoensis*. Intersections between species indicate the numbers of shared gene families, whereas unique family numbers are shown in species-specific areas. The center represents the number of families shared by all the 4 species.~~

Figure 5: Distribution of shared gene families among *P. generosa*, *P. martensi*, *S. broughtonii*, and *P. yessoensis*. Intersections between species indicate the numbers of shared gene families, whereas unique family numbers are shown in species-specific areas. The center represents the number of families shared by all the 4 species.~~Phylogenetic analysis of *P. generosa* with related species. The estimated species divergence time (million years ago) and the 95% confidential intervals are labeled at each branch site. Divergence times used for time recalibration is illuminated as red dots in the tree.~~

Formatted: Font: Bold

Figure 6~~. –~~: Dynamic evolution and distribution of gene families among *P. generosa* and related species. The numbers of gene gains (+) and losses (−) are shown on the branches, which are also displayed as pie plots: the green part for gene gain, the red part for gene losses and the blue part for gene remained. The divergence times are dated and displayed below the phylogenetic tree. MRCA: most recent common ancestor.

Formatted: Font: Bold

Figure 7~~. –~~: Comparative analysis of gene families based on Pfam annotation in *P. generosa* and related bivalves. A. The 65 most abundant gene families that *P. generosa*

Formatted: Font: Bold

possessing more gene numbers than 8 other bivalves. B. Chromosomal distribution of copine genes ~~predicted~~ in *P. generosa genome*.~~The phylogenetic tree of genes annotated to PF07002 domains in *P. generosa* and *S. constricta*, shown in the background colors of red and pink, respectively.~~ C. The phylogenetic tree of copine genes ~~annotated to PF07002 domains~~ in *P. generosa* and *S. constricta*, shown in the background colors of red and pink, respectively.~~Chromosomal distribution of copine genes predicted in *P. generosa genome*.~~ D. Domain structures of copine genes predicted in *P. generosa* genome. E. The 3D structures of ~~*Pg02g00048*~~Pg02g00048 gene in *P. generosa* and ~~*XP_0533888641*~~XP_0533888641 gene in *Mercenaria mercenaria*.

854

Table 1: Statistics of the DNA sequence data used for *P. generosa* genome assembly

Source	Platform	Library size	Clean data (Gb)	Read length (bp)	Sequencing coverage (x)
**Genome-short reads**	BGISEQ-500	300 bp	258.19	150	181
**Genome-long reads**	PacBio sequel	20 Kb	164.46	26,513*	115
**Hi-C**	BGISEQ-500	300 bp	233.49	150	163

[a]"26,513*" indicated the N50 of subreads.

Table 2: Statistics of the genome assembly of *P. generosa*

Statistics	Contig	Scaffold	Chromosome
**Total Number (#)**	2,086	39	19
**Total length (bp)**	1,473,137,789	1,474,161,289	1,432,060,667
**Average Length (bp)**	706,202	37,799,007	75,371,614
**N50 Length (bp)**	1,571,249	73,788,920	76,670,739
**N90 Length (bp)**	418,579	53,843,121	53,843,121
**Maximum Length (bp)**	6,469,558	101,196,518	101,196,518
**Minimum Length (bp)**	17	29,000	1,432,060,667
**GC content**	34.33%	34.33%	34.33%
**Anchored rate (%)**	94.70%		

Table 3: Repetitive element annotations in *P. generosa*

Repetitive sequence			Transposable elements		
Type	Length (bp)	% in genome	Type	Length (bp)	% in genome
DNA	318426608	21.601	DNA	318426608	21.601
LINE	134171392	9.102	LINE	134171392	9.102
SINE	27249093	1.848	SINE	27249093	1.848
LTR	55380608	3.756	LTR	55380608	3.756
Other	31141	0.002	Other	31141	0.002
Unknown	151891763	10.304	Unknown	151891763	10.304
Tandem repeat	347641207	23.582	Total	583000390	39.548
Total	854873570	57.991			

	Type	Length (bp)	% in genome
Transposable elements	DNA	318426608	21.601
	LINE	134171392	9.102
	SINE	27249093	1.848
	LTR	55380608	3.756
	Other	31141	0.002
	Unknown	151891763	10.304
	Total	583000390	39.548
Tandem repeat		347641207	23.582
Total		854873570	57.991

Table 4: BUSCO results for analysis of genome completeness for *P. generosa*

Type	Genome assembly		Gene set	
	Number of genes	Percentage (%)	Number of genes	Percentage (%)
**Complete BUSCOs (C)**	887	93.0	844	88.4
**Complete and single-copy BUSCOs (S)**	842	88.3	795	83.3
**Complete and duplicated BUSCOs (D)**	45	4.7	49	5.1
**Fragmented BUSCOs (F)**	34	3.6	49	5.1
**Missing BUSCOs (M)**	33	3.4	61	6.5
**Total BUSCO groups**	954	100	954	100

Table 5: The PCGs of *P. generosa* and 11 other species for evolutionary analysis

Species	Total genes	Unclustered genes	Families	Unique families	Ave. genes per family
P. generosa	35,034	6,168	12,034	1,749	2.4
A. purpuratus	26,256	3,720	13,196	290	1.71
B. platifrons	33,584	3,197	12,409	1,775	2.45
C. gigas	28,402	3,638	11,818	828	2.1
D. rerio	25,444	1,791	9,210	295	2.57
H. sapiens	20,229	1,488	9,251	226	2.03
P. yessoensis	24,521	1,704	13,017	137	1.75
P. maximus	26,152	1,518	13,276	164	1.86
P. martensi	25,526	2,403	11,043	318	2.09
S. broughtonii	24,045	2,770	11,314	538	1.88
X. tropicalis	19,967	1,016	9,226	159	2.05
C. elegans	33,552	5,600	8,201	3,720	3.41

 Click here to access/download;Figure;Figure6.pdf

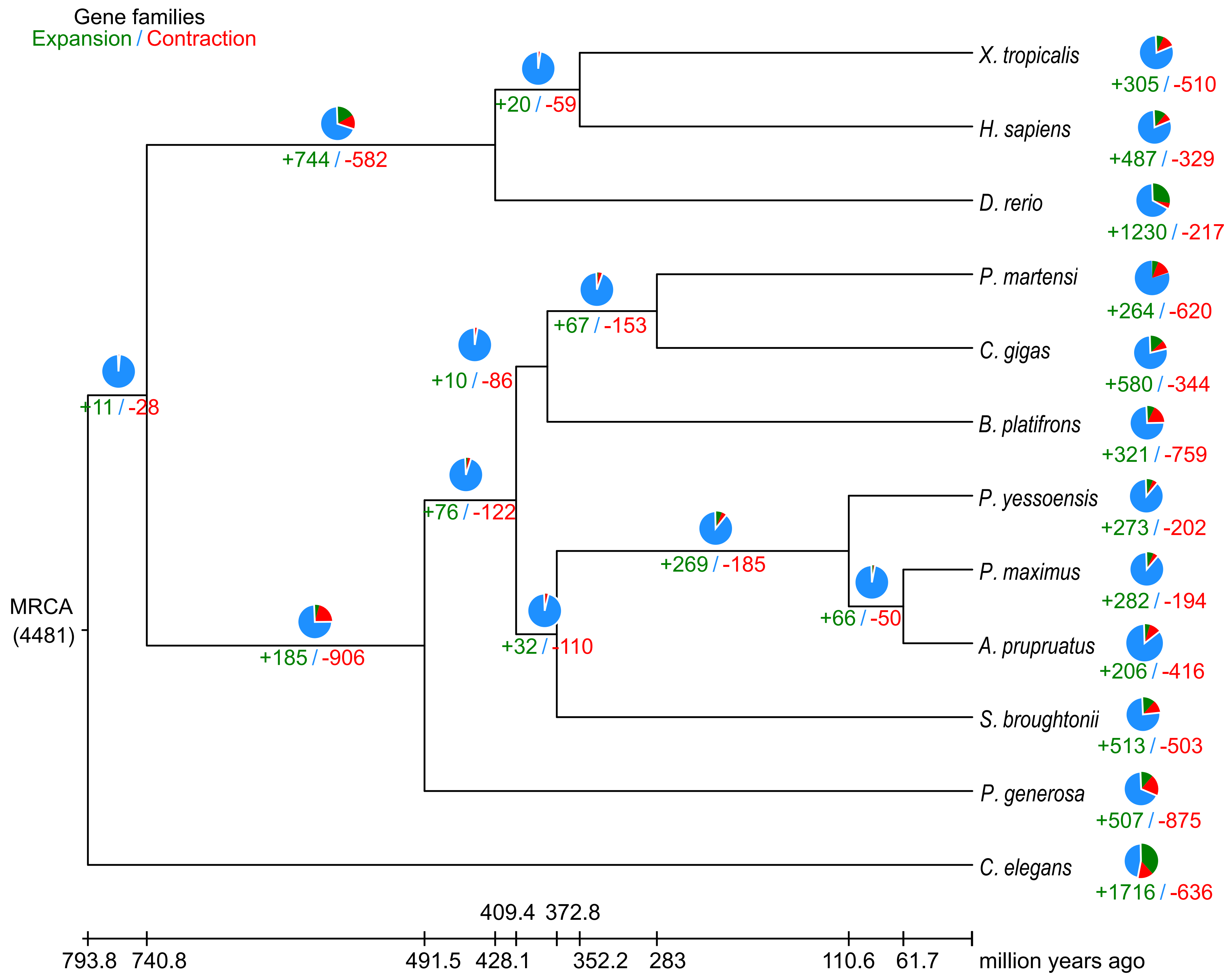

Figure1

Click here to access/download;Figure;Figure1.pdf

A B

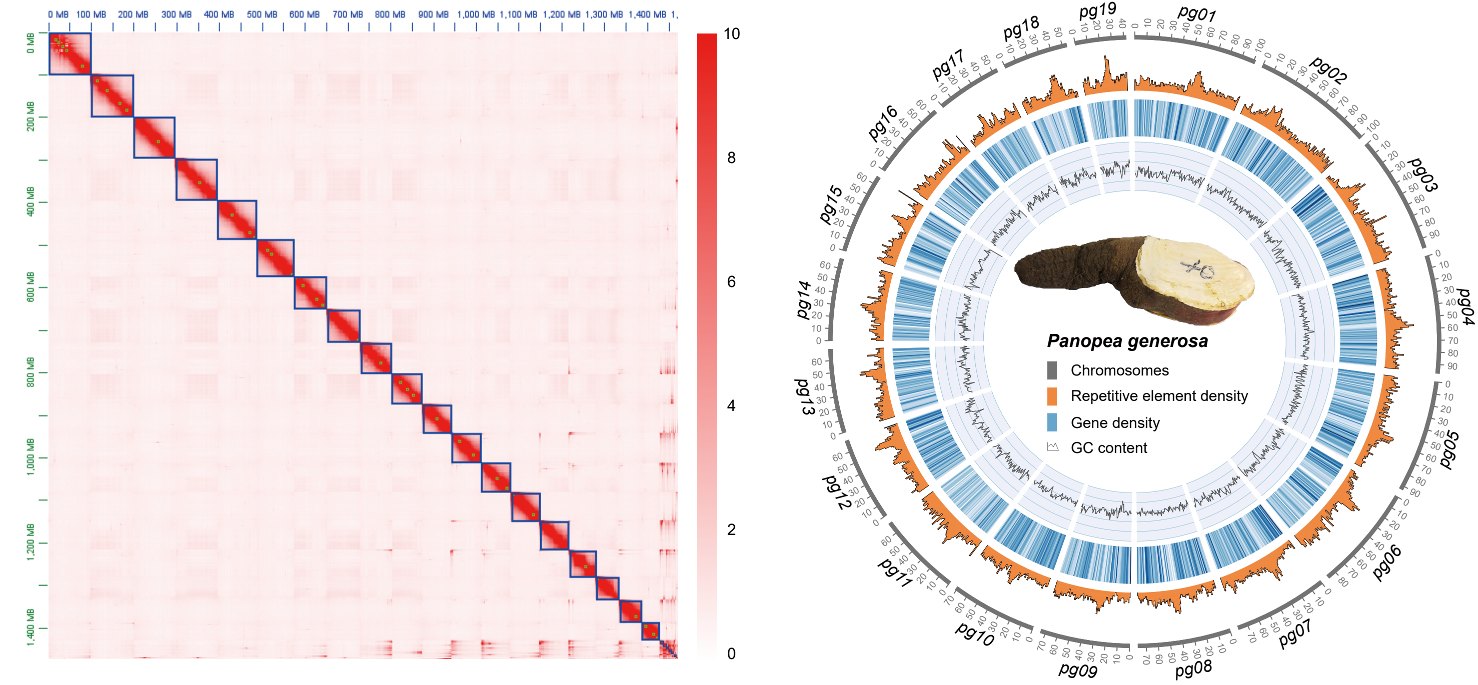

Figure2

Click here to access/download;Figure;Figure2.pdf

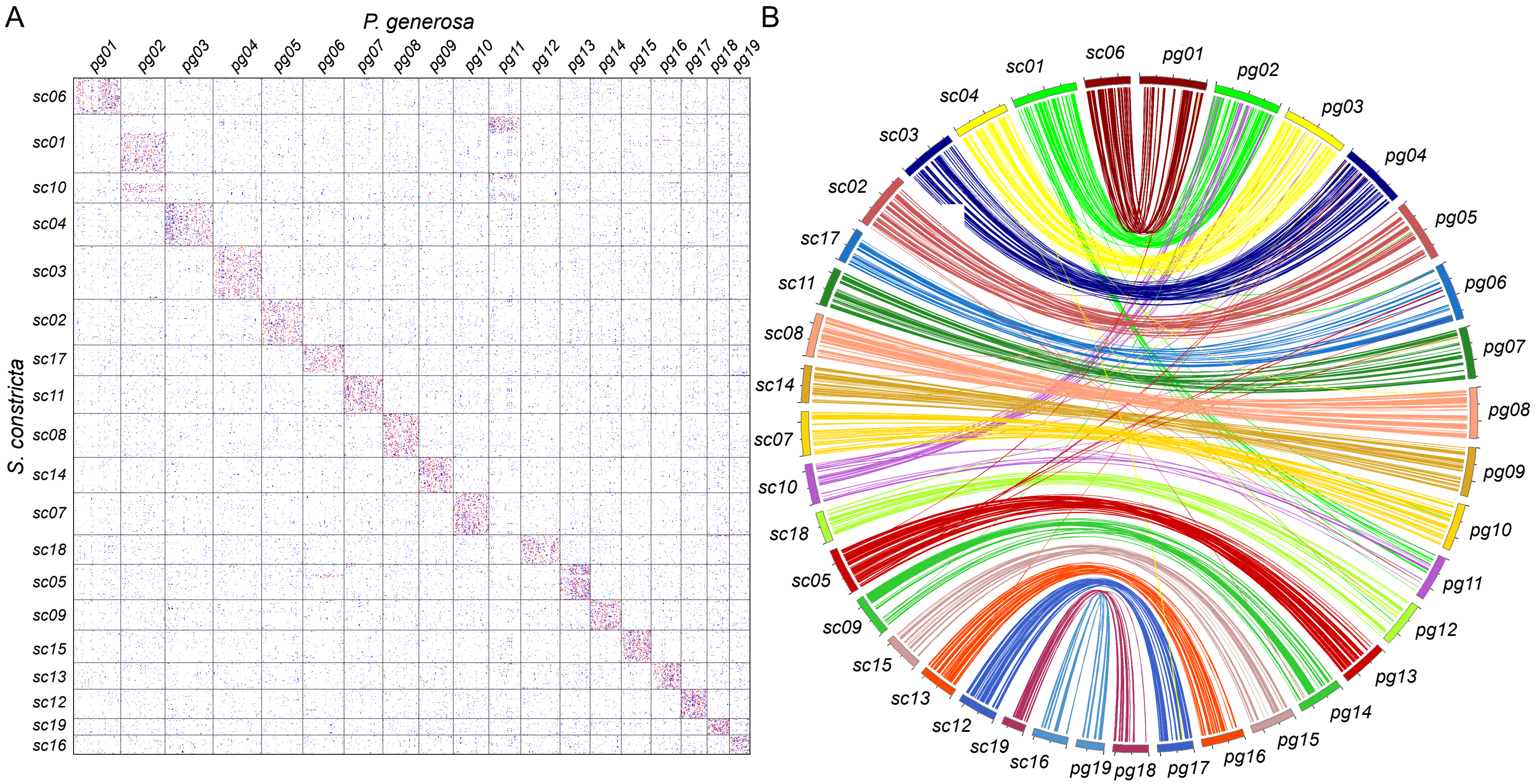

Figure3

Click here to access/download;Figure;Figure3.pdf

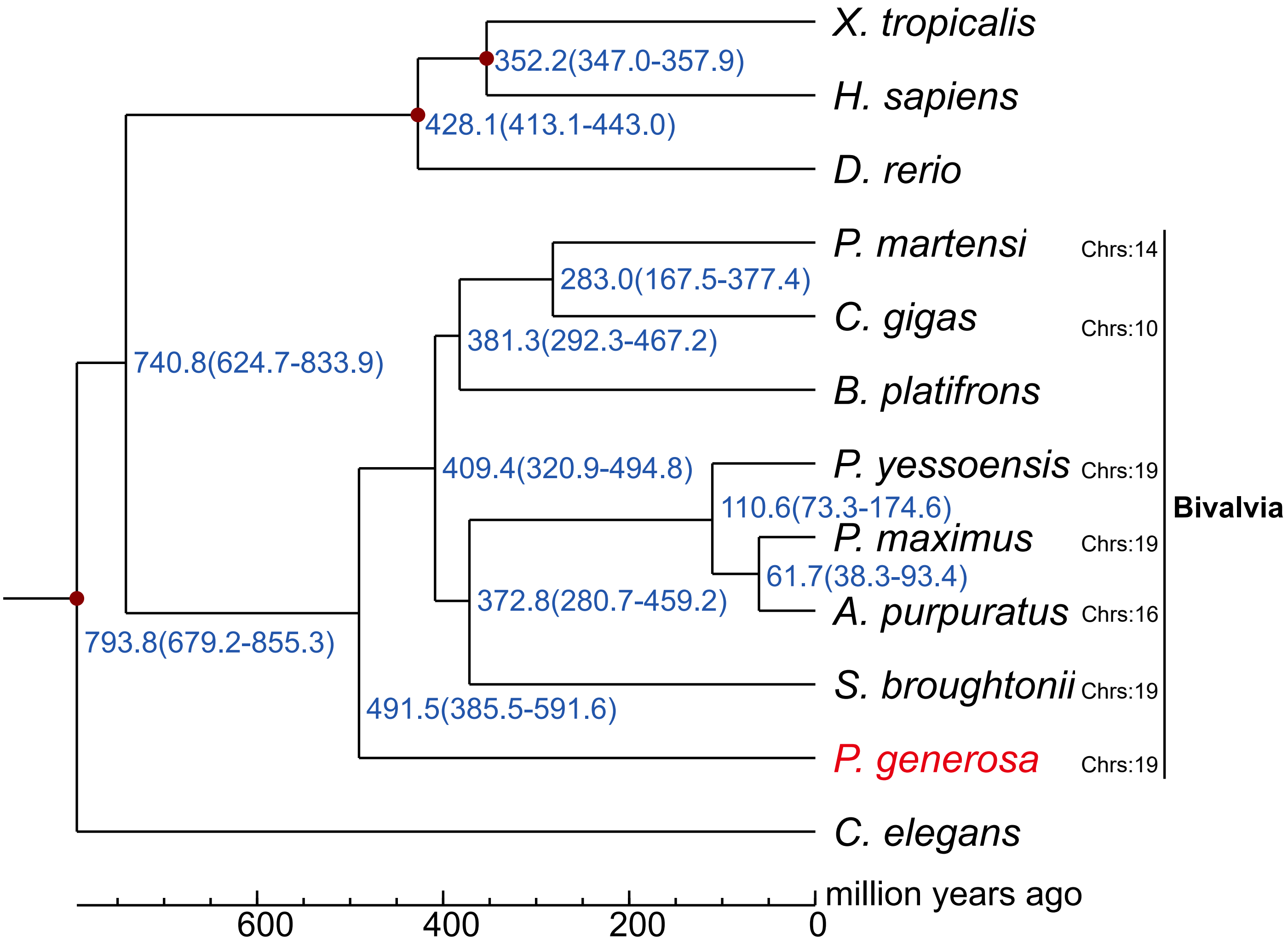

Figure4

Click here to access/download;Figure;Figure4.pdf

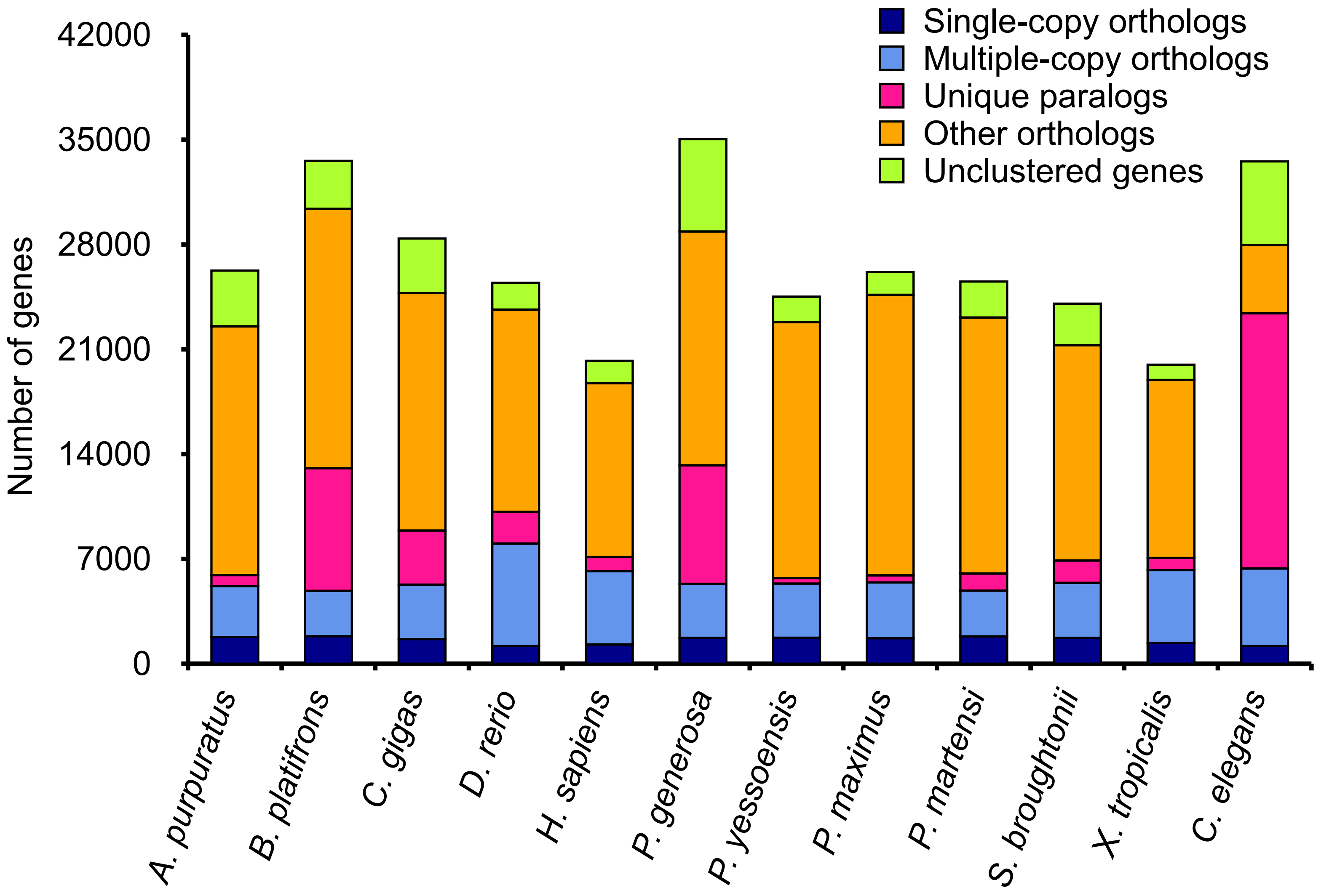

Figure5

Click here to access/download;Figure;Figure5.pdf

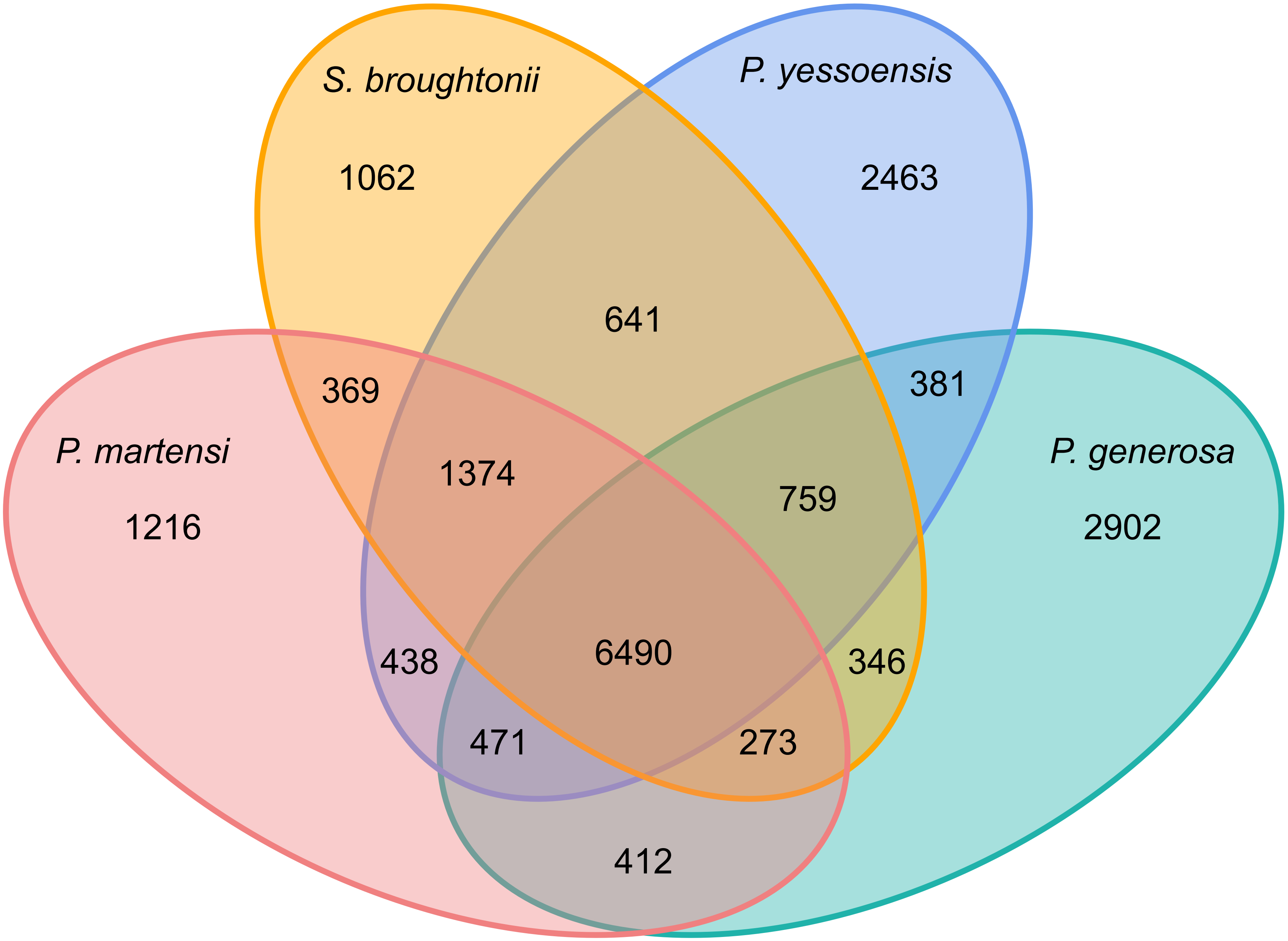

Number of gene families

Figure7

Click here to access/download;Figure;Figure7.pdf

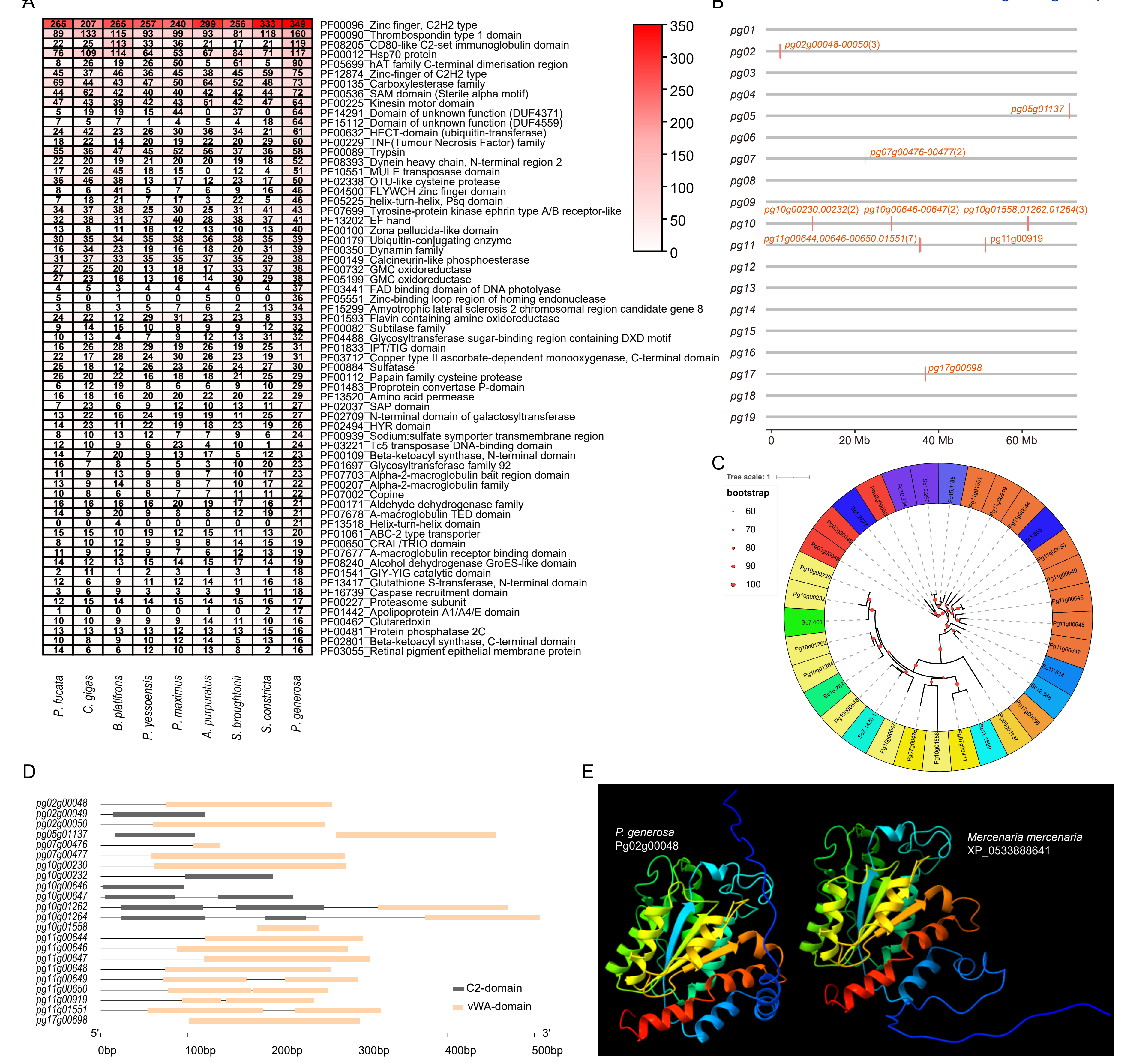

Supplementary figures

Click here to access/download
Supplementary Material
Supplementary figures.docx

Supplementary tables

Click here to access/download
Supplementary Material
Supplementary tables.xlsx
