# Supplementary material for: Chromosome-level genome assembly of the Pacific geoduck Panopea generosa reveals major inter- and intrachromosomal rearrangements and substantial expansion of the copine gene family
Source: Gigascience. 2023 Dec 19;12:giad105. doi: 10.1093/gigascience/giad105 (PMC10729735; doi:10.1093/gigascience/giad105)
Supplement: giad105_GIGA-D-22-00284_Revision_3 [file giad105_giga-d-22-00284_revision_3.pdf]

GigaScience

Chromosome-level genome assembly of the Pacific geoduck Panopea generosa reveals major inter- and intra-chromosomal rearrangements and substantial expansion of the copine gene family

--Manuscript Draft--

**Manuscript Number:**	GIGA-D-22-00284R3
**Full Title:**	Chromosome-level genome assembly of the Pacific geoduck Panopea generosa reveals major inter- and intra-chromosomal rearrangements and substantial expansion of the copine gene family
**Article Type:**	Data Note
**Funding Information:**	Taishan Scholar Foundation of Shandong Province — Professor Nansheng Chen<br>Strategic Priority Research Program of Chinese Academy of Sciences (XDB42000000) — Professor Nansheng Chen<br>Chinese Academy of Sciences Pioneer Hundred Talents Program — Professor Nansheng Chen<br>Earmarked Workstation Fund for QRJH — Professor Chunde Wang, Professor Nansheng Chen
**Abstract:**	The Pacific geoduck Panopea generosa (class Bivalvia, order Adapedonta, family Hiatellidae, genus Panopea) is the largest known burrowing bivalve with considerable commercial value. Pacific geoduck and other geoduck clams play important roles in maintaining ecosystem health for their filter feeding habit and coupling pelagic and benthic processes. Here, we report a high-quality chromosome-level genome assembly of P. generosa to characterize its phylogeny and molecular mechanisms of its life strategies. The assembled P. generosa genome consists of 19 chromosomes with a size of 1.47 Gb, a contig N50 length of 1.6 Mb, and a scaffold N50 length of 73.8 Mb. The BUSCO test of the genome assembly showed 93.0% completeness. Constructed chromosome synteny revealed many occurrences of inter- and intra-chromosomal rearrangements between P. generosa and Sinonovacula constricta. Of the 35,034 predicted protein coding genes (PCGs), 30,700 (87.6%) could be functionally annotated in public databases, indicating the high quality of genome annotation. Comparison of gene copy numbers of gene families among P. generosa and 11 selected species identified 507 rapidly expanded P. generosa gene families that are functionally enriched in immune and gonad development and may be involved in its complex survival strategies. In particular, genes carrying the copine domains underwent additional duplications in P. generosa, which might be important for neuronal development and immune response. The availability of a fully annotated chromosome-level genome provides a valuable data set for genetic breeding of P. generosa.
**Corresponding Author:**	Nansheng Chen<br>Institute of Oceanology Chinese Academy of Sciences<br>Qingdao, Shandong CHINA
**Corresponding Author Secondary Information:**	
**Corresponding Author's Institution:**	Institute of Oceanology Chinese Academy of Sciences
**Corresponding Author's Secondary Institution:**	
**First Author:**	Jing Wang
**First Author Secondary Information:**	
**Order of Authors:**	Jing Wang
	Qing Xu
	Min Chen

Powered by Editorial Manager® and ProduXion Manager® from Aries Systems Corporation

	Yang Chen
	Chunde Wang
	Nansheng Chen
**Order of Authors Secondary Information:**	
**Response to Reviewers:**	Manuscript number: GIGA-D-22-00284R2<br>Title: “Chromosome-level genome assembly of the Pacific geoduck Panopea generosa reveals major inter- and intra-chromosomal rearrangements and substantial expansion of the copine gene family”<br><br>Responses to reviewers:<br>Reviewer #1: The authors have done a good job at address my comment on the previous version of their manuscript. While I am generally happy with the state of the manuscript, I still have some minor grammatical issues that can be easily addressed by the authors. Additionally, I agree with Reviewer 2 that the Discussion section is very repetitive of the Results section and adds very little new insight to the manuscript. I would suggest, if possible, that the Discussion be rephrased to include a discussion of the significance of the results, or that it be reduced to reduce repetition.<br>Response: Thanks for the comments and suggestions.<br><br>L72-73: "These genomes can provide a resource for comparative genomic for gaining evolutionary and other insights of bivalves and even molluscs" TO "These genomes can provide a resource for comparative genomics, and for gaining evolutionary and other insights into bivalves and molluscs"<br>Response: Corrected.<br><br>L95: "gene family expansion and contraction" TO "gene family expansion and contraction analysis"<br>Response: Corrected.<br><br>L128-131: "Of these PCGs, 30,700 (87.6%) were annotated with according to the public database including Nr, Swissprot, KEGG, KOG, TrEMBL, Interpro, and GO (Supplementary Table S5), suggesting the high quality of genome annotation." TO "Of these PCGs, 30,700 (87.6%) had functional annotations assigned using comparisons to public databases, including NCBIs nr, Swiss-Prot, KEGG, KOG, TrEMBL, InterPro, and GO (Supplementary Table S5), supporting the high quality of the genome."<br>Response: Corrected.<br><br>L133: "elements varied from" TO "elements varying from"<br>Response: Corrected.<br><br>L137: "93.0% of core PCGs were" TO "93.0% of the core genes used by BUSCO were"<br>Response: Corrected.<br><br>L138: ", higher than that" TO ", which is higher than that"<br>Response: Corrected.<br><br>L139: "of annotated PCGs using BUSCO, which were aligned to 88.4% of the full-length core orthologs, suggesting that quality of the annotation can be further improved in the future." TO "of the predicted PCGs using BUSCO, which identified 88.4% of the core genes as full-length, suggesting that quality of the predicted genes can be further improved in the future."<br>Response: Corrected.<br><br>L150: "chromosomes of these two species were both 19" TO "chromosomes in these two species is still 19"<br>Response: Corrected.<br><br>L154-155: "two species P. generosa and S. constricta," TO "two species (P. generosa and S. constricta),"<br>Response: Corrected.

Powered by Editorial Manager® and ProduXion Manager® from Aries Systems Corporation

L159: "orthologous genes of the 12" TO "orthologous genes from 12"
Response: Corrected.

L219-221: "while some has both C2-domain and the vWA-domain in P. generosa (Figure 7D) like copine genes identified in other species [33]." TO "however some have both a C2-domain and a vWA-domain in P. generosa (Figure 7D), like copine genes identified in other species [33]."
Response: Corrected.

L222: "due to errors of genome assembly or gene prediction" TO "due to genome assembly or gene prediction errors"
Response: Corrected.

L230: "important bivalves the Pacific geoduck P. generosa," TO "important bivalve, the Pacific geoduck P. generosa,"
Response: Corrected.

L235: "instance of chromosome-level genomes of the bivalve species" TO "instance of a chromosome-level genome assembly from a bivalve species"
Response: Corrected.

L259: "that the numbers of PCGs of many gene families" TO "that the number of PCGs in many gene families"
Response: Corrected.

L260: "substantially in evolution" TO "substantially during the evolution of this group"
Response: Corrected.

L262: "genes were identified" TO "genes identified"
Response: Corrected.

L265-267: "composition of functional domains, which include C2 domain and vWA-domain (Figure 7D), and the similarity of three-dimensional structure to that of known copine genes (Figure 7E) suggests functional conservation" TO "composition of functional domains, which includes the C2 and vWA domains (Figure 7D), and the similarity of three-dimensional structure to that of known copine genes (Figure 7E), suggests conservation"
Response: Corrected.

L268: "completion of P. generosa facilitates genome comparative analysis" TO "completion of the genome P. generosa facilitates comparative analysis"
Response: Corrected.

L271: "construction of P. generosa chromosome-level genome" TO "construction of a chromosome-level genome assembly of P. generosa"
Response: Corrected.

L272: "in this organism, but also" TO "in this organism, but will also"
Response: Corrected.

I agree with Reviewer 2 that the discussion is very repetitive of the Results section and does not add much insight beyond what has already been said. If possible, I would advise the Authors to revise this section so that it provides a higher-level overview of the significance of their results.
Response: We have reduced repetitive statements in the discussion section.

L281: "samples- were transferred to laboratory" TO "samples were transferred to the laboratory"
Response: Corrected.

L365: "PCGs in P. generosa" TO "PCGs in the P. generosa"
Response: Corrected.

L424: "family-wide P-Values were" TO "family-wide p-values were"

Powered by Editorial Manager® and ProduXion Manager® from Aries Systems Corporation

	Response: Corrected.
**Additional Information:**	
**Question**	**Response**
Are you submitting this manuscript to a special series or article collection?	No
**Experimental design and statistics**<br><br>Full details of the experimental design and statistical methods used should be given in the Methods section, as detailed in our Minimum Standards Reporting Checklist. Information essential to interpreting the data presented should be made available in the figure legends.<br><br>Have you included all the information requested in your manuscript?	Yes
**Resources**<br><br>A description of all resources used, including antibodies, cell lines, animals and software tools, with enough information to allow them to be uniquely identified, should be included in the Methods section. Authors are strongly encouraged to cite Research Resource Identifiers (RRIDs) for antibodies, model organisms and tools, where possible.<br><br>Have you included the information requested as detailed in our Minimum Standards Reporting Checklist?	Yes
**Availability of data and materials**<br><br>All datasets and code on which the conclusions of the paper rely must be either included in your submission or deposited in publicly available repositories (where available and ethically appropriate), referencing such data using a unique identifier in the references and in the "Availability of Data and Materials" section of your manuscript.	Yes

Have you have met the above requirement as detailed in our Minimum Standards Reporting Checklist?	

Powered by Editorial Manager® and ProduXion Manager® from Aries Systems Corporation

Manuscript

Click here to access/download;Manuscript;Manuscript-Nov22.docx

Chromosome-level genome assembly of the Pacific geoduck *Panopea generosa* reveals major inter- and intra-chromosomal rearrangements and substantial expansion of the copine gene family

Jing Wang[1,2,3], Qing Xu[1,2,3], Min Chen[4], Yang Chen[1,2,3], Chunde Wang[4,5*], Nansheng Chen[1,2,3,6*]

[1]CAS Key Laboratory of Marine Ecology and Environmental Sciences, Institute of Oceanology, Chinese Academy of Sciences, Qingdao, China

[2]Laboratory of Marine Ecology and Environmental Science, Qingdao National Laboratory for Marine Science and Technology, Qingdao, China

[3]Center for Ocean Mega-Science, Chinese Academy of Sciences, Qingdao, China

[4]Yantai Institute of Coastal Zone Research and Center for Ocean Mega-Science, Chinese Academy of Sciences, Yantai, China

[5]Marine Science and Engineering College, Qingdao Agricultural University, Qingdao, China

[6]Department of Molecular Biology and Biochemistry, Simon Fraser University, Burnaby, BC, Canada

Jing Wang Email: wangjing2019@qdio.ac.cn; Qing Xu Email: xuqing_77@163.com; Min Chen Email: mchen@yic.ac.cn; Yang Chen Email: cy4043@hevttc.edu.cn.

*Correspondence address. Nansheng Chen, CAS Key Laboratory of Marine Ecology and Environmental Sciences, Institute of Oceanology, Chinese Academy of Sciences, Qingdao, China. Email: chenn@qdio.ac.cn; Chunde Wang, Yantai Institute of Coastal Zone Research and Center for Ocean Mega-Science, Chinese Academy of Sciences, Yantai,

China. E-mail: chundewang2007@163.com.

Jing Wang [0000-0003-2267-4287];

Qing Xu [0000-0003-0395-0557];

Min Chen [0000-0002-9146-2504];

Yang Chen [0000-0002-9146-2504];

Chunde Wang [0000-0002-6931-7394];

Nansheng Chen [0000-0002-0615-7215]

Abstract

The Pacific geoduck *Panopea generosa* (class Bivalvia, order Adapedonta, family Hiatellidae, genus *Panopea*) is the largest known burrowing bivalve with considerable commercial value. Pacific geoduck and other geoduck clams play important roles in maintaining ecosystem health for their filter feeding habit and coupling pelagic and benthic processes. Here, we report a high-quality chromosome-level genome assembly of *P. generosa* to characterize its phylogeny and molecular mechanisms of its life strategies. The assembled *P. generosa* genome consists of 19 chromosomes with a size of 1.47 Gb, a contig N50 length of 1.6 Mb, and a scaffold N50 length of 73.8 Mb. The BUSCO test of the genome assembly showed 93.0% completeness. Constructed chromosome synteny revealed many occurrences of inter- and intra-chromosomal rearrangements between *P. generosa* and *Sinonovacula constricta*. Of the 35,034 predicted protein-coding genes (PCGs), 30,700 (87.6%) could be functionally annotated in public databases, indicating the high quality of genome annotation. Comparison of gene copy numbers of gene families among *P. generosa* and 11 selected species identified 507 rapidly expanded *P. generosa* gene families that are functionally enriched in immune and gonad development and may be involved in its complex survival strategies. In particular, genes carrying the copine domains underwent additional duplications in *P. generosa*, which might be important for neuronal development and immune response. The availability of a fully annotated chromosome-level genome provides a valuable data set for genetic breeding of *P. generosa*.

Keywords: *Panopea generosa*, chromosome-level genome assembly, genetic breeding,

evolutionary adaptation

Introduction

The Pacific geoduck *Panopea generosa* (NCBI:txid1049056; marinespecies.org:taxname:545994) is one member of genus *Panopea* which includes the world's largest burrowing bivalves. *P. generosa* is usually found in low intertidal and subtidal sediments throughout the northeast Pacific coast, including the United States (Alaska, Washington, and California), Canada (British Columbia), and Mexico (north Baja Pacific Coast) [1, 2]. Geoducks can reach more than 25 cm in shell length, and more than 100 cm in siphon length [3]. Geoduck adults are usually buried in muddy-sandy sediment at depths ranging 60–100 cm, with only their siphon tips exposed to respire, capture food, and release secretion/excretion products and gametes. The sedentary behavior may contribute to their long life spans which can be as long as 168 years for *P. generosa* [4]. Due to these unique life strategies, it is expected that geoduck should have distinctive growth and development mechanisms, especially in relation to benthic life and immune system.

Geoduck clams play important roles in maintaining ecosystem health for their filter feeding habit and coupling pelagic and benthic processes by ejecting undigested mucus-bound feces and pseudo feces to the sediment surface. They are prey for sea otters, fishes, crabs, and sea stars [5, 6]. As marine calcifiers, shell concentrations of *Panopea* inside Scalichnus burrows have been analyzed to reconstruct the sequence of events related to storm events [7]. Geoduck clams possess great commercial fishery value in Canada and the USA [8]. Since the recruitment of geoducks have been low due to overfishing and their vulnerability to environmental changes [9, 10], there has been an increasing interest in

genetic breeding of geoducks.

Bivalves are an ancient lineage of bilaterian, and are a diverse Class of Mollusca. To date, the chromosome-level genomes of only about 40 bivalve species have been assembled [11]. These genomes can provide a resource for comparative genomics, and for gaining evolutionary and other insights into bivalves and molluscs. These genomes show a remarkable level of diversity. For example, the assembled genome sizes of bivalves vary widely, ranging from 543.9 Mb in *Lutraria thynchaena* [12] to 2.6 Gb in *Modiolus philippinarum* [13] (Supplementary Table S1). Among bivalves, the genome sizes of most superorder Imparidentia species ranged from 1 Gb to 1.8 Gb, and that of the species in the order Adapedonta, which includes *P. generosa*, ranged from 1 Gb to 1.5 Gb [14].

The numbers of chromosomes also vary substantially among bivalves, suggesting active genome recombination during the evolution of bivalves [15]. While some species of the order Ostreida, including *Crassostrea gigas* [16], *Crassostrea virginica*, *Crassostrea hongkongensis* [17], *Crassostrea ariakensis* [18], *Crassostrea angulate*, and *Ostrea edulis*, have 10 chromosomes, the species of the order Pterioidea and Mytilida have 14–15 chromosomes, such as *Pinctada fucata* [19], *Mytilus coruscus* [20] and *Limnoperna fortunei* (Supplementary Table S1). The chromosome numbers of the order Myida varied from 16–17. Interestingly, the chromosome number of species in most other orders is 19, including Venerida, Cardiida, Unionida, Arcida, and Pectinida . The chromosome number of all reported species in the order Adapedonta, which includes *P. generosa*, is also 19.

Nevertheless, a high-quality chromosome-level reference genome of *P. generosa* is currently not available, hindering the development of geoduck genetic breeding programs.

In this study, we reported the first chromosome-scale genome assembly for *P. generosa* generated using cutting-edge technologies including next-generation sequencing, long read sequencing, and high-throughput chromosome conformation capture (Hi-C) technologies. We further performed gene family clustering, phylogenetic analysis, and gene family expansion and contraction analysis, in order to understand its adaptation, growth, development and immunity. The availability of this genome information will facilitate research in molecular evolution and genetic breeding.

Results

Genome sequencing and assembly

For the genome assembly of *P. generosa*, short reads were obtained for estimating the genome size, heterozygosity rate, and repeat content, long reads were obtained for initial genome assembly, and Hi-C reads were obtained for the construction of chromosomes (Table 1).

Genome size, heterozygosity rate, and repeat content of *P. generosa* estimated by the *k*-mer analysis [21] of the short reads were 1.47 Gb, 1.37% and 68.08% respectively (Supplementary Figure S1 and Table S2). The assembled genome of *P. generosa*, which was generated using PacBio long reads (N50 length = 26,513 bp) with Falcon and Hi-C data, consisted of 19 pseudomolecules (Figure 1; Supplementary Table S3), with a contig anchoring rate of 94.70%. The assembled *P. generosa* genome has a total length of 1.47 Gb (1,474,161,289 bp) with a contig N50 length of 1.6 Mb and a scaffold N50 length of 73.79 Mb (Table 2).

The heterozygosity rate of *P. generosa* (1.37%) fell within the range of the

heterozygosity rates of bivalves whose genomes have been sequenced and assembled, which vary broadly from 0.11% in *Margaritifera margaritifera* [22] to 3.20% in *Crassostrea gigas* [23], The heterozygosity rate of *P. generosa* (1.37%) was close to that of another burrowing bivalve *Sinonovacula constricta* (1.55%) and a deep-sea mussel *Bathymodiolus platifrons* (1.03–1.24%) [13, 24] (Supplementary Table S4).

Genome annotation and evaluation

The majority (57.99%) of the *P. generosa* genome was estimated to be repetitive elements (Table 3), which is similar to but lower than the predicted content of repetitive elements using *k*-mer analysis (68.08%). The top three most frequent categories of repetitive elements in the *P. generosa* genome were DNA transposons (21.6%), long interspersed nuclear elements (LINEs, 9.1%) and long terminal repeats (LTRs, 3.76%) (Table 3).

A total number of 35,034 protein-coding genes (PCGs) were annotated in the *P. generosa* genome. The average gene length, average CDS length, average number of exons per gene, average exon length, and average intron length were 13,469 bp, 1273 bp, 6, 220 bp, and 2171 bp, respectively. Of these PCGs, 30,700 (87.6%) had functional annotations assigned using comparisons to public databases, including NCBIs nr, Swiss-Prot, KEGG, KOG, TrEMBL, InterPro, and GO (Supplementary Table S5), supporting the high quality of the genome.

The distribution of repetitive elements was highly uneven, with regional content of the repetitive elements varying from 34.76% to 84.89% in the *P. generosa* genome, with peaks occurring at regions with low gene density (Figure 1B).

To evaluate the completeness of the assembly, the *P. generosa* genome was tested using BUSCO [25] with the metazoa_odb10 database (954 core genes). We found that 93.0% of the core genes used by BUSCO were identified as full-length in the *P. generosa* genome (Table 4), which is higher than that of *S. constricta* (91.5%) [14] (Supplementary Table S6). We further tested the completeness of the predicted PCGs using BUSCO, which identified 88.4% of the core genes as full-length, suggesting that quality of the predicted genes can be further improved in the future.

Chromosomal synteny analysis between *P. generosa* and *S. constricta*

Comparative analysis of genome-wide gene collinearity revealed a high chromosome synteny between *P. generosa* and *S. constricta*. Of the 19 *P. generosa* chromosomes, 17 chromosomes were found to have one-to-one correspondences with 17 *S. constricta* chromosomes (Figure 2). However, several large-scale inter-chromosomal rearrangements were also identified, such as the arrangement among *P. generosa* chromosomes *pg02* and *pg11* and *S. constricta* chromosomes *sc01* and *sc10* (Figure 2). Despite these large-scale inter-chromosomal rearrangements, the number of chromosomes in these two species is still 19.

In addition to these major inter-chromosomal rearrangement events, comparative analysis of these two genomes also revealed extensive intra-chromosomal rearrangements, which resulted in low gene synteny within these chromosomes. Instead of a clear diagonal linear relationship between genes of these two species (*P. generosa* and *S. constricta*), a near random scattering of the relationships were observed (Figure 2A). These intra-chromosomal rearrangement events were also clearly shown in Figure

2B, such as *P. generosa* chromosome *pg01* vs *S. constricta* chromosome *sc06*.

Evolutionary analysis of *P. generosa* and other bivalves

Phylogenetic analysis using 326 one-to-one single-copy orthologous genes from 12 species showed that *P. generosa* was tightly clustered with other bivalves as expected (Figure 3). According to the phylogenetic tree, the divergence time of *P. generosa* from its nearest node, which represents the common ancestor of many other bivalves, was approximately 491.5 Mya (Figure 3). This divergence time between *P. generosa* and other bivalves was similar to the divergence time between *S. constricta* and other bivalves [14]. Interestingly, the numbers of chromosomes of bivalves vary substantially, and closely related bivalves can have different numbers of chromosomes (Figure 3). The oysters *C. gigas* with 10 chromosomes and *P. martensi* with 14 chromosomes diverged from the clam *S. broughtoni* and scallops with 19 chromosomes 409.4 MYA. What's more, the scallop *A. purpuratus* with 16 chromosomes diverged from another scallop *P. maximus* with 19 chromosomes 61.7 MYA.

Comparative analysis of gene families

In total, 30,616 gene families were identified among *P. generosa* and 11 other species (*Pinctada martensi*, *C. gigas*, *B. platifrons*, *Patinopecten yessoensis*, *Pecten maximus*, *Argopecten purpuratus*, *Scapharca broughtonii*, *Homo sapiens*, *Xenopus tropicaalis, Danio rerio, and Caenorhabditis elegans*) (Table 5, Figure 4, Supplementary Table S7). As compared with the other 11 species, 7917 genes belonging to 1749 gene families were found to be *P. generosa*-specific, which fell into the range of 1567–15051 species-specific gene families identified in 12 bivalves [26]. Comparative analysis of the PCGs of *P.*

martensi, *S. broughtonii*, *P. yessoensis*, and *P. generosa* revealed 6490 common gene families shared by these species and 2902 gene families specific to *P. generosa* (Figure 5).

A total of 507 rapidly expanded gene families (involving 2,734 genes) and 875 rapidly contracted gene families (involving 792 genes) were identified in the *P. generosa* genome compared to the most recent common ancestor of both *P. generosa* and other 11 species (Figure 6). The annotation with the KEGG pathway database [27] revealed that the genes of expanded families were distributed in 123 pathways, which were mainly enriched in organismal systems, genes associated with diseases, environmental information processing (e.g. phototransduction), and phosphatidylinositol signaling system, suggesting their important contribution to the adaptation of benthic bivalves. According to the enriched KEGG pathways of expanded gene families in *P. generosa* (Supplementary Table S8), there were a few significant enriched pathways (Q value < 0.05) related to gonad development. For example, adrenergic signaling in cardiomyocytes, and glycine, serine and threonine metabolism which have been shown to function as part of spermatogenesis of the fluted giant clam *Tridacna squamosa* [28]. Moreover, oocyte meiosis, apoptosis, Ras signaling pathway, calcium signaling pathway, steroid hormone biosynthesis, GnRH signaling pathway, insulin signaling pathway, oxytocin signaling pathway, and ovarian steroidogenesis were documented to be enriched in *Procambarus clarkii* ovary development [29]. Geoducks have become a focus of significant aquaculture research and development with a considerable commercial value [30, 31]. The enriched gonad development-related pathways and genes could provide basic data for the further genetic

breeding research of *P. generosa* and its closely related species.

We further compared gene families in different bivalves by searching for functional domains contained in PCGs in *P. generosa* and eight other bivalves using InterProScan [32]. Examination of the top 65 most frequent domains that had specific expansions in *P. generosa* (Figure 7A) showed that the gene numbers of many important gene families were substantially expanded in *P. generosa*, including these containing the GIY-YIG catalytic domain (PF01541), the caspase recruitment domain (PF16739), the ApoA/ApoE domain (PF01442), and the copine domain (PF07002). In particular, the copy number of the copine gene family, which has been implicated in a range of cell signaling [33], was 22 in *P. generosa*, twice as many as those identified in *S. constricta*. Examination of positions of the *P. generosa* copine genes revealed that they were distributed in chromosomes *pg02*, *pg05*, *pg07*, *pg10*, *pg11* and *pg17* (Figure 7B). Interestingly, many genes formed local clusters (e.g. eight copies in Pg11), suggesting that the large copine gene set observed in *P. generosa* might have been achieved via tandem duplication of the copine genes in recent evolution. Phylogenetic analysis of the copine genes annotated in *P. generosa* (22 genes) and *S. constricta* (11 genes) revealed good orthologous relationships, as well as one-to-multiple relationships (Figure 7C), confirming that genes inside copine gene clusters in *P. generosa* (Figure 7B) were highly similar.

Most of the copine homologs contain the vWA-domain, however some have both C2-domain and the vWA-domain in *P. generosa* (Figure 7D), like copine genes identified in other species [33]. Lengths of the coding sequences of some copies were comparatively short, which might be due to genome assembly or gene prediction errors. Prediction of 3D

structures using AlphaFold2 [34] revealed that their structures are highly conserved among different species, suggesting the conservation of protein functions. For example, the three-dimensional structure of Pg02g00048 in *P. generosa* showed high similarity to that of XP_0533888641 in *Mercenaria mercenaria* [35] (Figure 7E).

Discussion

Through the completion of this project, we have successfully constructed the first high-quality chromosome-level genome assembly of the ecologically and economically important bivalve, the Pacific geoduck *P. generosa*, enriching the expanding list of chromosome-level genomes of bivalves. The *P. generosa* genome represents the third genome of the third species in the order Adapedonta, after *S. constricta* and *Solen grandis*. The *P. generosa* genome assembly represented another instance of a chromosome-level genome assembly from a bivalve species [11], an ancient lineage of bilaterian, and a diverse Class of Mollusca (Supplementary Table S1).

Comparative analysis between the genomes of *P. generosa* and its most closely related species *S. constricta* revealed extensive inter- and intra-chromosomal exchanges. For example, large chromosomal fragments of *P. generosa pg02* and *pg11* matched to two *S. constricta* chromosomes (*sc01* and *sc10*), respectively (Figure 2). Nevertheless, the numbers of chromosomes of both *P. generosa* and *S. constricta* were still identical (19 chromosomes). Within chromosomes, the order of genes showed even more extensive alterations, resulting in the lack of clear diagonal alignments (Figure 2). Among bivalves the numbers of chromosomes vary substantially, suggesting active genome recombination during the evolution of bivalves [15]. The chromosome numbers of bivalves in the

Infraclass Heteroconchia are 16–19, relatively higher than those in the Infraclass Pteriomorphia which are 10–19. In the Infraclass Pteriomorphia most oysters and scallops possess 19 chromosomes, similar to those of most clams in the Infraclass Heteroconchia. In the phylogenetic analysis of the Infraclass Pteriomorphia the blood clam was closer to the scallops, compared with oysters (Figure 3).

Comparative analysis revealed that the number of PCGs in many gene families with important functions also changes substantially during the evolution of this group (Figure 7A). In particular, the copine gene family was found to be substantially expanded in *P. generosa*, with 22 copine genes identified. The number of copine genes in *P. generosa* was twice of those in *P. constricta*. Many of these 22 copine genes in *P. generosa* formed tandem clusters, with one cluster containing eight copine genes, suggesting that these genes were formed via tandem duplications. The composition of functional domains, which includes the C2 and vWA-domains (Figure 7D), and the similarity of three-dimensional structure to that of known copine genes (Figure 7E), suggests conservation of function of copine genes in *P. generosa*. Thus, the completion of the genome *P. generosa* facilitates comparative analysis of gene families to uncover important leads for exploring molecular insight into its physiology and evolution.

The successful construction of a chromosome-level genome assembly of *P. generosa* not only enables genomic identification and analysis of important genes in this organism, but will also enables comparative analysis of bivalve genomes, which is critical for tracking the species formation, evolution, and biodiversity of bivalves.

Methods

Sampling collection

Geoduck *P. generosa* samples were collected from the Strait of Georgia (49°41′12″N, 124°51′33″W) of British Columbia, Canada in the spring of 2019. The samples showed typical morphological features of *P. generosa*. The identification of the samples was also supported by the high similarities of the molecular marker *cox1* to the reference sequence of *P. generosa* (PID of 99.55, coverage of 100%) [36]. The samples were transferred to laboratory and kept in a tank with running water for a week. One sample was chosen and dissected on ice to collect tissue samples, including labial palp, heart, foot, gonad, gill, hepatopancreas, siphon, and mantle muscle. This animal was identified to be a female as indicated by the presence of eggs in the smear of the gonad under a compound microscope. Dissected tissues were quickly frozen in liquid nitrogen and then stored at −80°C before DNA and RNA extraction.

DNA library construction and sequencing

Genomic DNA of *P. generosa* was extracted using a standard phenol-chloroform extraction method [37]. The quality of DNA was determined by gel electrophoresis to ensure the DNA samples met library sequencing requirements. Sequence libraries with insert size of 300 bp were constructed for BGISEQ-500 sequencing platform (RRID:SCR_017979) according to manufacturer's protocol. The sequencing data produced were used in the genome size estimation by *k*-mer analysis [21] and for correcting errors in the Pilon (RRID:SCR_014731) assembly [38]. A Hi-C library with insert size of 300 bp was constructed to provide long-range information (without position information) on the grouping and linear organization of sequences along entire

chromosomes to assemble the scaffolds into chromosome-level scaffolds [39]. For Hi-C library construction, gonad tissue was dissociated, and cells were collected and crosslinked with 1% formaldehyde (Sigma) and 0.2M glycine (Sigma). After that, the fixed powder was resuspended in nuclei isolation buffer and then incubated in 0.5% SDS for 10 min at 62°C. Then the reaction was quenched with 10% Triton X-100 (Sigma) and the nuclei were collected by centrifugation. Then the DNA was digested with MboI (NEB), and the overhang was filled and biotinylated before ligated by T4 DNA ligase (NEB). Before library construction, the purified DNA was sheared, and biotin-containing fragments were captured on streptavidin-coated beads using Dynabeads MyOne Streptavidin T1 (Invitrogen). The fragments were then end-repaired and linked with adaptors before eight cycles of PCR reaction with KAPA HiFi HotStart ReadyMix (Kapa Biosystem). After that, the Hi-C library was sequenced with BGISEQ-500 platform. And a PacBio library with insert size of 20 Kb was constructed to obtain long reads by the PacBio Sequel platform using the Sequel Sequencing Kit 3.0. The adapters and low-quality reads in raw data generated by the BGISEQ platform were cut off by SOAPnuke1.5.6 using the parameter as "-n 0.01 -l 20 -q 0.1 -i -Q 2 -G -M 2 -A 0.5 -d" [40]. PacBio raw data were filtered with the default parameters by using Pacific Biosciences SMRT analysis software (v2.3.1) to filter the low quality reads.

RNA library construction and sequencing

RNA-Seq and Iso-Seq were conducted to obtain transcriptome data to aid genome annotation. The total RNAs was extracted by Trizol (Invitrogen, Carlsbad, CA, USA) from eight tissues of the same *P. generosa* individual, including labial palp, heart, foot, gonad,

gill, hepatopancreas, siphon, and mantle muscle. The quality and quantity of RNA in each sample was assessed using a NanoDrop and an Agilent 2100 bioanalyzer (Thermo Fisher Scientific, MA, USA). The construction of mRNA libraries for RNA-Seq, the mRNA was enriched by mRNA Capture Beads (BGI, LB00V60), and incubated at 85ºC for 8 minutes for fragmentation. Reverse transcription was performed with Strand Specificity Reagent and 1st Strand Enzyme Mix (Optimal Dual-mode mRNA Library Prep Kit, BGI, LR00R96) to generate the first strand cDNA. After that the second strand cDNA generation and end repair were performed with 2nd Strand Buffer and 2nd Strand Enzyme Master Mix. Then the adaptors (BGI, LA00R04) were ligated to the cDNAs. Then the library was purified and selected depending upon product requirements for amplification. The mRNA libraries were sequenced using the BGISEQ-500 platform. For Iso-Seq, the total RNA was extracted from the equally mixed tissues of the 8 tissues above. The PacBio SMRTbell library was prepared using the SMARTer PCR cDNA Synthesis kit (Clontech), the Qubit dsDNA HS Assay Kit 2.0 (Invitrogen) and the Agilent DNA 12000 kit (Agilent Technologies), and sequenced by the PacBio Sequel sequencer (RRID:SCR_017989) with Sequel Sequencing Kit 3.0.

Genome size estimation and genome assembly

Genome size of *P. generosa* was estimated using *k*-mer analysis. Counting of *k*-mers was conducted using Jellyfish (RRID:SCR_005491, version 2.2.10) [21]. The genome size, heterozygosity, and repeat content were estimated using GCE 1.0.2 [41]. For genome assembly, long reads generated from PacBio Sequel platform were assembled using Falcon (RRID:SCR_016089) [42], which was subsequently polished using Arrow. Short

paired-end clean reads from BGISEQ-500 were then used for correcting post-processing errors and resolving conflicts of assembly via Pilon (RRID:SCR_014731, version 1.22) [38]. The assembled contigs were corrected for mis-joins, orders, orients and anchored contigs from the draft assembly into a candidate chromosome-length assembly by Hi-C data using Juicer (RRID:SCR_017226) [43] and 3d-DNA [44]. The scaffolds shorter than 20 Kb were removed. Finally, the candidate assembly were reviewed with Juicebox Assembly Tools (RRID:SCR_021172) for quality control and interactive corrections [45]. The Hi-C heatmap was visualized using Juicebox (RRID:SCR_021172) (with the bin length of 100 kb) as the interactive signals between each pair of two bins. The completeness of genome assembly was assessed by BUSCO (RRID:SCR_015008, version5.4.3) [25] using the metazoa_odb10 database. The genome landscape illustrating the length, repeat element density, gene density and GC content was created by circos-0.69-9 (RRID:SCR_011798) [46].

Annotations of gene structure and function

Homologous and *de novo* predictions were both applied to annotate transposable elements in the *P. generosa* genome. In homologous prediction, RepeatMasker (RRID:SCR_012954) and RepeatProteinMask [47] were used to screen the *P. generosa* genome for known transposable elements in the RepBase library (RRID:SCR_021169) [48]. In *de novo* prediction, RepeatModeler (version 1.0.4) was first used for de novo candidate database construction of repetitive elements, and repetitive sequences were then annotated using RepeatMasker. Tandem repeats were de novo predicted using Tandem repeats finder (version 4.07) [49]. The results were then integrated and duplicates were

eliminated.

Three complementary approaches were adopted to predict PCGs in the *P. generosa* genome, including homology-based prediction, *de novo* annotation, and transcriptome-based prediction. For homology-based prediction, gene sets from eight closely related bivalves (*P. yessoensis, P. fucata, Mytilus galloprovincialis, Limnoperna fortune, A. purpuratus, S. constricta, S. broughtonii, and C. gigas*) were used. First, protein repertoires of those organisms were aligned against the *P. generosa* genome using TBLASTN (RRID:SCR_011822) [50]. Then gene structures were predicted from these blast hits by Exonerate v2.2.0 [51]. *de novo* gene prediction was performed using a combination of Augustus (RRID:SCR_008417) [52] and SNAP (RRID:SCR_007936) [53] with default settings. Models used for each gene predictor Augustus and SNAP training were obtained from a set of high-quality proteins generated from the RNA-Seq and ISO-seq dataset by MAKER 2 (RRID:SCR_005309). For transcriptome-based prediction using RNA-Seq data, RNA-Seq reads were directly mapped to the genome using TopHat2 (RRID:SCR_013035) [54]. The mapped reads were subsequently assembled into gene models (Cufflinks-set) by Cufflinks (RRID:SCR_014597) [55]. For transcriptome-based prediction based on Iso-Seq data, Iso-Seq reads were directly mapped to the genome using GMAP (RRID:SCR_008992) [56]. The mapped reads were subsequently assembled by PASA (RRID:SCR_014656) [57]. Gene predictions from the homology-based approach, *de novo* approach, RNA-Seq-based and Iso-Seq-based evidences were merged, and redundancy was removed to form a comprehensive consensus gene set using Maker 2 (RRID:SCR_005309) [58]. To validate the completeness of the gene structure annotation,

we also used BUSCO (version5.4.3) with the metazoa_odb10 database [25].

Collinearity analysis

Homologous PCGs in *P. generosa* and *S. constricta* were identified using BLAST v2.11.0 (blastp, E value 1e^{-5}), which were used for subsequent analysis using WGDI [59]. WGDI analysis results included dotplot and syntenic blocks with default parameters.

Phylogenetic analysis and divergence time estimation

Gene families were constructed using the OrthoMCL (RRID:SCR_007839) pipeline [53]. We selected *P. generosa* and other 11 species (*C. gigas*, *P. yessoensis*, *P. maximus*, *A. purpuratus*, *S. broughtonii*, *P. martensi*, *B. platifrons*, *H. sapiens*, *X. tropicaalis*, *D. rerio*, and *C. elegans*) for gene family analysis. For the gene set of each genome, only the transcript with the longest coding sequence was selected from alternate splice transcripts. Genes with less than 50 amino acids were removed from further analysis. Protein sequences were aligned by “all-vs-all BLASTP” (E value = 1e^{-5}) [44]. Then the Markov clustering (MCL) algorithm implemented in OrthoMCL was used to group orthologues and paralogues from all input species with an inflation value of 1.5 [60].

The phylogenetic tree was constructed following procedures described in previous studies [14, 35, 61]. Briefly, for phylogenetic tree construction and divergence time estimation, shared single copy genes of *P. generosa* and 11 other species were used. The protein sequences of single-copy orthologs among the 12 species were aligned using MUSCLE v3.7 (RRID:SCR_011812) [62] with default parameters. Phylogenetic relationships were inferred based on the super-matrix estimated from the concatenated alignment of single-copy genes using the maximum likelihood (ML) [63] method

implemented in RAxML v2.2 (RRID:SCR_006086) [64] with the optimal amino acid substitution model selected by the PROTGAMMALGX parameter.

Based on gene family identification and phylogenetic analysis, single copy genes and mcmctree in PAML [65] were used to estimate divergence time [66-69]. The time correction points were *C. elegans* and *H. sapiens* (678.3–855.2 MYA), *D. rerio* and *H. sapiens* (413.1–443.0 MYA), *X. tropicalis* and *H. sapiens* (347.0–357.9 MYA). The time correction points were taken from the Timetree website. The operating parameters of mcmctree: burn in = 10000, sample number = 1000000, sample frequency = 50.

Gene family analysis and three dimensional protein structure modeling

The clustering results of gene families and the phylogenetic tree with divergence time estimated were used to analyze the expansion and contraction of orthologous gene families between ancestor and each of the 12 species (*P. generosa* and the other 11 species) using a stochastic birth and death model with lambda parameter by CAFE (RRID:SCR_005983, version 4.0) [70]. This model was further used to calculate the number of gene families along each lineage on the phylogenetic tree. A probabilistic graphical model was introduced to calculate the probability of transitions in gene family size from parent to child nodes. The family-wide p-values were calculated in each lineage based on the conditional likelihood.

In addition, we also compared the gene families between *P. generosa* and the other eight bivalves. Pfam domains of *P. generosa* and the other eight bivalves were obtained using InterProscan [32] (5.54-87.0) and visualized using TBtools (v1.120) [71]. The positions of copine genes in *P. generosa* chromosomes were illustrated using R packages

gggenes, and the structure of copine genes were illustrated using Gene Structure Display Server 2.0 on line [72]. Phylogenetic trees of copine genes of *P. generosa* and *S. constricta* were constructed using the maximum likelihood (ML) [63] method implemented in MEGA (v7.0.26) with 1000 bootstrap replicates.

Three-dimensional protein structure models were predicted using AlphaFold2 and visualized using ChimeraX [34].

Data Availability

The Whole Genome project of *P. generosa* has been deposited at NCBI/BioProject PRJNA859289. The raw next-generation sequencing reads of DNA are available at SRA (SRR22190027-SRR22190030); raw long-read PacBio sequencing reads of DNA are available at SRA (SRR22190026; raw next-generation sequencing reads of RNA are available at SRA (SRR22190032); raw Hi-C reads are available at SRA (SRR22190025); and raw long-read PacBio sequencing reads of RNA are available at SRA(SRR22190031). The genome assembly data have been deposited under accession No. JAPMAH000000000.1. All additional supporting data are available in the *GigaScience* repository, GigaDB [73].

Additional Files

Supplementary Figure S1: The 17-mer count distribution for the genome size estimation.

Supplementary Figure S2: The enriched KEGG pathways of significantly expanded gene families (p≤0.01, top 20) in *P. generosa*

Supplementary Table S1: The genome assembly information of bivalves in the public

database

Supplementary Table S2: Statistics of 17-mer analysis

Supplementary Table S3: The chromosomes information of *P. generosa*

Supplementary Table S4: The heterozygosity of bivalves reported

Supplementary Table S5: Functional annotation of the predicted protein-coding genes in *P. generosa* genome assembly

Supplementary Table S6: The assembly quality of chromosomal genomes of bivalves by BUSCO

Supplementary Table S7: The information of 30,616 gene families of *P. generosa* and 11 other species

Supplementary Table S8: The enriched KEGG pathways of expanded gene families in *P. generosa* genome assembly

Abbreviations

PCG: protein-coding gene; Akt: RAC serine/threonine-protein kinase; bp: base pairs; BLAST: Basic Local Alignment Search Tool; BUSCO: Benchmarking Universal Single-Copy Orthologs; BWA: Burrows-Wheeler Aligner; cAMP: cyclic adenosine monophosphate; CAMs: cell adhesion molecules; cGMP-PKG: cGMP-dependent protein kinase G; Gb: gigabase pairs; GC: guanine-cytosine; GnRH: Gonadotropin-releasing hormone; GO: gene ontology; Hi-C: High-throughput/resolution chromosome conformation capture; kb: kilobase pairs; KEGG: Kyoto Encyclopedia of Genes and Genomes; Mb: megabase pairs; MYA: million years ago; NCBI: National Center for Biotechnology Information; NF-kappa B:

nuclear factor kappa-B; NOD-like receptor: nucleotide-binding oligomerization domain-like receptor; PacBio: Pacific Biosciences; RAxML: Randomized Axelerated Maximum Likelihood; PI3K: phosphatidylinositol-4,5-bisphosphate 3-kinase catalytic subunit alpha/beta/delta; Rap1: Ras-related protein1; RNA-Seq: RNA sequencing; Iso-Seq: Isoform-sequencing; tRNA: transfer RNA; TRP channel: transient receptor potential ion channel.

Competing Interests

The authors declare that they have no competing interests.

Funding

This study was supported by the Taishan Scholar Project Special Fund (to Nansheng Chen), the Strategic Priority Research Program of Chinese Academy of Sciences (XDB42000000), the Chinese Academy of Sciences Pioneer Hundred Talents Program (to Nansheng Chen), and an Earmarked Workstation Fund for QRJH (to Chunde Wang and Nansheng Chen).

Authors' Contributions

N.C. and C.W. conceived and designed the study. M.C. and Y.C. prepared the samples. J.W. and Q.X. performed analyses. J.W. wrote the paper with input from co-authors. All authors read and approved the final version for submission.

References

1. González-Peláez SS, Leyva-Valencia I, Pérez-Valencia SA, et al. Distribution limits of the geoduck clams *Panopea generosa* and *P. globosa* on the Pacific coast of Mexico. *Malacologia* 2013;**56**:85-94.
2. Vadopalas B, Pietsch TW, Friedman CS. The proper name for the geoduck: resurrection of *Panopea generosa* Gould , 1850, from the synonymy of *Panopea abrupta* (Conrad, 1849) (Bivalvia: Myoida: Hiatellidae). *Malacologia* 2010;**52**:169-73.
3. Goodwin CL, Pease BC. Geoduck, *Panopea abrupta* (Conrad, 1849), size, density, and quality as related to various environmental parameters in Puget Sound, Washington. *J Shellfish Res* 1991;**10**:65-77.
4. Orensanz JM, Hand CM, Parma AM, et al. Precaution in the harvest of Methuselah's clams the difficulty of getting timely feedback from slow-paced dynamics. *Can J Fish Aquat Sci* 2004;**61**:1355-72.
5. Newell RIE. Ecosystem influences of natural and cultivated populations of suspension-feeding bivalve molluscs: A review. *J Shellfish Res* 2004;**23**:51-61.
6. Straus KM, MacDonald PS, Crosson LM, et al. Effects of geoduck aquaculture on the environment: A synthesis of current knowledge. Washington Sea Grant Technical Report WSG-TR 13-02, 2013.
7. Santos A, Aguirre J, Rodríguez-Tovar FJ, et al. Multi-storm events recorded on *Panopea* burrows (Pliocene, Spain): The importance of sequestered information inside burrows. *Palaeogeogr Palaeoclimatol Palaeoecol* 2018;**507**:155-67.
8. Bureau D., Hajas W., Hand C.M., et al. Age, size structure and growth parameters of geoducks (*Panopea abrupta*, Conrad 1849) from seven locations in British Columbia sampled in 2001 and 2002. Canadian Technical Report of Fisheries and Aquatic Sciences, 2003, p. 29.
9. Valero JL, Canada O, Madryn P, et al. Geoduck (*Panopea abrupta*) recruitment in the Pacific Northwest: long-term changes in relation to climate. *CalCOFI Reports* 2004;**45**:80-6.
10. Sloan NA, Robinson SMC. Age and gonad development in the geoduck clam *Panopea abrupta* (Conrad) from southern British Columbia, Canada. *J Shellfish Res* 1984;**4**:131-7.
11. Liu F, Li Y, Yu H, et al. MolluscDB: an integrated functional and evolutionary genomics database for the hyper-diverse animal phylum Mollusca. *Nucleic Acids Res* 2020;**49**:D988-D997.
12. Thai BT, Lee YP, Gan HM, et al. Whole genome assembly of the snout otter clam, *Lutraria rhynchaena*, using Nanopore and Illumina Data, benchmarked against bivalve genome assemblies. *Front Genet* 2019;**10**:1158.
13. Sun J, Zhang Y, Xu T, et al. Adaptation to deep-sea chemosynthetic environments as revealed by mussel genomes. *Nat Ecol Evol* 2017;**1**:0121.
14. Ran Z, Li Z, Yan X, et al. Chromosome-level genome assembly of the razor clam *Sinonovacula constricta* (Lamarck, 1818). *Mol Ecol Resour* 2019;**19**:1647-58.
15. Halanych KM, Kocot KM. Genome evolution: Shellfish genes. *Nat Ecol Evol* 2017;**1**:0142.

16. Peñaloza C, Gutierrez AP, Eöry L, et al. A chromosome-level genome assembly for the Pacific oyster *Crassostrea gigas*. *GigaScience* 2021;**10**:giab020.
17. Peng J, Li Q, Xu L, et al. Chromosome-level analysis of the *Crassostrea hongkongensis* genome reveals extensive duplication of immune-related genes in bivalves. *Mol Ecol Resour* 2020;**20**:980-94.
18. Wu B, Chen X, Yu M, et al. Chromosome-level genome and population genomic analysis provide insights into the evolution and environmental adaptation of Jinjiang oyster *Crassostrea ariakensis*. *Mol Ecol Resour* 2021;**22**:1529-44.
19. Du X, Fan G, Jiao Y, et al. The pearl oyster *Pinctada fucata martensii* genome and multi-omic analyses provide insights into biomineralization. *GigaScience* 2017;**6**:1-12.
20. Yang JL, Feng DD, Liu J, et al. Chromosome-level genome assembly of the hard-shelled mussel *Mytilus coruscus*, a widely distributed species from the temperate areas of East Asia. *GigaScience* 2021;**10**:giab024.
21. Marçais G, Kingsford C. A fast, lock-free approach for efficient parallel counting of occurrences of k-mers. *Bioinformatics* 2011;**27**:764-70.
22. Gomes-Dos-Santos A, Lopes-Lima M, Machado AM, et al. The Crown Pearl: a draft genome assembly of the European freshwater pearl mussel *Margaritifera margaritifera* (Linnaeus, 1758). *DNA Res* 2021;**28**:1–10.
23. Zhang G, Fang X, Guo X, et al. The oyster genome reveals stress adaptation and complexity of shell formation. *Nature* 2012;**490**:49-54.
24. Zhang T, Yin J, Tang S, et al. Dissecting the chromosome-level genome of the Asian Clam (*Corbicula fluminea*). *Sci Rep* 2021;**11**:15021.
25. Simão FA, Waterhouse RM, Ioannidis P, et al. BUSCO: assessing genome assembly and annotation completeness with single-copy orthologs. *Bioinformatics* 2015;**31**:3210-2.
26. Teng W, Xie X, Nie H, et al. Chromosome-level genome assembly of *Scapharca kagoshimensis* reveals the expanded molecular basis of heme biosynthesis in ark shells. *Mol Ecol Resour* 2022;**22**:295-306.
27. Kanehisa M, Goto S, Kawashima S, et al. The KEGG resource for deciphering the genome. *Nucleic Acids Res* 2004;**32**:D277-D280.
28. Li J, Zhou Y, Zhou Z, et al. Comparative transcriptome analysis of three gonadal development stages reveals potential genes involved in gametogenesis of the fluted giant clam (Tridacna squamosa). *BMC Genomics* 2020;**21**:872.
29. Jiang H, Liu H, Ma X, et al. Transcriptome analysis of *Procambarus clarkii* to screen genes related to ovary development,immunity and growth. *J Fish Chn* 2021;**45**:396-414.
30. Ren Y, Liu W, Pearce CM, et al. Effects of selected mixed-algal diets on growth and survival of early postset juveniles of the Pacific geoduck clam, *Panopea generosa* (Gould, 1850). *Aquac Nutr* 2015;**21**: 152-61.
31. Nava-Gómez GE, Garcia-Esquivel Z, Carpizo-Ituarte E, et al. Survival and growth of geoduck clam larvae (*Panopea generosa*) in flow-through culture tanks under laboratory conditions. *Aquac Res* 2018;**49**:294-300.
32. Zdobnov EM, Apweiler R. InterProScan--an integration platform for the signature-recognition methods in InterPro. *Bioinformatics* 2001;**17**:847-8.

33. Perestenko PV, Pooler AM, Noorbakhshnia M, et al. Copines-1, -2, -3, -6 and -7 show different calcium-dependent intracellular membrane translocation and targeting. *FEBS J* 2010;**277**:5174-89.
34. Jumper J, Evans R, Pritzel A, et al. Highly accurate protein structure prediction with AlphaFold. *Nature* 2021;**596**:583-9.
35. Song H, Guo X, Sun L, et al. The hard clam genome reveals massive expansion and diversification of inhibitors of apoptosis in Bivalvia. *BMC Biology* 2021;**19**:15.
36. Bisbal-Pardo CI, Del Río-Portilla MA, Rocha-Olivares A. The complete mitochondrial DNA of the Pacific Geoduck clam (*Panopea generosa*). *Mitochondrial DNA A DNA Mapp Seq Anal* 2016;**27**:1955-6.
37. Green M, J S. Molecular cloning: a laboratory manual. 4th Edn. Vol. II. New York, NK: Cold Spring Harbor Laboratory Press.
38. Walker BJ, Abeel T, Shea T, et al. Pilon: an integrated tool for comprehensive microbial variant detection and genome assembly improvement. *PloS One* 2014;**9**:e112963.
39. Burton JN, Adey A, Patwardhan RP, et al. Chromosome-scale scaffolding of de novo genome assemblies based on chromatin interactions. *Nat Biotechnol* 2013;**31**:1119-25.
40. Chen Y, Chen Y, Shi C, et al. SOAPnuke: a MapReduce acceleration-supported software for integrated quality control and preprocessing of high-throughput sequencing data. *GigaScience* 2018;**7**:1-6.
41. Liu B, Shi Y, Yuan J, et al. Estimation of genomic characteristics by analyzing *k*-mer frequency in de novo genome projects. *arXiv: Genomics* 2013.
42. Pendleton M, Sebra R, Pang AW, et al. Assembly and diploid architecture of an individual human genome via single-molecule technologies. *Nat Methods* 2015;**12**:780-6.
43. Durand NC, Shamim MS, Machol I, et al. Juicer provides a one-click system for analyzing loop-resolution Hi-C experiments. *Cell Syst* 2016;**3**:95-8.
44. Dudchenko O, Batra SS, Omer AD, et al. De novo assembly of the *Aedes aegypti* genome using Hi-C yields chromosome-length scaffolds. *Science* 2017;**356**:92-5.
45. Robinson JT, Turner D, Durand NC, et al. Juicebox.js provides a cloud-based visualization system for Hi-C data. *Cell Syst* 2018;**6**:256-8.
46. Krzywinski M, Schein J, Birol I, et al. Circos: an information aesthetic for comparative genomics. *Genome Res* 2009;**19**:1639-45.
47. Bergman CM, Quesneville H. Discovering and detecting transposable elements in genome sequences. *Brief Bioinform* 2007;**8**:382-92.
48. Bao W, Kojima KK, Kohany O. Repbase Update, a database of repetitive elements in eukaryotic genomes. *Mobile DNA* 2015;**6**:11.
49. Benson G. Tandem repeats finder: a program to analyze DNA sequences. *Nucleic Acids Res* 1999;**27**:573-80.
50. Altschul SF, Gish W, Miller W, et al. Basic local alignment search tool. *J Mol Biol* 1990;**215**:403-10.
51. Slater GS, Birney E. Automated generation of heuristics for biological sequence comparison. *BMC Bioinformatics* 2005;**6**:31.
52. Stanke M, Morgenstern B. AUGUSTUS: a web server for gene prediction in eukaryotes

that allows user-defined constraints. *Nucleic Acids Res* 2005;**33**:W465-W467.
53. Johnson AD, Handsaker RE, Pulit SL, et al. SNAP: a web-based tool for identification and annotation of proxy SNPs using HapMap. *Bioinformatics* 2008;**24**:2938-9.
54. Kim D, Pertea G, Trapnell C, et al. TopHat2: accurate alignment of transcriptomes in the presence of insertions, deletions and gene fusions. *Genome Biol* 2013;**14**:R36.
55. Trapnell C, Roberts A, Goff L, et al. Differential gene and transcript expression analysis of RNA-seq experiments with TopHat and Cufflinks. *Nat Protoc* 2012;**7**:562-78.
56. Wu TD, Watanabe CK. GMAP: a genomic mapping and alignment program for mRNA and EST sequences. *Bioinformatics* 2005;**21**:1859-75.
57. Haas BJ, Delcher AL, Mount SM, et al. Improving the Arabidopsis genome annotation using maximal transcript alignment assemblies. *Nucleic Acids Res* 2003;**31**:5654-66.
58. Holt C, Yandell M. MAKER2: an annotation pipeline and genome-database management tool for second-generation genome projects. *BMC Bioinformatics* 2011;**12**:491.
59. Sun P, Jiao B, Yang Y, et al. WGDI: A user-friendly toolkit for evolutionary analyses of whole-genome duplications and ancestral karyotypes. *Mol Plant* 2022;15:1841-51.
60. Li L, Stoeckert CJ, Jr., Roos DS. OrthoMCL: identification of ortholog groups for eukaryotic genomes. *Genome Res* 2003;**13**:2178-89.
61. Tian HF, Hu QM, Li Z. A high-quality de novo genome assembly of one swamp eel (*Monopterus albus*) strain with PacBio and Hi-C sequencing data. *G3 (Bethesda)* 2021;11.
62. Edgar RC. MUSCLE: multiple sequence alignment with high accuracy and high throughput. *Nucleic Acids Res* 2004;**32**:1792-7.
63. Guindon S, Gascuel O. A simple, fast, and accurate algorithm to estimate large phylogenies by maximum likelihood. *Syst Biol* 2003;**52**:696-704.
64. Stamatakis A. RAxML-VI-HPC: maximum likelihood-based phylogenetic analyses with thousands of taxa and mixed models. *Bioinformatics* 2006;**22**:2688-90.
65. Yang Z. PAML 4: phylogenetic analysis by maximum likelihood. *Mol Biol Evol* 2007;**24**:1586-91.
66. Thorne JL, Kishino H, Painter IS. Estimating the rate of evolution of the rate of molecular evolution. *Mol Biol Evol* 1998;**15**:1647-57.
67. Vogel JP, Garvin DF, Mockler TC, et al. Genome sequencing and analysis of the model grass Brachypodium distachyon. *Nature* 2010;**463**:763-8.
68. Blanc G, Wolfe KH. Widespread paleopolyploidy in model plant species inferred from age distributions of duplicate genes. *Plant Cell* 2004;**16**:1667-78.
69. Sanderson MJ. r8s: inferring absolute rates of molecular evolution and divergence times in the absence of a molecular clock. *Bioinformatics* 2003;**19**:301-2.
70. Han MV, Thomas GW, Lugo-Martinez J, et al. Estimating gene gain and loss rates in the presence of error in genome assembly and annotation using CAFE 3. *Mol Biol Evol* 2013;**30**:1987-97.
71. Chen C, Chen H, Zhang Y, et al. TBtools: An integrative toolkit developed for interactive analyses of big biological data. *Mol Plant* 2020;**13**:1194-202.
72. Hu B, Jin J, Guo AY, et al. GSDS 2.0: an upgraded gene feature visualization server. *Bioinformatics* 2015;**31**:1296-7.

73. Wang J, Xu Q, Chen M, et al. Supporting data for "Chromosome-level genome assembly of the Pacific geoduck Panopea generosa" GigaScience Database. 2023. http://dx.doi.org/10.5524/102469.

Figure 1: The *P. generosa* genome construction and and genome landscape. (A) Hi-C analysis of *P. generosa* genome contigs. Chromosomes are arranged in the size order from left to right and from top to bottom. The color bar illuminates the logarithm of the contact density from red (10) to white (0) in the plot. (B) The genomic landscape of *P. generosa* (from outer to inner circles): a, the 19 chromosomes; b–d, repetitive element density, gene density, and GC density across the genome, respectively, drawn in 1 Mb non-overlapping windows.

Figure 2: Chromosome synteny of *P. generosa* and *S. constricta*. (A) A dotplot displaying correspondence of homologous genes in *P. generosa* and *S. constricta*. The red, blue, and gray dots present three similarity levels of homologous gene pairs between *P. generosa* and *S. constricta*. The best match genes are shown in red, while the next best match genes in blue and the least best match genes in gray. (B) A CIRCOS plot of synteny analysis between *P. generosa* and *S. constricta*. Lines in different colors depict different interchromosomal synteny.

Figure 3: Phylogenetic analysis of *P. generosa* with related species. The estimated species divergence time (million years ago) and the 95% confidential intervals are labeled at each branch site. Divergence times used for time recalibration were illuminated as red dots in the tree. Numbers of chromosomes of each Bivalvia species were shown.

Figure 4: The distribution of single-copy orthologs, multiple-copy orthologs, unique

paralogs, other orthologs, and unclustered genes in *P. generosa* and related species.

Figure 5: Distribution of shared gene families among *P. generosa*, *P. martensi*, *S. broughtonii*, and *P. yessoensis*. Intersections between species indicate the numbers of shared gene families, whereas unique family numbers are shown in species-specific areas. The center represents the number of families shared by all the 4 species.

Figure 6: Dynamic evolution and distribution of gene families among *P. generosa* and related species. The numbers of gene gains (+) and losses (−) are shown on the branches, which are also displayed as pie plots: the green part for gene gain, the red part for gene losses and the blue part for gene remained. The divergence times are dated and displayed below the phylogenetic tree. MRCA: most recent common ancestor.

Figure 7: Comparative analysis of gene families based on Pfam annotation in *P. generosa* and related bivalves. A. The 65 most abundant gene families that *P. generosa* possessing more gene numbers than 8 other bivalves. B. Chromosomal distribution of copine genes in *P. generosa genome*. C. The phylogenetic tree of copine genes in *P. generosa* and *S. constricta*, shown in the background colors of red and pink, respectively.D. Domain structures of copine genes predicted in *P. generosa* genome. E. The 3D structures of Pg02g00048 gene in *P. generosa* and XP_0533888641 gene in *Mercenaria mercenaria*.

Table 1: Statistics of the DNA sequence data used for *P. generosa* genome assembly

Source	Platform	Library size	Clean data (Gb)	Read length (bp)	Sequencing coverage (x)
**Genome-short reads**	BGISEQ-500	300 bp	258.19	150	181
**Genome-long reads**	PacBio sequel	20 Kb	164.46	26,513*	115
**Hi-C**	BGISEQ-500	300 bp	233.49	150	163

[a]"26,513*" indicated the N50 of subreads.

Table 2: Statistics of the genome assembly of *P. generosa*

Statistics	Contig	Scaffold	Chromosome
**Total Number (#)**	2,086	39	19
**Total length (bp)**	1,473,137,789	1,474,161,289	1,432,060,667
**Average Length (bp)**	706,202	37,799,007	75,371,614
**N50 Length (bp)**	1,571,249	73,788,920	76,670,739
**N90 Length (bp)**	418,579	53,843,121	53,843,121
**Maximum Length (bp)**	6,469,558	101,196,518	101,196,518
**Minimum Length (bp)**	17	29,000	1,432,060,667
**GC content**	34.33%	34.33%	34.33%
**Anchored rate (%)**	94.70%		

Table 3: Repetitive element annotations in *P. generosa*

~~Repetitive sequence~~			~~Transposable elements~~		
~~Type~~	~~Length (bp)~~	~~% in genome~~	~~Type~~	~~Length (bp)~~	~~% in genome~~
~~DNA~~	~~318426608~~	~~21.601~~	~~DNA~~	~~318426608~~	~~21.601~~
~~LINE~~	~~134171392~~	~~9.102~~	~~LINE~~	~~134171392~~	~~9.102~~
~~SINE~~	~~27249093~~	~~1.848~~	~~SINE~~	~~27249093~~	~~1.848~~
~~LTR~~	~~55380608~~	~~3.756~~	~~LTR~~	~~55380608~~	~~3.756~~
~~Other~~	~~31141~~	~~0.002~~	~~Other~~	~~31141~~	~~0.002~~
~~Unknown~~	~~151891763~~	~~10.304~~	~~Unknown~~	~~151891763~~	~~10.304~~
~~Tandem repeat~~	~~347641207~~	~~23.582~~	~~Total~~	~~583000390~~	~~39.548~~
~~Total~~	~~854873570~~	~~57.991~~			

	**Type**	**Length (bp)**	**% in genome**
	DNA	318426608	21.601
	LINE	134171392	9.102
	SINE	27249093	1.848
**Transposable elements**	LTR	55380608	3.756
	Other	31141	0.002
	Unknown	151891763	10.304
	Total	583000390	39.548
**Tandem repeat**		347641207	23.582
**Total**		854873570	57.991

Table 4: BUSCO results for analysis of genome completeness for *P. generosa*

Type	Genome assembly		Gene set	
	Number of genes	Percentage (%)	Number of genes	Percentage (%)
**Complete BUSCOs (C)**	887	93.0	844	88.4
**Complete and single-copy BUSCOs (S)**	842	88.3	795	83.3
**Complete and duplicated BUSCOs (D)**	45	4.7	49	5.1
**Fragmented BUSCOs (F)**	34	3.6	49	5.1
**Missing BUSCOs (M)**	33	3.4	61	6.5
**Total BUSCO groups**	954	100	954	100

Table 5: The PCGs of *P. generosa* and 11 other species for evolutionary analysis

Species	Total genes	Unclustered genes	Families	Unique families	Ave. genes per family
P. generosa	35,034	6,168	12,034	1,749	2.4
A. purpuratus	26,256	3,720	13,196	290	1.71
B. platifrons	33,584	3,197	12,409	1,775	2.45
C. gigas	28,402	3,638	11,818	828	2.1
D. rerio	25,444	1,791	9,210	295	2.57
H. sapiens	20,229	1,488	9,251	226	2.03
P. yessoensis	24,521	1,704	13,017	137	1.75
P. maximus	26,152	1,518	13,276	164	1.86
P. martensi	25,526	2,403	11,043	318	2.09
S. broughtonii	24,045	2,770	11,314	538	1.88
X. tropicalis	19,967	1,016	9,226	159	2.05
C. elegans	33,552	5,600	8,201	3,720	3.41

Figure 6 Click here to access/download;Figure;Figure6.pdf

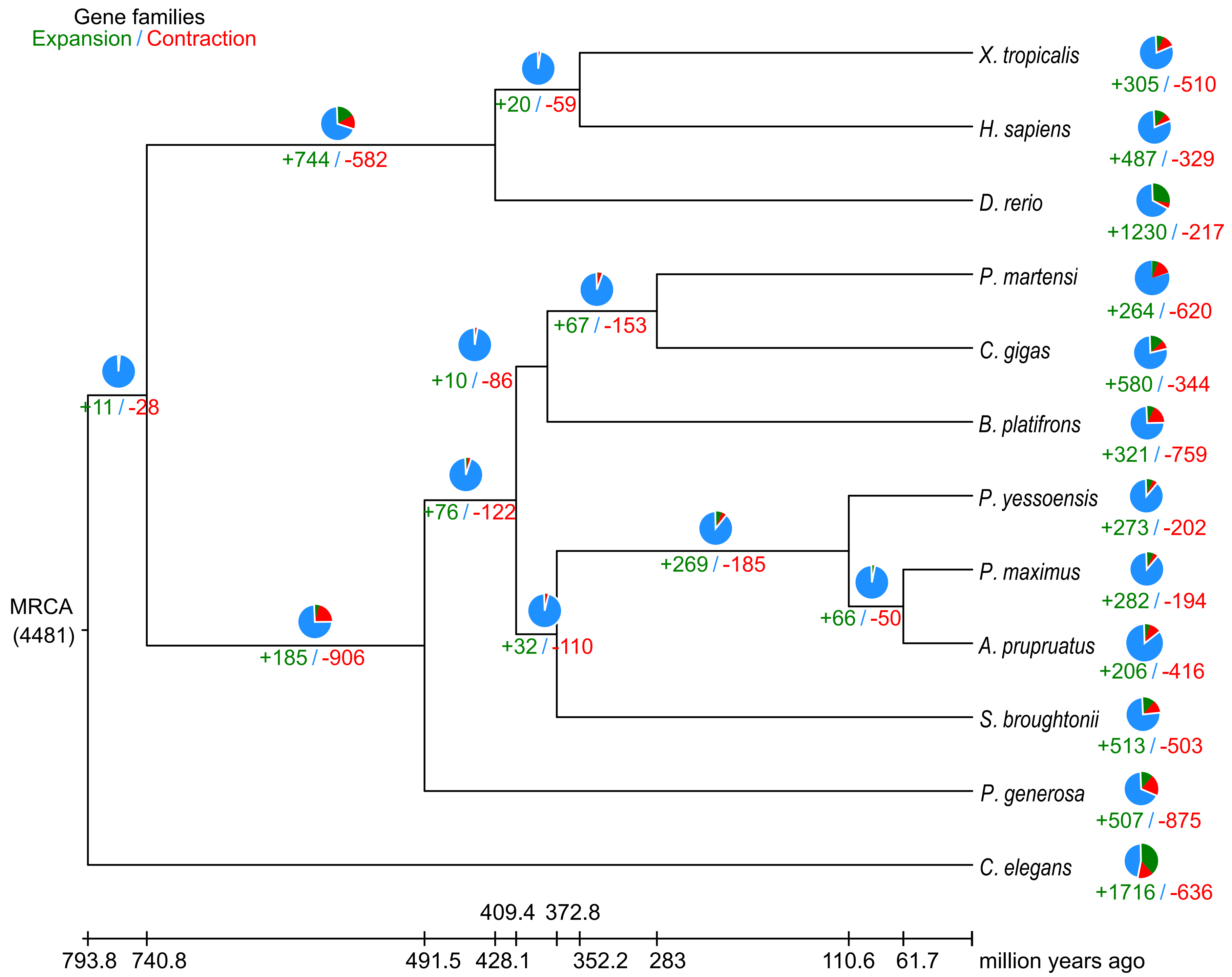

Figure1

Click here to access/download;Figure;Figure1.pdf

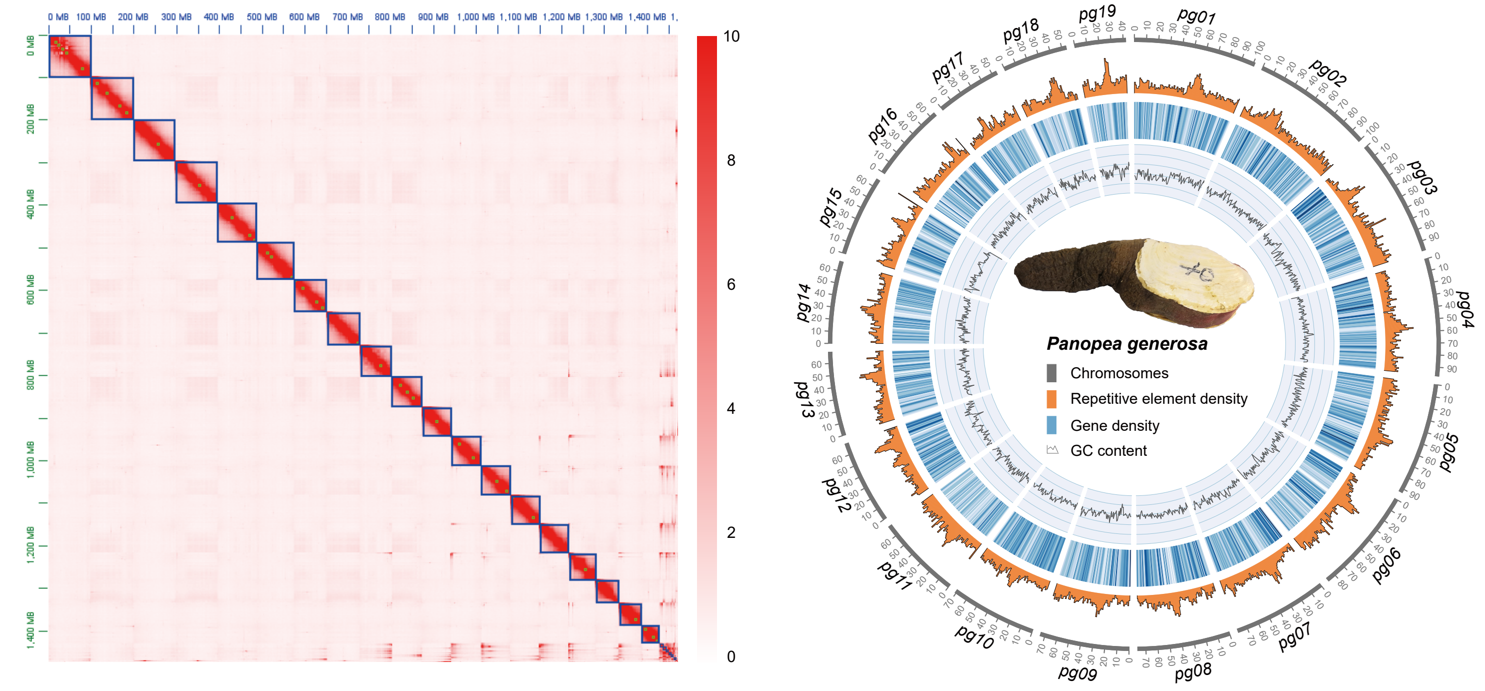

Figure2

Click here to access/download;Figure;Figure2.pdf

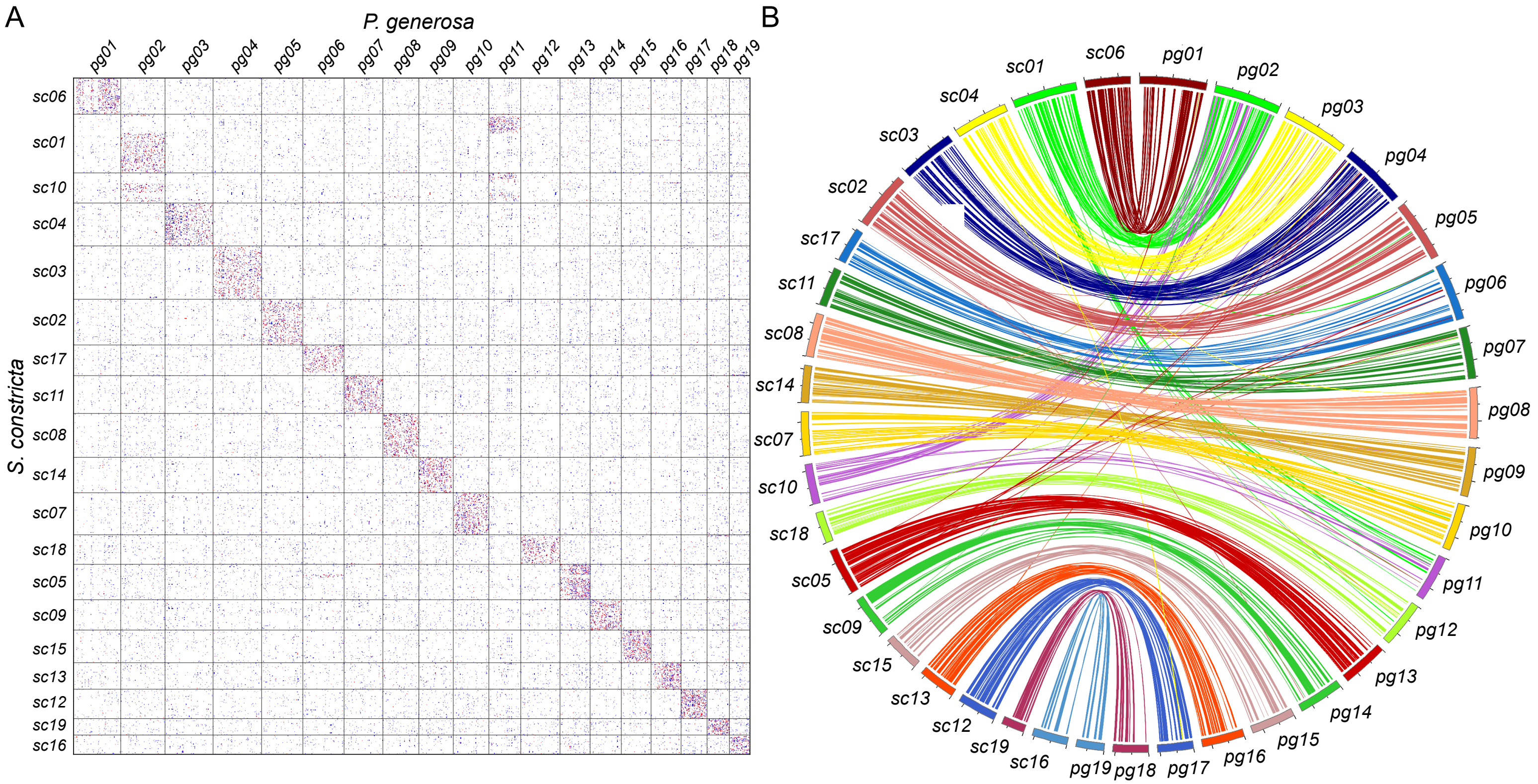

Figure4

Click here to access/download;Figure;Figure4.pdf

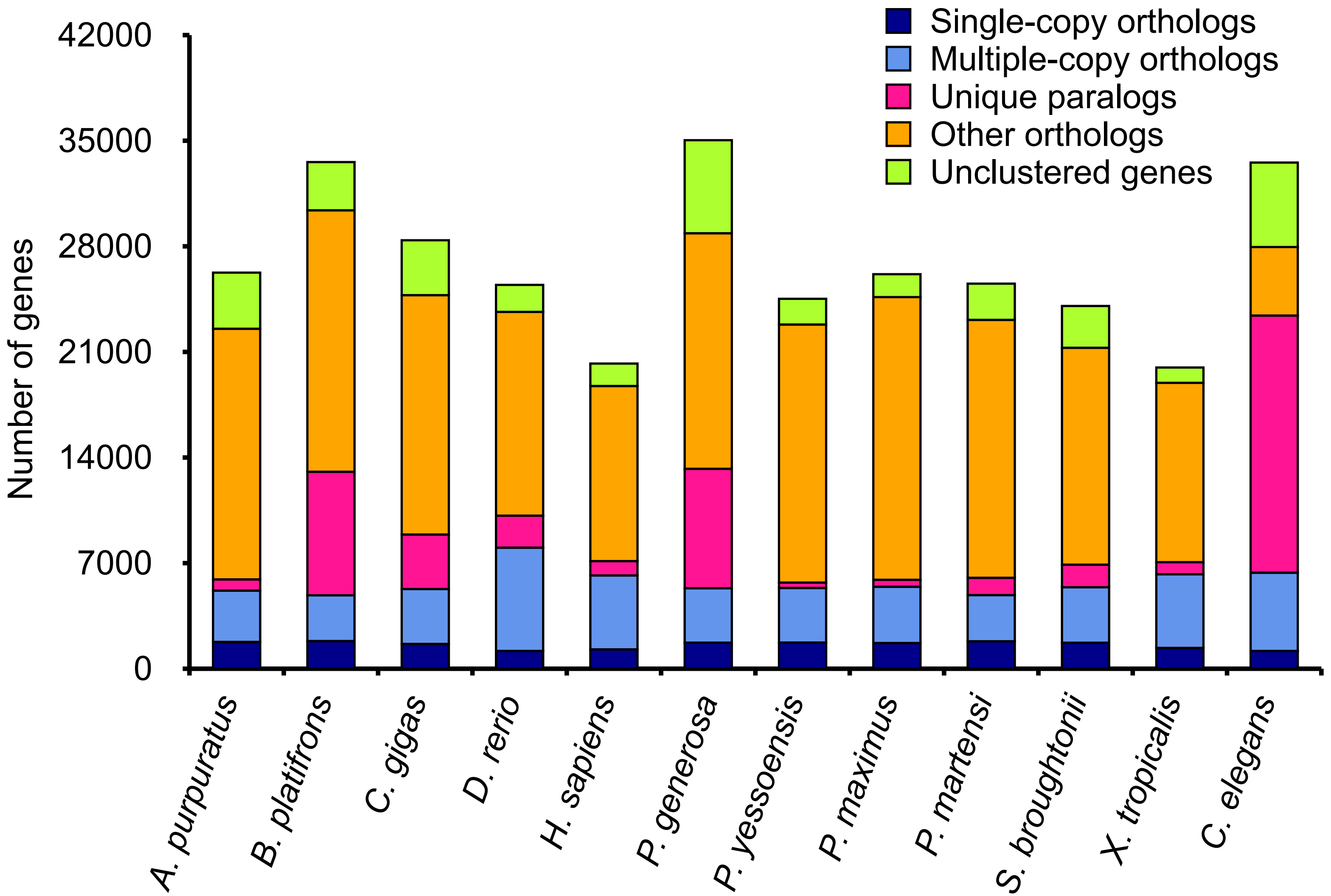

Figure5

Click here to access/download;Figure;Figure5.pdf

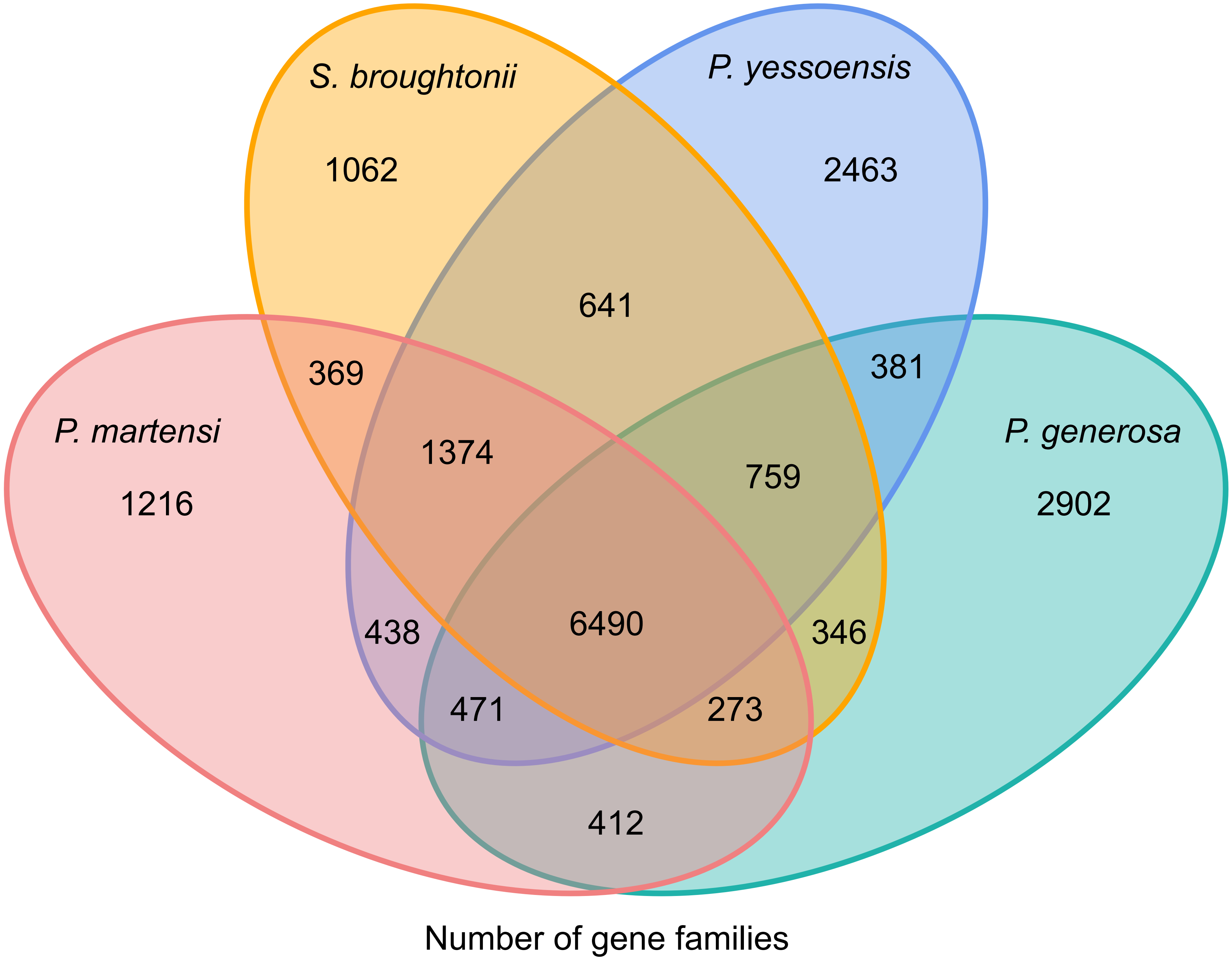

Figure7

Click here to access/download;Figure;Figure7.pdf

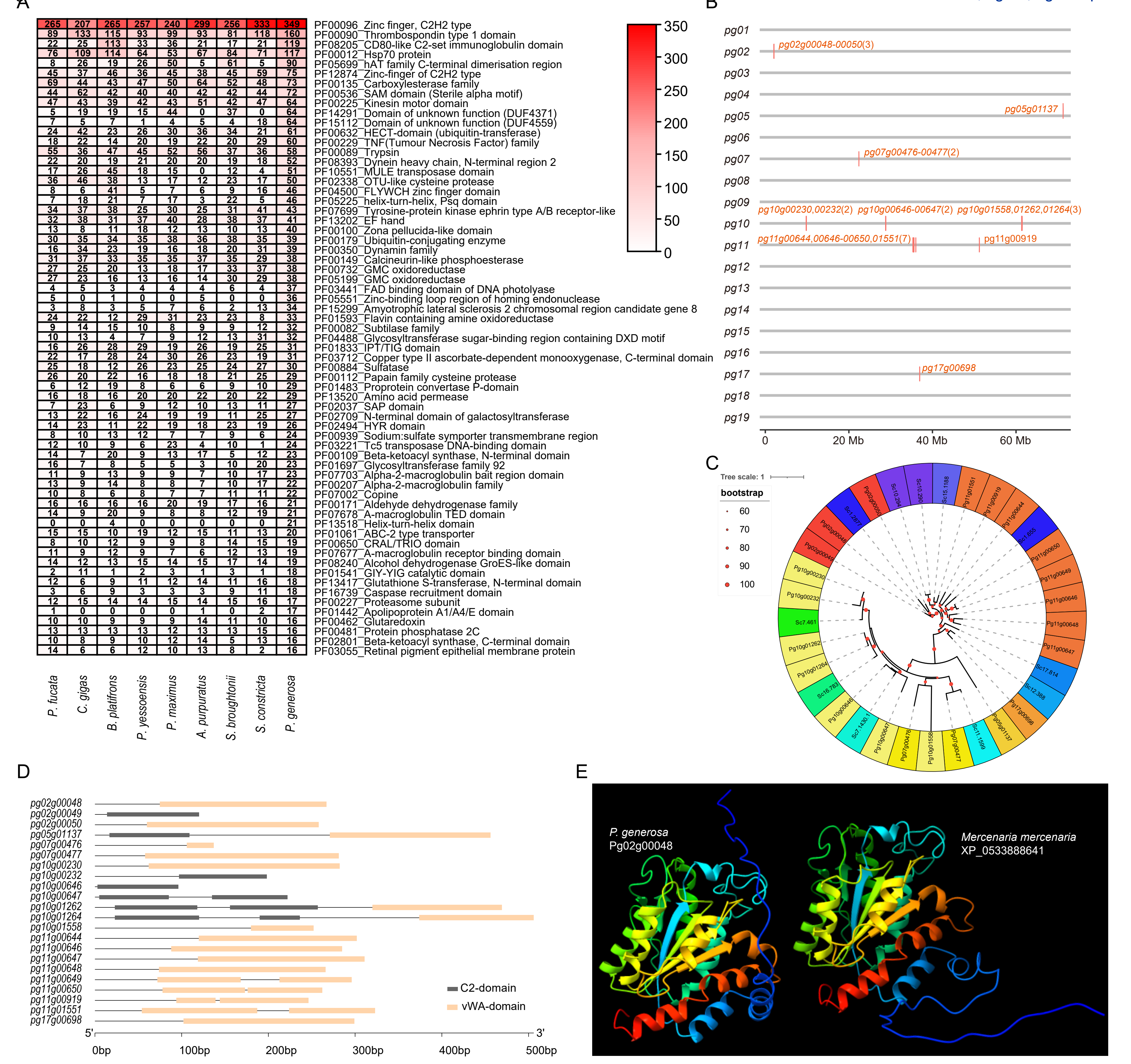

Figure 3

Click here to access/download;Figure;Figure3.pdf

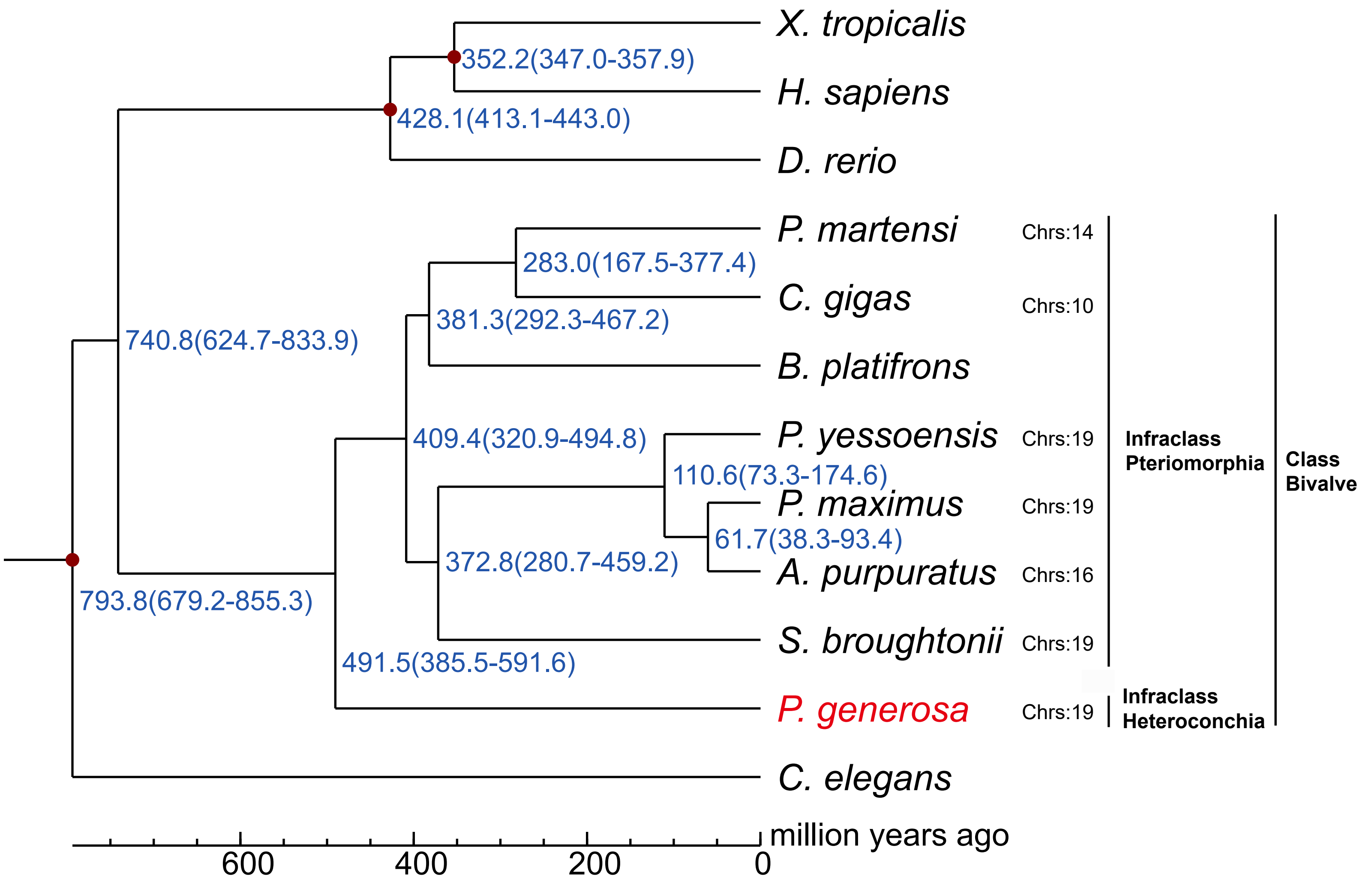

Supplementary figures

Click here to access/download
Supplementary Material
Supplementary figures.docx

Supplementary tables

Click here to access/download
Supplementary Material
Supplementary tables.xlsx
