# Supplementary material for: Chromosome-level genome assembly of the Pacific geoduck Panopea generosa reveals major inter- and intrachromosomal rearrangements and substantial expansion of the copine gene family
Source: Gigascience. 2023 Dec 19;12:giad105. doi: 10.1093/gigascience/giad105 (PMC10729735; doi:10.1093/gigascience/giad105)
Supplement: giad105_Supplemental_Tables_and_Figures [file giad105_supplemental_tables_and_figures.zip › Supplementary figures.docx]

**Chromosome-level genome assembly of the Pacific geoduck *Panopea generosa* reveals major inter- and intra-chromosomal rearrangements and substantial expansion of the copine gene family**

Jing Wang^1,2,3^, Qing Xu^1,2,3^, Min Chen^4^, Yang Chen^1,2,3^, Chunde Wang^4,5*^, Nansheng Chen^1,2,3,6*^

^1^CAS Key Laboratory of Marine Ecology and Environmental Sciences, Institute of Oceanology, Chinese Academy of Sciences, Qingdao, China

^2^Laboratory of Marine Ecology and Environmental Science, Qingdao National Laboratory for Marine Science and Technology, Qingdao, China

^3^Center for Ocean Mega-Science, Chinese Academy of Sciences, Qingdao, China

^4^Yantai Institute of Coastal Zone Research and Center for Ocean Mega-Science, Chinese Academy of Sciences, Yantai, China

^5^Marine Science and Engineering College, Qingdao Agricultural University, Qingdao, China

^6^Department of Molecular Biology and Biochemistry, Simon Fraser University, Burnaby, BC, Canada


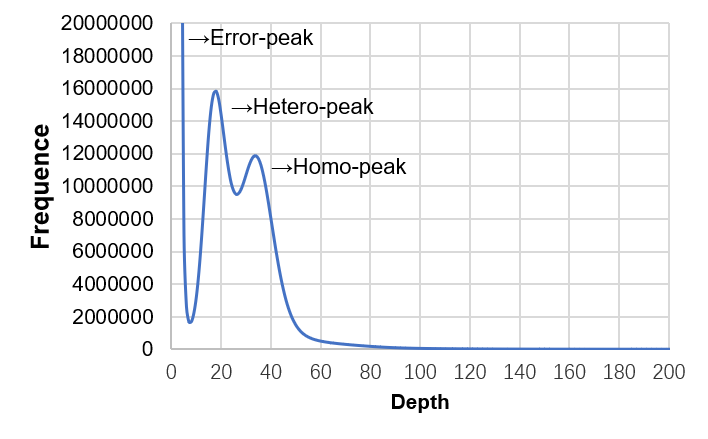


**Supplementary** **Figure S1:** The 17-mer count distribution for the genome size estimation. Note that the peaks around the depths of 18 and 36 represent the heterozygous, and main peak, respectively. The genome size, heterozygouse rate, and repeat content of *P. generosa* were estimated to be 1.47 Gb, 1.37% and 68.08% respectively.

**
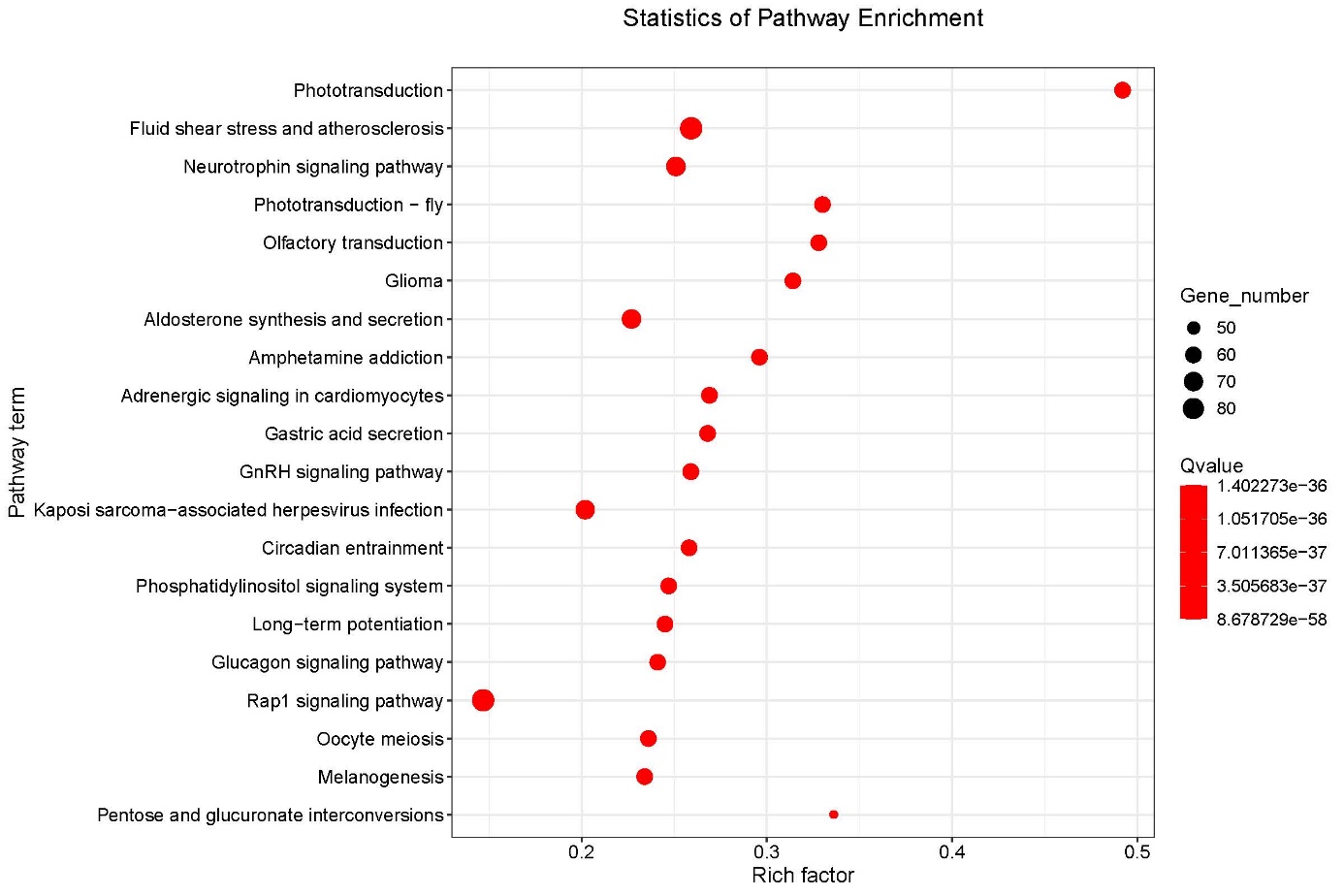
**

**Supplementary Figure S2:** The enriched KEGG pathways of significantly expanded gene families (p≤0.01, top 20) in *P. generosa*.
