# Supplementary material for: Chromosome-level genome assembly of the Pacific geoduck Panopea generosa reveals major inter- and intrachromosomal rearrangements and substantial expansion of the copine gene family
Source: Gigascience. 2023 Dec 19;12:giad105. doi: 10.1093/gigascience/giad105 (PMC10729735; doi:10.1093/gigascience/giad105)
Supplement: giad105_Response_to_Reviewer_Comments_Original_Submission [file giad105_response_to_reviewer_comments_original_submission.pdf]

Author's Response To Reviewer Comments

Reviewer #1: The manuscript by Wang et al. reports the high-quality chromosome-level assembly of the economically important Pacific geoduck Panopea generosa. While I believe that this work will be of significant value to the scientific community, the manuscript requires significant rephrasing and additional details added before it can be published. Specifically, a lot of additional details need to be provided in the methods section, including which tools were used, their versions and parameters, as well as clarification of the points that I detail below. Rephrasing and additional details are also required in the results section to improve understanding by the reader. I also could not find Supplementary Tables S2 and S6 online. If I have missed them then I apologies, however, if they we not uploaded then this needs to be corrected for the next round of reviewers. Please see my specific comments below.
Response 1: We thank the reviewer for confirming the value of our work and for providing valuable suggestions for improving the manuscript. We have made additional analyses and have substantially revised the manuscript. Details are provided below.

Page 5: "They are preys for" should read "They are prey for"
Response 2: Corrected.

Page 5: "shell concentrations" of what? Did you mean "shell composition"? Also, I am unsure what you mean by "sequence of events related to storm events". What specifically are these events that are related to storms?
Response 3: These are terms used in palaeogeography, palaeoclimatology, and palaeoecology. The “shell concentrations” is used as a tool for palaeoenvironmental reconstructions and palaeoecological interpretations, has been increasingly recognized in the last decades with a wealth of publications (Santos A et al., Palaeogeography, Palaeoclimatology, Palaeoecology. 2018;507:155-67.). As Panopea species may reach 160 years in age and usually occupies a dynamic depositional setting, the trace fossils produced by this bivalve may provide a clear record of the long-term ability to respond to changes in the physical environments. As a result, they may be used as proxies for estimating the magnitude and frequency of sedimentation and erosion events.

Page 6: "gene family expansion and contraction" should probably read "gene family expansion and contraction analysis"
Response 4: Revised.

Page 6: "Genomic DNA of P. generosa was extracted using a standard phenol-chloroform extraction method" I understand that this process is commonly used for DNA extraction however additional detail about your exact application of the method or at minimum references to the protocol that you followed is required to assist the reader.
Response 5: A reference is added in the revised manuscript.

Page 7: "Sequence libraries with insert size of 300 bp were constructed" by who? A service provider? Using which protocol? This needs to be listed.
Response 6: Sequencing libraries were constructed by a sequence company, BGI Genomics, BGI-Shenzhen (China) using protocols according to manufacturer, which is Whole Genome Sequencing Library Preparation (DNBSEQ) BGI-NGS-JK-DNA-001 A0”. This information is included in the revised manuscript.

Page 7: "genome size estimation, k-mer analysis", weren't k-mers used to estimate genome size? This sentence makes it sound like they are two separate processes.
Response 7: We have revised this sentence in the revised manuscript.

Page 7: "Hi-C library with insert size of 300 bp was constructed". Who constructed the library, using what protocol, and which company actually did the sequencing?
Response 8: The sequence libraries were constructed by a sequence company, BGI Genomics, BGI-Shenzhen (China). The protocol has been supplemented in the main text.

Page 7: "The raw sequence data generated …..". List programs, versions, and parameters used to filter the BGIseq and PacBio reads.
Response 9: Programs, versions, and parameters used to filter the BGISEQ and PacBio reads have been added in the revised manuscript.

Page 7: "Each RNA sample was qualified and". I don't think that this is the correct use of the word "qualified". Maybe try "The quality and quantity of RNA in each sample was assessed using a NanoDrop …."
Response 10: The description has been rephrased as "The quality and quantity of RNA in each sample was assessed using a NanoDrop and an Agilent 2100 bioanalyzer (Thermo Fisher Scientific, MA, USA)".

Page 7: "For RNA-seq, the mRNA library was constructed by purification, fragmentation, a first-strand cDNA generation, a second-strand cDNA synthesis, and RNA index addition." List kits and protocols used for this analysis.
Response 11: The detailed information of kits and protocols used for RNA-seq has been added in the revised manuscript.

Page 8: "Counting of k-mers was conducted using Jellyfish". Which other tool or method was used to estimate genome size after k-mers were produced by jellyfish? Based on Figure S1 I would guess that you used your own workflow for genome size estimation. A description and references for this approach is required. Also, the "k" in k-mers should be italicized through the manuscript.
Response 12: We only applied jellyfish for estimating the genome size in this project, which is important for determining the quantity of PacBio sequencing needed for the genome assembly. Spelling has been revised in the revised manuscript.

Page 8: "Short paired-end clean reads from BGIseq-500 were then polished using Pilon" The short reads were used by Pilon to correct the assembly? This sentence makes it seem like Pilon was used to correct the short reads, which I don't think is correct.
Response 13: The sentence has been corrected as "Short paired-end clean reads from BGISEQ-500 were then used for correcting post-processing errors and resolving conflicts of assembly via Pilon (RRID:SCR_014731, version 1.22) [33]".

Page 8: "Chromosomes were further corrected using Juicebox". More detail is required here since I assume you did some manual corrections using Juicebox. If you just used an automated correction workflow then these details need to be given.
Response 14: We have rephrased this part in the revised manuscript.

Page 8: "The completeness of genome assembly was assessed using BUSCO". Using which lineage dataset?
Response 15: The metazoa_odb10 was used in the BUSCO analysis. This information is added in the revised manuscript.

Page 8: (1) "In homologous prediction, RepeatMasker (RepeatMasker, RRID:SCR_012954) and the associated RepeatProteinMask [18] were performed by alignment against Repbase library (v21.12)". This sentence is a little hard to follow. I assume that you mean the repeats from Repbase were aligned against the reference genome using the mentioned programs? Based on how it is currently phrased, it is unclear what is being aligned against Repbase. (2) Also please provide details on any parameters given to each program when they are run. The following sentence can also be restructured to make it clear that repeats were identified de novo in the genome assembly and what the parameters used for each of the tools were.
Response 16:
(1) This sentence has been corrected as "In homologous prediction, RepeatMasker (RepeatMasker, RRID:SCR_012954) and RepeatProteinMask [18] were used to screen the P. generosa genome for known transposable elements in the RepBase library (Repbase, RRID:SCR_021169) [19]".
(2) The following sentence has been corrected as "In de novo prediction, RepeatModeler (version 1.0.4) was first used for de novo candidate database construction of repetitive elements, and repetitive sequences were then annotated using RepeatMasker.".

Page 8: "Besides, tandem repeats". Remove the word "Besides" from this sentence.
Response 17: Removed.

Page 9: "The results were then integrated and redundancy was eliminated." How was redundancy eliminated? Using which tool?
Response 18: For clarity, we have changed "redundancy" to "duplicate".

Page 9: "from different branches were chosen to confirm the completeness of the gene set." I am unsure what you mean by this sentence. Do you mean one genome per taxonomic group was selected? What is mean by "to confirm the completeness"?
Response 19: This sentence has been rephrased as "For homology-based prediction, gene sets from eight closely related species in bivalves (Patinopecten yessoensis, Pinctada fucata, Mytilus galloprovincialis, Limnoperna fortune, Argopecten purpuratus, Sinonovacula constricta, Scapharca broughtonii, and Crassostrea gigas) were used".

Page 9: "hits were then conjoined by Solar software (SOLAR, RRID:SCR_000850) [25]." I don't think that this software is a gene prediction tool. From what I can tell it is a QLT analysis tool. Is this correct?
Response 20: Exonerate v2.2.0 was used to predict the gene structures.

Page 9: "For de novo annotation, six programs were simultaneously used…..". Version numbers and parameters are required for each of these tools.
Response 21: Details of the version numbers and parameters are provided in the revised manuscript.

Page 9: "Among them, Augustus, snap and GlimmerHMM were trained using PASA-T-set gene models." What is the "PASA-T-set"? This set of (I assume) RNA-seq based gene modes is never defined in the text.
Response 22: We have rephrased this part in the revised manuscript.

Page 10: "Gene predictions from the homology-based approach, …." List version, parameters, and evidence weights used for EvidenceModeler. Also, list lineage dataset used for BUSCO analysis.
Response 23: The description has been rephrased as "Gene predictions from the homology-based approach, de novo approach, RNA-Seq-based and Iso-Seq-based evidences were merged, and redundancy was removed to form a comprehensive consensus gene set using Maker 2 (RRID:SCR_005309) [52]". To validate the completeness of the gene structure annotation, we also used BUSCO (version5.4.3) with the metazoa_odb10 database [23].

Page 10: "Gene families were constructed according to OrthoMCL (OrthoMCL DB: Ortholog Groups of Protein Sequences, RRID:SCR_007839) pipeline" should probably read "Gene families were constructed using the OrthoMCL (OrthoMCL DB: Ortholog Groups of Protein Sequences, RRID:SCR_007839) pipeline"
Response 24: Corrected.

Page 10: "… Caenorhabditis elegans) were filtered. When multiple …" The transition between these two sentences is a little hard to follow. When I first read it, I didn't immediately understand that the following sentence lists the filtering steps. Please rephrase this section.
Response 25: These two sentences were rephrased as "We selected P. generosa and other 11 species (C. gigas, P. yessoensis, P. maximus, A. purpuratus, S. broughtonii, Pinctada martensi, Bathymodiolus platifrons, Homo sapiens, Xenopus tropicaalis, Danio rerio, and Caenorhabditis elegans) for gene family analysis. For the gene set of each genome, only the transcript with the longest coding sequence was selected from alternate splice transcripts. Genes with less than 50 amino acids were removed from further analysis.".

Page 10: "(E value = 1e-5 by default) [24]" what does the "by default" mean here? If you are setting the e-value to be 1e-5 then you are not running the program using default parameters?
Response 26: The words "by default" have been removed from the manuscript.

Page 10-11: "SCR_007839) 2.0 with the expansion coefficient 1.5 [38]. Finally, to perform multiple sequence alignment for single copy genes clustered, and concatenate sequence alignment of single-copy gene families. Based on the clustering results, the division of gene family was obtained. To construct phylogenetic trees, protein sequences of each single copy gene of P. generosa and other 11 species were

aligned using MUSCLE (MUSCLE, RRID:SCR_011812) [39], and then the protein alignment results were converted into CDS alignment results."
I am slightly confused by this section. You selected gene clusters that were single copy in how many species? What does "Based on the clustering results, the division of gene family was obtained" Mean? Do you mean that each cluster was considered a gene family? How were the protein alignment converted to CDS alignment? Using which tool? Please rephrase this section to make your approach clearer.
Response 27:
(1) We selected gene clusters that were single copy in P. generosa and 11 other species (C. gigas, P. yessoensis, P. maximus, A. purpuratus, S. broughtonii, Pinctada martensi, Bathymodiolus platifrons, Homo sapiens, Xenopus tropicaalis, Danio rerio, and Caenorhabditis elegans).
(2) The sentence "Based on the clustering results, the division of gene family was obtained" was removed in the revised manuscript.
(3) Each of the single-copy gene cluster consists of a single gene from each species. The construction procedures of the phylogenetic tree have been corrected in the revised manuscript.

Page 11: "Next, conserved sites were extracted ….." So you only used conserved sites for phylogenetic analysis? Conserved in how many species? All of them? Were these used for phylogenetic analysis? I assume not since if they are conserved (i.e., no variation between species) then they wouldn't tell you anything about how the species are related.
Response 28: The "conserved sites" should be better described as alignable sites.

Page 11: "The phylogenetic tree was constructed …." So, a single model was selected to represent the whole super alignment? Since each single copy gene family used to create the super alignment would likely have a slightly different optimal model, does it make sense to pick just a single model for all of them? Why not perform a partition aware phylogenetic analysis (like what is implemented in the IQtree program)?
Response 29: The phylogenetic tree was constructed following procedures described previous studies (Tian et al., 2021; Ran et al., 2019; Song et al., 2021).

Page 11: "The clustering results of gene families …" Were the gene families filtered in any way before being used for this analysis? You also use the term "orthologous gene families", which you hadn't before in the text, it would help the reader if you picked a single way of referring to the clusters and used it throughout the text.
Response 30: A sentence was added in the revised manuscript: "Then the Markov clustering (MCL) algorithm implemented in OrthoMCL was used to group orthologues and paralogues from all input species with an inflation value of 1.5".

Page 11: "A probabilistic graphical model …." Implemented in which program? Same with the following sentence, which tool calculated the p-values?
Response 31: They were all done by CAFÉ.

Page 12: "Genome survey analysis using …" Do you mean "genome size estimation using k-mers"?
Response 32: Yes. This sentence has been corrected as "The genome size of P. generosa was estimated to be 1.47 Gb using k-mer analysis (Table 1)".

Page 12: "included 39 scaffolds anchored to 19 chromosomes with an anchoring rate of 94.70%." I do not quite understand what you mean by this statement. So, there are 39 scaffolds that comprised 19 chromosomes? Or 19 of the scaffolds are the main chromosomes and 20 are unplaced scaffolds? Also, I have trouble believing that you only got 39 scaffolds from the assembly workflow. You will always get a lot of small scaffolds; I assume that these were removed at some stage, but I see no discussion of this in the methods. This need to be added so that people know how you generated an assembly with so few scaffolds.
Response 33: The P. generosa genome consists of 39 scaffolds whose lengths longer than 20 Kb, among which 19 chromosomes being the main chromosomes and 20 being unplaced scaffolds. We have supplemented the information in the main text.

Page 14: I could not find Supplementary Table S2 available anywhere, so I was unable to assess this section.

Response 34: It is now called Supplementary Table S1 in the revised manuscript.

Page 14: "and Mytilus edulis have14 chromosomes" add a space between "have 14"
Response 35: Corrected.

Page 14: Which tools were used to create the images in Figure 1? They should be explained in the methods and cited appropriately.
Response 36: Detailed methods and references are added in the revised manuscript.

Table 3: It might be best just to show the column with the combined results and the full table in supplements since you never discuss the other columns in the text. Also, this information is already kind of included as Supplementary table S3, not sure what the differences are between these two tables and if both need to be presented in the manuscript. I recommend consolidating everything into a less redundant format.
Response 37: We have integrated previous Table 3 and Supplementary Table S3 to create a new table (Table 3 in the revised manuscript).

Page 15: This number of predicted genes, number of exons, BUSCO completeness scores, and proportion of repeat genome content is hard to interpret without context of other closely related species. For instance, are these numbers different from what we would expect or are they standard and unremarkable? Without a big supplementary table listing all these stats for all related species it is not possible to really make any conclusions about this genome.
Response 38: We have re-analyzed the assembly quality of genomes of all bivalve species with chromosomal-level genome assemblies using BUSCO 5.4.3 and using the lineage-specific dataset of metazoa_odb10 (Supplementary Table S6 in the revised manuscript).

(1) Also, Supplementary table S4 lists the stats for the "Maker" gene set. Maker was not one of the listed tools used for gene prediction, so I am unsure exactly which gene set was used for this analysis. Also, looking at the data uploaded to the ftp site, only the "Maker" genes were provided. As far as I was aware, the final gene set should have been created using EvidenceModeler not Maker. Additionally, you list the results of functional annotation but don't provide the data anywhere. (2) Please add it to the additional files associated with the manuscript.
Response 39:
(1) We used “MAKER” to merge the annotation results by the homology-based approach, de novo approach, RNA-Seq-based and Iso-Seq-based evidences to create the final gene set. Thus, the gene set “Maker” is the final gene set used for analysis. The sentence has been corrected as “Gene predictions from the homology-based approach, de novo approach, RNA-Seq-based and Iso-Seq-based evidences were merged, and redundancy was removed to form a comprehensive consensus gene set using Maker 2 (RRID:SCR_005309) [52].”.
(2) The results of functional annotation has been added as Supplementary Table S9.

Page 15: What about the BUSCO scores for the predicted genes. How to they compare? You only list the genome stats in the results section but describe running BUSCO on the predicted genes in the methods.
Response 40: We have added BUSCO scores for the predicted genes in the revised manuscript (Table 4).

Page 16: "P. generosa and other 11 species" should read "P. generosa and 11 other species"
Response 41: Corrected.

Page 16: "30,616 gene families were identified as single-copy and multiple-copy genes, unique paralogs, other orthologs, and unclustered genes" This sentence confused me. What are the definitions for each of these categories? They are not listed in the methods, so I am unable to assess exactly what this all means. Also, the exact numbers of each category should be presented so that we know how many of each were produced by this analysis.
Response 42: We showed the details of gene families in various categories for P. generosa and 11 other species in Figure 3 (in the revised manuscript). As per suggestion of the reviewer, we supplemented the table listing the information of all the 30,616 gene families among the 12 species in Supplementary Table S8.

Page 16: You state that "1749 gene families that are specific to P. generosa", however later in the paragraph you say that "2902 gene families that are specific to P. generosa". I am unsure why these number have changed or what the difference is between the two sets. The latter number is the one that is listed in Figure 3.
Response 43: This sentence has been corrected as "As compared with the other 11 species, there were 7917 genes belonging to 1749 gene families that are specific to P. generosa"

Page 16: (1) The number of gene families in Figure 3 that are common to the 4 species is listed as 6490 but in the text is stated as 6940. Is this a typo? (2) Also, are these gene families specific to just this group and not the other species in your analysis? Or can they be shared with the other species as well? This is not fully clear from the text.
Response 44:
(1) The number was a typo and has been corrected (the number is "6490").
(2) These gene families are not specific to just this group. The numbers of gene families in Figure 3 (renamed as Figure 4 in the revised manuscript) were based on statistics of P. generosa and 11 other species (Supplementary Table S8). The numbers in statistic (Figure 4) showed the shared and specific gene family numbers among P. generosa, P. martensi, S. broughtonii, and P. yessoensis.

Page 16: "326 single-copy orthologous gene families from these 12 species showed". You are talking about single-copy orthogroups with genes identified in each species right? Or just a subset? This is not clear from your methods or results sections.
Response 45: The "326 single-copy orthologous gene families" represent single-copy orthologous genes shared by all 12 species.

Page 16: It might be helpful for the reader if you add a shaded box (or another form of annotation) indicating where the bivalve clade is in the tree and how many chromosomes each species has. Since I don't think you explicitly list this for each of your species in the text.？
Response 46: We have added a black bar in the phylogenetic tree to mark the bivalve species (Figure 5 in the revised manuscript).

Page 18: Supplementary Table S6 does not appear to be available with the manuscript so I was unable to assess this section fully.
Response 47: "Supplementary Table S6" was renamed "Supplementary Table S9" in the revised version.

Page 18: "there were a few significant enrichment pathways" I think should read "there were a few significant enriched pathways"
Response 48: Corrected.

Page 18: "revealed 166 pathways" Pathways or genes? I can't assess what is being tested without the supplementary table.
Response 49: The pathway information was included in Supplementary Table S9.

Page 18: "mainly represented in 5 biochemical metabolic pathways of KEGG category…." Do you mean 5 metabolic KEGG categories? As it is written I am not sure exactly what you are trying to say in this sentence. Also, the high level KEGG categories are very general and not particularly informative, maybe just focus on the subcategories.
Response 50: This sentence was removed from the manuscript due to adjustment of the content.

Page 18: "Significantly expanded and contracted genes were found to be related to ovarian …." How can these categories that you list be significantly associated with both expanded and contracted gene families? If you are significantly expanded then they are unlikely to also come up as significantly contracted in your analysis?
Response 51: We agree with the reviewer that this sentence should be corrected as "Significantly expanded genes were found to be related to ovarian …". In the revised manuscript, this sentence was removed due to adjustment of the content.

Page 18: "According to enrichment analyses…." I think it should read "According to the enrichment analyses….". Also, I think you should rephrase this section as it is a little unclear which of the categories that you are listing are from your analysis (if any of them are?) and which are from the literature. Currently, you are mostly just listing categories from the literature and I am not really sure how they relate to your analysis or the conclusions that you are trying to make about this particular species.
Response 52: We have rephased this section as shown in the main text.

Page 19: "Two significantly enriched pathwaFcys…." I think additional letters accidentally got added to the word "pathways".
Response 53: This sentence has been corrected as "Two significantly enriched pathways…".

Page 20: "P. generosa is a highly complex species with a high heterozygosity of 1.37% and 57.99% repeat sequences in genome." I think this should be rephrased to something like "P. generosa is a complex species with a genome that has high heterozygosity (1.37%) and repetitive sequence content (57.99%).". Also, is 1.37% heterozygosity high? Compared to what?
Response 54: The heterozygosity of 1.37% is comparable with that of most bivalves (Supplementary Table S3). We revised the statements in the revised manuscript.

Page 19: "This study may provide a" Rephrase the last part of this section to something like "This study provides a high-quality genomic resource that will support future phylogenetic, evolution, adaptation, and immunological studies. Additionally, it will support the genetic breeding of geoducks.".
Response 55: Corrected.

Page 21: I was unable to find any of the ID that you list in the "Data Availability" section on NCBI. I assume they have not yet been made public?
Response 56: The data were released on April 06, 2023 (Although we submitted in November, 2022 with "Release immediately" option).

Page 22: Some of the abbreviations are formatted weirdly. For example, RAxML doesn't have a definition. Neither does Iso-seq. Please double check this section.
Response 57: We have revised the abbreviations in the revised version of the list.

Figure 5: What do the colors in the pie charts represent? The "combined change across lineages" pie chart is hard to interpret as there is no context for the colors or number given. Please add more detail into the figures and figure legends to help the reader interpret the data.
Response 58: The green parts represent gene gains, the red parts represent gene losses and the blue parts represent gene remained. The "combined change across lineages" pie chart is no use, which has been removed from this Figure (as Figure 6 in the revised version). The figure legend has been supplemented with detailed information.

Reviewer #2:
The manuscript reports a chromosome-level genome of the Pacific geoduck Panopea generosa using Pacbio and Hi-C technologies. As an important species in both agriculture and ecology, the Panopea generosa genome is a valuable reference data for the study of clams. The quality of the assemblies seems not bad for the mollusk species. The current genome manuscript is publishable when a substantial revision is made.

1. The final genome should be described more clearly. It is necessary to display the statistic information of each chromosomes' assemblies, such as length and number of unclosed gaps and contigs.
Response 59: We added chromosome information in Table 2 in the revised manuscript. We also showed statistic information of each chromosomes' assemblies in Supplementary Table S4.

2. Distribution of 17-mer occurrence reflects a high heterozygosity (double-peak distribution) in the genome, which are not suitable for estimation of its genome size (k-mer analysis suitable for the single peak k-mer distribution). Do the authors have any experimental evidence (e.g. flow cytometry analysis) to define the genome size? The author should give more reliable information for it.
Response 60: We did not carry out cytometry analysis in this project. The k-mer analysis was done in this

project for estimating the genome size, which was used to determining PacBio sequencing quantity for genome assembly.

3. The authors said that the sequenced individual was collected in Strait of Georgia. How do the authors identify it as the Pacific geoduck P. generosa, mitochondrial sequencing or morphological characterization? It should be clarified.
Response 61: The sample was identified both morphologically and molecularly. The sample showed typical morphological features of P. generosa. This identification was further confirmed by the high percentage identity of the molecular marker cox1 (99.55%) over the entire sequence (coverage = 100%). Details are added in the revised manuscript.

4. The phylogenetic analysis includes human, zebrafish and toad orthologs. The relationship between each of the three vertebrates and P. generosa are so remote that similarity among the homologs are low, which inevitably causes more errors in gene clustering. Besides, the authors use three time points to calibrate the speciation, C. elegans and H. sapiens (678.3-855.2 MYA), D. rerio and H. sapiens (413.1-443.0 MYA) and X. tropicalis and H. sapiens (347.0-357.9 MYA, all of which have the great evolution distance. These time points are not appropriate for the calculation of divergence time among clams. Also, the constructed phylogenetic tree does not have an outgroup. In short, the authors should redo the gene clustering with OrthoMCL by selecting most of the invertebrate species and use the time point among clams or mollusks in fossil calibration. An outgroup should be assigned in the constructed phylogenetic trees.
Response 62: The purpose of this phylogenetic analysis was to illustrate the evolutionary position of P. generosa in metazoan. For the high quality of the phylogenetic tree, we were selective about the genomes used for this analysis. Four model organisms were chosen because they are best studied organisms with high-quality genome assemblies and genome annotations, and they represent good indicators of evolutionary positions. Also, only bivalves with high quality genome assemblies were chosen in this analysis. With the increase of high-quality genome assemblies, more genome will be included in future analysis.

5 The authors carry out the enrichment analysis of the expanded gene families. Actually, it is a tedious work, which do not make sense. Any survey genomes can do that. The chromosome-lever genome is supposed to exhibit more chromosome structure, such as gene collinearity or chromosome synteny among different related species, which is able to reflect not only assembly quality but also data importance. Most of the genome sequencing and assembling are completed by a commercial sequencing company, which means that the manuscript should include more analyzed information by the authors themselves. Referencing the recently-published papers in Gigascience, the manuscript should normally make one or more tries of evolutionary analyzation or gene expression profiling, not just the description of the assembly itself. Accordingly, the authors, on the one hand, should improve this description by providing a couple of figures to illustrate the annotation of the expanded gene families; one the other hand, construct the gene/chromosome synteny among 3 or more related species (illustrated as the circus image) to check the chromosome arrangement of evolutionary analysis.
Response 63: We appreciate the suggestions. We have carried out additional synteny analysis and gene family analysis. In particular, through comparative analysis of the chromosome-level genome assembly of P. generosa and its closely related species S. constricta, we uncovered major inter-chromosome changes. We also observed substantial intra-chromosomal changes. A new figure (Figure 2) is included in the revised manuscript to describe these new results. We have also included the identification of expanded gene families with functional domains in P. generosa. In particular, the copine gene family showed substantial expansion in P. generosa, with twice as many genes in this gene family compared to its closely related species. A new figure (Figure 7) is included in the revised manuscript.

6. In main text, the BUSCO test indicates the genome completeness of 90.9%. However, in the supplementary files, the Hi-C BUSCO and annotation BUSCO test show the completeness at 93% and 88%, respectively. The authors should clarify it and provide the BUSCO test table in the manuscript.
Response 64: As the genome as evaluated multiple times using different BUSCO version, results were inconsistent in the original submission. BUSCO results have been revised in the revised manuscript.

7. The English writing of the manuscript should be improved when a revision is submitted.
Response 65: We have done extensive revision of the writing of the manuscript.
