# Supplementary material for: Chromosome-level genome assembly of the Pacific geoduck Panopea generosa reveals major inter- and intrachromosomal rearrangements and substantial expansion of the copine gene family
Source: Gigascience. 2023 Dec 19;12:giad105. doi: 10.1093/gigascience/giad105 (PMC10729735; doi:10.1093/gigascience/giad105)
Supplement: giad105_Response_to_Reviewer_Comments_Revision_1 [file giad105_response_to_reviewer_comments_revision_1.pdf]

Author's Response To Reviewer Comments

Manuscript number: GIGA-D-22-00284R1
Title: “Chromosome-level genome assembly of the Pacific geoduck Panopea generosa reveals major inter- and intra-chromosomal rearrangements and substantial expansion of the copine gene family”

Responses to reviewers:

Reviewer #1: The authors have made a good attempt as address the comments on the previous version of the manuscript. While this version is a significant improvement, I still feel that there are outstanding issues that need to be addressed before it can be accepted for publication. Generally, the phrasing of the manuscript has been greatly improved, however, additional work is still required (some of which I have tried to detail in my specific comments below). Additionally, the authors have included some interesting now analysis in this version of the manuscript, however, these results are occasionally not well integrated into the text of the manuscript (some of the results in the results section could be reordered to improve the flow of the manuscript), do not always have a clear purpose (such as the Alphafold2 results), or are not described in the methods. I would suggest that the authors carefully review the new analysis to make sure it all fits within the manuscript. I have tried to give specific comments addressing each of these issues below.
Response 1: Thanks for the comments and suggestions. We have re-organized the content, and have expanded the method section.

It would be nice if in the next round the authors added line numbers to the manuscript so that it is easier to call out parts of the manuscript.
Response 2: Added.

Page 3: "revealed major inter- and intra-chromosomal exchanges" - Do you mean "rearrangements" not "exchanges". "Exchanges" makes it sounds like the two species are swapping DNA (HGT), which I assume you don't mean.
Response 3: We meant “rearrangement”. Thanks for your correction and we have revised.

Page 3: "neuronal development, immune response" should probably read "neuronal development and immune response". The comma is a little confusing.
Response 4: Corrected.

Page 5: "(which can be as long as 168 years) for P. generosa [4]"
I assume it should read "(which can be as long as 168 years for P. generosa [4])"
Response 5: Corrected.

Page 6: "recombination in evolution in bivalves" should probably read "recombination during the evolution of bivalves"
Response 6: Corrected.

Page 6: "Nevertheless, high-quality chromosome-level" should probably read "Nevertheless, a high-quality chromosome-level"
Response 7: Corrected.

Page 7: "The availability of the genome information" should probably read "The availability of this genome information"
Response 8: Corrected.

Page 7: I am not sure how the results in Table 1 support the sentence "The genome size of P. generosa was estimated to be 1.47 Gb using k-mer analysis (Table 1) [22]."
Nothing in that table describes genome size or any genome-related statistics.
Response 9: We thank the review for pointing out this inconsistency. We have rewritten the paragraph.

Page 7: I still feel that the method that the authors used to estimate genome size is not adequately described. In their response to my comment on the previous version of the manuscript the authors mentioned that Jellyfish was used for k-mer enumeration, however, once you have decomposed the data into k-mers you still need to use additional tools (such as GenomeScope) or formula (k-mer count / homozygous peak coverage) to produce the final genome size/heterozygosity/repeat content estimations that the authors cite.

Additionally, the authors quote heterozygosity and repeat sequence proportion values but don't indicate how they were estimated from the enumerated k-mers (i.e., using which tool or formula). More detail about k-mer analysis needs to be provided in the methods.
Response 10: We are sorry for not providing adequate details. The genome size, heterozygosity, and repeat content were estimated using GCE 1.0.2. Relevant details are incorporated in the revised manuscript.

Page 7: (1) "The heterozygosity of P. generosa was comparable to those of most bivalves (Supplementary Table S3)." You compared the value from your genome to those of published genomes and came to what conclusion? This sentence doesn't really add anything to the results since there is no take away from it. Please elaborate on how your heterozygosity compares to that of the published genomes.
(2) Also, it would be best to add reference to the genomes in Table S3, to acknowledge the authors whose data you are citing.
Response 11: (1) This part is now rephrased in the revised manuscript. (2) References and NCBI accession numbers have been added.

Page 7: I might suggest that "Genome assembly using PacBio long reads (N50 = 26,513 bp) and Falcon assembler obtained an initial size of 1.51 Gb. Further assembly using Hi-C data obtained a genome with 19 pseudomolecules, suggesting 19 chromosomes of the P. generosa genome (Figure 1A), with an anchoring rate of 94.70%. This genome assembly has a total length of 1,474,161,289 bp with a contig N50 of 1.57 Mb and a scaffold N50 of 73.79 Mb (Figure 1B; Table 2; Supplementary Table S4). As expected, the genomic regions with low gene density typically had high repeat content, while the regions with high repeat content usually had high GC content."
Should read something like:
"The genome assembly produced using PacBio long reads (N50 = 26,513 bp) and the Falcon assembler had an initial size of 1.51 Gb. Further refinement using Hi-C data (anchoring rate of 94.70%) produced an assembly with 19 pseudomolecules, putatively the 19 chromosomes predicted in P. generosa (Figure 1A). This genome assembly has a total length of 1,474,161,289 bp with a contig N50 of 1.57 Mb and a scaffold N50 of 73.79 Mb (Figure 1B; Table 2; Supplementary Table S4). As expected, the genomic regions with low gene density typically had high repeat content, while the regions with high repeat content typically had high GC content."
Response 12: This part is now rephrased in the revised manuscript.

Page 7: Great work with Table S1. A very comprehensive picture of the available genomes. Why do some of the genomes not have references? Are they unpublished? If so, then you should state the location where you downloaded/accessed the data.
Response 13: Corrected.

Page 8: "The majority (57.99%) of the P. generosa genome was repetitive elements estimated using de novo searching and homolog prediction (Table 3). Distribution of these repetitive elements was uneven with repetitive content per 1 Mb varied from 34.76% to 84.89% (Figure 1B)."
Should probably read:
"The majority (57.99%) of the P. generosa genome was estimated, using de novo searching and homolog prediction, to be repetitive elements (Table 3). Distribution of these repetitive elements was uneven across the genome, with repetitive content per 1 Mb varying from 34.76% to 84.89% (Figure 1B)."
Response 14: The two sentences have been modified, and the second sentence has been moved to the third paragraph in the part “Genome annotation and evaluation”.

Table 3: It's a little bit repetitive having the "Reparative sequence" and "Transposable elements" columns

with almost all the same rows. Potentially just show the "Repetitive sequence" column.
Also, the "Total" values do not add up correctly (the Total "% in genome" for the "Reparative sequence" column should be 70.19% by my calculations, not 57.99%). Please double check all values.
Response 15: The structure of Table 3 was reformatted to avoid the repetitive content. In addition, we checked all the values for correctness. In fact, the total values were not simply the sum of all the values in the columns because of potential overlaps among different types of repetitive sequences.

Page 8: "Of these PCGs, 30,700 genes were annotated to contain conserved functional motifs (Supplementary Table S5, Table S7)." Should probably read "Of these PCGs, 30,700 were annotated with conserved functional motifs (Supplementary Table S5, Table S7)."
Response 16: Corrected.

Page 8: "and annotated protein-coding gene set were". Just the annotated protein-coding genes? Or all protein-coding genes?
Also, can you use the "PCG" acronym here?
Response 17: We meant "all protein-coding genes".

Page 8: "To evaluate the completeness of the assembly, the P. generosa genome assembly and annotated protein-coding gene set were assessed using BUSCO [23] with the metazoa_odb10 database (954 core genes), respectively."
This sentence is a little confusing. What is the "respectively" referring to since there are not to elements at the start and end of the sentence that you are associating together. Please rephrase.
Response 18: This sentence has been rephrased.

Page 8: "Regarding the gene set,"
Can these genes be referred to as "PCGs"?
Response 19: Yes. This sentence has been rephrased.

Page 8: "Although the P. generosa genome has 19 chromosomes as many other species in the order Adapedota" should probably read "Whereas the P. generosa genome has 19 chromosomes, the same number as in other species in the order Adapedota"
Response 20: Thanks for your correction. This sentence has been removed in the revised manuscript.

Page 9: "whose genome has been assembled at the chromosome-level revealed that 8
these two genomes have good chromosomal collinearity in general" should probably read "whose genome has been assembled at the chromosome-level, revealed that in general these two genomes have good chromosomal collinearity"
Response 21: This sentence has been rephrased.

Page 9: "clear one-to-one r correspondences S. constricta chromosomes" should probably read "clear one-to-one r correspondence with S. constricta chromosomes"
Also, what is "r correspondence"? I am unfamiliar with that term.
Response 22: The word "r" was an error, and was removed. This sentence has been removed in the rephrased paragraph.

Page 9: Would the term "rearrangements" be more appropriate than "exchanges" to use throughout the manuscript?
Response 23: Yes, the words "exchanges" have been corrected into "rearrangements".

Page 9: "also revealed that extensive intra-chromosomal" should probably be "also revealed extensive intra-chromosomal"
Response 24: Corrected.

Page 9: Sorry, I still find the description of the shared and specific gene families confusing. You state that there are two different groups of gene families that are specific to P. generosa. I assume the 2902 set is considering just the three other species shown in Figure 4?
Also, why were these three other species chosen for analysis and visualization in Figure 4?

Why focus on just these species in Figure 4? You use all species for the orthogroup analysis, what benefit does focusing on just these four add to the analysis/manuscript?
Response 25: The 2902 set was specific to P. generosa, which were not in the three other species in the old Figure 4 (Figure 5 as a new version). The three other species P. martensi, P. yessoensis, and S. broughtonii were chosen for comparison, considering that they were from different taxonomic group and possessing different phylogenetic relationship in the phylogenetic tree.

Page 10: "identified in P. generosa genome" should probably read "identified in the P. generosa genome"
Response 26: Corrected.

Page 10: "166 pathways from the expanded gene" Some of those pathways at the end of your list have large p- and q-values. I would suggest filtering the data using a reasonable cutoff.
Response 27: We filtered the data by "q-value≤0.05", and then expanded genes enriched significantly in 123 pathways.

Page 10: "(Supplementary Table S9) The" should probably read "(Supplementary Table S9). The"
Response 28: Thanks for your correction. This part has been removed in the rephrased paragraph.

Page 10: "Fluid shear stress and atherosclerosis, Phosphatidylinositol signaling system, suggesting their important contribution to the adaptation of benthic bivalves."
This part of the sentence is a little confusing. You are listing a lot of terms here and the sentence doesn't flow well as a result. Consider rephrasing.
Also, are you missing an "and" at the end of the list?
Response 29: The "and" has been added, and this sentence has been rephrased.

Page 10: "which were found related to" should probably read "which have been shown to function as part of"
Response 30: Corrected.

Page 11: "Examination of the top 65 domains of gene families that presented expansions in P. generosa (Figure 7A) showed that the gene numbers of many important gene families were substantially expanded in P. generosa, including these containing the GIY-YIG catalytic domain (PF01541), the caspase recruitment domain (PF16739), the ApoA/ApoE domain (PF01442), and the copine domain (PF07002)."
This sentence is confusing. You are looking at the expanded gene families, of course they are"substantially expanded in P. generosa", that is why you are looking at them right? Please rephrase.
Also, are the domains that you list the top most abundant or just the ones that you found most interesting? This is unclear.
Response 31: The domains listed in Figue7A were the 65 most abundant gene families that P. generosa possessing more gene numbers than 8 other bivalves, not just the ones that we found most interesting.

Page 11: "In particular, the copine gene family,…."
This paragraph opens with a statement about InterProScan, but then only talks about Pfam domains and gene copy expansion. This whole section could be reordered so that your results about gene family expansion are together (up with the paragraph starting "A total of 507 expanded gene families"), and the functional results (Pfam + KEGG) are together.
Response 32: The evolution of gene families was analyzed using two different but complementary approaches. The analysis using CAFÉ identifies the expansion and contraction of gene families, whose impact are further evaluated by functional analysis using GO and KEGG analysis. The analysis using InterProScan focused on genes whose protein sequences harbor particular functional domains (i.e., Pfam domains).

Page 11: "Interestingly, many genes formed local clusters"
This is very interesting. Do you see a single copy of these genes in roughly the same chromosomes in S. constricta? That is, does S. constricta have the "original" gene from each of the local clusters in its chromosomes or have these genes also spread throughout the P. generosa genome and then expanded locally.
Also, is this a common mechanism for gene family expansion? i.e., do the other genes that show expansion

in this species also have identifiable local clusters?
Response 33: There were 11 copine genes distributed in 8 chromosomes of S. constricta genome. In general, there were 1-2 copine genes in the same chromosome S. constricta genome. This is a common mechanism for gene family expansion, such as IAP genes in Bathymodiolus platifrons (Sun et al, 2017).

Sun et al. Adaptation to deep-sea chemosynthetic environments as revealed by mussel genomes. 2017. Nat. Ecol. Evol. 1, 0121.

Figure 7: Double check the figure legend. I think the captions for B and C are switched?
In the text you describe these genes as the "copine genes", but in the figure legend you say that they are "genes annotated to PF07002 domains". Are these the same or are these different trees? This need to be clarified/consistent terminology used.
What are the support values for each of the nodes in the tree? Do the genes group based on their location across the chromosome? I would assume so since they are local duplications but it might be good to annotate or indicate where in the tree the genes from these local clusters fall.
Response 34: The support values for nodes were shown in the revised tree, when the support value was above 60. The genes from the same chromosome were clustered together, such as copine genes from chromosome 11, or copine genes from chromosome 10 and 7.

Page 12: "as for copine genes identified in other species"
As for what? This sentence is phrased like you are going to say something else but don't. Do you mean "like for copine genes identified in other species"?
Response 35: Yes, we do mean "like copine genes identified in other species".

Page 12: "imperfect annotation" Do you mean "imperfect prediction"?
"Annotation" usually means "functional annotation". If you are talking about gene structure issues you are usually talking about "gene prediction".
Response 36: Yes, "imperfect prediction" is correct.

Page 12: "Through the construction of the first high-quality chromosome-level genome assembly of the ecologically and economically important bivalves the Pacific geoduck P. generosa with cutting-edge genomic technologies, important insights into its genetic makeup and evolution have been gained.
Should probably read:
"The construction of the first high-quality chromosome-level genome assembly using cutting-edge technologies of the ecologically and economically important bivalve, the Pacific geoduck P. generosa, provides important insights into its genetic makeup and evolution.
Response 37: Corrected. This sentence has been rephrased.

Page 12: "The assembled genome size consists of 19 chromosomes with a genome size of 1.47 Gb, and a contig N50 of 1.6 Mb and 19 chromosomes."
Should probably read:
"The assembled genome consists of 19 chromosomes, has a size of 1.47 Gb, and a contig N50 of 1.6 Mb."
Response 38: Corrected.

Page 12: "and the conservation of PCGs in bivalves."
Does this show the conservation of PCGs in bivalves? I would think only your orthogroup analysis would do that, not the gene prediction/annotation workflow.
Response 39: This sentence has been removed in the revised manuscript.

Pahe 12-13: "P. generosa is a highly complex species with a heterozygosity of 1.37% and 57.99% repeat sequences in genome."
Does this make it complex? Compared to what? It seems pretty standard compared to other bivalves. Please justify or remove.
Response 40: This sentence has been removed in the revised manuscript.

Page 13: "Large chromosomal fragments of P. generosa Pg02 and Pg11 matched to two S. constricta chromosomes (Chr1 and Chr10) and two S. constricta chromosomes (Chr1 and Chr10), respectively

(Figure 2)."
This sentence repeats the phrase "two S. constricta chromosomes (Chr1 and Chr10)", please rephrase.
Response 41: Corrected.

Page 13 and 14: It is unclear how important it is that the copine genes contain C2 and vWA-domains. Slightly more context for this statement is required to help the reader understand why this is interesting. Also, what is the significance of the Alphafold2 results? It is unsurprising that homologous genes would have highly similar structures. Unless there is something else that these results add to the manuscript I would suggest removing the Alphafold2 results and discussion. Additionally, the Alphafold2 work is not described in the methods.
Response 42: Because the copine genes expanded in P. generosa, it is important to learn the information of their distribution and structure.

Page 17: "The Hi-C heatmap was visualized using Juicebox (RRID:SCR_021172) presenting the counts of paired reads which each two bins aligned (with the bin length of 100 kb) as the interactive signals between each pair of two bins."
This sentence is a little confusing. Please consider rephrasing to improve readability.
Response 43: Corrected.

Page 17: "version5.4.3) [23]. using the"
Remove the full stop.
Response 44: Corrected.

Page 18: "Exonerate v2.2.0"
Can Exonerate take hits and reconstruct genes? My understanding is that Exonerate (by default) does the alignment for you and builds the gene from that. If there is a specific functionality of Exonerate that take pre-computed hits then this needs to be detailed.
Response 45: We did not mean that Exonerate reconstruct genes. In fact BLASTN was used to find hits from the P. generosa genome, which were aligned with gene sets from eight closely related bivalves (Patinopecten yessoensis, Pinctada fucata, Mytilus galloprovincialis, Limnoperna fortune, Argopecten purpuratus, Sinonovacula constricta, Scapharca broughtonii, and Crassostrea gigas). Exonerate was used to predict the structures (such as exon and intron) of each blast hits.

Page 18: I assume you used pre-trained models for Augustus and SNAP de novo prediction? If so, which models?
Response 46: Models used for each gene predictor Augustus and Snap traning were obtained from a set of high-quality proteins generated from the RNA-Seq and Iso-Seq dataset by MAKER 2. This detail was added in the revised manuscript.

Page 19: "procedures described previous studies" should probably read "procedures described in previous studies"
Response 47: Corrected.

Page 20: I see no new methods describing the InterProScan, Alphafold2, phylogenetic analysis (Figure 7C), or synteny/chromosome reorganization analysis.
The methods for these new analyses need to be extensively described.
Response 48: Corrected.

Figures: Please make sure that you adequately describe the figure in each figure legend. For example, the figure legend for Figure 2 does not describe each panel (A and B), the panels in Figure 7 maybe mislabeled, etc.
Response 49: Corrected.

Reviewer #2: The authors improve the manuscript somewhat by adding more analyses of rapidly expended gene clusters and a couple of potential functional genes, as well as the chromosome synteny between two related species. As far as the whole manuscript is concerned, most of the studies in genomics or evolution

analysis are so superficial that it cannot make much sense sometimes, except a chromosome-level genome assembly. In view of the data value of the genome assemblies, I think this manuscript is able to be published as a DATANOTE paper when the second round revision is done. The authors should revise the manuscript according to all the listed corrections and comments (D1—D18) in the attached file. The final manuscript should also match the format of the DATANOTE paper. As to the terrible English writing (no improvement at all), I have tried to edit the abstract and result sections as possible. The discussion section should be re-written.

Response 50: Thanks for the comments and correction. We have checked all the problems in the revised manuscript.
