# Supplementary material for: Chromosome-level genome assembly of the Pacific geoduck Panopea generosa reveals major inter- and intrachromosomal rearrangements and substantial expansion of the copine gene family
Source: Gigascience. 2023 Dec 19;12:giad105. doi: 10.1093/gigascience/giad105 (PMC10729735; doi:10.1093/gigascience/giad105)
Supplement: giad105_Response_to_Reviewer_Comments_Revision_2 [file giad105_response_to_reviewer_comments_revision_2.pdf]

# Author's Response To Reviewer Comments

Manuscript number: GIGA-D-22-00284R2
Title: "Chromosome-level genome assembly of the Pacific geoduck Panopea generosa reveals major inter- and intra-chromosomal rearrangements and substantial expansion of the copine gene family"

Responses to reviewers:
Reviewer #1: The authors have done a good job at address my comment on the previous version of their manuscript. While I am generally happy with the state of the manuscript, I still have some minor grammatical issues that can be easily addressed by the authors. Additionally, I agree with Reviewer 2 that the Discussion section is very repetitive of the Results section and adds very little new insight to the manuscript. I would suggest, if possible, that the Discussion be rephrased to include a discussion of the significance of the results, or that it be reduced to reduce repetition.
Response: Thanks for the comments and suggestions.

L72-73: "These genomes can provide a resource for comparative genomic for gaining evolutionary and other insights of bivalves and even molluscs" TO "These genomes can provide a resource for comparative genomics, and for gaining evolutionary and other insights into bivalves and molluscs"
Response: Corrected.

L95: "gene family expansion and contraction" TO "gene family expansion and contraction analysis"
Response: Corrected.

L128-131: "Of these PCGs, 30,700 (87.6%) were annotated with according to the public database including Nr, Swissprot, KEGG, KOG, TrEMBL, Interpro, and GO (Supplementary Table S5), suggesting the high quality of genome annotation." TO "Of these PCGs, 30,700 (87.6%) had functional annotations assigned using comparisons to public databases, including NCBIs nr, Swiss-Prot, KEGG, KOG, TrEMBL, InterPro, and GO (Supplementary Table S5), supporting the high quality of the genome."
Response: Corrected.

L133: "elements varied from" TO "elements varying from"
Response: Corrected.

L137: "93.0% of core PCGs were" TO "93.0% of the core genes used by BUSCO were"
Response: Corrected.

L138: ", higher than that" TO ", which is higher than that"
Response: Corrected.

L139: "of annotated PCGs using BUSCO, which were aligned to 88.4% of the full-length core orthologs, suggesting that quality of the annotation can be further improved in the future." TO "of the predicted PCGs using BUSCO, which identified 88.4% of the core genes as full-length, suggesting that quality of the predicted genes can be further improved in the future."
Response: Corrected.

L150: "chromosomes of these two species were both 19" TO "chromosomes in these two species is still 19"
Response: Corrected.

L154-155: "two species P. generosa and S. constricta," TO "two species (P. generosa and S. constricta),"
Response: Corrected.

L159: "orthologous genes of the 12" TO "orthologous genes from 12"
Response: Corrected.

L219-221: "while some has both C2-domain and the vWA-domain in P. generosa (Figure 7D) like copine

genes identified in other species [33]." TO "however some have both a C2-domain and a vWA-domain in P. generosa (Figure 7D), like copine genes identified in other species [33]."
Response: Corrected.

L222: "due to errors of genome assembly or gene prediction" TO "due to genome assembly or gene prediction errors"
Response: Corrected.

L230: "important bivalves the Pacific geoduck P. generosa," TO "important bivalve, the Pacific geoduck P. generosa,"
Response: Corrected.

L235: "instance of chromosome-level genomes of the bivalve species" TO "instance of a chromosome-level genome assembly from a bivalve species"
Response: Corrected.

L259: "that the numbers of PCGs of many gene families" TO "that the number of PCGs in many gene families"
Response: Corrected.

L260: "substantially in evolution" TO "substantially during the evolution of this group"
Response: Corrected.

L262: "genes were identified" TO "genes identified"
Response: Corrected.

L265-267: "composition of functional domains, which include C2 domain and vWA-domain (Figure 7D), and the similarity of three-dimensional structure to that of known copine genes (Figure 7E) suggests functional conservation" TO "composition of functional domains, which includes the C2 and vWA domains (Figure 7D), and the similarity of three-dimensional structure to that of known copine genes (Figure 7E), suggests conservation"
Response: Corrected.

L268: "completion of P. generosa facilitates genome comparative analysis" TO "completion of the genome P. generosa facilitates comparative analysis"
Response: Corrected.

L271: "construction of P. generosa chromosome-level genome" TO "construction of a chromosome-level genome assembly of P. generosa"
Response: Corrected.

L272: "in this organism, but also" TO "in this organism, but will also"
Response: Corrected.

I agree with Reviewer 2 that the discussion is very repetitive of the Results section and does not add much insight beyond what has already been said. If possible, I would advise the Authors to revise this section so that it provides a higher-level overview of the significance of their results.
Response: We have reduced repetitive statements in the discussion section.

L281: "samples- were transferred to laboratory" TO "samples were transferred to the laboratory"
Response: Corrected.

L365: "PCGs in P. generosa" TO "PCGs in the P. generosa"
Response: Corrected.

L424: "family-wide P-Values were" TO "family-wide p-values were"
Response: Corrected.
