# Supplementary material for: Chromosome-level genome assembly of the Pacific geoduck Panopea generosa reveals major inter- and intrachromosomal rearrangements and substantial expansion of the copine gene family
Source: Gigascience. 2023 Dec 19;12:giad105. doi: 10.1093/gigascience/giad105 (PMC10729735; doi:10.1093/gigascience/giad105)
Supplement: giad105_Reviewer_1_Report_Original_Submission — Timothy Gordon Stephens, Ph.D. -- 11/24/2022 Reviewed [file giad105_reviewer_1_report_original_submission.pdf]

Reviewer Report

Title: Chromosome-level genome assembly of the Pacific geoduck Panopea generosa reveals major inter- and intra-chromosomal rearrangements and substantial expansion of the copine gene family

Version: Original Submission **Date:** 11/24/2022

Reviewer name: Timothy Gordon Stephens, Ph.D.

Reviewer Comments to Author:

The manuscript by Wang et al. reports the high-quality chromosome-level assembly of the economically important Pacific geoduck Panopea generosa. While I believe that this work will be of significant value to the scientific community, the manuscript requires significant rephrasing and additional details added before it can be published. Specifically, a lot of additional details need to be provided in the methods section, including which tools were used, their versions and parameters, as well as clarification of the points that I detail below. Rephrasing and additional details are also required in the results section to improve understanding by the reader. I also could not find Supplementary Tables S2 and S6 online. If I have missed them then I apologies, however, if they we not uploaded then this needs to be corrected for the next round of reviewers. Please see my specific comments below.Page 5: "They are preys for" should read "They are prey for"Page 5: "shell concentrations" of what? Did you mean "shell composition"? Also, I am unsure what you mean by "sequence of events related to storm events". What specifically are these events that are related to storms?Page 6: "gene family expansion and contraction" should probably read "gene family expansion and contraction analysis"Page 6: "Genomic DNA of P. generosa was extracted using a standard phenol-chloroform extraction method" I understand that this process is commonly used for DNA extraction however additional detail about your exact application of the method or at minimum references to the protocol that you followed is required to assist the reader.Page 7: "Sequence libraries with insert size of 300 bp were constructed" by who? A service provider? Using which protocol? This needs to be listed.Page 7: "genome size estimation, k-mer analysis", weren't k-mers used to estimate genome size? This sentence makes it sound like they are two separate processes.Page 7: "Hi-C library with insert size of 300 bp was constructed". Who constructed the library, using what protocol, and which company actually did the sequencing?Page 7: "The raw sequence data generated .....". List programs, versions, and parameters used to filter the BGIseq and PacBio reads.Page 7: "Each RNA sample was qualified and". I don't think that this is the correct use of the word "qualified". Maybe try "The quality and quantity of RNA in each sample was assessed using a NanoDrop …."Page 7: "For RNA-seq, the mRNA library was constructed by purification, fragmentation, a first-strand cDNA generation, a second-strand cDNA synthesis, and RNA index addition." List kits and protocols used for this analysis.Page 8: "Counting of k-mers was conducted using Jellyfish". Which other tool or method was used to estimate genome size after k-mers were produced by jellyfish? Based on Figure S1 I would guess that you used your own workflow for genome size estimation. A description and references for this approach is required. Also, the "k" in k-mers should be italicized through the manuscript.Page 8: "Short paired-end clean reads from BGIseq-500 were then polished using Pilon" The

short reads were used by Pilon to correct the assembly? This sentence makes it seem like Pilon was used to correct the short reads, which I don't think is correct.Page 8: "Chromosomes were further corrected using Juicebox". More detail is required here since I assume you did some manual corrections using Juicebox. If you just used an automated correction workflow then these details need to be given.Page 8: "The completeness of genome assembly was assessed using BUSCO". Using which lineage dataset?Page 8: "In homologous prediction, RepeatMasker (RepeatMasker, RRID:SCR_012954) and the associated RepeatProteinMask [18] were performed by alignment against Repbase database". This sentence is a little hard to follow. I assume that you mean the repeats from Repbase were aligned against the reference genome using the mentioned programs? Based on how it is currently phrased, it is unclear what is being aligned against Repbase. Also please provide details on any parameters given to each program when they are run. The following sentence can also be restructured to make it clear that repeats were identified de novo in the genome assembly and what the parameters used for each of the tools were.Page 8: "Besides, tandem repeats". Remove the work "Besides" from this sentence.Page 9: "The results were then integrated and redundancy was eliminated." How was redundancy eliminated? Using which tool?Page 9: "from different branches were chosen to confirm the completeness of the gene set." I am unsure what you mean by this sentence. Do you mean one genome per taxonomic group was selected? What is mean by "to conform the completeness"?Page 9: "hits were then conjoined by Solar software (SOLAR, RRID:SCR_000850) [25]." I don't think that this software is a gene prediction tool. From what I can tell it is a QLT analysis tool. Is this correct?Page 9: "For de novo annotation, six programs were simultaneously used.....". Version numbers and parameters are required for each of these tools.Page 9: "Among them, Augustus, snap and GlimmerHMM were trained using PASA-T-set gene models." What is the "PASA-T-set"? This set of (I assume) RNA-seq based gene modes is never defined in the text.Page 10: "Gene predictions from the homology-based approach, ..." List version, parameters, and evidence weights used for EvidenceModeler. Also, list lineage dataset used for BUSCO analysis.Page 10: "Gene families were constructed according to OrthoMCL (OrthoMCL DB: Ortholog Groups of Protein Sequences, RRID:SCR_007839) pipeline" should probably read "Gene families were constructed using the OrthoMCL (OrthoMCL DB: Ortholog Groups of Protein Sequences, RRID:SCR_007839) pipeline"Page 10: "... Caenorhabditis elegans) were filtered. When multiple ..." The transition between these two sentences is a little hard to follow. When I first read it, I didn't immediately understand that the following sentence lists the filtering steps. Please rephrase this section.Page 10: "(E value = 1e-5 by default) [24]" what does the "by default" mean here? If you are setting the e-value to be 1e-5 then you are not running the program using default parameters?Page 10-11: "SCR_007839) 2.0 with the expansion coefficient 1.5 [38]. Finally, to perform multiple sequence alignment for single copy genes clustered, and concatenate sequence alignment of single-copy gene families. Based on the clustering results, the division of gene family was obtained. To construct phylogenetic trees, protein sequences of each single copy gene of P. generosa and other 11 species were aligned using MUSCLE (MUSCLE, RRID:SCR_011812) [39], and then the protein alignment results were converted into CDS alignment results."I am slightly confused by this section. You selected gene clusters that were single copy in how many species? What does "Based on the clustering results, the division of gene family was obtained" Mean? Do you mean that each cluster was considered a gene family? How were the protein alignment converted to CDS alignment? Using which tool? Please rephrase this section to make your approach clearer.Page 11: "Next, conserved sites were extracted ....."

So you only used conserved sites for phylogenetic analysis? Conserved in how many species? All of them? Were these used for phylogenetic analysis? I assume not since if they are conserved (i.e., no variation between species) then they wouldn't tell you anything about how the species are related.Page 11: "The phylogenetic tree was constructed …." So, a single model was selected to represent the whole super alignment? Since each single copy gene family used to create the super alignment would likely have a slightly different optimal model, does it make sense to pick just a single model for all of them? Why not perform a partition aware phylogenetic analysis (like what is implemented in the IQtree program)?Page 11: "The clustering results of gene families …" Were the gene families filtered in any way before being used for this analysis? You also use the term "orthologous gene families", which you hadn't before in the text, it would help the reader if you picked a single way of referring to the clusters and used it throughout the text.Page 11: "A probabilistic graphical model …." Implemented in which program? Same with the following sentence, which tool calculated the p-values?Page 12: "Genome survey analysis using …" Do you mean "genome size estimation using k-mers"?Page 12: "included 39 scaffolds anchored to 19 chromosomes with an anchoring rate of 94.70%." I do not quite understand what you mean by this statement. So, there are 39 scaffolds that comprised 19 chromosomes? Or 19 of the scaffolds are the main chromosomes and 20 are unplaced scaffolds? Also, I have trouble believing that you only got 39 scaffolds from the assembly workflow. You will always get a lot of small scaffolds; I assume that these were removed at some stage, but I see no discussion of this in the methods. This need to be added so that people know how you generated an assembly with so few scaffolds.Page 14: I could not find Supplementary Table S2 available anywhere, so I was unable to assess this section.Page 14: "and Mytilus edulis have14 chromosomes" add a space between "have 14"Page 14: Which tools were used to create the images in Figure 1? They should be explained in the methods and cited appropriately.Table 3: It might be best just to show the column with the combined results and the full table in supplements since you never discuss the other columns in the text. Also, this information is already kind of included as Supplementary table S3, not sure what the differences are between these two tables and if both need to be presented in the manuscript. I recommend consolidating everything into a less redundant format.Page 15: This number of predicted genes, number of exons, BUSCO completeness scores, and proportion of repeat genome content is hard to interpret without context of other closely related species. For instance, are these numbers different from what we would expect or are they standard and unremarkable? Without a big supplementary table listing all these stats for all related species it is not possible to really make any conclusions about this genome.Also, Supplementary table S4 lists the stats for the "Maker" gene set. Maker was not one of the listed tools used for gene prediction, so I am unsure exactly which gene set was used for this analysis. Also, looking at the data uploaded to the ftp site, only the "Maker" genes were provided. As far as I was aware, the final gene set should have been created using EvidenceModeler not Maker. Additionally, you list the results of functional annotation but don't provide the data anywhere. Please add it to the additional files associated with the manuscript.Page 15: What about the BUSCO scores for the predicted genes. How to they compare? You only list the genome stats in the results section but describe running BUSCO on the predicted genes in the methods.Page 16: "P. generosa and other 11 species " should read "P. generosa and 11 other species"Page 16: "30,616 gene families were identified as single-copy and multiple-copy genes, unique paralogs, other orthologs, and unclustered genes" This sentence confused me. What are the definitions for each of these categories? They are not listed in the methods, so I am unable to assess

exactly what this all means. Also, the exact numbers of each category should be presented so that we know how many of each were produced by this analysis.Page 16: You state that "1749 gene families that are specific to P. generosa", however later in the paragraph you say that "2902 gene families that are specific to P. generosa". I am unsure why these number have changed or what the difference is between the two sets. The latter number is the one that is listed in Figure 3.Page 16: The number of gene families in Figure 3 that are common to the 4 species is listed as 6490 but in the text is stated as 6940. Is this a typo? Also, are these gene families specific to just this group and not the other species in your analysis? Or can they be shared with the other species as well? This is not fully clear from the text.Page 16: "326 single-copy orthologous gene families from these 12 species showed". You are talking about single-copy orthogroups with genes identified in each species right? Or just a subset? This is not clear from your methods or results sections.Page 16: It might be helpful for the reader if you add a shaded box (or another form of annotation) indicating where the bivalve clade is in the tree and how many chromosomes each species has. Since I don't think you explicitly list this for each of your species in the text.Page 18: Supplementary Table S6 does not appear to be available with the manuscript so I was unable to assess this section fully.Page 18: "there were a few significant enrichment pathways" I think should read "there were a few significant enriched pathways"Page 18: "revealed 166 pathways" Pathways or genes? I can't assess what is being tested without the supplementary table.Page 18: "mainly represented in 5 biochemical metabolic pathways of KEGG category…." Do you mean 5 metabolic KEGG categories? As it is written I am not sure exactly what you are trying to say in this sentence. Also, the high level KEGG categories are very general and not particularly informative, maybe just focus on the subcategories.Page 18: "Significantly expanded and contracted genes were found to be related to ovarian …." How can these categories that you list be significantly associated with both expanded and contracted gene families? If you are significantly expanded then they are unlikely to also come up as significantly contracted in your analysis?Page 18: "According to enrichment analyses…." I think it should read "According to the enrichment analyses….". Also, I think you should rephrase this section as it is a little unclear which of the categories that you are listing are from your analysis (if any of them are?) and which are from the literature. Currently, you are mostly just listing categories from the literature and I am not really sure how they relate to your analysis or the conclusions that you are trying to make about this particular species.Page 19: "Two significantly enriched pathwaFcys…." I think additional letters accidentally got added to the word "pathways".Page 20: "P. generosa is a highly complex species with a high heterozygosity of 1.37% and 57.99% repeat sequences in genome." I think this should be rephrased to something like "P. generosa is a complex species with a genome that has high heterozygosity (1.37%) and repetitive sequence content (57.99%).". Also, is 1.37% heterozygosity high? Compared to what?Page 19: "This study may provide a" Rephrase the last part of this section to something like "This study provides a high-quality genomic resource that will support future phylogenetic, evolution, adaptation, and immunological studies. Additionally, it will support the genetic breeding of geoducks.".Page 21: I was unable to find any of the ID that you list in the "Data Availability" section on NCBI. I assume they have not yet been made public?Page 22: Some of the abbreviations are formatted weirdly. For example, RAxML doesn't have a definition. Neither does Iso-seq. Please double check this section.Figure 5: What do the colors in the pie charts represent? The "combined change across lineages" pie chart is hard to interpret as there is no context for the colors or number given. Please add more detail into the figures and figure legends to help the reader interpret the data.

Level of Interest

Please indicate how interesting you found the manuscript: Choose an item.

Quality of Written English

Please indicate the quality of language in the manuscript: Choose an item.

Declaration of Competing Interests

Please complete a declaration of competing interests, considering the following questions:

- Have you in the past five years received reimbursements, fees, funding, or salary from an organisation that may in any way gain or lose financially from the publication of this manuscript, either now or in the future?
- Do you hold any stocks or shares in an organisation that may in any way gain or lose financially from the publication of this manuscript, either now or in the future?
- Do you hold or are you currently applying for any patents relating to the content of the manuscript?
- Have you received reimbursements, fees, funding, or salary from an organization that holds or has applied for patents relating to the content of the manuscript?
- Do you have any other financial competing interests?
- Do you have any non-financial competing interests in relation to this paper?

If you can answer no to all of the above, write 'I declare that I have no competing interests' below. If your reply is yes to any, please give details below.

I declare that I have no competing interests

I agree to the open peer review policy of the journal. I understand that my name will be included on my report to the authors and, if the manuscript is accepted for publication, my named report including any attachments I upload will be posted on the website along with the authors' responses. I agree for my report to be made available under an Open Access Creative Commons CC-BY license (http://creativecommons.org/licenses/by/4.0/). I understand that any comments which I do not wish to be included in my named report can be included as confidential comments to the editors, which will not be published.

Choose an item.

To further support our reviewers, we have joined with Publons, where you can gain additional credit to further highlight your hard work (see: https://publons.com/journal/530/gigascience). On publication of

this paper, your review will be automatically added to Publons, you can then choose whether or not to claim your Publons credit. I understand this statement.

Yes Choose an item.
