# Supplementary material for: Chromosome-level genome assembly of the Pacific geoduck Panopea generosa reveals major inter- and intrachromosomal rearrangements and substantial expansion of the copine gene family
Source: Gigascience. 2023 Dec 19;12:giad105. doi: 10.1093/gigascience/giad105 (PMC10729735; doi:10.1093/gigascience/giad105)
Supplement: giad105_Reviewer_1_Report_Revision_1 — Timothy Gordon Stephens, Ph.D. -- 6/27/2023 Reviewed [file giad105_reviewer_1_report_revision_1.pdf]

Reviewer Report

Title: Chromosome-level genome assembly of the Pacific geoduck Panopea generosa reveals major inter- and intra-chromosomal rearrangements and substantial expansion of the copine gene family

Version: Revision 1 **Date:** 6/27/2023

Reviewer name: Timothy Gordon Stephens, Ph.D.

Reviewer Comments to Author:

The authors have made a good attempt as address the comments on the previous version of the manuscript. While this version is a significant improvement, I still feel that there are outstanding issues that need to be addressed before it can be accepted for publication. Generally, the phrasing of the manuscript has been greatly improved, however, additional work is still required (some of which I have tried to detail in my specific comments below). Additionally, the authors have included some interesting now analysis in this version of the manuscript, however, these results are occasionally not well integrated into the text of the manuscript (some of the results in the results section could be reordered to improve the flow of the manuscript), do not always have a clear purpose (such as the Alphafold2 results), or are not described in the methods. I would suggest that the authors carefully review the new analysis to make sure it all fits within the manuscript. I have tried to give specific comments addressing each of these issues below.It would be nice if in the next round the authors added line numbers to the manuscript so that it is easier to call out parts of the manuscript.Page 3: "revealed major inter- and intra-chromosomal exchanges" - Do you mean "rearrangements" not "exchanges". "Exchanges" makes it sounds like the two species are swapping DNA (HGT), which I assume you don't mean.Page 3: "neuronal development, immune response" should probably read "neuronal development and immune response". The comma is a little confusing.Page 5: "(which can be as long as 168 years) for P. generosa [4]"I assume it should read "(which can be as long as 168 years for P. generosa [4])"Page 6: "recombination in evolution in bivalves" should probably read "recombination during the evolution of bivalves"Page 6: "Nevertheless, high-quality chromosome-level" should probably read "Nevertheless, a high-quality chromosome-level"Page 7: "The availability of the genome information" should probably read "The availability of this genome information"Page 7: I am not sure how the results in Table 1 support the sentence "The genome size of P. generosa was estimated to be 1.47 Gb using k-mer analysis (Table 1) [22]."Nothing in that table describes genome size or any genome-related statistics.Page 7: I still feel that the method that the authors used to estimate genome size is not adequately described. In their response to my comment on the previous version of the manuscript the authors mentioned that Jellyfish was used for k-mer enumeration, however, once you have decomposed the data into k-mers you still need to use additional tools (such as GenomeScope) or formula (k-mer count / homozygous peak coverage) to produce the final genome size/heterozygosity/repeat content estimations that the authors cite.Additionally, the authors quote heterozygosity and repeat sequence proportion values but don't indicate how they were estimated from the enumerated k-mers (i.e., using which tool or formula). More detail about k-mer analysis needs to be provided in the methods.Page 7: "The heterozygosity of P.

generosa was comparable to those of most bivalves (Supplementary Table S3)." You compared the value from your genome to those of published genomes and came to what conclusion? This sentence doesn't really add anything to the results since there is no take away from it. Please elaborate on how your heterozygosity compares to that of the published genomes.Also, it would be best to add reference to the genomes in Table S3, to acknowledge the authors whose data you are citing.Page 7: I might suggest that "Genome assembly using PacBio long reads (N50 = 26,513 bp) and Falcon assembler obtained an initial size of 1.51 Gb. Further assembly using Hi-C data obtained a genome with 19 pseudomolecules, suggesting 19 chromosomes of the P. generosa genome (Figure 1A), with an anchoring rate of 94.70%. This genome assembly has a total length of 1,474,161,289 bp with a contig N50 of 1.57 Mb and a scaffold N50 of 73.79 Mb (Figure 1B; Table 2; Supplementary Table S4). As expected, the genomic regions with low gene density typically had high repeat content, while the regions with high repeat content usually had high GC content."Should read something like:"The genome assembly produced using PacBio long reads (N50 = 26,513 bp) and the Falcon assembler had an initial size of 1.51 Gb. Further refinement using Hi-C data (anchoring rate of 94.70%) produced an assembly with 19 pseudomolecules, putatively the 19 chromosomes predicted in P. generosa (Figure 1A). This genome assembly has a total length of 1,474,161,289 bp with a contig N50 of 1.57 Mb and a scaffold N50 of 73.79 Mb (Figure 1B; Table 2; Supplementary Table S4). As expected, the genomic regions with low gene density typically had high repeat content, while the regions with high repeat content typically had high GC content."Page 7: Great work with Table S1. A very comprehensive picture of the available genomes. Why do some of the genomes not have references? Are they unpublished? If so, then you should state the location where you downloaded/accessed the data.Page 8: "The majority (57.99%) of the P. generosa genome was repetitive elements estimated using de novo searching and homolog prediction (Table 3). Distribution of these repetitive elements was uneven with repetitive content per 1 Mb varied from 34.76% to 84.89% (Figure 1B)."Should probably read:"The majority (57.99%) of the P. generosa genome was estimated, using de novo searching and homolog prediction, to be repetitive elements (Table 3). Distribution of these repetitive elements was uneven across the genome, with repetitive content per 1 Mb varying from 34.76% to 84.89% (Figure 1B)."Table 3: It's a little bit repetitive having the "Reparative sequence" and "Transposable elements" columns with almost all the same rows. Potentially just show the "Repetitive sequence" column.Also, the "Total" values do not add up correctly (the Total "% in genome" for the "Reparative sequence" column should be 70.19% by my calculations, not 57.99%). Please double check all values.Page 8: "Of these PCGs, 30,700 genes were annotated to contain conserved functional motifs (Supplementary Table S5, Table S7)." Should probably read "Of these PCGs, 30,700 were annotated with conserved functional motifs (Supplementary Table S5, Table S7)."Page 8: "and annotated protein-coding gene set were". Just the annotated protein-coding genes? Or all protein-coding genes?Also, can you use the "PCG" acronym here?Page 8: "To evaluate the completeness of the assembly, the P. generosa genome assembly and annotated protein-coding gene set were assessed using BUSCO [23] with the metazoa_odb10 database (954 core genes), respectively."This sentence is a little confusing. What is the "respectively" referring to since there are not to elements at the start and end of the sentence that you are associating together. Please rephrase.Page 8: "Regarding the gene set,"Can these genes be referred to as "PCGs"?Page 8: "Although the P. generosa genome has 19 chromosomes as many other species in the order Adapedota" should probably read "Whereas the P. generosa genome has 19 chromosomes, the same number as in other

species in the order Adapedota"Page 9: "whose genome has been assembled at the chromosome-level revealed that 8these two genomes have good chromosomal collinearity in general" should probably read "whose genome has been assembled at the chromosome-level, revealed that in general these two genomes have good chromosomal collinearity"Page 9: "clear one-to-one r correspondences S. constricta chromosomes" should probably read "clear one-to-one r correspondence with S. constricta chromosomes"Also, what is "r correspondence"? I am unfamiliar with that term.Page 9: Would the term "rearrangements" be more appropriate than "exchanges" to use throughout the manuscript?Page 9: "also revealed that extensive intra-chromosomal" should probably be "also revealed extensive intra-chromosomal"Page 9: Sorry, I still find the description of the shared and specific gene families confusing. You state that there are two different groups of gene families that are specific to P. generosa. I assume the 2902 set is considering just the three other species shown in Figure 4?Also, why were these three other species chosen for analysis and visualization in Figure 4?Why focus on just these species in Figure 4? You use all species for the orthogroup analysis, what benefit does focusing on just these four add to the analysis/manuscript?Page 10: "identified in P. generosa genome" should probably read "identified in the P. generosa genome"Page 10: "166 pathways from the expanded gene" Some of those pathways at the end of your list have large p- and q-values. I would suggest filtering the data using a reasonable cutoff.Page 10: "(Supplementary Table S9) The" should probably read "(Supplementary Table S9). The"Page 10: "Fluid shear stress and atherosclerosis, Phosphatidylinositol signaling system, suggesting their important contribution to the adaptation of benthic bivalves."This part of the sentence is a little confusing. You are listing a lot of terms here and the sentence doesn't flow well as a result. Consider rephrasing.Also, are you missing an "and" at the end of the list?Page 10: "which were found related to" should probably read "which have been shown to function as part of"Page 11: "Examination of the top 65 domains of gene families that presented expansions in P. generosa (Figure 7A) showed that the gene numbers of many important gene families were substantially expanded in P. generosa, including these containing the GIY-YIG catalytic domain (PF01541), the caspase recruitment domain (PF16739), the ApoA/ApoE domain (PF01442), and the copine domain (PF07002)."This sentence is confusing. You are looking at the expanded gene families, of course they are"substantially expanded in P. generosa", that is why you are looking at them right? Please rephrase.Also, are the domains that you list the top most abundant or just the ones that you found most interesting? This is unclear.Page 11: "In particular, the copine gene family,…."This paragraph opens with a statement about InterProScan, but then only talks about Pfam domains and gene copy expansion. This whole section could be reordered so that your results about gene family expansion are together (up with the paragraph starting "A total of 507 expanded gene families"), and the functional results (Pfam + KEGG) are together.Page 11: "Interestingly, many genes formed local clusters"This is very interesting. Do you see a single copy of these genes in roughly the same chromosomes in S. constricta? That is, does S. constricta have the "original" gene from each of the local clusters in its chromosomes or have these genes also spread throughout the P. generosa genome and then expanded locally.Also, is this a common mechanism for gene family expansion? i.e., do the other genes that show expansion in this species also have identifiable local clusters?Figure 7: Double check the figure legend. I think the captions for B and C are switched?In the text you describe these genes as the "copine genes", but in the figure legend you say that they are "genes annotated to PF07002 domains". Are these the same or are these different trees? This need to be clarified/consistent terminology used.What are the support values for each of the nodes in the tree?

Do the genes group based on their location across the chromosome? I would assume so since they are local duplications but it might be good to annotate or indicate where in the tree the genes from these local clusters fall.Page 12: "as for copine genes identified in other species"As for what? This sentence is phrased like you are going to say something else but don't. Do you mean "like for copine genes identified in other species"?Page 12: "imperfect annotation" Do you mean "imperfect prediction"?"Annotation" usually means "functional annotation". If you are talking about gene structure issues you are usually talking about "gene prediction".Page 12: "Through the construction of the first high-quality chromosome-level genome assembly of the ecologically and economically important bivalves the Pacific geoduck P. generosa with cutting-edge genomic technologies, important insights into its genetic makeup and evolution have been gained.Should probably read:"The construction of the first high-quality chromosome-level genome assembly using cutting-edge technologies of the ecologically and economically important bivalve, the Pacific geoduck P. generosa, provides important insights into its genetic makeup and evolution.Page 12: "The assembled genome size consists of 19 chromosomes with a genome size of 1.47 Gb, and a contig N50 of 1.6 Mb and 19 chromosomes."Should probably read:"The assembled genome consists of 19 chromosomes, has a size of 1.47 Gb, and a contig N50 of 1.6 Mb."Page 12: "and the conservation of PCGs in bivalves."Does this show the conservation of PCGs in bivalves? I would think only your orthogroup analysis would do that, not the gene prediction/annotation workflow.Pahe 12-13: "P. generosa is a highly complex species with a heterozygosity of 1.37% and 57.99% repeat sequences in genome."Does this make it complex? Compared to what? It seems pretty standard compared to other bivalves. Please justify or remove.Page 13: "Large chromosomal fragments of P. generosa Pg02 and Pg11 matched to two S. constricta chromosomes (Chr1 and Chr10) and two S. constricta chromosomes (Chr1 and Chr10), respectively (Figure 2)."This sentence repeats the phrase "two S. constricta chromosomes (Chr1 and Chr10)", please rephrase.Page 13 and 14: It is unclear how important it is that the copine genes contain C2 and vWA-domains. Slightly more context for this statement is required to help the reader understand why this is interesting.Also, what is the significance of the Alphafold2 results? It is unsurprising that homologous genes would have highly similar structures. Unless there is something else that these results add to the manuscript I would suggest removing the Alphafold2 results and discussion. Additionally, the Alphafold2 work is not described in the methods.Page 17: "The Hi-C heatmap was visualized using Juicebox (RRID:SCR_021172) presenting the counts of paired reads which each two bins aligned (with the bin length of 100 kb) as the interactive signals between each pair of two bins."This sentence is a little confusing. Please consider rephrasing to improve readability.Page 17: "version5.4.3) [23]. using the"Remove the full stop.Page 18: "Exonerate v2.2.0"Can Exonerate take hits and reconstruct genes? My understanding is that Exonerate (by default) does the alignment for you and builds the gene from that. If there is a specific functionality of Exonerate that take pre-computed hits then this needs to be detailed.Page 18: I assume you used pre-trained models for Augustus and SNAP de novo prediction? If so, which models?Page 19: "procedures described previous studies" should probably read "procedures described in previous studies"Page 20: I see no new methods describing the InterProScan, Alphafold2, phylogenetic analysis (Figure 7C), or synteny/chromosome reorganization analysis.The methods for these new analyses need to be extensively described.Figures: Please make sure that you adequately describe the figure in each figure legend. For example, the figure legend for Figure 2 does not describe each panel (A and B), the panels in Figure 7 maybe mislabeled, etc.

Level of Interest

Please indicate how interesting you found the manuscript: Choose an item.

Quality of Written English

Please indicate the quality of language in the manuscript: Choose an item.

Declaration of Competing Interests

Please complete a declaration of competing interests, considering the following questions:

- Have you in the past five years received reimbursements, fees, funding, or salary from an organisation that may in any way gain or lose financially from the publication of this manuscript, either now or in the future?
- Do you hold any stocks or shares in an organisation that may in any way gain or lose financially from the publication of this manuscript, either now or in the future?
- Do you hold or are you currently applying for any patents relating to the content of the manuscript?
- Have you received reimbursements, fees, funding, or salary from an organization that holds or has applied for patents relating to the content of the manuscript?
- Do you have any other financial competing interests?
- Do you have any non-financial competing interests in relation to this paper?

If you can answer no to all of the above, write 'I declare that I have no competing interests' below. If your reply is yes to any, please give details below.

I declare that I have no competing interests

I agree to the open peer review policy of the journal. I understand that my name will be included on my report to the authors and, if the manuscript is accepted for publication, my named report including any attachments I upload will be posted on the website along with the authors' responses. I agree for my report to be made available under an Open Access Creative Commons CC-BY license (http://creativecommons.org/licenses/by/4.0/). I understand that any comments which I do not wish to be included in my named report can be included as confidential comments to the editors, which will not be published.

Choose an item.

To further support our reviewers, we have joined with Publons, where you can gain additional credit to further highlight your hard work (see: https://publons.com/journal/530/gigascience). On publication of

this paper, your review will be automatically added to Publons, you can then choose whether or not to claim your Publons credit. I understand this statement.

Yes Choose an item.
