# Supplementary material for: Chromosome-level genome assembly of the Pacific geoduck Panopea generosa reveals major inter- and intrachromosomal rearrangements and substantial expansion of the copine gene family
Source: Gigascience. 2023 Dec 19;12:giad105. doi: 10.1093/gigascience/giad105 (PMC10729735; doi:10.1093/gigascience/giad105)
Supplement: giad105_Reviewer_1_Report_Revision_2 — Timothy Gordon Stephens, Ph.D. -- 9/27/2023 Reviewed [file giad105_reviewer_1_report_revision_2.pdf]

Reviewer Report

Title: Chromosome-level genome assembly of the Pacific geoduck Panopea generosa reveals major inter- and intra-chromosomal rearrangements and substantial expansion of the copine gene family

Version: Revision 2 **Date:** 9/27/2023

Reviewer name: Timothy Gordon Stephens, Ph.D.

Reviewer Comments to Author:

The authors have done a good job at address my comment on the previous version of their manuscript. While I am generally happy with the state of the manuscript, I still have some minor grammatical issues that can be easily addressed by the authors. Additionally, I agree with Reviewer 2 that the Discussion section is very repetitive of the Results section and adds very little new insight to the manuscript. I would suggest, if possible, that the Discussion be rephrased to include a discussion of the significance of the results, or that it be reduced to reduce repetition.L72-73: "These genomes can provide a resource for comparative genomic for gaining evolutionary and other insights of bivalves and even molluscs" TO "These genomes can provide a resource for comparative genomics, and for gaining evolutionary and other insights into bivalves and molluscs"L95: "gene family expansion and contraction" TO "gene family expansion and contraction analysis"L128-131: "Of these PCGs, 30,700 (87.6%) were annotated with according to the public database including Nr, Swissprot, KEGG, KOG, TrEMBL, Interpro, and GO (Supplementary Table S5), suggesting the high quality of genome annotation." TO "Of these PCGs, 30,700 (87.6%) had functional annotations assigned using comparisons to public databases, including NCBIs nr, Swiss-Prot, KEGG, KOG, TrEMBL, InterPro, and GO (Supplementary Table S5), supporting the high quality of the genome."L133: "elements varied from" TO "elements varying from"L137: "93.0% of core PCGs were" TO "93.0% of the core genes used by BUSCO were"L138: ", higher than that" TO ", which is higher than that"L139: "of annotated PCGs using BUSCO, which were aligned to 88.4% of the full-length core orthologs, suggesting that quality of the annotation can be further improved in the future." TO "of the predicted PCGs using BUSCO, which identified 88.4% of the core genes as full-length, suggesting that quality of the predicted genes can be further improved in the future."L150: "chromosomes of these two species were both 19" TO "chromosomes in these two species is still 19"L154-155: "two species P. generosa and S. constricta," TO "two species (P. generosa and S. constricta),"L159: "orthologous genes of the 12" TO "orthologous genes from 12"L219-221: "while some has both C2-domain and the vWA-domain in P. generosa (Figure 7D) like copine genes identified in other species [33]." TO "however some have both a C2-domain and a vWA-domain in P. generosa (Figure 7D), like copine genes identified in other species [33]."L222: "due to errors of genome assembly or gene prediction" TO "due to genome assembly or gene prediction errors"L230: "important bivalves the Pacific geoduck P. generosa," TO "important bivalve, the Pacific geoduck P. generosa,"L235: "instance of chromosome-level genomes of the bivalve species" TO "instance of a chromosome-level genome assembly from a bivalve species"L259: "that the numbers of PCGs of many gene families" TO "that the number of PCGs in many gene families"L260: "substantially in evolution" TO "substantially during the

evolution of this group"L262: "genes were identified" TO "genes identified"L265-267: "composition of functional domains, which include C2 domain and vWA-domain (Figure 7D), and the similarity of three-dimensional structure to that of known copine genes (Figure 7E) suggests functional conservation" TO "composition of functional domains, which includes the C2 and vWA domains (Figure 7D), and the similarity of three-dimensional structure to that of known copine genes (Figure 7E), suggests conservation"L268: "completion of P. generosa facilitates genome comparative analysis" TO "completion of the genome P. generosa facilitates comparative analysis"L271: "construction of P. generosa chromosome-level genome" TO "construction of a chromosome-level genome assembly of P. generosa"L272: "in this organism, but also" TO "in this organism, but will also"I agree with Reviewer 2 that the discussion is very repetitive of the Results section and does not add much insight beyond what has already been said. If possible, I would advise the Authors to revise this section so that it provides a higher-level overview of the significance of their results.L281: "samples- were transferred to laboratory" TO "samples were transferred to the laboratory"L365: "PCGs in P. generosa" TO "PCGs in the P. generosa"L424: "family-wide P-Values were" TO "family-wide p-values were"

Level of Interest

Please indicate how interesting you found the manuscript: Choose an item.

Quality of Written English

Please indicate the quality of language in the manuscript: Choose an item.

Declaration of Competing Interests

Please complete a declaration of competing interests, considering the following questions:

- Have you in the past five years received reimbursements, fees, funding, or salary from an organisation that may in any way gain or lose financially from the publication of this manuscript, either now or in the future?
- Do you hold any stocks or shares in an organisation that may in any way gain or lose financially from the publication of this manuscript, either now or in the future?
- Do you hold or are you currently applying for any patents relating to the content of the manuscript?
- Have you received reimbursements, fees, funding, or salary from an organization that holds or has applied for patents relating to the content of the manuscript?
- Do you have any other financial competing interests?
- Do you have any non-financial competing interests in relation to this paper?

If you can answer no to all of the above, write 'I declare that I have no competing interests' below. If your reply is yes to any, please give details below.

I declare that I have no competing interests

I agree to the open peer review policy of the journal. I understand that my name will be included on my report to the authors and, if the manuscript is accepted for publication, my named report including any attachments I upload will be posted on the website along with the authors' responses. I agree for my report to be made available under an Open Access Creative Commons CC-BY license (http://creativecommons.org/licenses/by/4.0/). I understand that any comments which I do not wish to be included in my named report can be included as confidential comments to the editors, which will not be published.

Choose an item.

To further support our reviewers, we have joined with Publons, where you can gain additional credit to further highlight your hard work (see: https://publons.com/journal/530/gigascience). On publication of this paper, your review will be automatically added to Publons, you can then choose whether or not to claim your Publons credit. I understand this statement.

Yes Choose an item.
