# Supplementary material for: Chromosome-level genome assembly of the Pacific geoduck Panopea generosa reveals major inter- and intrachromosomal rearrangements and substantial expansion of the copine gene family
Source: Gigascience. 2023 Dec 19;12:giad105. doi: 10.1093/gigascience/giad105 (PMC10729735; doi:10.1093/gigascience/giad105)
Supplement: giad105_Reviewer_2_Report_Original_Submission — Ying Lu -- 3/20/2023 Reviewed [file giad105_reviewer_2_report_original_submission.pdf]

Reviewer Report

Title: Chromosome-level genome assembly of the Pacific geoduck Panopea generosa reveals major inter- and intra-chromosomal rearrangements and substantial expansion of the copine gene family

Version: Original Submission **Date:** 3/20/2023

Reviewer name: Ying Lu

Reviewer Comments to Author:

Comments to GIGA-D-22-00284The manuscript reports a chromosome-level genome of the Pacific geoduck Panopea generosa using Pacbio and Hi-C technologies. As an important species in both agriculture and ecology, the Panopea generosa genome is a valuable reference data for the study of clams. The quality of the assemblies seems not bad for the mollusk species. The current genome manuscript is publishable when a substantial revision is made.Comments:1. The final genome should be described more clearly. It is necessary to display the statistic information of each chromosomes' assemblies, such as length and number of unclosed gaps and contigs.2. Distribution of 17-mer occurrence reflects a high heterozygosity (double-peak distribution) in the genome, which are not suitable for estimation of its genome size (k-mer analysis suitable for the single peak k-mer distribution). Do the authors have any experimental evidence (e.g. flow cytometry analysis) to define the genome size? The author should give more reliable information for it.3. The authors said that the sequenced individual was collected in Strait of Georgia. How do the authors identify it as the Pacific geoduck P. generosa, mitochondrial sequencing or morphological characterization? It should be clarified.4. The phylogenetic analysis includes human, zebrafish and toad orthologs. The relationship between each of the three vertebrates and P. generosa are so remote that similarity among the homologs are low, which inevitably causes more errors in gene clustering. Besides, the authors use three time points to calibrate the speciation, C. elegans and H. sapiens (678.3-855.2 MYA), D. rerio and H. sapiens (413.1-443.0 MYA) and X. tropicalis and H. sapiens (347.0-357.9 MYA, all of which have the great evolution distance. These time points are not appropriate for the calculation of divergence time among clams. Also, the constructed phylogenetic tree does not have an outgroup. In short, the authors should redo the gene clustering with OrthoMCL by selecting most of the invertebrate species and use the time point among clams or mollusks in fossil calibration. An outgroup should be assigned in the constructed phylogenetic trees.5 The authors carry out the enrichment analysis of the expanded gene families. Actually, it is a tedious work, which do not make sense. Any survey genomes can do that. The chromosome-lever genome is supposed to exhibit more chromosome structure, such as gene collinearity or chromosome synteny among different related species, which is able to reflect not only assembly quality but also data importance. Most of the genome sequencing and assembling are completed by a commercial sequencing company, which means that the manuscript should include more analyzed information by the authors themselves. Referencing the recently-published papers in Gigascience, the manuscript should normally make one or more tries of evolutionary analyzation or gene expression profiling, not just the description of the assembly itself. Accordingly, the authors, on the one hand, should improve

this description by providing a couple of figures to illustrate the annotation of the expanded gene families,; one the other hand, construct the gene/chromosome synteny among 3 or more related species (illustrated as the circus image) to check the chromosome arrangement of evolutionary analysis.6. In main text, the BUSCO test indicates the genome completeness of 90.9%. However, in the supplementary files, the Hi-C BUSCO and annotation BUSCO test show the completeness at 93% and 88%, respectively. The authors should clarify it and provide the BUSCO test table in the manuscript.7. The English writing of the manuscript should be improved when a revision is submitted.

Level of Interest

Please indicate how interesting you found the manuscript: Choose an item.

Quality of Written English

Please indicate the quality of language in the manuscript: Choose an item.

Declaration of Competing Interests

Please complete a declaration of competing interests, considering the following questions:

- Have you in the past five years received reimbursements, fees, funding, or salary from an organisation that may in any way gain or lose financially from the publication of this manuscript, either now or in the future?
- Do you hold any stocks or shares in an organisation that may in any way gain or lose financially from the publication of this manuscript, either now or in the future?
- Do you hold or are you currently applying for any patents relating to the content of the manuscript?
- Have you received reimbursements, fees, funding, or salary from an organization that holds or has applied for patents relating to the content of the manuscript?
- Do you have any other financial competing interests?
- Do you have any non-financial competing interests in relation to this paper?

If you can answer no to all of the above, write 'I declare that I have no competing interests' below. If your reply is yes to any, please give details below.

I declare that I have no competing interests

I agree to the open peer review policy of the journal. I understand that my name will be included on my report to the authors and, if the manuscript is accepted for publication, my named report including any attachments I upload will be posted on the website along with the authors' responses. I agree for my report to be made available under an Open Access Creative Commons CC-BY license (http://creativecommons.org/licenses/by/4.0/). I understand that any comments which I do not wish to

be included in my named report can be included as confidential comments to the editors, which will not be published.

Choose an item.

To further support our reviewers, we have joined with Publons, where you can gain additional credit to further highlight your hard work (see: https://publons.com/journal/530/gigascience). On publication of this paper, your review will be automatically added to Publons, you can then choose whether or not to claim your Publons credit. I understand this statement.

Yes Choose an item.
