# Supplementary material for: Chromosome-level genome assembly of the Pacific geoduck Panopea generosa reveals major inter- and intrachromosomal rearrangements and substantial expansion of the copine gene family
Source: Gigascience. 2023 Dec 19;12:giad105. doi: 10.1093/gigascience/giad105 (PMC10729735; doi:10.1093/gigascience/giad105)
Supplement: giad105_Reviewer_2_Report_Revision_1 — Ying Lu -- 7/30/2023 Reviewed [file giad105_reviewer_2_report_revision_1.pdf]

Reviewer Report

Title: Chromosome-level genome assembly of the Pacific geoduck Panopea generosa reveals major inter- and intra-chromosomal rearrangements and substantial expansion of the copine gene family

Version: Revision 1 **Date:** 7/30/2023

Reviewer name: Ying Lu

Reviewer Comments to Author:

The authors improve the manuscript somewhat by adding more analyses of rapidly expended gene clusters and a couple of potential functional genes, as well as the chromosome synteny between two related species. As far as the whole manuscript is concerned, most of the studies in genomics or evolution analysis are so superficial that it cannot make much sense sometimes, except a chromosome-level genome assembly. In view of the data value of the genome assemblies, I think this manuscript is able to be published as a DATANOTE paper when the second round revision is done. The authors should revise the manuscript according to all the listed corrections and comments (D1ï¼D18) in the attached file. The final manuscript should also match the format of the DATANOTE paper. As to the terrible English writing (no improvement at all), I have tried to edit the abstract and result sections as possible. The discussion section should be re-written.

Level of Interest

Please indicate how interesting you found the manuscript: Choose an item.

Quality of Written English

Please indicate the quality of language in the manuscript: Choose an item.

Declaration of Competing Interests

Please complete a declaration of competing interests, considering the following questions:

- Have you in the past five years received reimbursements, fees, funding, or salary from an organisation that may in any way gain or lose financially from the publication of this manuscript, either now or in the future?
- Do you hold any stocks or shares in an organisation that may in any way gain or lose financially from the publication of this manuscript, either now or in the future?
- Do you hold or are you currently applying for any patents relating to the content of the manuscript?
- Have you received reimbursements, fees, funding, or salary from an organization that holds or has applied for patents relating to the content of the manuscript?

- Do you have any other financial competing interests?
- Do you have any non-financial competing interests in relation to this paper?

If you can answer no to all of the above, write 'I declare that I have no competing interests' below. If your reply is yes to any, please give details below.

I declare that I have no competing interests.

I agree to the open peer review policy of the journal. I understand that my name will be included on my report to the authors and, if the manuscript is accepted for publication, my named report including any attachments I upload will be posted on the website along with the authors' responses. I agree for my report to be made available under an Open Access Creative Commons CC-BY license (http://creativecommons.org/licenses/by/4.0/). I understand that any comments which I do not wish to be included in my named report can be included as confidential comments to the editors, which will not be published.

Choose an item.

To further support our reviewers, we have joined with Publons, where you can gain additional credit to further highlight your hard work (see: https://publons.com/journal/530/gigascience). On publication of this paper, your review will be automatically added to Publons, you can then choose whether or not to claim your Publons credit. I understand this statement.

Yes Choose an item.
